# Light-Triggered Catalytic Asymmetric Allylic Benzylation with Photogenerated *C*-Nucleophiles

**DOI:** 10.1021/acs.joc.0c00175

**Published:** 2020-02-21

**Authors:** Suva Paria, Edoardo Carletti, Michela Marcon, Alessio Cherubini-Celli, Andrea Mazzanti, Marzio Rancan, Luca Dell’Amico, Marcella Bonchio, Xavier Companyó

**Affiliations:** † Department of Chemical Sciences, University of Padova, via Marzolo 1, 35131 Padova, Italy; ‡ Department of Industrial Chemistry “Toso Montanari”, University of Bologna, viale del Risorgimento 4, 40136 Bologna, Italy; § ICMATE-CNR, Department of Chemical Sciences, University of Padova, via Marzolo 1, 35131 Padova, Italy

## Abstract

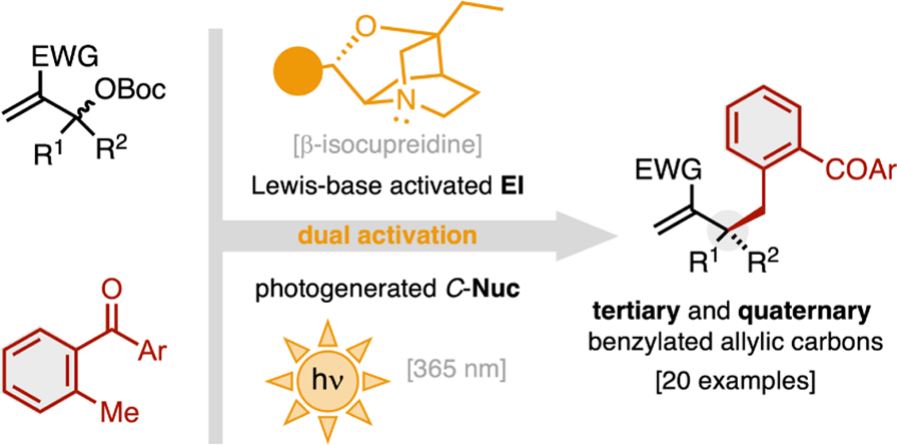

Herein is reported the asymmetric allylic benzylation of Morita–Baylis–Hillman (MBH) carbonates with 2-methylbenzophenone (MBP) derivatives as nonstabilized photogenerated *C*-nucleophiles. The dual activation of both reaction partners, chiral Lewis-base activation of the electrophile and light activation of the nucleophile, enables the stereoselective installation of benzyl groups at the allylic position to forge tertiary and quaternary carbon centers.

## Introduction

**Scheme 1:**
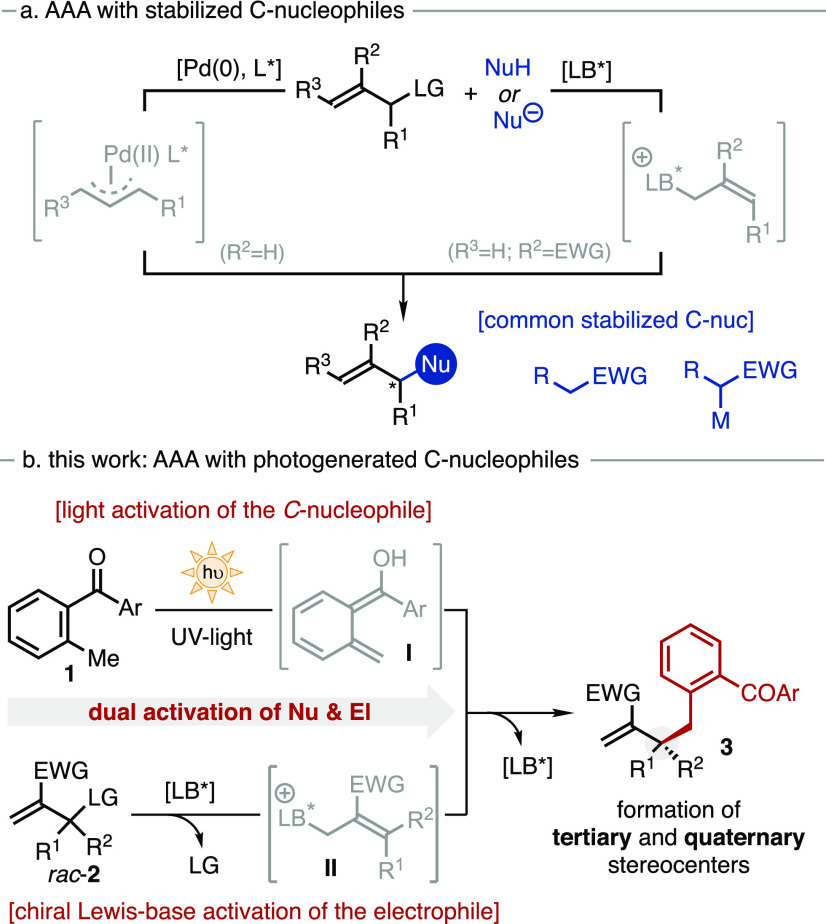
Catalytic Asymmetric Allylic Alkylation with (a) Stabilized Nucleophiles and (b) Photogenerated *C*-Nucleophile

The asymmetric allylic alkylation (AAA) reaction lies among the most powerful strategies for the catalytic construction of C–C bonds in a stereoselective manner. The pioneering works of Trost,^1^ together with the seminal reports by Kim and Lu^2^ established the foundations of both the transition-metal-catalyzed^3^ and the Lewis-base-catalyzed^4^ AAA reactions (Scheme 1a). Although extensive efforts have been devoted to expanding their generality and harnessing their asymmetric potential,^5−7^ these transformations are mainly restricted to the use of stabilized or acidic *C*-nucleophiles, such as alkali-metal salts,^5^ active methylene compounds,^6^ or enolates^7^ (Scheme 1a). Conversely, the use of nonacidic or nonstabilized nucleophiles remains largely underdeveloped,^8^ being particularly elusive in the AAA catalyzed by chiral Lewis bases.

Photochemistry is currently emerging as a complementary and more sustainable approach in synthetic chemistry. The unprecedented reactivity of excited organic molecules under light irradiation has tremendously expanded the playground for new reaction discovery.^9^ In 1961, Yang and Rivas first recognized the ability of 2-methylbenzophenone derivatives (MBP **1**, Scheme 1b) to generate, under UV-light irradiation, the fleeting photoenol intermediate **I**.^10^ However, its synthetic potential^11^ as nucleophile has only been successfully implemented in asymmetric catalysis very recently.^12^ In 2016, the [4 + 2]-cycloaddition between **I** and maleimides via H-bonding catalysis was reported.^12b^ Subsequently, the asymmetric Mannich-type reaction with cyclic imines^12c^ and the desymmetrization of 1,3-diketones through aldol addition were accomplished under the same activation strategy.^12d^ Interestingly, the stereoselective trapping of the photoenol intermediate (**I**) is not only restricted to H-bond donor activation. In 2017, the photoenol **I** was implemented in iminium-ion catalysis for its asymmetric conjugate additions to enals^12e^ and enones.^12f^

Herein is presented the asymmetric allylic benzylation of racemic Morita–Baylis–Hillman carbonates (MBH, **2**) with MBP derivatives **1** as nonstabilized *C*-pronucleophiles (Scheme 1b). The dual activation of both reaction partners, chiral Lewis-base activation of the electrophile and light activation of the nucleophile, enables the stereoselective installation of benzyl groups at allylic positions for the construction of tertiary and all-carbon quaternary stereocenters.

## Results and Discussion

MBH carbonate **2a** and MBP **1a** were chosen as the model substrates. The initial survey of nucleophilic catalysts in toluene identified tertiary amines **4** bearing an oxazatwistane ring as the more efficient catalyst type.^13^ β-Isocupreidine (**4a**, Table 1) in toluene furnished the benzylated product **3a** in 39% yield and 65:35 *er* (entry 1). Modifications in the catalyst scaffold did not relevantly improve the reaction outcome (e.g., Table 1, entries 2–4).^13^

**Table 1:**
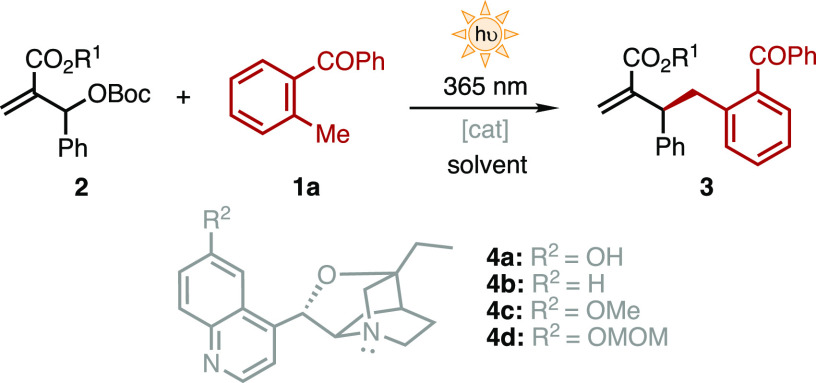
Screening of Reaction Conditions^a^^,^^b^

ent.	R^1^	cat (mol %)	solvent	yield (%)^c^	*er*^d^
1	Me (**2a**)	**4a** (20)	toluene	39	65:35
2	Me (**2a**)	**4b** (20)	toluene	10	64:36
3	Me (**2a**)	**4c** (20)	toluene	53	63:37
4	Me (**2a**)	**4d** (20)	toluene	42	65:35
5	Me (**2a**)	-	toluene	n.r.	-
6^e^	Me (**2a**)	**4a** (20)	toluene	n.r.	-
7	Me (**2a**)	**4a** (20)	CH_2_Cl_2_	37	55:45
8	Me (**2a**)	**4a** (20)	MeCN	21	59:31
9	Me (**2a**)	**4a** (20)	MeOH	65	73:27
10	Me (**2a**)	**4a** (20)	*t*-BuOH	44	67:33
11	Et (**2b**)	**4a** (20)	MeOH	32	75:25
12	*i*-Pr (**2c**)	**4a** (20)	MeOH	42	81:19
13	*t*-Bu (**2d**)	**4a** (20)	MeOH	9	90:10
14	Ph (**2e**)	**4a** (20)	toluene	60	76:24
15	1-Nap (**2f**)	**4a** (20)	toluene	62	80:20
16	2-Nap (**2g**)	**4a** (20)	toluene	63	78:22
17	2-Nap (**2g**)	**4a** (10)	toluene	65	78:22
18^f^	2-Nap (**2g**)	**4a** (10)	toluene	66 (62)	80:20

a Selected results.

b General conditions: a solution of **2** (0.1 mmol), **1** (0.5 mmol), and catalyst **4** in 1 mL of degassed solvent (0.1 M) was irradiated with a 9 W 365 nm light bulb for 3 h at 25 ± 2 °C.

c NMR yield. Isolated yields in brackets.

d Determined by chiral high-performance liquid chromatography (HPLC).

e No light irradiation.

f 4 Å MS (20 mg) were used. n.r.: no reaction.

No traces of product could be detected in the absence of either catalyst (entry 5) or light irradiation (entry 6), confirming the proposed dual activation strategy. Subsequently, different solvents were tested using the commercially available β-isocupreidine **4a** (entries 7–10). The best results were observed in MeOH, obtaining the benzylated product **3a** in 65% yield and 73:27 *er* (entry 9). Then, the effect of the ester group (R^1^) on the MBH carbonate **2** was systematically investigated. Increasing the bulkiness in R^1^ (entries 9, 11–13) improved the enantiomeric ratio of the benzylated product albeit with reduction in the yield. For instance, the *t*-Bu product **3d** was obtained in 90:10 *er* and 9% yield (entry 13). Aromatic substituents in the ester terminus were subsequently explored (entries 14–16). In these cases, toluene was used to avoid transesterification with the solvent. Gratifyingly, 1- and 2-naphthyl (**2f**–**g**) provided the corresponding products with good yields and enhanced enantiocontrol (entries 15–16). Further, reduction of the catalyst loading to 10 mol % (entry 17) together with the use of 4 Å molecular sieves (entry 18) allowed to isolate the benzylated product **3g** in 62% yield and 80:20 *er* (entry 18). Other reaction parameters including solvent mixtures, additives, different light sources, temperature, and concentration were exhaustively screened without further improvement.^13^

**Figure 1:**
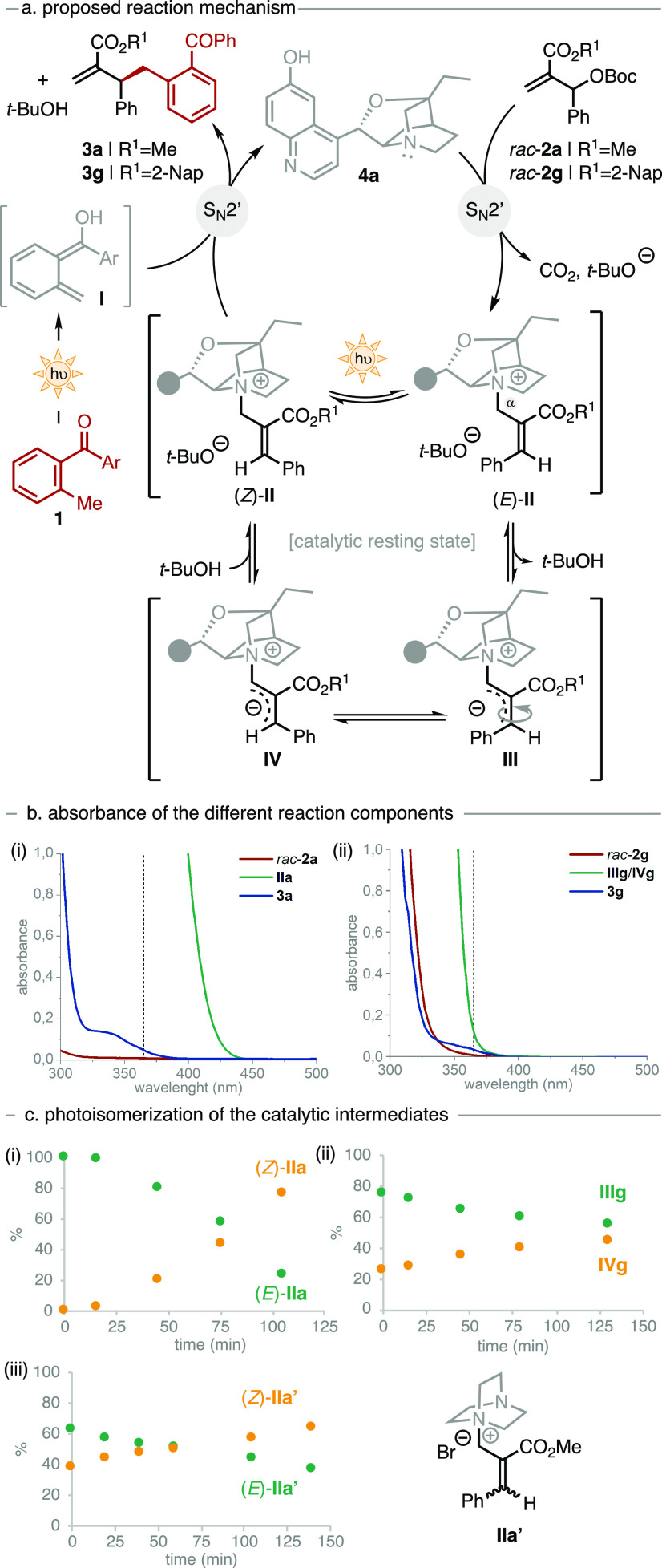
(a) Proposed reaction mechanism. (b) Absorbance of the different reaction components. (c) Light-mediated isomerization of the catalytic intermediates at 365 nm.

According to the extensive optimization results, we reasoned that the observed moderate enantiocontrol might not be due to inefficient shielding of the electrophilic double bond of **II** by the catalyst. Instead, alternative reaction pathways might be operative, such as photoisomerization of the catalytic intermediate **II**.^14^ In this scenario, even an effective shielding of the E/Z double bond on **II** would afford the product **3** in moderate *er* (Figure 1). To study the interaction of the catalytic system with light, two different model reactions were selected; the methyl ester derivative **2a** in MeOH and the 2-naphthyl ester substrate **2g** in toluene (Figure 1a). The quantitative formation of the catalytic intermediate (*E*)-**IIa** (Figure 1ci) was observed when an equimolecular mixture of **2a** and **4a** was dissolved in MeOH-*d*_3_. Conversely, when equal molar amounts of **2g** and **4a** were mixed in toluene-*d*_8_, a diastereomeric 75:25 mixture of the allylic ylides **IIIg** and **IVg** was formed (Figure 1cii), reflecting the registered *er* under catalytic conditions. The different nature of both catalytic species was confirmed by ^13^C NMR. While the Cα resonance of **IIa** appears at 54 ppm, the corresponding signal in **IIIg**/**IVg** appears at 126 ppm, confirming the sp^3^ character of the former and the sp^2^ character of the latter. This difference can be attributed to the presence of *t*-BuO^–^ along with the different nature of the solvent. In polar protic solvent, the equilibrium is completely shifted toward **II**, whereas in aprotic solvents, the strong base is able to deprotonate the allylic carbon (Cα) to generate the corresponding allylic ylides (**III** and **IV**). Subsequently, the absorbances of the different reaction components were measured (Figure 1b). While the starting materials **2a** and **2g** do not absorb at 365 nm (red lines), the corresponding products (**3a** and **3g**) start to absorb at 395 nm because of the presence of the benzophenone moiety (blue lines). Notably, both catalytic species (**IIa** and **IIIg**/**IVg**) strongly absorb at the operative wavelength (green line). Owing to the presence of the conjugated system, **IIa** presents a redshift absorbance up to the visible region (450 nm), while the absorbance of **IIIg**/**IVg** is confined below 400 nm. In situ monitoring of the reaction evolution by semicontinuous ^1^H NMR^15^ revealed that the catalytic intermediates **IIa** and **IIIg**/**IVg** are the corresponding catalytic resting states. Therefore, the presence of these species in significant concentration during the reaction course along with their absorbance profiles makes them susceptible to further light-triggered processes. Indeed, when a solution of diastereopure (*E*)-**IIa** in MeOH-*d*_3_ was irradiated at 365 nm, isomerization toward the intermediate (*Z*)-**IIa** took place within 2 h (Figure 1ci). Similarly, irradiation of a solution of **IIIg**/**IVg** in toluene-*d*_8_ led to a change in the diastereomeric ratio, albeit at a slower rate (from 75:25 to 55:45, Figure 1cii). To exclude the possibility of a base-mediated isomerization, the bromide salt **IIa′** containing a nonbasic counteranion was synthesized as an E/Z mixture (Figure 1ciii).^16^ A 365 nm irradiation of a methanolic solution of **IIa′** led to the isomerization of the double bond from *E*/*Z* = 62:38 to 37:63, confirming the direct photoisomerization of the catalytic species.^14^ The slower isomerization of the catalytic intermediate **IIIg**/**IVg** compared to **IIa** explains the enhanced asymmetric induction obtained with naphthyl ester MBH carbonates (**2f** and **2g**).

**Scheme 2:**
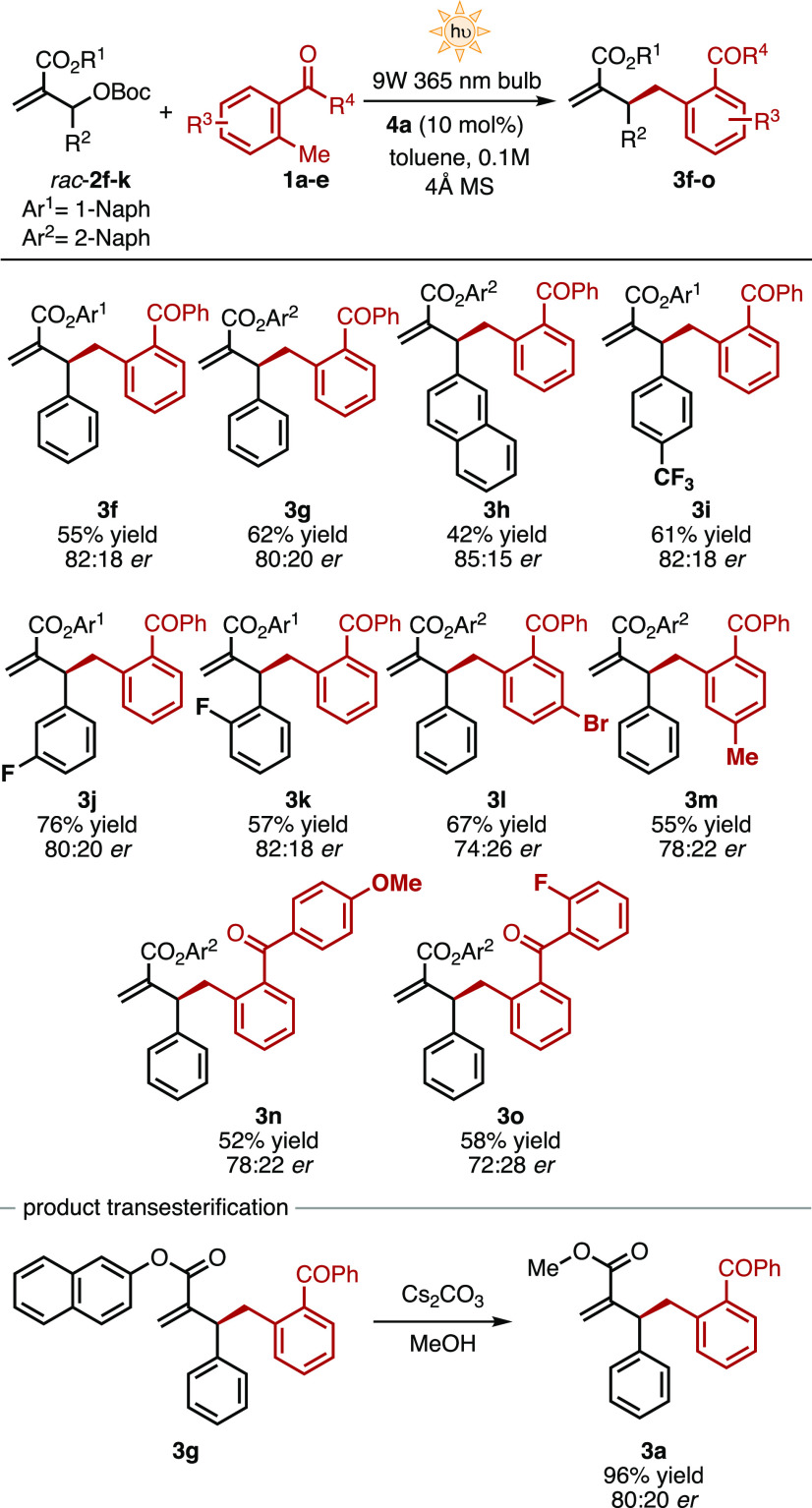
Light-Triggered Catalytic Asymmetric Allylic Benzylation of MBH Carbonates 2 for the Formation of Tertiary Allylic Carbons General conditions: a solution of **2** (0.1 mmol), **1** (0.5 mmol), 10 mol % of **4a** (0.01 mmol), and 20 mg of 4 Å MS in 1 mL of degassed toluene (0.1 M) was irradiated with a 9 W 365 nm bulb at 25 ± 2 °C.

At this point, we analyzed how steric and electronic variations on the structure of both reaction partners (**1** and **2**) impact the reaction outcome. Hence, MBH carbonates bearing a naphthyl ester group catalyzed by 10 mol % of **4a** in toluene under 9 W 365 nm bulb irradiation were selected as the most suitable reactants (Scheme 2). Substitution in the aromatic ring of the MBH carbonate (R^2^ in **2**) with electron-withdrawing groups in different positions is well tolerated. Benzylated products bearing *p*-CF_3_ (**3i**), *m*-F (**3j**), and *o*-F (**3k**) groups were obtained in good yields (57–76%) maintaining the optimized asymmetric induction (80:20–82:18 *er*). The best result in terms of enantiocontrol was obtained when a 2-naphthyl group was placed in position R^2^, affording the product **3h** in 85:15 *er*.

Subsequently, the effect of substitution on the MBP pronucleophile **1** was investigated. Electron-donating and electron-withdrawing substituents are tolerated in both aromatic rings (R^3^ and R^4^ in **1**). However, the final products are generally obtained in lower enantiomeric ratios compared to the unsubstituted MBP (**1a**). Electron-poor substituents such as Br and F furnished the final products **3l** and **3o** in 67 and 58% yields and 74:26 and 72:28 *er*, respectively, while electron-rich groups such as Me and MeO provided the benzylated products in lower yields and superior enantioselectivity (55% yield and 78:22 *er* for **3m**; 52% yield and 78:22 *er* for **3n**).To expand the versatility of the benzylated products synthesized, we then studied the interconversion of the ester group from naphthyl to the more common methyl group. The simple treatment of the product **3g** in a basic solution of MeOH furnished the corresponding methyl ester-derived product **3a** in 96% yield without any erosion in the enantiomeric purity (80:20 *er*, Scheme 2).

**Scheme 3:**
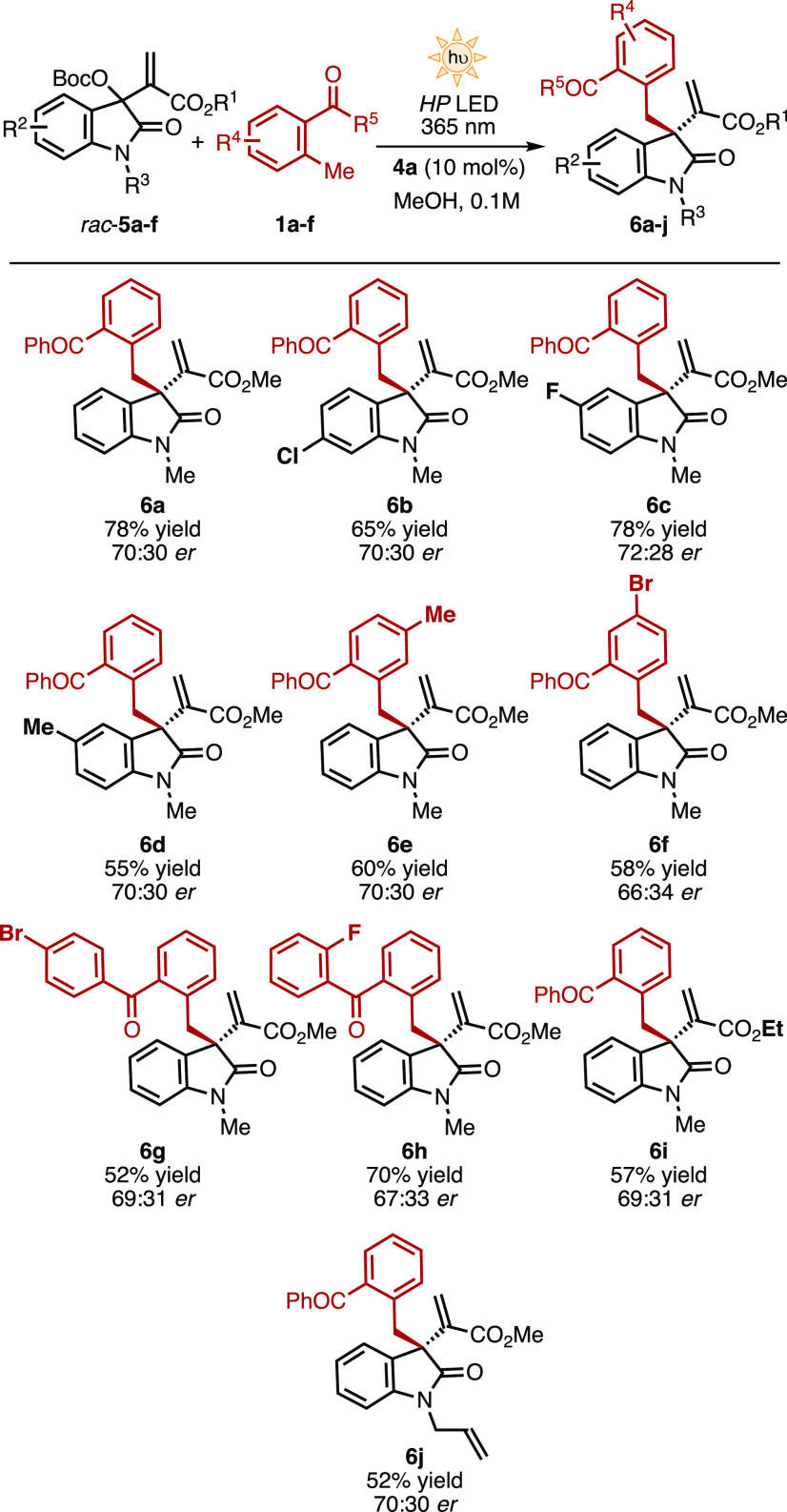
Light-Triggered Catalytic Asymmetric Allylic Benzylation of Isatin-Derived MBH Carbonates (5) for the Formation of Quaternary Allylic Carbons General conditions: a solution of **5** (0.1 mmol), **1** (0.5 mmol), and 10 mol % of **4a** (0.01 mmol) in 1 mL of degassed MeOH (0.1 M) was irradiated with a high-power 365 nm LED at 350 mA at 25 ± 2 °C.

Subsequently, we investigated the possibility of constructing all-carbon quaternary allylic stereocenters (Scheme 3). Isatin-derived MBH carbonates (**5**) have mainly been employed in [3 + 2] cycloadditions under Lewis-base catalysis,^4^ and only few examples account for the direct installation of nucleophiles in allylic position.^17^

The light-triggered benzylation of **5** (Scheme 3) represents a direct entry for the assembly of 3,3-disubstituted oxindoles,^18^ a scaffold present in several natural products and pharmaceuticals possessing a wide range of biological activities.^19^ The initial screening assessed the nature of the amide-protecting group and the ester group of **5**. Aliphatic groups in both positions were found to be more suitable in terms of starting material and product stability.^13^ Subsequently, the diverse reaction parameters were carefully evaluated. The best results were obtained employing 10 mol % of β-isocupreidine **4a** in MeOH under high-power 365 nm light-emitting diode (LED) irradiation at 350 mA, affording **6a** in 78% yield and 70:30 *er*. As in the case of linear substrates **2**, the isomerization of the catalytic intermediate was found to be responsible for the moderate asymmetric induction.^13^ Groups with diverse electronic characteristics are well tolerated in the electrophile (**5**), providing the benzylated products **6b**–**d** in 55–78% yield. Also, electron-rich and electron-poor substituents in both aromatic rings of the MBP derivative (**1**) provided the corresponding products **6e**–**h** in moderate to good yields (52–70%). Other aliphatic ester and amide-protecting groups can also be accommodated in **5**, such as the ethyl ester-derived **6i** (57% yield) and the *N*-allyl product **6j** (52% yield), providing structural diversity. Nevertheless, the 3,3-disubstituted benzylated oxindoles **6a–j** were isolated in moderate enantiomeric ratios (66:34–72:28 *er*).

One of the main pitfalls associated with synthetic photochemical methodologies is the troublesome scaling up. Being photons the key reagent, it is not obvious how to vary the equivalents of photons in the same extent when the scale of a certain photochemical transformation is changed. As a result, further optimizations are generally required, including changes in the light source, light intensity, and reaction setup. In this scenario, microfluidic photochemical technology offers a compelling opportunity to address this issue,^20^ enabling an increased light penetration and a more uniform and effective irradiation along with an easy upscaling by continuous-flow synthesis. Hence, we implemented the light-triggered asymmetric allylic benzylation of MBH carbonate **5** with 2-methylbenzophenone (**1a**) catalyzed by **4a** under a microfluidic photoreactor (MFP).^11c−11e^

**Scheme 4:**
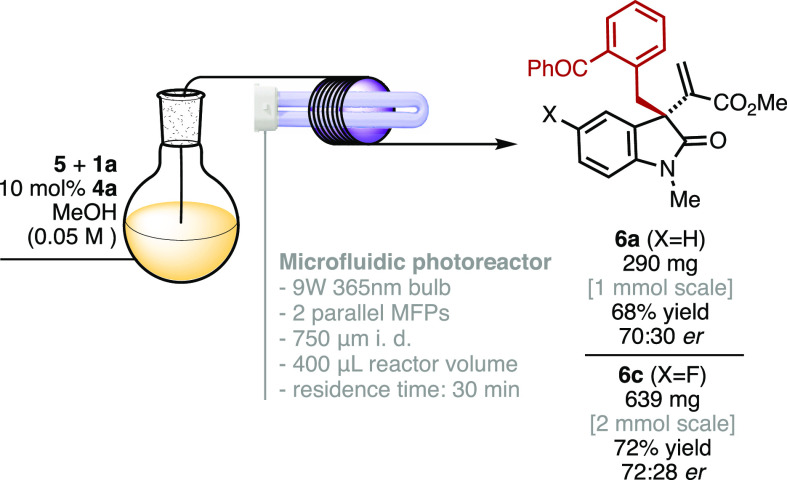
Implementation of the Light-Triggered Catalytic Asymmetric Allylic Benzylation into a Microfluidic Photoreactor (MFP) for Large-Scale Continuous-Flow Synthesis

Scheme 4 shows the general representation of the MFP, which is composed of a poly(tetrafluoroethylene) (PTFE) tubing wrapped around a U-shaped 9 W 365 nm bulb lamp with an inner reactor volume of 400 μL. The different reaction parameters including residence time, concentration, catalyst loading, and reagents stoichiometry were screened at 0.01–0.05 mmol scale.^13^ The best results were obtained using 10 mol % of catalyst **4a** at 0.05 M concentration with a residence time of 30 min. These conditions were used to scale up the present catalytic photochemical transformation in continuous-flow synthesis (Scheme 4). The light-triggered asymmetric allylic benzylation of **5a** at 1 mmol scale was conducted under two parallel MFP setups for 13 h, furnishing 290 mg of the benzylated product **6a** (68% yield) maintaining the registered enantiomeric ratio (70:30 *er*). Further, the presented transformation was successfully scaled 20-fold up under microfluidic conditions, producing 639 mg of product **6c** (72% yield, 72:28 *er*) in 25 h. Therefore, by simply extending the continuous-flow synthesis time, the final benzylated product can be easily produced in the desired scale.

The absolute configurations of the products **3a** and **6a** were ascertained by means of time-dependent density-functional theory (TD-DFT) calculations of the electronic circular dichroism (ECD) spectra, and the rest of products (**3f**–**o** and **6b–j**) were assigned by analogy.^13^

## Conclusions

In summary, the light-triggered asymmetric allylic benzylation of MBH carbonates **2** and **5** is presented. The dual activation of both reaction partners enables the utilization of 2-methyl benzophenone derivatives (**1**) as nonstabilized, photogenerated *C*-pronucleophiles. The final benzylated products (**3** and **6**), bearing tertiary and quaternary allylic stereocenters, are obtained in good yields (up to 78%) and moderate to good enantioselectivities (up to 85:15 *er*). Mechanistic investigations revealed that the light-mediated isomerization of the electrophilic catalytic intermediates is the main cause of the observed moderate asymmetric induction. Notably, the present methodology can be implemented into a microfluidic photoreactor setup for large-scale continuous-flow synthesis.

## Experimental Section

### General Directions

Commercial grade reagents and solvents were purchased at the highest commercial quality from Sigma-Aldrich, TCI, and Fluorochem and used as received, unless otherwise stated. Chromatographic purification of products was accomplished using flash chromatography on silica gel (SiO_2_, 0.04–0.063 mm) purchased from Macherey-Nagel, with the indicated solvent system according to the standard techniques. Thin-layer chromatography (TLC) analysis was performed on precoated Merck TLC plates (silica gel 60 GF254, 0.25 mm). Visualization of the developed chromatography was performed by checking UV absorbance (254 nm) as well as with aqueous potassium permanganate solutions. Organic solutions were concentrated under reduced pressure on a Büchi rotary evaporator. NMR spectra were recorded on a Bruker Avance DPX 200 equipped with a QNP probehead, Bruker 400 Avance III HD equipped with a BBI-z grad probehead 5 mm, and a Bruker 500 Avance III equipped with a BBI-ATM-z grad probehead 5 mm. The chemical shifts (δ) for ^1^H and ^13^C are given in ppm relative to residual signals of the solvents (CDCl_3_@7.26 ppm ^1^H NMR, 77.16 ppm ^13^C NMR). Coupling constants are given in hertz. The following abbreviations are used to indicate the multiplicity: s, singlet; d, doublet; t, triplet; q, quartet; m, multiplet; bs, broad signal. NMR yields were calculated by using pyrazine as internal standard. HPLC analysis on chiral stationary phase was performed on a UHPLC Agilent 1290 Infinity, using Phonemenex Lux 5u Cellulose-1, Lux 5u Cellulose-4, and Lux 5u Cellulose-5 chiral columns. The exact conditions for the analyses are specified within the characterization section. HPLC traces were compared to racemic samples prepared performing the reactions using DABCO as catalyst. High-resolution mass spectra (HRMS) were obtained using Waters GCT gas chromatograph coupled with a time-of-flight mass spectrometer (GC/MS-TOF) with electron ionization (EI) or MicroTOF II (Bruker Daltonics): HPLC-MS-TOF (ESI). The continuous-flow reactions were carried out using capillary reactors made with PTFE tubing (0.75 mm ID, 1.58 mm OD), and fitting connections were purchased from Sigma-Aldrich. Reagents were pumped using a Syrris Asia pump, and 9 W 365 nm bulb lamps were purchased from Amazon. High-power 365 nm LEDs were purchased from OSA Opto Light GmbH.

### Synthesis of Starting Materials

2-Methylbenzophenone (MBP) derivatives **1a** and **1c** were purchased at the highest commercial quality from Sigma-Aldrich and used as received. MBP derivatives **1b**, **1d**–**f** were synthesized as described in the literature.^11^

#### (5-Bromo-2-methylphenyl)(phenyl)methanone (**1b**)

Synthesized following a reported procedure^12b^ as a pale yellow oil (1.02 g, 82% yield) after flash chromatography on silica gel using petroleum ether/EtOAc 98:2 as eluent. ^1^H NMR (400 MHz, CDCl_3_): δ 7.85–7.78 (m, 2H), 7.62 (ddt, *J* = 8.7, 6.9, 1.3 Hz, 1H), 7.55–7.42 (m, 4H), 7.19 (dd, *J* = 8.2, 0.8 Hz, 1H), 2.27 (s, 3H).

#### (4-Methoxyphenyl)(*o*-tolyl)methanone (**1d**)

Synthesized following a reported procedure^12b^ as a pale yellow oil (649 mg, 48% yield) after flash chromatography on silica gel using petroleum ether/EtOAc 95:5 as eluent. ^1^H NMR (400 MHz, CDCl_3_): δ 7.85–7.77 (m, 2H), 7.43–7.35 (m, 1H), 7.33–7.21 (m, 4H), 6.99–6.89 (m, 2H), 3.89 (s, 3H), 2.33 (s, 3H).

#### (2-Fluoroxyphenyl)(*o*-tolyl)methanone (**1e**)

Synthesized following a reported procedure^12b^ as a pale yellow oil (380 mg, 44% yield) after flash chromatography on silica gel using petroleum ether/EtOAc 98:2 as eluent. ^1^H NMR (200 MHz, CDCl_3_): δ 7.67–7.00 (m, 8H), 2.49 (s, 3H).

#### (4-Bromophenyl)(*o*-tolyl)methanone (**1f**)

Synthesized following a reported procedure^12b^ as a pale yellow oil (1.03 g, 63% yield) after flash chromatography on silica gel using petroleum ether/EtOAc 97:3 as eluent. ^1^H NMR (400 MHz, CDCl_3_): δ 7.72–7.65 (m, 2H), 7.64–7.59 (m, 2H), 7.47–7.38 (m, 1H), 7.36–7.24 (m, 4H), 2.35 (s, 1H).

The MBH carbonates **2a–e** were synthesized as described in the literature.^6,21^ The MBH carbonates **2f–k** were synthesized as follows: to a mixture of the corresponding aromatic aldehyde (5 mmol, 1 equiv) and the naphthyl acrylate (1.2 equiv) was added DABCO (1 equiv) and stirred for 45 min at room temperature. For liquid aldehydes, the reaction was performed in neat conditions, whereas 1 mL of MeCN was used as solvent for solid aldehydes, after which the mixture was diluted with CH_2_Cl_2_ (2 mL) and directly purified by silica gel column chromatography to furnish the corresponding MBH alcohol. To a solution of MBH alcohol (3 mmol, 1 equiv) in CH_2_Cl_2_ was added a mixture of (Boc)_2_O (1.1 equiv) and DMAP (0.1 equiv) at 0 °C and stirred for 30 min, after which the reaction mixture was diluted with CH_2_Cl_2_ and a solution of 4 M HCl was added. The organic layer was separated and washed with saturated NaHCO_3_. Subsequently, the organic layer was treated with brine, dried over anhydrous Na_2_SO_4_, and concentrated in vacuo to give the crude product. Purification by column chromatography using a mixture of EtOAc/petroleum ether as eluent furnished the pure MBH carbonate **2f–k**.

#### Naphthalen-1-yl 2-(((*tert*-butoxycarbonyl)oxy)(phenyl)methyl) Acrylate (**2f**)

Synthesized following the general procedure as a white solid (934 mg, 77% yield) after flash chromatography on silica gel using petroleum ether/EtOAc 95:5 as eluent. ^1^H NMR (200 MHz, CDCl_3_): δ 7.84 (d, *J* = 8.0 Hz, 1H), 7.73 (d, *J* = 8.2 Hz, 1H), 7.62–7.30 (m, 9H), 7.16 (d, *J* = 7.5 Hz, 1H), 6.74 (d, *J* = 14.2 Hz, 2H), 6.19 (s, 1H), 1.49 (s, 9H) ppm. ^13^C{^1^H} NMR (101 MHz, CDCl_3_): δ 163.6, 152.4, 146.3, 139.2, 137.3, 134.6, 128.7, 128.0, 127.9, 127.6, 126.6, 126.4, 126.4, 126.2, 125.3, 121.2, 118.0, 82.8, 76.0, 27.8 ppm. HRMS calculated for [C_25_H_24_O_5_ + Na]^+^: 427.1521, found: 427.1499.

#### Naphthalen-2-yl 2-(((*tert*-butoxycarbonyl)oxy)(phenyl)methyl) Acrylate (**2g**)

Synthesized following the general procedure as a white solid (946 mg, 78% yield) after flash chromatography on silica gel using petroleum ether/EtOAc 95:5 as eluent. ^1^H NMR (400 MHz, CDCl_3_): δ 7.90–7.70 (m, 3H), 7.53–7.30 (m, 8H), 7.13 (dd, *J* = 8.9, 2.3 Hz, 1H), 6.66 (d, *J* = 18.5 Hz, 2H), 6.12 (d, *J* = 1.5 Hz, 1H), 1.48 (s, 9H) ppm. ^13^C{^1^H} NMR (101 MHz, CDCl_3_): δ 163.6, 152.4, 148.0, 139.4, 137.4, 133.6, 131.50, 129.4, 128.6, 128.6, 127.8, 127.7, 127.6, 127.5, 126.5, 125.7, 120.9, 118.5, 82.8, 75.9, 27.8 ppm. HRMS calculated for [C_25_H_24_O_5_ + Na]^+^: 427.1521, found: 427.1499.

#### Naphthalen-2-yl 2-(((*tert*-butoxycarbonyl)oxy)(naphthalen-2-yl)methyl) Acrylate (**2h**)

Synthesized following the general procedure as a white solid (1.08 g, 79% yield) after flash chromatography on silica gel using petroleum ether/EtOAc 95:5 as eluent. ^1^H NMR (400 MHz, CDCl_3_): δ 8.01 (d, *J* = 1.6 Hz, 1H), 7.92–7.80 (m, 5H), 7.78–7.74 (m, 1H), 7.65–7.59 (m, 1H), 7.55–7.45 (m, 5H), 7.16 (dd, *J* = 8.9, 2.3 Hz, 1H), 6.83 (s, 1H), 6.75 (s, 1H), 6.22 (d, *J* = 1.5 Hz, 1H), 1.51 (s, 9H) ppm. ^13^C{^1^H} NMR (101 MHz, CDCl_3_): δ 192.3, 163.7, 152.4, 148.0, 139.4, 134.7, 134.6, 133.6, 133.3, 133.1, 131.4, 129.4, 128.4, 128.3, 127.7, 127.7, 127.6, 127.2, 126.5, 126.4, 126.3, 125.7, 125.17, 122.8, 120.9, 118.5, 27.8 ppm. HRMS calculated for [C_25_H_24_O_5_ + H]^+^: 455.1859, found: 455.1825.

#### Naphthalen-1-yl 2-(((*tert*-butoxycarbonyl)oxy)(4-(trifluoromethyl)phenyl)methyl)acrylate (**2i**)

Synthesized following the general procedure as a pale yellow solid (865 mg, 61% yield) after flash chromatography on silica gel using petroleum ether/EtOAc 95:5 as eluent. ^1^H NMR (200 MHz, CDCl_3_): δ 8.06–7.10 (m, 11H), 6.81 (s, 1H), 6.69 (s, 1H), 6.23 (s, 1H), 1.48 (s, 9H) ppm. ^13^C{^1^H} NMR (126 MHz, CDCl_3_): δ 163.4, 152.2, 146.2, 141.5, 138.7, 134.6, 130.8 (q, *J* = 32.5 Hz), 128.2, 128.2, 128.0, 126.5, 126.5, 126.5, 126.3, 125.7, 125.6, 125.3, 120.9, 118.0, 83.3, 75.1, 27.7 ppm. ^19^F NMR (376 MHz, CDCl_3_): δ −63.04 ppm. HRMS calculated for [C_26_H_23_F_3_O_5_ + Na]^+^: 495.1395, found: 495.1363.

#### Naphthalen-1-yl 2-(((*tert*-butoxycarbonyl)oxy)(3-fluorophenyl)methyl) Acrylate (**2j**)

Synthesized following the general procedure as a pale yellow solid (608 mg, 48% yield) after flash chromatography on silica gel using petroleum ether/EtOAc 95:5 as eluent. ^1^H NMR (200 MHz, CDCl_3_): δ 7.89–7.68 (m, 2H), 7.56–7.29 (m, 7H), 7.25–6.97 (m, 3H), 6.80 (s, 1H), 6.66 (s, 1H), 6.20 (d, *J* = 1.3 Hz, 1H), 1.49 (s, 9H) ppm. ^13^C{^1^H} NMR (101 MHz, CDCl_3_): δ 163.4, 162.9 (d, *J*_C–F_ = 247 Hz), 152.2, 146.3, 140.0, 138.9, 134.6, 130.3, 130.2, 128.1, 127.9, 126.6, 126.5, 126.4, 126.2, 125.3, 123.6, 123.5, 121.0, 118.02, 115.7 (d, *J*_C–F_ = 21.1 Hz), 114.8 (d, *J*_C–F_ = 22.4 Hz), 83.1, 75.1, 27.7 ppm. ^19^F NMR (376 MHz, CDCl_3_): δ −112.72 ppm. HRMS calculated for [C_25_H_23_BrO_5_ + Na]^+^: 445.1427, found: 445.1406.

#### Naphthalen-1-yl 2-(((*tert*-butoxycarbonyl)oxy)(2-fluorophenyl)methyl) Acrylate (**2k**)

Synthesized following the general procedure as a white solid (659 mg, 52% yield) after flash chromatography on silica gel using petroleum ether/EtOAc 9:1 as eluent. ^1^H NMR (200 MHz, CDCl_3_): δ 7.91–7.66 (m, 3H), 7.59–7.29 (m, 8H), 7.25–7.02 (m, 4H), 6.99 (s, 1H), 6.82 (s, 1H), 6.13 (s, 1H), 1.48 (s, 10H) ppm. ^13^C{^1^H} NMR (101 MHz, CDCl_3_): δ 163.4, 160.5 (d, *J*_C–F_ = 250 Hz), 152.2, 146.3, 138.0, 134.6, 130.5, 129.0, 128.9, 128.8, 127.9, 126.6, 126.4, 126.4, 126.2, 125.3, 124.8 (d, *J*_C–F_ = 13.4 Hz), 124.3, 124.3, 121.1, 118.0, 115.9 (d, *J*_C–F_ = 21.4 Hz), 83.1, 70.0, 27.7 ppm. ^19^F NMR (376 MHz, CDCl_3_): δ −116.82 ppm. HRMS calculated for [C_25_H_23_BrO_5_ + Na]^+^: 445.1427, found: 445.1406.

Catalysts **4b**–**d** were prepared as reported in the literature.^22^ The isatin-derived MBH carbonates **5a**–**f** were synthesized as described in the literature.^23^

#### Methyl 2-(3-((*tert*-butoxycarbonyl)oxy)-1-methyl-2-oxoindolin-3-yl) Acrylate (**5a**)

Synthesized following the general procedure as a white solid (498 mg, 71% yield) after flash chromatography on silica gel using petroleum ether/EtOAc 85:15 as eluent. ^1^H NMR (200 MHz, CDCl_3_): δ 7.33 (td, *J* = 7.7, 1.4 Hz, 1H), 7.18 (d, *J* = 7.5 Hz, 1H), 6.99 (td, *J* = 7.5, 1.0 Hz, 1H), 6.85 (d, *J* = 7.8 Hz, 1H), 6.60–6.54 (m, 1H), 6.53 (s, 1H), 3.57 (s, 3H), 3.29 (s, 3H), 1.34 (s, 9H) ppm. Spectroscopic data are in accordance with the literature.^23a^

#### Methyl 2-(3-((*tert*-butoxycarbonyl)oxy)-6-chloro-1-methyl-2-oxoindolin-3-yl)acrylate (**5b**)

Synthesized following the general procedure as a white solid (375 mg, 48% yield) after flash chromatography on silica gel using petroleum ether/EtOAc 8:2 as eluent. ^1^H NMR (200 MHz, CDCl_3_): δ 7.09 (d, *J* = 7.7 Hz, 1H), 6.97 (dd, *J* = 7.9, 1.8 Hz, 1H), 6.85 (d, *J* = 1.6 Hz, 1H), 6.57 (s, 1H), 6.54 (s, 1H), 3.59 (s, 3H), 3.28 (s, 3H), 1.36 (s, 9H) ppm. Spectroscopic data are in accordance with the literature.^23a^

#### Methyl 2-(3-((*tert*-butoxycarbonyl)oxy)-5-fluoro-1-methyl-2-oxoindolin-3-yl)acrylate (**5c**)

Synthesized following the general procedure as a white solid (392 mg, 60% yield) after flash chromatography on silica gel using petroleum ether/EtOAc 8:2 as eluent. ^1^H NMR (200 MHz, CDCl_3_): δ 7.11–6.91 (m, 2H), 6.83–6.72 (m, 1H), 6.59 (s, 1H), 6.54 (s, 1H), 3.60 (s, 3H), 3.29 (s, 3H), 1.37 (s, 9H) ppm. Spectroscopic data are in accordance with the literature.^23b^

#### Methyl 2-(3-((*tert*-butoxycarbonyl)oxy)-1,5-dimethyl-2-oxoindolin-3-yl)acrylate (**5d**)

Synthesized following the general procedure as a pale yellow solid (495 mg, 65% yield) after flash chromatography on silica gel using petroleum ether/EtOAc 8:2 as eluent. ^1^H NMR (200 MHz, CDCl_3_): δ 7.13 (d, *J* = 7.3 Hz, 1H), 7.00 (s, 1H), 6.74 (d, *J* = 8.1 Hz, 1H), 6.56 (s, 1H), 6.53 (s, 1H), 3.58 (s, 3H), 3.28 (s, 3H), 2.29 (s, 3H), 1.36 (s, 9H) ppm. Spectroscopic data are in accordance with the literature.^23b^

#### Ethyl 2-(3-((*tert*-butoxycarbonyl)oxy)-1-methyl-2-oxoindolin-3-yl)acrylate (**5e**)

Synthesized following the general procedure as a white solid (562 mg, 82% yield) after flash chromatography on silica gel using petroleum ether/EtOAc 85:15 as eluent. ^1^H NMR (200 MHz, CDCl_3_): δ 7.33 (td, *J* = 7.8, 1.3 Hz, 1H), 7.18 (dd, *J* = 7.4, 0.8 Hz, 1H), 6.99 (td, *J* = 7.6, 0.9 Hz, 1H), 6.84 (d, *J* = 7.8 Hz, 1H), 6.57 (s, 1H), 6.51 (s, 1H), 3.98 (qd, *J* = 7.2, 4.5 Hz, 2H), 3.29 (s, 3H), 1.34 (s, 9H), 1.09 (t, *J* = 7.1 Hz, 3H) ppm. Spectroscopic data are in accordance with the literature.^23b^

#### Methyl 2-(1-allyl-3-((*tert*-butoxycarbonyl)oxy)-2-oxoindolin-3-yl)acrylate (**5f**)

Synthesized following the general procedure as a pale yellow solid (599 mg, 64% yield) after flash chromatography on silica gel using petroleum ether/EtOAc 9:1 as eluent. ^1^H NMR (200 MHz, CDCl_3_): δ 7.38–7.26 (m, 1H), 7.19 (d, *J* = 8.5 Hz, 1H), 6.98 (t, *J* = 7.5 Hz, 1H), 6.85 (d, *J* = 8.3 Hz, 1H), 6.54 (d, *J* = 7.6 Hz, 2H), 6.06–5.81 (m, 1H), 5.47 (d, *J* = 17.2 Hz, 1H), 5.26 (d, *J* = 10.4 Hz, 1H), 4.56–4.28 (m, 2H), 3.57 (s, 3H), 1.35 (s, 9H) ppm. Spectroscopic data are in accordance with the literature.^23b^

### General Procedure 1: Light-Triggered Catalytic Asymmetric Benzylation of MBH Carbonates **2f–k** for the Synthesis of Compounds **3f–o**

To an oven-dried 4 mL screw cap vial equipped with a septum is added 4 Å activated molecular sieve (20 mg) and the MBH carbonates **2f–k** (0.1 mmol, 1 equiv). The vial is purged with argon and β-isocupreidine **4a** (10 mol %) is subsequently added. Previously argon-degassed anhydrous toluene (1 mL) is introduced into the vial followed by the corresponding 2-methylbenzophenone derivative **1a–e** (5 equiv). After a final purge with argon, the vial is sealed with parafilm and irradiated with the 9 W 365 nm bulb photochemical setup.^13^ After 3 h, the reaction mixture is directly loaded into a silica gel column chromatography and purified using mixtures of petroleum ether/EtOAc as eluent to furnish enantioenriched benzylated products **3f–o**.

#### Naphthalen-1-yl (*S*)-4-(2-benzoylphenyl)-2-methylene-3-phenylbutanoate (**3f**)

Synthesized following the general procedure 1 as an amorphous white solid (26.5 mg, 55% yield, 82:18 *er*) after flash chromatography on silica gel using petroleum ether/EtOAc 98:2 as eluent. ^1^H NMR (400 MHz, CDCl_3_): δ 7.83 (d, *J* = 8.3 Hz, 1H), 7.74–7.65 (m, 3H), 7.61–7.53 (m, 1H), 7.42 (m, 6H), 7.35–7.24 (m, 3H), 7.24–7.13 (m, 6H), 7.06 (d, *J* = 7.5 Hz, 1H), 6.67 (s, 1H), 5.99 (s, 1H), 4.44 (dd, *J* = 9.2, 6.4 Hz, 1H), 3.61–3.37 (m, 2H) ppm. ^13^C{^1^H} NMR (101 MHz, CDCl_3_): δ 198.4, 165.5, 146.7, 142.7, 141.3, 139.1, 138.7, 137.7, 134.6, 133.1, 131.0, 130.3, 130.2, 129.2, 128.4, 128.4, 128.3, 127.8, 126.8, 126.7, 126.6, 126.3, 125.9, 125.4, 125.3, 121.3, 118.0, 48.2, 37.2 ppm. HRMS calculated for [C_34_H_26_O_3_ + H]^+^: 483.1960, found: 483.1946. [α]_D_^30^ + 5.94 (*c* = 0.5, CHCl_3_, 82:18 *er*). HPLC analysis: Phenomenex Lux 5u Cellulose-4, hexane/*i*-PrOH = 95:5, flow rate = 1.0 mL/min, λ = 254 nm, retention time; *t*_R_(major) = 20.1 min, *t*_R_(minor) = 22.0 min.

#### Naphthalen-2-yl (*S*)-4-(2-benzoylphenyl)-2-methylene-3-phenylbutanoate (**3g**)

Synthesized following the general procedure 1 as an amorphous white solid (29.9 mg, 62% yield, 80:20 *er*) after flash chromatography on silica gel using petroleum ether/EtOAc 98:2 as eluent. ^1^H NMR (400 MHz, CDCl_3_): δ 7.88–7.72 (m, 3H), 7.69–7.63 (m, 2H), 7.61–7.54 (m, 1H), 7.50–7.34 (m, 7H), 7.28–7.20 (m, 2H), 7.19–7.08 (m, 5H), 7.01 (dd, *J* = 8.9, 2.3 Hz, 1H), 6.56 (s, 1H), 5.91 (d, *J* = 1.3 Hz, 1H), 4.37 (dd, *J* = 8.8, 6.7 Hz, 1H), 3.69–3.26 (m, 2H) ppm. ^13^C{^1^H} NMR (101 MHz, CDCl_3_): δ 198.3, 165.4, 148.3, 142.7, 141.2, 139.1, 138.7, 137.7, 133.6, 133.0, 131.3, 130.9, 130.3, 130.1, 129.2, 129.1, 128.3, 128.3, 128.2, 127.7, 127.6, 126.6, 126.57, 126.4, 125.6, 125.3, 121.0, 118.4, 48.1, 37.1 ppm. HRMS calculated for [C_34_H_26_O_3_ + H]^+^: 483.1960, found: 483.1946. [α]_D_^30^ + 16.10 (*c* = 1.0, CHCl_3_, 80:20 *er*). HPLC analysis: Phenomenex Lux 5u Cellulose-4, hexane/*i*-PrOH = 95:5, flow rate = 1.0 mL/min, λ = 254 nm, retention time; *t*_R_(major) = 16.2 min, *t*_R_(minor) = 22.2 min.

#### Naphthalen-2-yl (*S*)-4-(2-benzoylphenyl)-2-methylene-3-(naphthalen-2-yl) Butanoate (**3h**)

Synthesized following the general procedure 1 as an amorphous white solid (22.4 mg, 42% yield, 85:15 *er*) after flash chromatography on silica gel using petroleum ether/EtOAc 98:2 as eluent. ^1^H NMR (400 MHz, CDCl_3_): δ 7.83–7.79 (m, 1H), 7.77 (d, *J* = 8.9 Hz, 1H), 7.73–7.69 (m, 2H), 7.61 (dd, *J* = 11.6, 8.1 Hz, 2H), 7.55 (d, *J* = 1.7 Hz, 1H), 7.52–7.48 (m, 2H), 7.46 (dd, *J* = 6.9, 3.3 Hz, 2H), 7.44–7.34 (m, 6H), 7.27–7.18 (m, 5H), 7.02 (dd, *J* = 8.9, 2.4 Hz, 1H), 6.63 (s, 1H), 6.01 (s, 1H), 4.55 (dd, *J* = 9.6, 5.9 Hz, 1H), 3.72–3.50 (m, 2H) ppm. ^13^C{^1^H} NMR (101 MHz, CDCl_3_): δ 198.3, 165.4, 148.3, 142.9, 139.2, 138.7, 138.5, 137.3, 133.6, 133.4, 132.9, 132.4, 131.3, 131.0, 130.3, 130.1, 129.2, 129.1, 128.0, 127.9, 127.7, 127.7, 127.6, 127.5, 127.0, 126.7, 126.6, 126.4, 125.8, 125.6, 125.4, 125.3, 121.0, 118.5, 48.3, 36.9 ppm. HRMS calculated for [C_38_H_28_O_3_ + H]^+^: 533.2117, found: 533.2122. [α]_D_^30^ + 6.16 (*c* = 1.0, CHCl_3_, 85:15 *er*). HPLC analysis: Phenomenex Lux 5u Cellulose-5, hexane/*i*-PrOH = 95:5, flow rate = 1.0 mL/min, λ = 254 nm, retention time; *t*_R_(minor) = 25.4 min, *t*_R_(major) = 30.6 min.

#### Naphthalen-1-yl (*S*)-4-(2-benzoylphenyl)-2-methylene-3-(4-(trifluoromethyl) phenyl) Butanoate (**3i**)

Synthesized following the general procedure 1 as an amorphous white solid (33.6 mg, 61% yield, 82:18 *er*) after flash chromatography on silica gel using petroleum ether/EtOAc 98:2 as eluent. ^1^H NMR (400 MHz, CDCl_3_): δ 7.84 (d, *J* = 8.2 Hz, 1H), 7.72 (d, *J* = 8.2 Hz, 1H), 7.58 (d, *J* = 5.6 Hz, 3H), 7.49–7.39 (m, 7H), 7.32–7.26 (m, 6H), 7.15 (d, *J* = 8.4 Hz, 1H), 7.08 (d, *J* = 7.5 Hz, 1H), 6.75 (s, 1H), 6.08 (s, 1H), 4.50 (t, *J* = 7.8 Hz, 1H), 3.54 (d, *J* = 7.8 Hz, 2H) ppm. ^13^C{^1^H} NMR (101 MHz, CDCl_3_): δ 198.2, 165.0, 146.5, 145.4, 142.0, 138.6, 138.5, 137.5, 134.5, 133.2, 131.0, 130.4, 130.1, 129.4, 128.8, 128.5, 128.3, 127.9, 127.1, 126.6, 126.4, 126.3, 126.11, 125.6, 125.3, 125.3, 125.2, 120.8, 117.9, 48.3, 36.7 ppm. ^19^F NMR (376 MHz, CDCl_3_): δ −62.68. HRMS calculated for [C_35_H_25_F_3_O_3_ + H]^+^: 551.1834, found: 551.1848. [α]_D_^30^ + 20.35 (*c* = 0.7, CHCl_3_, 82:18 *er*). HPLC analysis: Phenomenex Lux 5u Cellulose-5, hexane/*i*-PrOH = 95:5, flow rate = 1.0 mL/min, λ = 254 nm, retention time; *t*_R_(minor) = 10.4 min, *t*_R_(major) = 11.7 min.

#### Naphthalen-1-yl (*S*)-4-(2-benzoylphenyl)-3-(3-fluorophenyl)-2-methylenebutanoate (**3j**)

Synthesized following the general procedure 1 as an amorphous white solid (38.0 mg, 76% yield, 80:20 *er*) after flash chromatography on silica gel using petroleum ether/EtOAc 98:2 as eluent. ^1^H NMR (400 MHz, CDCl_3_): δ 7.85 (d, *J* = 8.2 Hz, 1H), 7.75–7.67 (m, 3H), 7.61–7.55 (m, 1H), 7.44 (m, 5H), 7.36–7.26 (m, 5H), 7.10 (m, 2H), 6.93 (m, 1H), 6.90–6.80 (m, 2H), 6.72 (s, 1H), 6.02 (d, *J* = 1.3 Hz, 1H), 4.44 (dd, *J* = 9.2, 6.4 Hz, 1H), 3.60–3.39 (m, 2H) ppm. ^13^C{^1^H} NMR (101 MHz, CDCl_3_): δ 198.2, 165.1, 162.8 (d, *J*_C–F_ = 246 Hz), 146.6, 144.0, 143.9, 142.1, 138.7, 138.6, 137.5, 134.5, 133.1, 131.0, 130.3, 129.8, 129.7, 129.3, 128.3, 127.9, 127.0, 126.7, 126.37, 126.0, 125.6, 125.3, 124.1, 124.1, 121.1, 118.0, 115.3 (d, *J*_C–F_ = 21.5 Hz), 113.7 (d, *J*_C–F_ = 21.0 Hz), 48.0, 37.0 ppm. ^19^F NMR (376 MHz, CDCl_3_): δ −113.63. HRMS calculated for [C_34_H_25_FO_3_ + H]^+^: 501.1866, found: 501.1849. [α]_D_^30^ + 28.19 (*c* = 1.0, CHCl_3_, 80:20 *er*). HPLC analysis: Phenomenex Lux 5u Cellulose-5, hexane/*i*-PrOH = 95/5, flow rate = 1.0 mL/min, λ = 254 nm, retention time; *t*_R_(minor) = 16.9 min, *t*_R_(major) = 19.8 min.

#### Naphthalen-1-yl (*R*)-4-(2-benzoylphenyl)-3-(2-fluorophenyl)-2-methylenebutanoate (**3k**)

Synthesized following the general procedure 1 as an amorphous white solid (28.5 mg, 57% yield, 82:18 *er*) after flash chromatography on silica gel using petroleum ether/EtOAc 98:2 as eluent. ^1^H NMR (500 MHz, CDCl_3_): δ 7.86–7.82 (m, 1H), 7.73–7.70 (m, 3H), 7.57 (t, *J* = 7.4 Hz, 1H), 7.49–7.45 (m, 1H), 7.44–7.40 (m, 3H), 7.38–7.35 (m, 1H), 7.34–7.24 (m, 6H), 7.14 (m, 1H), 7.06 (dd, *J* = 7.5, 1.1 Hz, 1H), 6.98 (td, *J* = 7.5, 1.3 Hz, 1H), 6.89 (m, 1H), 6.73 (s, 1H), 6.02 (m, 1H), 4.79 (dd, *J* = 9.4, 6.2 Hz, 1H), 3.62 (dd, *J* = 13.8, 6.2 Hz, 1H), 3.44 (dd, *J* = 13.8, 9.4 Hz, 1H) ppm. ^13^C{^1^H} NMR (101 MHz, CDCl_3_): δ 198.3, 165.1, 164.0, 1, 160.9 (d, *J*_C–F_ = 247 Hz), 146.6, 141.5, 138.7, 138.5, 137.7, 134.5, 133.0, 130.8, 130.3, 130.3, 129.4 (d, *J*_C–F_ = 4.1 Hz), 129.3, 128.5 (d, *J*_C–F_ = 14.2 Hz), 128.3, 128.3, 127.8, 127.62, 127.6, 126.7, 126.3, 126.3, 125.9, 125.5, 125.3, 124.1 (d, *J*_C–F_ = 3.6 Hz), 121.1, 118.0, 115.5 (d, *J*_C–F_ = 22.5 Hz), 40.0 (d, *J*_C–F_ = 2.0 Hz), 36.0. ^19^F NMR (376 MHz, CDCl_3_): δ −117.27. HRMS calculated for [C_34_H_25_FO_3_ + H]^+^: 501.1866, found: 501.1847. [α]_D_^30^ + 50.05 (*c* = 0.7, CHCl_3_, 82:18 *er*). HPLC analysis: Phenomenex Lux 5u Cellulose-4, hexane/*i*-PrOH = 95:5, flow rate = 0.6 mL/min, λ = 254 nm, retention time; *t*_R_(minor) = 35.6 min, *t*_R_(major) = 37.2 min.

#### Naphthalen-2-yl (*S*)-4-(2-benzoyl-4-bromophenyl)-2-methylene-3-phenylbutanoate (**3l**)

Synthesized following the general procedure 1 as an amorphous white solid (37.6 mg, 67% yield, 74:26 *er*) after flash chromatography on silica gel using petroleum ether/EtOAc 98:2 as eluent. ^1^H NMR (400 MHz, CDCl_3_): δ 7.84–7.74 (m, 3H), 7.69–7.64 (m, 2H), 7.64–7.55 (m, 1H), 7.51–7.43 (m, 5H), 7.38 (dd, *J* = 4.9, 2.2 Hz, 2H), 7.19–7.09 (m, 6H), 7.02 (dd, *J* = 8.9, 2.3 Hz, 1H), 6.56 (s, 1H), 5.87 (d, *J* = 1.3 Hz, 1H), 4.32 (dd, *J* = 9.3, 6.3 Hz, 1H), 3.66–3.25 (m, 2H) ppm. ^13^C{^1^H} NMR (101 MHz, CDCl_3_): δ 196.6, 165.3, 148.3, 142.6, 140.8, 138.0, 136.9, 133.6, 133.5, 133.0, 132.6, 131.4, 131.4, 130.3, 129.2, 128.5, 128.4, 128.3, 127.7, 127.6, 126.8, 126.6, 126.49, 125.6, 121.0, 119.3, 118.4, 47.9, 36.7 ppm. HRMS calculated for [C_34_H_25_BrO_3_ + H]^+^: 563.1051, found: 563.1041. [α]_D_^30^ + 7.32 (*c* = 0.4, CHCl_3_, 74:26 *er*). HPLC analysis: Phenomenex Lux 5u Cellulose-4, hexane/*i*-PrOH = 95:5, flow rate = 0.9 mL/min, λ = 254 nm, retention time; *t*_R_(minor) = 13.7 min, *t*_R_(major) = 15.5 min.

#### Naphthalen-2-yl (*S*)-4-(2-benzoyl-4-methylphenyl)-2-methylene-3-phenylbutanoate (**3m**)

Synthesized following the general procedure 1 as an amorphous white solid (27.3 mg, 55% yield, 78:22 *er*) after flash chromatography on silica gel using petroleum ether/EtOAc 98:2 as eluent. ^1^H NMR (400 MHz, CDCl_3_): δ 7.87–7.72 (m, 3H), 7.69–7.61 (m, 2H), 7.56 (t, *J* = 7.4 Hz, 1H), 7.53–7.36 (m, 5H), 7.21–7.08 (m, 7H), 7.08–6.97 (m, 2H), 6.58 (s, 1H), 5.93 (s, 1H), 4.37 (dd, *J* = 8.8, 6.7 Hz, 1H), 3.74–3.26 (m, 2H), 2.36 (s, 3H) ppm. ^13^C{^1^H} NMR (101 MHz, CDCl_3_): δ 198.4, 165.4, 148.4, 142.8, 141.4, 140.5, 139.5, 138.1, 135.8, 133.6, 132.7, 131.8, 131.3, 130.3, 129.7, 129.2, 128.3, 128.2, 128.1, 127.7, 127.6, 126.6, 126.57, 126.4, 126.0, 125.6, 121.1, 118.4, 48.2, 37.0, 21.4 ppm. HRMS calculated for [C_35_H_28_O_3_ + H]^+^: 497.2117, found: 497.2121. [α]_D_^30^ + 26.90 (*c* = 0.9, CHCl_3_, 78:22 *er*). HPLC analysis: Phenomenex Lux 5u Cellulose-4, hexane/*i*-PrOH = 95:5, flow rate = 1.0 mL/min, λ = 254 nm, retention time; *t*_R_(major) = 16.9 min, *t*_R_(minor) = 22.4 min.

#### Naphthalen-2-yl (*S*)-4-(2-(4-methoxybenzoyl)phenyl)-2-methylene-3-phenylbutanoate (**3n**)

Synthesized following the general procedure 1 as an amorphous white solid (26.7 mg, 52% yield, 78:22 *er*) after flash chromatography on silica gel using petroleum ether/EtOAc 98:2 as eluent. ^1^H NMR (400 MHz, CDCl_3_): δ 7.85–7.73 (m, 3H), 7.69 (d, *J* = 8.8 Hz, 2H), 7.47 (ddd, *J* = 7.0, 4.6, 1.7 Hz, 2H), 7.35 (dd, *J* = 8.6, 2.5 Hz, 2H), 7.30–7.22 (m, 3H), 7.15 (q, *J* = 7.1, 6.5 Hz, 5H), 7.01 (dd, *J* = 8.9, 2.2 Hz, 1H), 6.89 (d, *J* = 8.9 Hz, 2H), 6.56 (s, 1H), 5.90 (s, 1H), 4.37 (dd, *J* = 9.0, 6.7 Hz, 1H), 3.59–3.26 (m, 2H) ppm. ^13^C{^1^H} NMR (101 MHz, CDCl_3_): δ 197.1, 165.4, 163.6, 148.3, 142.7, 141.4, 139.2, 138.5, 133.6, 132.7, 131.3, 130.8, 130.5, 129.7, 129.2, 128.5, 128.3, 128.2, 127.7, 127.6, 126.6, 126.6, 126.4, 125.6, 125.3, 121.0, 118.4, 113.5, 55.4, 47.9, 37.2 ppm. HRMS calculated for [C_35_H_28_O_4_ + H]^+^: 513.2066, found: 513.2064. [α]_D_^30^ + 36.67 (*c* = 0.5, CHCl_3_, 78:22 *er*). HPLC analysis: Phenomenex Lux 5u Cellulose-5, hexane/*i*-PrOH = 95:5, flow rate = 1.0 mL/min, λ = 254 nm, retention time; *t*_R_(minor) = 50.3 min, *t*_R_(major) = 59.8 min.

#### Naphthalen-2-yl (*S*)-4-(2-(2-fluorobenzoyl)phenyl)-2-methylene-3-phenylbutanoate (**3o**)

Synthesized following the general procedure 1 as an amorphous white solid (29.0 mg, 58% yield, 72:28 *er*) after flash chromatography on silica gel using petroleum ether/EtOAc 98:2 as eluent. ^1^H NMR (400 MHz, CDCl_3_): δ 7.84–7.73 (m, 3H), 7.55–7.44 (m, 4H), 7.39–7.32 (m, 3H), 7.25–7.18 (m, 7H), 7.14 (ddd, *J* = 8.2, 5.6, 1.0 Hz, 2H), 7.03 (dd, *J* = 8.9, 2.3 Hz, 1H), 6.62 (s, 1H), 5.99 (d, *J* = 1.2 Hz, 1H), 4.43 (dd, *J* = 9.1, 6.3 Hz, 1H), 3.77–3.43 (m, 2H) ppm. ^13^C{^1^H} NMR (101 MHz, CDCl_3_): δ 195.3, 165.4, 161.0 (d, *J*_C–F_ = 256 Hz), 148.4, 142.8, 141.5, 139.8, 138.6, 134.0, 133.9, 133.6, 131.7, 131.7, 131.5, 131.3, 131.1, 130.2, 129.2, 128.4, 128.3, 127.5 (d, *J*_C–F_ = 12.0 Hz), 126.6, 126.4, 126.4, 125.8, 125.5, 124.1, 124.1, 121.1, 118.5, 116.5 (d, *J*_C–F_ = 22.0 Hz), 48.1, 37.4 ppm. ^19^F NMR (376 MHz, CDCl_3_): δ −110.69 ppm. HRMS calculated for [C_34_H_25_FO_3_ + H]^+^: 501.1866, found: 501.1847. [α]_D_^30^ + 1.08 (*c* = 0.9, CHCl_3_, 72:28 *er*). HPLC analysis: Phenomenex Lux 5u Cellulose-4, hexane/*i*-PrOH = 95:5, flow rate = 1.0 mL/min, λ = 254 nm, retention time; *t*_R_(major) = 16.4 min, *t*_R_(minor) = 22.0 min.

#### Methylen-2-yl-4-(2-benzoylphenyl)-2-methylene-3-phenylbutanoate (**3a**)

To a solution of **3g** (1 mmol) in MeOH is added 1.2 equiv of Cs_2_CO_3_, and the mixture is stirred overnight at room temperature. Subsequently, water is added to the reaction crude and the resulting solution is extracted three times with EtOAc. The organic layers are dried with anhydrous MgSO_4_, evaporated, and the resulting residue is purified by silica gel column chromatography using petroleum ether/EtOAc 95:5 as eluent. 356 mg of compound **3a** is obtained as an amorphous white solid in 96% yield and 80:20 *er*. ^1^H NMR (400 MHz, CDCl_3_): δ 7.67–7.63 (m, 2H), 7.58 (ddt, *J* = 8.8, 7.4, 1.4 Hz, 1H), 7.45–7.40 (m, 2H), 7.35 (ddd, *J* = 8.4, 6.7, 2.0 Hz, 1H), 7.26–7.18 (m, 3H), 7.15–7.02 (m, 6H), 6.28 (s, 1H), 5.69 (s, 1H), 4.27–4.21 (m, 1H), 3.61 (s, 3H), 3.42–3.27 (m, 2H). ^13^C{^1^H} NMR (101 MHz, CDCl_3_): δ 198.37, 167.12, 142.92, 141.43, 139.21, 138.69, 137.68, 133.05, 130.85, 130.01, 129.43, 128.97, 128.26, 128.22, 128.19, 126.51, 125.28, 124.90, 51.81, 47.80, 37.15. HRMS calculated for [C_25_H_22_O_3_ + Na]^+^: 393.148, found: 393.1467. [α]_D_^30^ + 31.35 (*c* = 0.7, CHCl_3_, 80:20 *er*). HPLC analysis: Phenomenex Lux 5u Cellulose-4, hexane/*i*-PrOH = 98:2, flow rate = 0.8 mL/min, λ = 254 nm, retention time; *t*_R_(minor) = 25.0 min, *t*_R_(major) = 28.1.

### General Procedure 2: Light-Triggered Catalytic Asymmetric Allylic Benzylation of MBH Carbonates **5a–f** for the Synthesis of Compounds **6a–j**

To an oven-dried 4 mL screw cap vial are added MBH carbonate **5a–f** (0.1 mmol, 1 equiv) and β-isocupreidine **4a** (0.1 equiv) under argon. Previously, argon-degassed anhydrous MeOH (1 mL) is introduced into the vial followed by 2-methylbenzophenone derivative **1a–e** (5 equiv). The vial is sealed with parafilm and irradiated under a 365 nm light source at 350 mA. After total consumption of the starting material (TLC analysis), the reaction mixture is directly loaded into a silica gel column chromatograph and purified using mixtures of petroleum ether/EtOAc as eluent to furnish the enantioenriched benzylated products **6a–j**.

#### Methyl (*R*)-2-(3-(2-benzoylbenzyl)-1-methyl-2-oxoindolin-3-yl)acrylate (**6a**)

Synthesized following the general procedure 2 as a red solid (33.2 mg, 78% yield, 70:30 *er*) after flash chromatography on silica gel using petroleum ether/EtOAc 90:10 as eluent. ^1^H NMR (400 MHz, CDCl_3_): δ 7.48–7.40 (m, 1H), 7.29–7.17 (m, 7H), 7.07–6.99 (m, 2H), 6.83 (td, *J* = 7.7, 1.1 Hz, 1H), 6.65–6.61 (m, 1H), 6.52 (s, 1H), 6.43 (d, *J* = 7.7 Hz, 1H), 6.26–6.20 (m, 1H), 6.17 (s, 1H), 4.28 (d, *J* = 12.7 Hz, 1H), 3.42 (s, 3H), 3.37 (d, *J* = 12.7 Hz, 1H), 2.91 (s, 3H) ppm. ^13^C{^1^H} NMR (101 MHz, CDCl_3_): δ 197.3, 177.8, 165.3, 144.3, 140.3, 138.1, 137.3, 135.2, 132.6, 132.5, 130.6, 130.1, 129.9, 129.7, 128.3, 127.7, 127.5, 125.7, 123.5, 122.5, 107.3, 56.1, 51.9, 35.5, 26.1 ppm. HRMS calculated for [C_27_H_23_NO_4_ + H]^+^: 426.1705, found: 426.1705. [α]_D_^30^ – 15.70 (*c* = 0.5, CHCl_3_, 70:30 *er*). HPLC analysis: Phenomenex Lux 5u Cellulose-5, hexane/*i*-PrOH = 80:20, flow rate = 1.0 mL/min, λ = 254 nm, retention time; *t*_R_(major) = 40.8 min, *t*_R_(minor) = 46.7 min.

#### Methyl (*R*)-2-(3-(2-benzoylbenzyl)-6-chloro-1-methyl-2-oxoindolin-3-yl)acrylate (**6b**)

Synthesized following the general procedure 2 as a red solid (29.9 mg, 65% yield, 70:30 *er*) after flash chromatography on silica gel using petroleum ether/EtOAc 90:10 as eluent. ^1^H NMR (400 MHz, CDCl_3_): δ 7.61–7.54 (m, 1H), 7.44–7.37 (m, 3H), 7.37–7.31 (m, 1H), 7.31–7.26 (m, 2H), 7.19–7.11 (m, 2H), 6.64 (s, 1H), 6.58 (d, *J* = 7.9 Hz, 1H), 6.51 (d, *J* = 1.8 Hz, 1H), 6.31 (s, 1H), 4.51 (d, *J* = 12.7 Hz, 1H), 3.53 (s, 3H), 3.38 (d, *J* = 12.6 Hz, 1H), 2.98 (s, 3H) ppm. ^13^C{^1^H} NMR (101 MHz, CDCl_3_): δ 145.5, 139.9, 137.8, 136.9, 133.9, 132.9, 132.7, 130.3, 130.1, 128.1, 128.0, 127.9, 125.8, 124.4, 122.3, 108.2, 55.8, 52.1, 35.1, 26.2 ppm. HRMS calculated for [C_27_H_22_ClNO_4_ + H]^+^: 460.1316, found: 460.1299. [α]_D_^30^ – 16.60 (*c* = 1.6, CHCl_3_, 70:30 *er*). HPLC analysis: Phenomenex Lux 5u Cellulose-4, hexane/*i*-PrOH = 80:20, flow rate = 1.0 mL/min, λ = 254 nm, retention time; *t*_R_(major) = 13.3 min, *t*_R_(minor) = 17.0 min.

#### Methyl (*R*)-2-(3-(2-benzoylbenzyl)-5-fluoro-1-methyl-2-oxoindolin-3-yl)acrylate (**6c**)

Synthesized following the general procedure 2 as an orange solid (34.6 mg, 78% yield, 72:28 *er*) after flash chromatography on silica gel using petroleum ether/EtOAc 90:10 as eluent. ^1^H NMR (200 MHz, CDCl_3_): δ 7.44 (dd, *J* = 38.3, 4.6 Hz, 9H), 7.12 (d, *J* = 3.3 Hz, 2H), 6.67–6.50 (m, 2H), 6.50–6.34 (m, 2H), 6.25 (s, 1H), 4.33 (d, *J* = 12.5 Hz, 1H), 3.51 (s, 3H), 3.40 (d, *J* = 12.6 Hz, 1H), 2.96 (s, 3H) ppm. ^13^C{^1^H} NMR (101 MHz, CDCl_3_): δ 197.2, 177.5, 165.1, 159.0 (d, *J*_C–F_ = 241 Hz), 139.8, 138.0, 137.0, 134.8, 132.9, 132.7, 130.4, 130.1, 130.0, 128.1, 127.9, 125.9, 114.6 (d, *J*_C–F_ = 23.4 Hz), 112.1 (d, *J*_C–F_ = 25.0 Hz), 107.8, 107.7, 56.4, 52.1, 35.4, 26.2 ppm. ^19^F NMR (376 MHz, CDCl_3_): δ −120.84. HRMS calculated for [C_27_H_22_FNO_4_ + H]^+^: 444.1611, found: 444.1604. [α]_D_^30^ – 22.20 (*c* = 1.8, CHCl_3_, 72:28 *er*). HPLC analysis: Phenomenex Lux 5u Cellulose-4, hexane/*i*-PrOH = 80:20, flow rate = 1.0 mL/min, λ = 254 nm, retention time; *t*_R_(major) = 29.2 min, *t*_R_(minor) = 33.7 min.

#### Methyl (*R*)-2-(3-(2-benzoylbenzyl)-1,5-dimethyl-2-oxoindolin-3-yl)acrylate (**6d**)

Synthesized following the general procedure 2 as a yellow solid (24.2 mg, 55% yield, 70:30 *er*) after flash chromatography on silica gel using petroleum ether/EtOAc 90:10 as eluent. ^1^H NMR (400 MHz, CDCl_3_): δ 7.48–7.42 (m, 1H), 7.36 (d, *J* = 7.6 Hz, 1H), 7.26–7.18 (m, 4H), 7.14 (d, *J* = 7.2 Hz, 2H), 7.00 (ddd, *J* = 15.8, 7.4, 1.2 Hz, 2H), 6.58 (d, *J* = 7.8 Hz, 1H), 6.53 (s, 1H), 6.40 (s, 1H), 6.32 (d, *J* = 7.8 Hz, 1H), 6.20 (s, 1H), 4.45 (d, *J* = 12.5 Hz, 1H), 3.43 (s, 3H), 3.28 (d, *J* = 12.5 Hz, 1H), 2.89 (s, 3H), 1.38 (s, 3H) ppm. ^13^C{^1^H} NMR (101 MHz, CDCl_3_): δ 197.5, 165.3, 141.9, 140.4, 137.8, 137.3, 135.6, 133.0, 132.6, 132.4, 130.5, 130.4, 130.1, 128.6, 127.7, 127.5, 124.5, 107.1, 56.2, 52.0, 26.1, 20.2 ppm. HRMS calculated for [C_28_H_25_NO_4_ + H]^+^: 440.1862, found: 440.1831. [α]_D_^30^ – 9.40 (*c* = 1.7, CHCl_3_, 70:30 *er*). HPLC analysis: Phenomenex Lux 5u Cellulose-4, hexane/*i*-PrOH = 80:20, flow rate = 1.0 mL/min, λ = 254 nm, retention time; *t*_R_(major) = 12.6 min, *t*_R_(minor) = 18.3 min.

#### Methyl (*R*)-2-(3-(2-benzoyl-5-methylbenzyl)-1-methyl-2-oxoindolin-3-yl)acrylate (**6e**)

Synthesized following the general procedure 2 as a yellow solid (26.4 mg, 60% yield, 70:30 *er*) after flash chromatography on silica gel using petroleum ether/EtOAc 90:10 as eluent. ^1^H NMR (400 MHz, CDCl_3_): δ 7.52 (tt, *J* = 7.8, 1.6 Hz, 1H), 7.37–7.27 (m, 5H), 7.13 (s, 1H), 7.03–6.89 (m, 3H), 6.73 (d, *J* = 7.3 Hz, 1H), 6.61 (s, 1H), 6.53 (d, *J* = 7.7 Hz, 1H), 6.36 (t, *J* = 7.5 Hz, 1H), 6.27 (s, 1H), 4.38 (d, *J* = 12.6 Hz, 1H), 3.51 (s, 3H), 3.42 (d, *J* = 12.6 Hz, 1H), 3.01 (s, 3H), 2.30 (s, 3H) ppm. ^13^C{^1^H} NMR (101 MHz, CDCl_3_): δ 197.5, 165.3, 141.9, 140.4, 137.8, 137.3, 135.6, 133.0, 132.6, 132.4, 130.5, 130.4, 130.1, 129.6, 128.6, 127.7, 127.5, 125.6, 124.5, 107.1, 56.2, 52.0, 26.1, 20.2 ppm. HRMS calculated for [C_28_H_25_NO_4_ + H]^+^: 440.1862, found: 440.1853. [α]_D_^30^ – 2.90 (*c* = 1.7, CHCl_3_, 70:30 *er*). HPLC analysis: Phenomenex Lux 5u Cellulose-5, hexane/*i*-PrOH = 80:20, flow rate = 1.0 mL/min, λ = 254 nm, retention time; *t*_R_(minor) = 34.9 min, *t*_R_(major) = 44.9 min.

#### Methyl (*R*)-2-(3-(2-benzoyl-4-bromobenzyl)-1-methyl-2-oxoindolin-3-yl)acrylate (**6f**)

Synthesized following the general procedure 2 as a white solid (29.3 mg, 58% yield, 66:34 er) after flash chromatography on silica gel using petroleum ether/EtOAc 90:10 as eluent. ^1^H NMR (400 MHz, CDCl_3_): δ 7.54 (t, *J* = 7.3 Hz, 1H), 7.42–7.16 (m, 8H), 6.92 (td, *J* = 7.7, 1.0 Hz, 1H), 6.68 (d, *J* = 7.3 Hz, 1H), 6.59–6.52 (m, 2H), 6.30 (t, *J* = 7.5 Hz, 1H), 6.19 (s, 1H), 4.21 (d, *J* = 12.8 Hz, 1H), 3.48 (s, 3H), 3.38 (d, *J* = 12.8 Hz, 1H), 3.01 (s, 3H) ppm. ^13^C{^1^H} NMR (101 MHz, CDCl_3_): δ 195.7, 177.6, 165.2, 144.3, 140.2, 139.9, 136.5, 134.3, 134.2, 133.0, 132.84, 132.4, 130.5, 129.4, 128.5, 128.0, 123.5, 122.6, 119.8, 107.6, 52.0, 35.0, 26.2 ppm. HRMS calculated for [C_27_H_23_BrNO_4_ + H]^+^: 504.0811, found: 504.0804. [α]_D_^30^ – 8.80 (*c* = 0.9, CHCl_3_, 66:34 *er*). HPLC analysis: Phenomenex Lux 5u Cellulose-1, hexane/*i*-PrOH = 95:5, flow rate = 1.0 mL/min, λ = 254 nm, retention time; *t*_R_(minor) = 24.0 min, *t*_R_(major) = 38.4 min.

#### Methyl (*R*)-2-(3-(2-(4-bromobenzoyl)benzyl)-1-methyl-2-oxoindolin-3-yl)acrylate (**6g**)

Synthesized following the general procedure 2 as a white solid (26.2 mg, 52% yield, 69:31 er) after flash chromatography on silica gel using petroleum ether/EtOAc 90:10 as eluent. ^1^H NMR (400 MHz, CDCl_3_): δ 7.46 (d, *J* = 8.5 Hz, 2H), 7.34–7.27 (m, 2H), 7.17–7.01 (m, 4H), 6.92 (t, *J* = 7.7 Hz, 1H), 6.71 (d, *J* = 7.3 Hz, 1H), 6.60 (s, 1H), 6.50 (d, *J* = 7.7 Hz, 1H), 6.34 (t, *J* = 7.5 Hz, 1H), 6.25 (s, 1H), 4.32 (d, *J* = 12.6 Hz, 1H), 3.49 (s, 3H), 3.44 (d, *J* = 12.6 Hz, 1H), 2.96 (s, 3H) ppm. ^13^C{^1^H} NMR (101 MHz, CDCl_3_): δ 196.2, 177.7, 144.4, 140.3, 137.6, 136.0, 135.3, 132.9, 132.1, 131.0, 130.2, 129.8, 129.8, 128.3, 127.7, 127.5, 125.8, 123.5, 122.4, 107.4, 56.1, 52.0, 35.4, 26.1. HRMS calculated for [C_27_H_23_BrNO_4_ + H]^+^: 504.0811, found: 504.0804. [α]_D_^30^ – 17.80 (*c* = 0.7, CHCl_3_, 69:31 *er*). HPLC analysis: Phenomenex Lux 5u Cellulose-4, hexane/*i*-PrOH = 80:20, flow rate = 1.0 mL/min, λ = 254 nm, retention time; *t*_R_(major) = 35.2 min, *t*_R_(minor) = 44.0 min.

#### Methyl (*R*)-2-(3-(2-(2-fluorobenzoyl)benzyl)-1-methyl-2-oxoindolin-3-yl)acrylate (**6h**)

Synthesized following the general procedure 2 as a yellow solid (31.0 mg, 70% yield, 67:33 *er*) after flash chromatography on silica gel using petroleum ether/EtOAc 90:10 as eluent. ^1^H NMR (400 MHz, CDCl_3_): δ 7.55–7.44 (m, 1H), 7.32–7.22 (m, 2H), 7.18–7.00 (m, 6H), 6.90 (d, *J* = 7.3 Hz, 1H), 6.57 (dd, *J* = 17.5, 7.7 Hz, 3H), 6.29 (s, 1H), 4.51 (d, *J* = 12.6 Hz, 1H), 3.54 (d, *J* = 11.7 Hz, 4H), 2.99 (s, 3H) ppm. ^13^C{^1^H} NMR (101 MHz, CDCl_3_): δ 194.1, 177.8, 165.3, 161.1 (d, *J*_C–F_ = 258 Hz), 144.5, 140.4, 138.2, 135.5, 133.8, 133.7, 132.6, 132.44, 130.6, 130.6, 129.9, 128.3, 127.4, 126.6 (d, *J*_C–F_ = 10.7 Hz), 126.1, 123.7, 123.5, 123.4, 122.2, 116.4 (d, *J*_C–F_ = 22.3 Hz), 107.6, 56.2, 51.9, 35.2, 26.0 ppm. ^19^F NMR (376 MHz, CDCl_3_): δ −109.74 ppm. HRMS calculated for [C_27_H_22_FNO_4_ + H]^+^: 444.1611, found: 444.1604. [α]_D_^30^ – 3.20 (*c* = 0.9, CHCl_3_, 67:33 *er*). HPLC analysis: Phenomenex Lux 5u Cellulose-4, hexane/*i*-PrOH = 95:5, flow rate = 1.0 mL/min, λ = 254 nm, retention time; *t*_R_(major) = 20.0 min, *t*_R_(minor) = 33.4 min.

#### Ethyl (*R*)-2-(3-(2-benzoylbenzyl)-1-methyl-2-oxoindolin-3-yl)acrylate (**6i**)

Synthesized following the general procedure 2 as a white solid (25.1 mg, 57% yield, 69:31 *er*) after flash chromatography on silica gel using petroleum ether/EtOAc 90:10 as eluent. ^1^H NMR (400 MHz, CDCl_3_): δ 7.53 (t, *J* = 6.9 Hz, 1H), 7.33 (dt, *J* = 11.0, 7.7 Hz, 6H), 7.12 (d, *J* = 6.2 Hz, 2H), 6.92 (t, *J* = 7.2 Hz, 1H), 6.73 (d, *J* = 7.3 Hz, 1H), 6.63 (s, 1H), 6.51 (d, *J* = 7.7 Hz, 1H), 6.33 (t, *J* = 7.5 Hz, 1H), 6.23 (s, 1H), 4.34 (d, *J* = 12.7 Hz, 1H), 3.98–3.82 (m, 2H), 3.47 (d, *J* = 12.6 Hz, 1H), 2.98 (s, 3H), 1.00 (t, *J* = 7.1 Hz, 3H) ppm. ^13^C{^1^H} NMR (101 MHz, CDCl_3_): δ 197.3, 177.8, 164.9, 140.5, 138.1, 137.3, 135.2, 132.6, 132.5, 130.6, 130.1, 129.9, 129.9, 128.2, 127.7, 127.5, 125.7, 123.5, 122.5, 107.2, 60.7, 56.0, 26.0, 13.7 ppm. HRMS calculated for [C_28_H_26_NO_4_ + H]^+^: 440.1862, found: 440.1853. [α]_D_^30^ – 18.90 (c = 0.2, CHCl_3_, 69:31 *er*). HPLC analysis: Phenomenex Lux 5u Cellulose-4, hexane/*i*-PrOH = 80:20, flow rate = 1.0 mL/min, λ = 254 nm, retention time; *t*_R_(major) = 15.9 min, *t*_R_(minor) = 25.2 min.

#### Methyl (*R*)-2-(1-allyl-3-(2-benzoylbenzyl)-2-oxoindolin-3-yl)acrylate (**6j**)

Synthesized following the general procedure 2 as an orange solid (23.5 mg, 52% yield, 70:30 *er*) after flash chromatography on silica gel using petroleum ether/EtOAc 90:10 as eluent. ^1^H NMR (400 MHz, CDCl_3_): δ 7.49 (tt, *J* = 5.7, 3.3 Hz, 1H), 7.35–7.24 (m, 7H), 7.16–7.07 (m, 2H), 6.81 (t, *J* = 7.3 Hz, 1H), 6.73 (d, *J* = 7.3 Hz, 1H), 6.59 (s, 1H), 6.49 (d, *J* = 7.8 Hz, 1H), 6.32–6.24 (m, 2H), 5.45 (ddt, *J* = 15.7, 10.4, 5.2 Hz, 1H), 5.07–4.99 (m, 1H), 4.84–4.76 (m, 1H), 4.41–4.27 (m, 2H), 3.97 (dd, *J* = 16.5, 5.5 Hz, 1H), 3.50 (s, 4H) ppm. ^13^C{^1^H} NMR (101 MHz, CDCl_3_): δ 197.3, 177.4, 165.2, 143.8, 140.6, 138.2, 137.1, 135.2, 133.3, 132.5, 131.5, 130.6, 130.3, 130.1, 129.5, 128.2, 127.7, 127.4, 125.7, 123.8, 122.3, 117.1, 108.4, 56.0, 51.9, 42.5 ppm. HRMS calculated for [C_29_H_26_NO_4_ + H]^+^: 452.1862, found: 452.1859. [α]_D_^30^ – 17.80 (*c* = 0.7, CHCl_3_, 70:30 *er*). HPLC analysis: Phenomenex Lux 5u Cellulose-4, hexane/*i*-PrOH = 95:5, flow rate = 1.0 mL/min, λ = 254 nm, retention time; *t*_R_(major) = 16.1 min, *t*_R_(minor) = 25.1 min.

### General Procedure for the Light-Triggered Catalytic Asymmetric Allylic Benzylation of **5** under a Microfluidic Photoreactor (MFP) in Large Scale

Large-scale synthesis of benzylated product **6** is performed using the optimal MFP conditions.^13^ To an oven-dried round-bottom flask, the MBH carbonate **5** (1 equiv) and β-isocupreidine **4a** (10 mol %) are added under argon atmosphere. The solids are dissolved in argon-degassed anhydrous MeOH (0.05 M), and 2-methylbenzophenone **1a** (5 equiv) is subsequently added in one portion. After further degassing with argon for 30 min, the solution is reacted using two parallel MFP setups with a flow rate of 13.3 μL/min (30 min residence time). Once all of the solution is flowed through the photoreactor, the crude product solution is treated with water and extracted three times with EtOAc. The organic layers are dried with anhydrous MgSO_4_ and evaporated to afford the crude product, which is subsequently purified by flash chromatography to afford the pure benzylated products **6a** (1 mmol scale, 290 mg, 68% yield, 70:30 *er*) and **6c** (2 mmol scale, 639 mg, 72% yield, 72:28 *er*).

## Supporting Information Available

Spectra of starting material; products and catalytic intermediates; optimization studies; photoisomerization experiments; HPLC traces; X-ray diffraction as well as DFT-ECD simulations for absolute configuration determination (PDF)
Crystallographic data (**6a**) (CIF)

## Supplementary Material

jo0c00175_si_001.pdf

jo0c00175_si_002.cif
