# Supplementary material for: Light-Triggered Catalytic Asymmetric Allylic Benzylation with Photogenerated C-Nucleophiles
Source: J Org Chem. 2020 Feb 21;85(6):4463–74. doi: 10.1021/acs.joc.0c00175 (PMC7997570; doi:10.1021/acs.joc.0c00175)

Supporting Information

Light-triggered Catalytic Asymmetric Allylic Benzylation with Photogenerated *C*-Nucleophiles

Suva Paria,[a] Edoardo Carletti,[a,†] Michela Marcon,[a,†] Alessio Cherubini-Celli,[a,†] Andrea Mazzanti,[b] Marzio Rancan,[c] Luca Dell'Amico,[a] Marcella Bonchio[a] and Xavier Companyó*,[a]

[a] Department of Chemical Sciences, University of Padova, via Marzolo 1, 35131 Padova, Italy

[b] Department of Industrial Chemistry "Toso Montanari", University of Bologna, viale del Risorgimento 4, 40136 Bologna, Italy

[c] Institute of Condensed Matter Chemistry and Technologies for Energy (ICMATE), National Research Council (CNR), Department of Chemical Sciences, University of Padova, via Marzolo 1, 35131 Padova, Italy

E-mail: xavier.companyo@unipd.it

Table of Contents

A. General Information **S-3**

B. Supplementary figures **S-4**

C. Asymmetric allylic benzylation of aldehyde-derived MBH carbonates for the formation of tertiary allylic carbons. **S-7**

C.1. General procedure S-7

C.2. Optimization of the reaction conditions S-9

D. Asymmetric allylic benzylation of isatin-derived MBH carbonates for the formation of quaternary allylic carbons **S-13**

D.1. General procedure S-13

D.2. Optimization of the reaction conditions S-13

D.3. Optimization in microfluidic conditions S-17

E. Isomerization studies **S-19**

E.1. Catalytic intermediate **IIa** derived from methyl MBH carbonate **2a** and β-ICP **4a** S-19

E.2. Catalytic intermediate **IIIg/IV**g derived from 2-naphtyl MBH carbonate **2g** and β-ICP **4a** S-24

E.3. Catalytic intermediate **Va** derived from isatin MBH carbonate **5a** and β-ICP **4a** S-28

F. Determination of the absolute configuration **S-36**

F.1. X-Ray diffraction of compound **6a** S-36

F.2. Absolute configuration of compound **6a** S-38

F.3. Absolute configuration of compound **3g** S-55

G. NMR spectra & HPLC traces **S-82**

H. References **S-135**

A. General Information

NMR spectra were recorded on Bruker Avance DPX 200 equipped with a QNP probehead, Bruker 400 Avance III HD equipped with a BBI-z grad probehead 5mm, and a Bruker 500 Avance III equipped with a BBI-ATM-z grad probehead 5mm. The chemical shifts (δ) for ^{1}H and ^{13}C are given in ppm relative to residual signals of the solvents ($CDCl_3$ @ 7.26 ppm ^{1}H NMR, 77.16 ppm ^{13}C NMR). Coupling constants are given in Hz. The following abbreviations are used to indicate the multiplicity: s, singlet; d, doublet; t, triplet; q, quartet; m, multiplet; bs, broad signal. NMR yields were calculated by using pyrazine as internal standard.

High-Resolution Mass Spectra (HRMS) were obtained using Waters GCT gas chromatograph coupled with a time-of-flight mass spectrometer (GC/MS-TOF) with electron ionization (EI) or MicroTOF II (Bruker Daltonics): HPLC-MS-TOF (ESI).
Chromatographic purification of products was accomplished using flash chromatography on silica gel (SiO_2, 0.04-0.063 mm) purchased from Machery-Nagel, with the indicated solvent system according to the standard techniques. Thin-layer chromatography (TLC) analysis was performed on pre-coated Merck TLC plates (silica gel 60 GF254, 0.25 mm). Visualization of the developed chromatography was performed by checking UV absorbance (254nm) as well as with aqueous potassium permanganate solutions. Organic solutions were concentrated under reduced pressure on a Büchi rotary evaporator.

Determination of Enantiomeric Purity: HPLC analysis on chiral stationary phase was performed on a UHPLC Agilent 1290 Infinity, using Phonemenex Lux 5u Cellulose-1, Lux 5u Cellulose-4 and Lux 5u Cellulose-5 chiral columns. The exact conditions for the analyses are specified within the characterization section. HPLC traces were compared to racemic samples prepared performing the reactions using DABCO as catalyst.

Microfluidic Photoreactor: The continuous flow reactions were carried out using capillary reactors made with PTFE tubing (0.75 mm I.D., 1.58 mm O.D.) and fitting connections purchased from Sigma-Aldrich. Reagents were pumped using a Syrris Asia pump (https://syrris.com/modules/asia-syringe-pump/). 9W 365 nm bulb lamps were purchased from Amazon (https://www.amazon.it/Foxnovo-sostituzione-lampadina-essiccatore-lampada/dp/B00JKE1T70). *High-power* 365 nm LEDs were purchased from OSA Opto Light GmbH (model OCU-440 UE365-X-T https://www.osa-opto.com/smd-leds/ocu-440-ue365.html).

Materials: Commercial grade reagents and solvents were purchased at the highest commercial quality from Sigma Aldrich, Alfa Aesar, TCI and Fluka and used as received, unless otherwise stated. Catalysts **4e-k, s, t** were prepared as reported in the literature.[1]

The authors are grateful to Prof. Jose Luis Vicario group, from the University of the Basque Country (UPV/EHU), for the generous sending of the bifunctional phosphine catalysts **4s-t**.

B. Supplementary figures

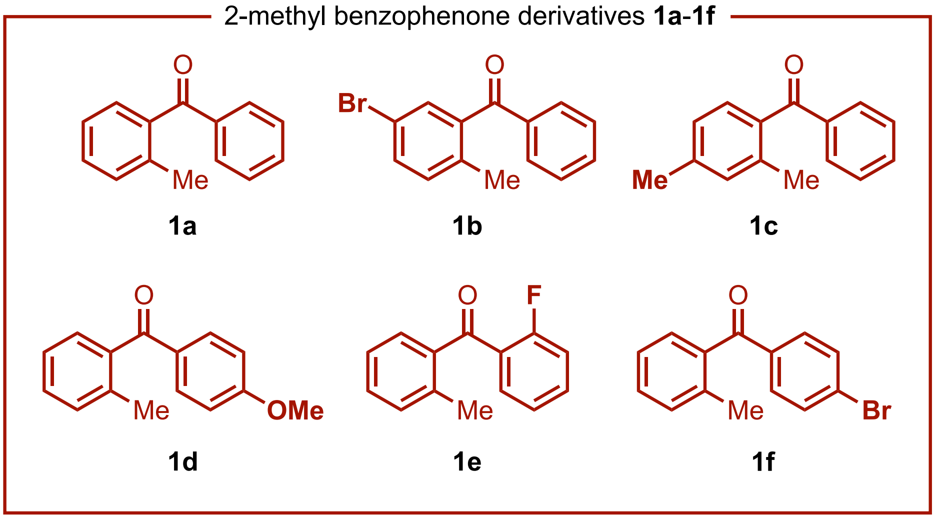

Figure S-1. 2-methyl benzophenone derivatives (**1a-f**) used in this work

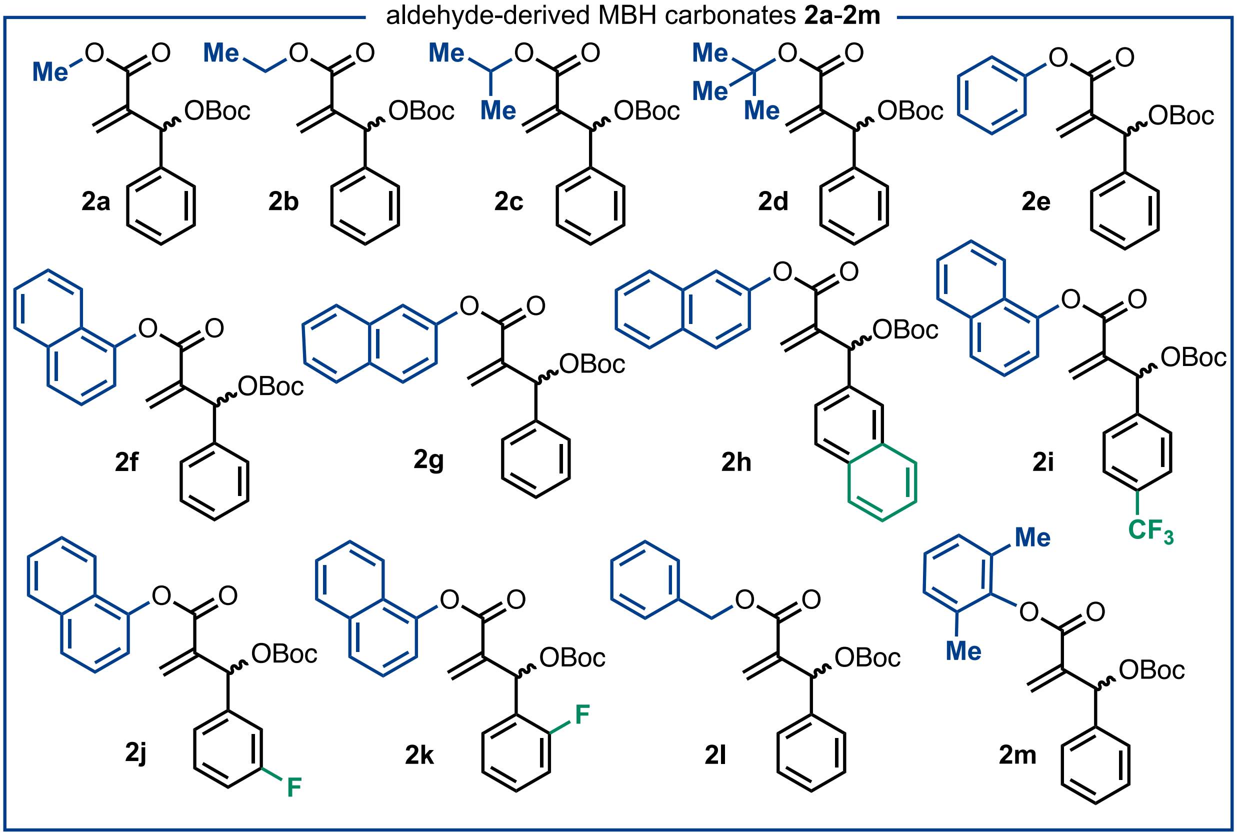

Figure S-2. Aldehyde-derived Morita-Baylis-Hillman carbonates (**2a-m**) used in this work

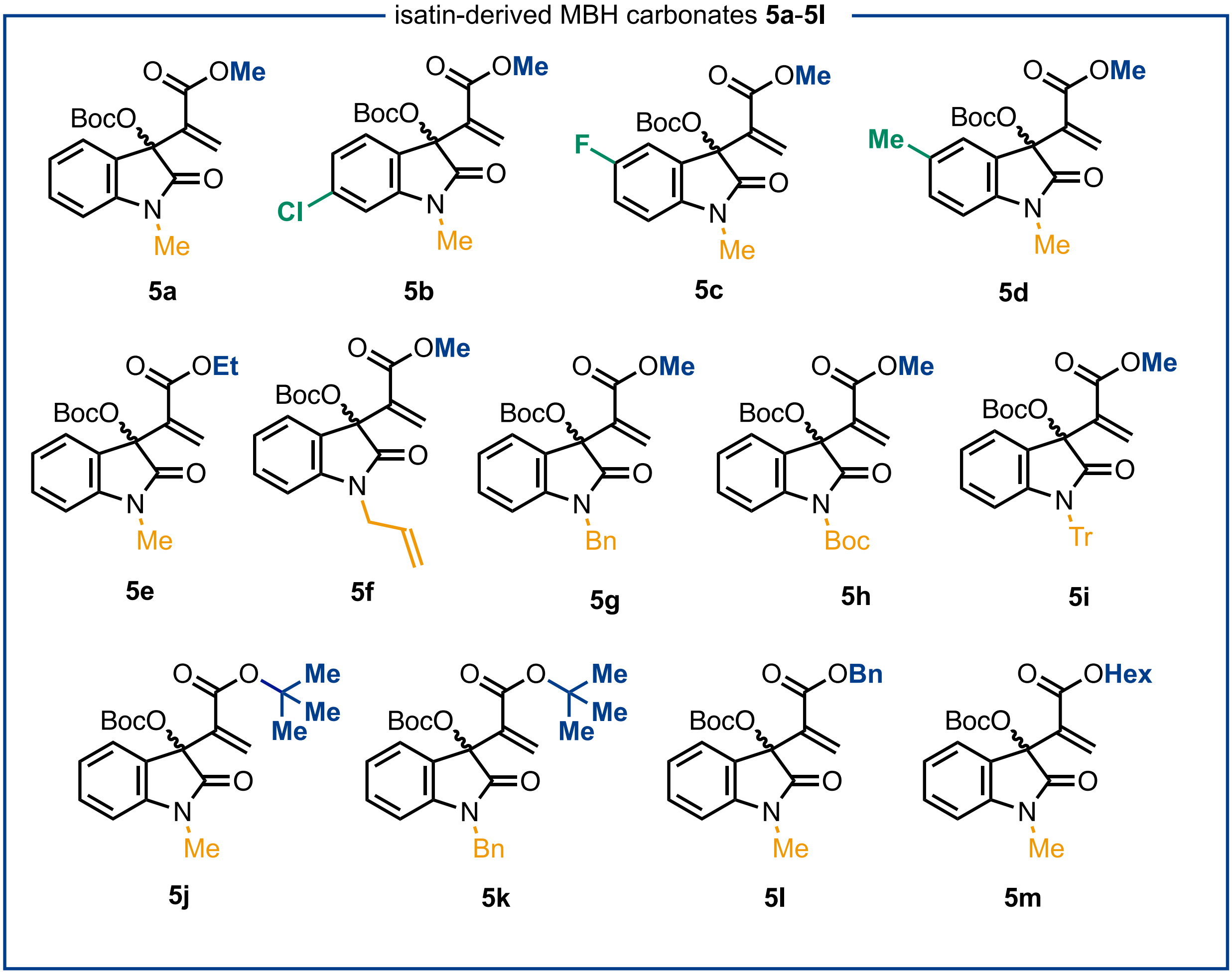

Figure S-3. Isatin-derived Morita-Baylis-Hillman carbonates (**5a-l**) used in this work

The spectra shown in Figures S-4 and S-5 were recorded by an AvaSpec ULS3648 high-resolution fiber-optic spectrometer which was placed at a fixed distance of 0.5 cm from the light source.

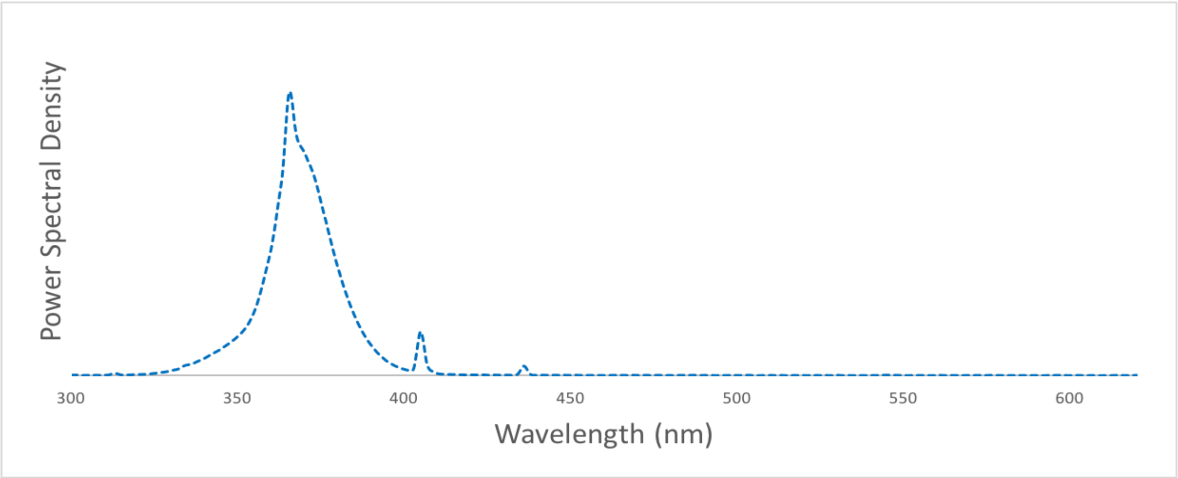

Figure S-4. Emission spectra of the 9W 365nm bulb light used in this study.

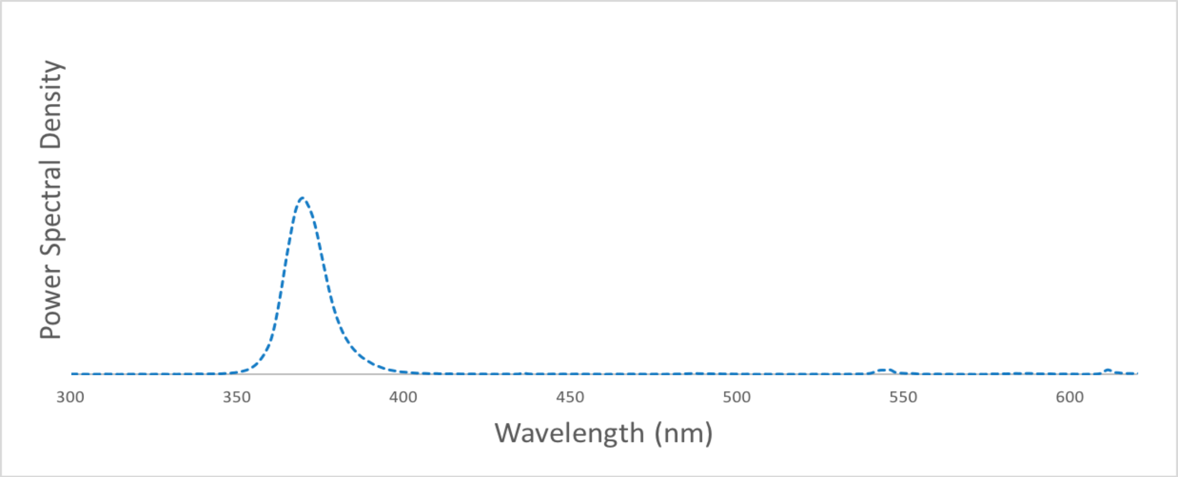

Figure S-5. Emission spectra of the LEDs used in this study.

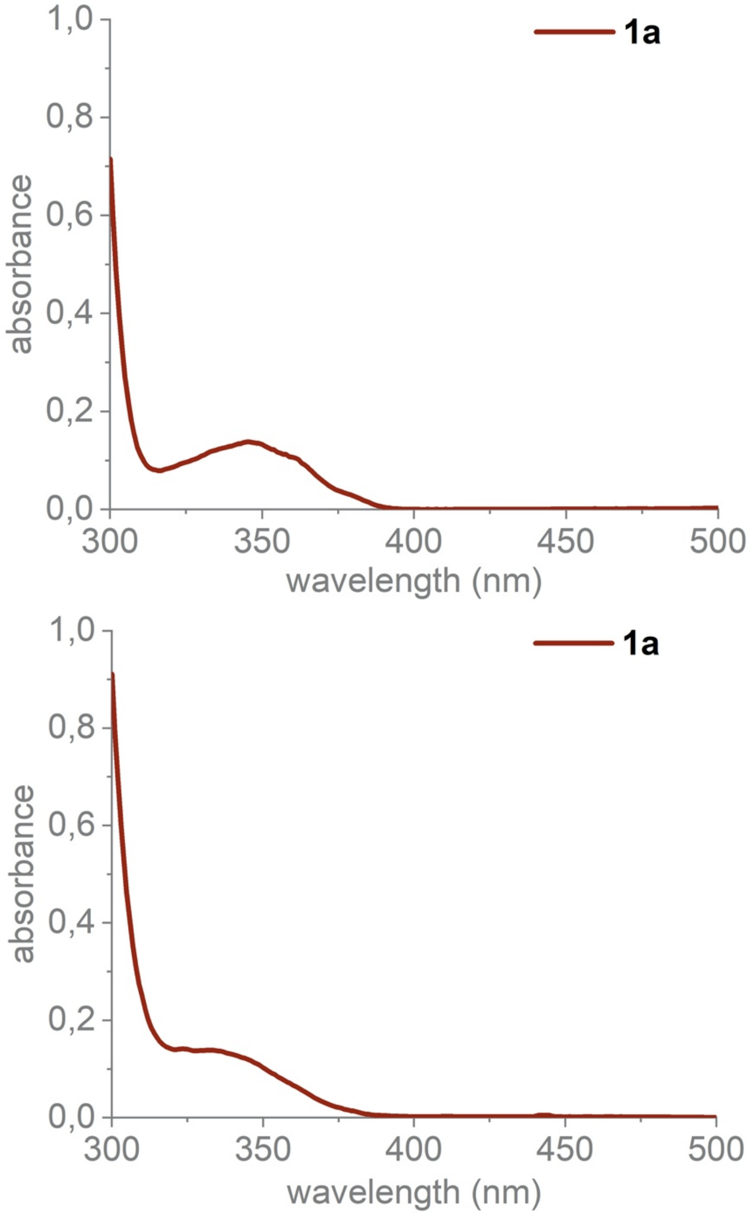

Figure S-6. Absorption spectra of 2-methyl benzophenone **1a** (10^{-3}M) in toluene (up) and MeOH (down).

C. Asymmetric allylic benzylation of aldehyde-derived MBH carbonates for the formation of tertiary allylic carbons.

C.1. General procedure

To an oven dried 4 mL screw cap vial equipped with a septum was added 4Å activated molecular sieve (20 mg) and the MBH carbonate **2** (0.1 mmol, 1 equiv.). Subsequently, the vial was purged with argon and β-isocupreidine **4a** (10 mol%) was added. Previously degassed anhydrous toluene (1 mL) was introduced to the vial followed by 2-methylbenzophenone derivative **1** (5 equiv.). After a final purge with argon, the vial was sealed with parafilm and irradiated with the selected photochemical setup (see Figure S-7 and Figure S-8). After 3h, the reaction mixture was directly loaded into a silica gel column chromatography and purified using mixtures of petroleum ether/EtOAc as eluent to furnish enantioenriched benzylated products **3**.
The racemic samples were prepared following the same procedure using DABCO (10 mol%) as catalyst.

Figure S-7 shows the 9W 365nm photochemical setup. The reaction vials in front of the 9W 365 nm bulb at approximatively 1.5 cm distance. To maintain a stable reaction temperature two fans were placed in close proximity to the reaction vials (25±2 °C) and the temperature was controlled by a thermometer.

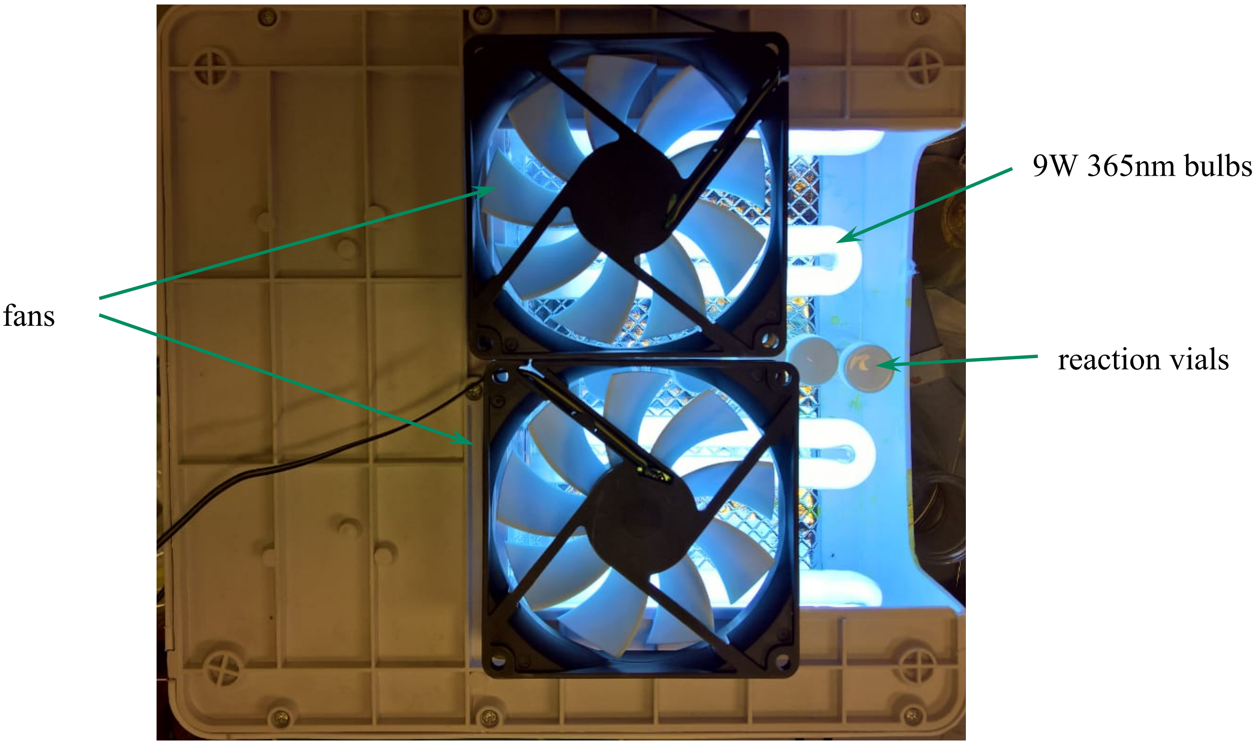

Figure S-7. 9W 365nm photochemical setup used in this work.

Figure S-8 shows the high-power 365nm LED photochemical setup. The reaction vial is inserted into an aluminum equipped with the *HP* 365 nm LED and connected to a chiller that enables to set the desired temperature. A thermometer in the middle of the aluminium block allows to measure the actual reaction temperature. A glass bell filled with a constant flow of N2 avoid the

condensation and freezing of the atmospheric moisture in the photochemical setup when working at low temperatures.

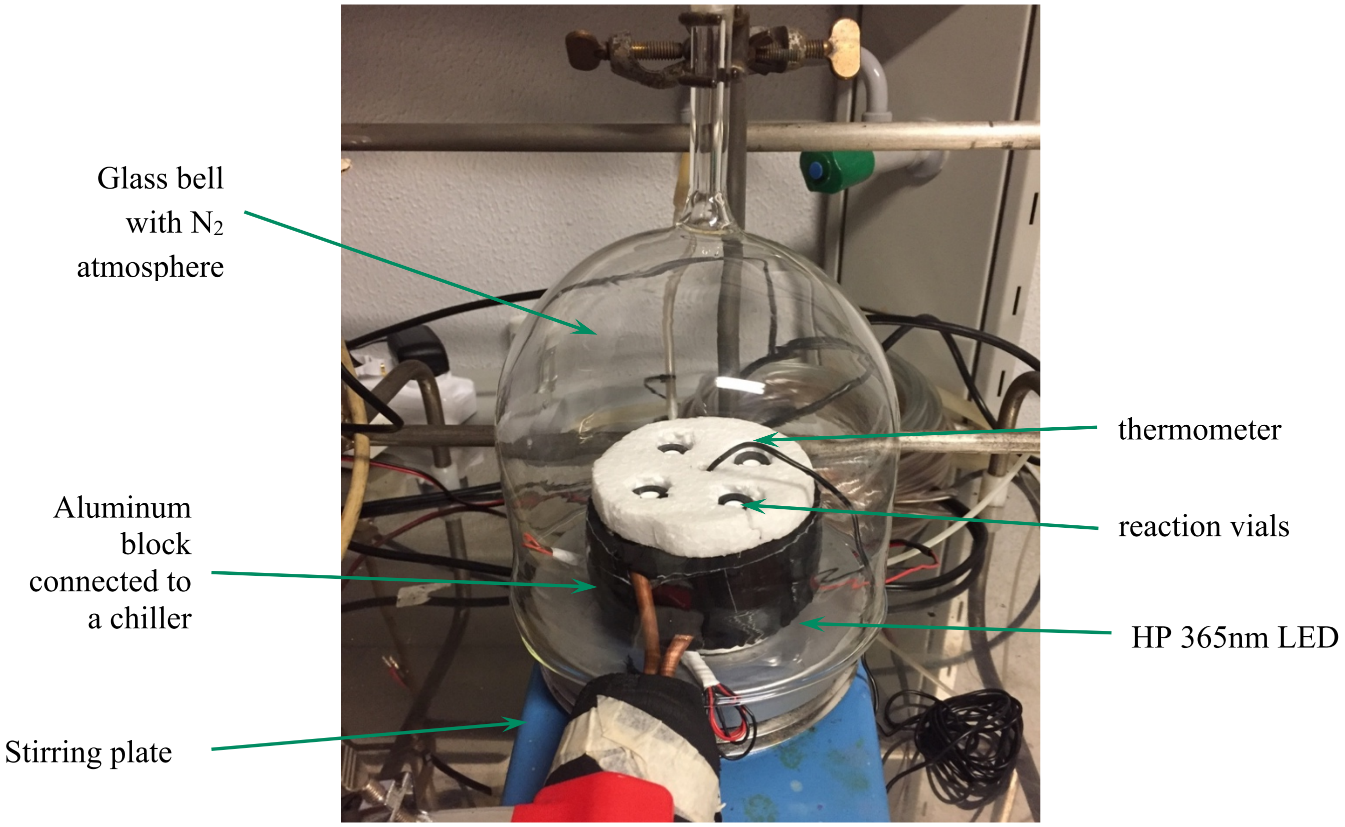

Figure S-8. High-power 365nm LED setup used in this work.

Figure S-9 shows the 9W 365nm setup for the photochemical semi-continuous ^{1}H NMR experiments used in this work to monitor the photoisomerization of the diverse catalytic species (note that the fans used to maintain stable the temperature were removed to take the picture).

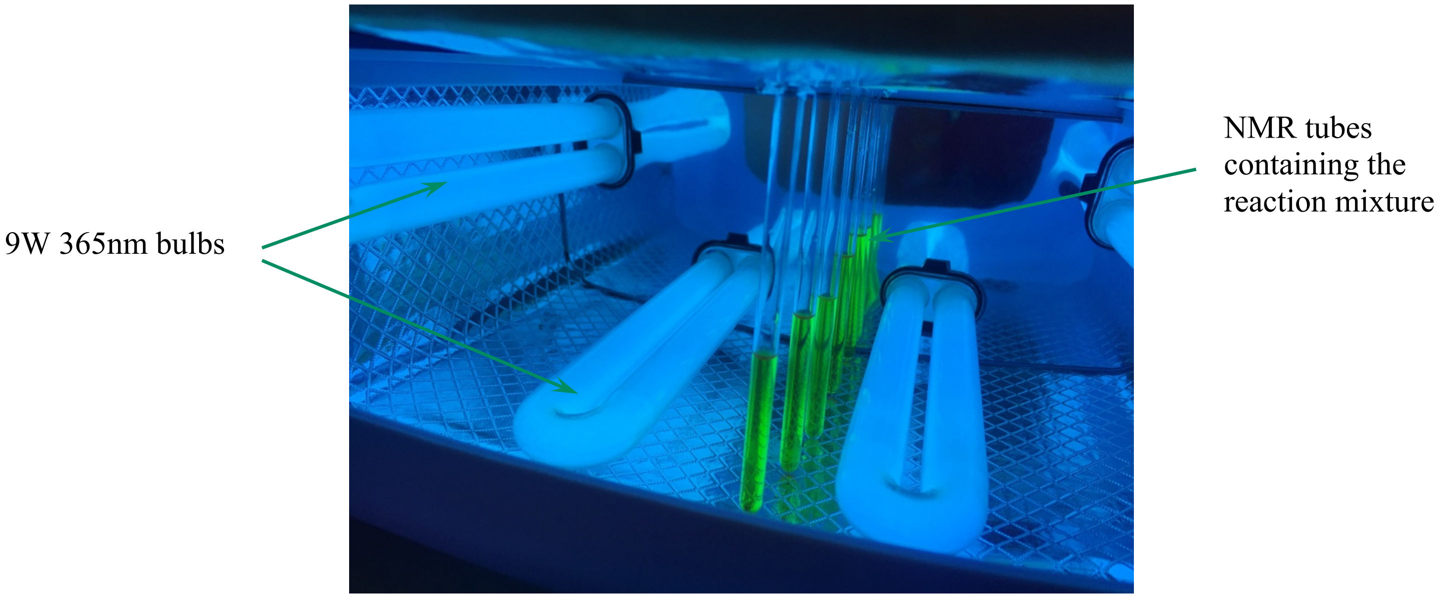

Figure S-9. 9W 365nm setup for photochemical semi-continuous ^{1}H NMR experiments

C.2. Optimization of the reaction conditions

Table S-1. Screening of chiral Lewis catalysts

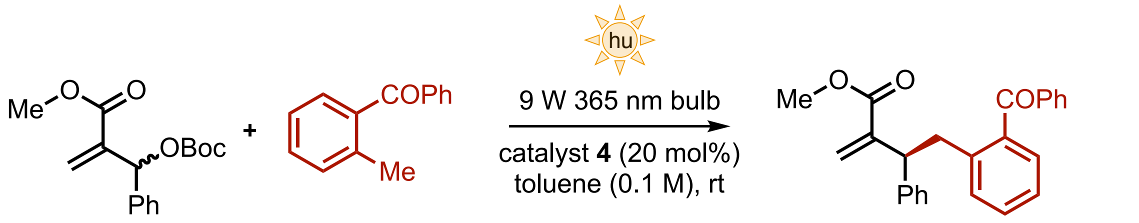

2a **1a** **3a**

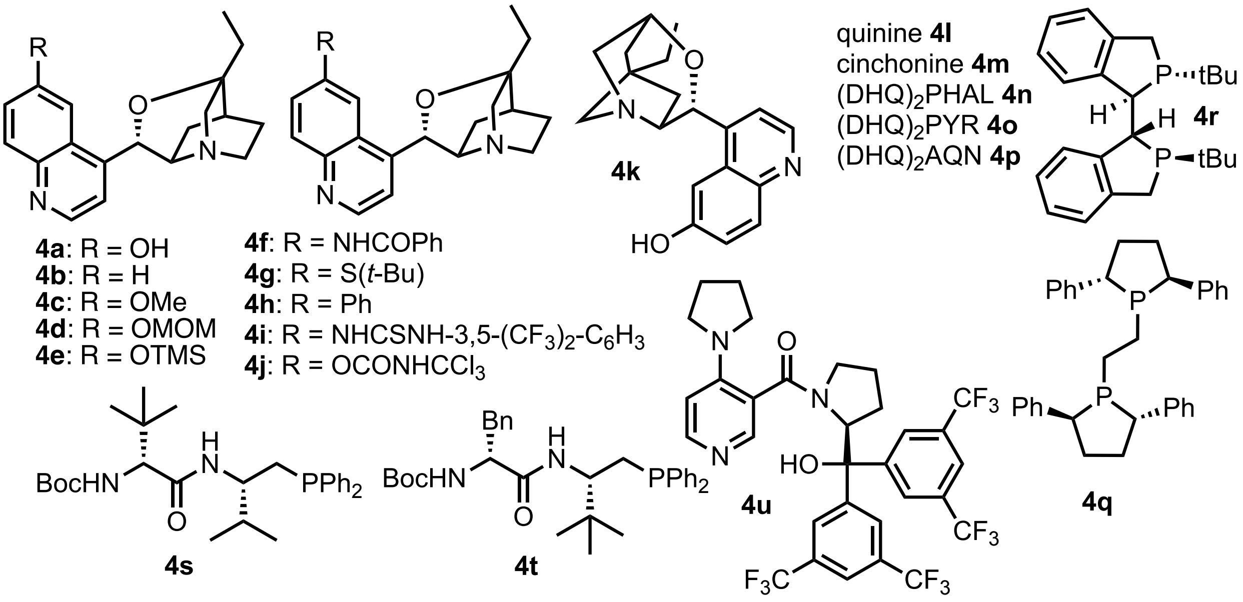

entry	catalyst	yield (%) of **3a**[b]	*er* of **3a**[c]
1	**4a**	39	65:35
2	**4b**	10	64:36
3	**4c**	53	63:37
4	**4d**	42	65:35
5	**4e**	48	64:36
6	**4f**	20	65:35
7	**4g**	43	57:43
8	**4h**	64	50:50
9	**4i**	21	52:48
10	**4j**	35	59:41
11	**4k**	14	60:40
12	**4l**	n.r.	-
13	**4m**	n.r.	-
14	**4n**	n.r.	-
15	**4o**	n.r.	-
11	**4p**	n.r.	-
12	**4q**	72	55:45
13	**4r**	n.r.	-
14	**4s**	n.r.	-
15	**4t**	n.r.	-
16	**4u**	23	74:26

[a]Reaction conditions: 0.1 mmol of **2a** (1 equiv.), 2-methylbenzophenone **1a** (5 equiv.), catalyst **4a-p** (20 mol%) in 1 mL of toluene irradiated with the 9W 365 nm bulb for 3h. [b]Determined by ^{1}H-NMR analysis using pyrazine as internal standard. [c]Determined by chiral HPLC analysis. n.r.= no reaction; n.d.= not determined

Next, we screen different solvents using β-isocupreidine **4a** as catalyst.

Table S-2. Screening of solvents[a]

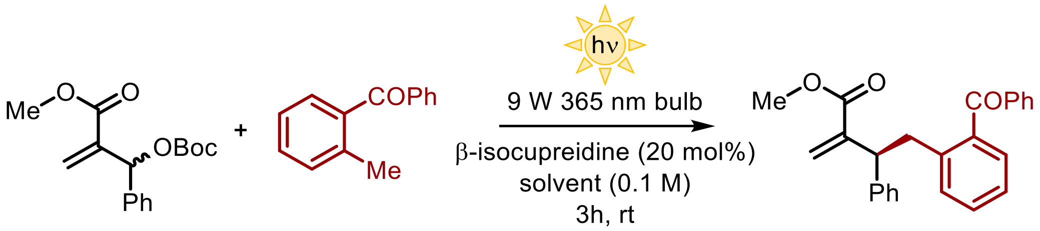

2a **1a** **3a**

entry	solvent	yield (%) of **3a**[b]	*er* of **3a**[c]
1	toluene	39	65:35
2	trifluorotoluene	14	59:41
3	1,2-dichlorobenzene	32	57:43
4	CH_2Cl_2	37	55:45
5	MeCN	21	59:31
6	THF	n.r.	-
7	Et_2O	n.r.	-
8	MeOH	65	73:27
9	*i*-PrOH	31	57:43
10	*t*-BuOH	44	67:33
11	hexafluoroisopropanol	n.r.	-

[a]Reaction conditions: 0.1 mmol of **2a** (1 equiv.), 2-methylbenzophenone **1a** (5 equiv.), β-isocupreidine (20 mol%) in 1 mL of solvent was irradiated at 365 nm for 3h. [b]Determined by ^{1}H-NMR analysis using pyrazine as internal standard. [c]Determined by chiral HPLC analysis

The blank reactions were performed to evaluate whether a potential uncatalyzed pathway could take place.

Table S- 3. Blank reactions

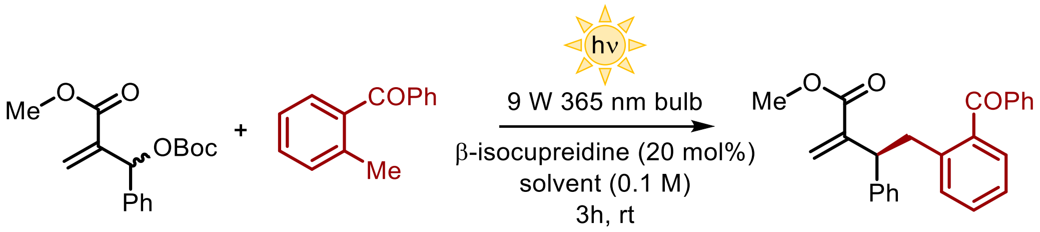

2a **1a** **3a**

entry	solvent	deviation from standard conditions	yield (%) of **3a**[b]	*er* of **3a**[c]
1	toluene	no catalyst **4a**	n.r.	-
2	toluene	no light irradiation	n.r.	-
3	MeOH	no catalyst **4a**	n.r.	-
4	MeOH	no light irradiation	n.r.	-

[a]Reaction conditions: 0.1 mmol of **2a** (1 equiv.), 2-methylbenzophenone **1a** (5 equiv.), β-isocupreidine (20 mol%) in 1 mL of solvent was irradiated at 365 nm for 3h. [b]Determined by ^{1}H-NMR analysis using pyrazine as internal standard. [c]Determined by chiral HPLC analysis

Having found methanol to afford the product in improved yield and enantioselectivity, we decided to screen different ester groups in MBH carbonates

Table S-4. Screening of MBH carbonates bearing different aliphatic ester groups[a]

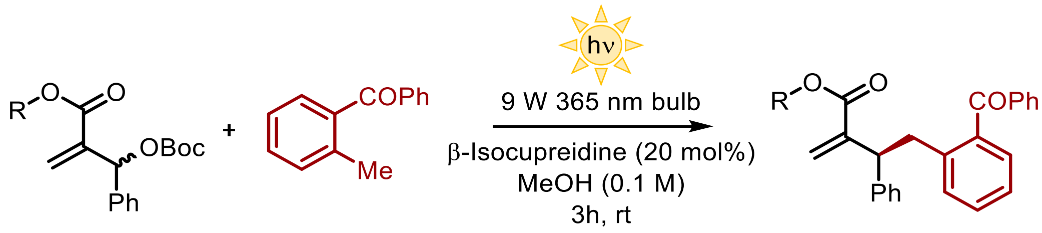

2 **1a** **3**

entry	R	yield (%) of **3**[b]	*er* of **3**[c]
1	Me (**2a**)	65	73:27
2	Et (**2b**)	32	75:25
3	*i*-Pr (**2c**)	42	81:19
4	*t*-Bu (**2d**)	9	90:10
5	Bn (**2l**)	12	73:27

[a]Reaction conditions: 0.1 mmol of **2** (1 equiv.), 2-methylbenzophenone **1a** (5 equiv.), β-isocupreidine (20 mol%) in 1 mL of MeOH was irradiated at 365 nm for 3h. [b]Determined by ^{1}H NMR analysis using pyrazine as internal standard. [c]Determined by chiral HPLC analysis

Although a positive correlation between size of the aliphatic ester moiety and the e.r. of the product could be observed, it was associated with decrease in the yield. The isopropyl substituted ester **2c** afforded the corresponding product with a promising 42% of yield and 81:19 e.r.. However, the difficult and hazard synthesis of the corresponding isopropyl acrylate prompted us to stop using this substrate.

Next, we turned our attention to different aryl acrylates. In this case, toluene was used as solvent since transesterification was found to be operative when MeOH was used as solvent forming the corresponding methyl ester product (**3a**).

Table S-5. Screening of MBH carbonates bearing different aromatic ester groups[a]

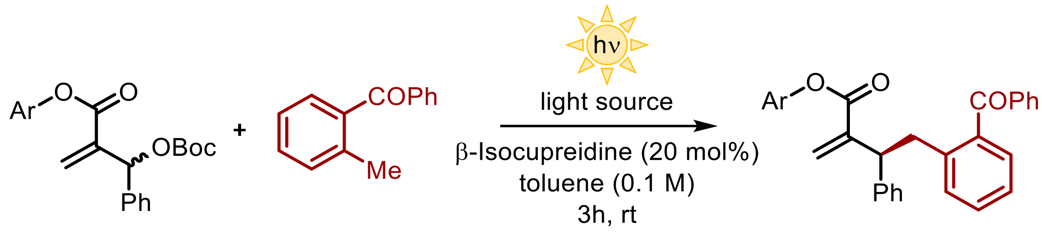

2 **1a** **3**

entry	Ar	yield (%) of **3**[b]	*er* of **3**[c]
1	Ph (**2e**)	60	76:24
2	2,6-$Me_2C_6H_3$ (**2m**)	53	80:20
3	1-naphtyl (**2f**)	59	80:20
4	2-naphtyl (**2g**)	63	78:22

[a]Reaction conditions: 0.1 mmol of **2** (1 equiv.), 2-methylbenzophenone **1a** (5 equiv.), β-isocupreidine (20 mol%) in 1 mL of MeOH was irradiated at 365 nm for 3h. [b]Determined by ^{1}H NMR analysis using pyrazine as internal standard. [c]Determined by chiral HPLC analysis

Having found the 2-naphthyl MBH carbonate to be the best substrate, we screened different reaction conditions including solvent, temperature, light source, additives and catalyst loading.

Table S- 6. Screening of other reaction conditions.

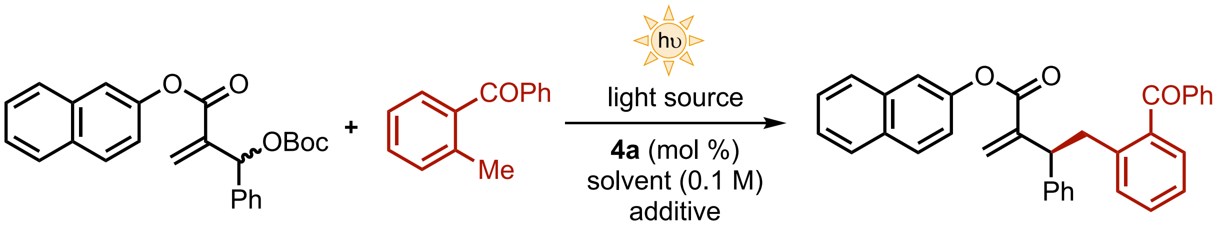

entry	**4a** (mol %)	solvent	additive	yield (%) of **3**[b]	*er* of **3**[c]
1	20	toluene	none	63	78:22
2	20	mesitylene	none	52	79:21
3[d]	20	cyclohexane	none	17	73:27
4	10	toluene	none	65	78:22
5	5	toluene	none	45	78:22
6	10	toluene	*t*-BuOH (1 equiv.)	33	76:24
7[e]	10	toluene	none	46	78:22
8[f]	10	toluene	none	32	77:23
**9**	**10**	**toluene**	**MS 4 Å (20 mg)**	**66 (62)**	**80:20**
10[g]	10	toluene	MS 4 Å (20 mg)	27	80:20
**11[h]**	**10**	**toluene**	**MS 4 Å (20 mg)**	**63 (55)**	**82:18**

[a]Reaction conditions: 0.1 mmol of **2g** (1 equiv.), 2-methylbenzophenone **1a** (5 equiv.) irradiated at 365 nm. [b]Determined by ^{1}H NMR analysis using pyrazine as internal standard, isolated yields are provided in parenthesis. [c]Determined by chiral HPLC analysis. [d]Heterogeneous reaction mixture. [e]2 equiv. of **1a** were used. [f]Irradiation with HP 365 nm single LED (current 200 mA). [g]Irradiation at 10 °C with HP 365 nm single LED (current 200 mA).[h]1-naphthyl MBH carbonate (**2f**) was used as substrate

D. Asymmetric allylic benzylation of isatin-derived MBH carbonates for the formation of quaternary allylic carbons.

D.1. General procedure

To an oven dried 4 mL screw cap vial were added MBH carbonate **5a-f** (0.1 mmol, 1 equiv.) and β-isocupreidine **4a** (0.1 equiv.) under argon. Previously degassed anhydrous MeOH (1 mL) was introduced to the vial followed by 2-methylbenzophenone derivative **1a-e** (5 equiv.). The vial was sealed with parafilm and irradiated under 365 nm light source at 350 mA (see Figure S-8 High-power 365nm LED setup). After total consumption of the starting material (TLC analysis), the reaction mixture was directly loaded into a silica gel column chromatography and purified using mixtures of petroleum ether/EtOAc as eluent to furnish the enantioenriched benzylated products **6a-j**.

Racemic samples were prepared following the same procedure using 20 mol% of DABCO as catalyst.

D.2. Optimization of the reaction conditions

Table S-7. Screening of amide-protecting and ester groups in toluene[a]

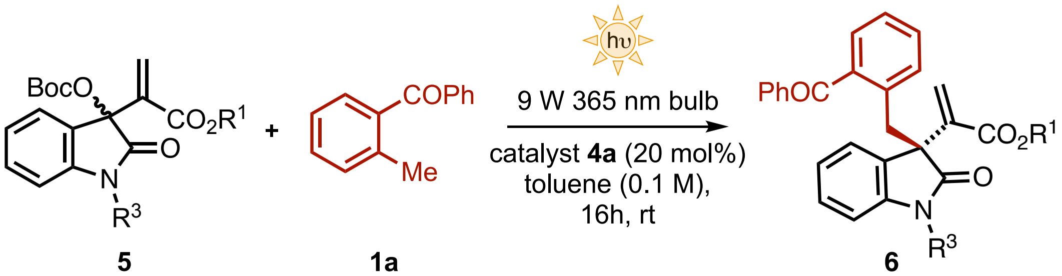

entry	R^1	R^3	**5**	yield (%) of **3**[b]	*er* of **3**[c]
1	Me	Me	**5a**	24	72:28
2	Me	Bn	**5g**	15	75:25
3	Me	Boc	**5h**	traces	n.d.
4	Me	Tr	**5i**	traces	n.d.
5	t-Bu	Me	**5j**	15	82:18
6	t-Bu	Bn	**5k**	10	76:26

[a]Reaction conditions: 0.1 mmol of **2** (1 equiv.), 2-methylbenzophenone **1a** (5 equiv.), β-isocupreidine (20 mol%) in 1 mL of MeOH was irradiated at 365 nm for 3h. [b]Determined by ^1H NMR analysis using pyrazine as internal standard. [c]Determined by chiral HPLC analysis

The initial assessment of the amide-protecting and the ester groups in **5** furnished the corresponding benzylated products **6** in very low yields albeit with full consumption of the MBH carbonate. This fact was attributed to the decomposition of the starting materials in the presence of catalyst **4a** in toluene.

The stability of the substrate **5** in different solvents in the presence of catalyst **4a** was investigated. As shown in Figure S-10, the starting material **5a** with 20% of **4a** is only stable in MeOH while in the other solvents, after 19h, the remaining starting material **5** spanned from 40% (for CD_2Cl_2) to 20% (for toluene_d$_8$). Therefore, in order to find suitable synthetic conditions, the subsequent optimizations were conducted using N-Me protected MBH carbonate **5a** with polar protic solvents.

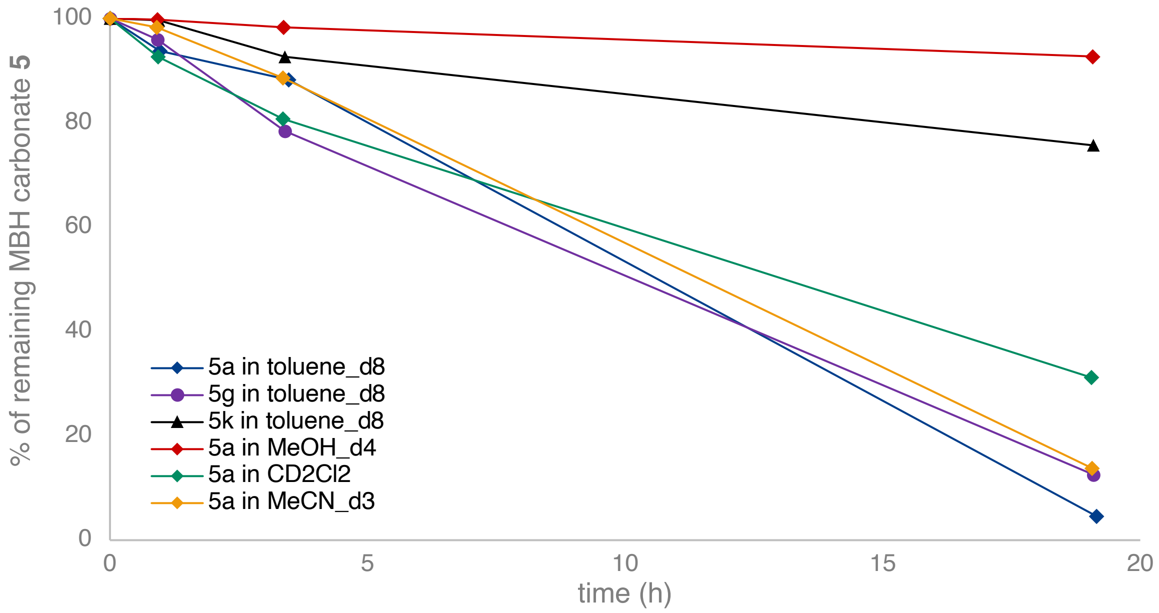

Figure S-10. Stability of 5 in the presence of 20 mol% of 4a in different solvents.

To rule out the possibility of potential uncatalyzed reaction pathway, the blank reactions were performed. As shown in Table S-8, in the absence of both chiral Lewis base or UV-light the reaction does not take place, confirming thus the proposed dual activation strategy.

Table S-8. Blank reactions

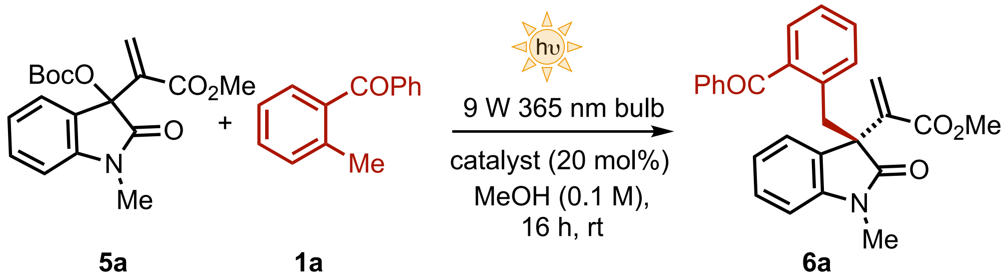

entry	solvent	deviation from standard conditions	yield (%) of **3a**[b]	*er* of **3a**[c]
1	MeOH	no catalyst **4a**	n.r.	-
2	MeOH	no light irradiation	n.r.	-

[a]Reaction conditions: 0.1 mmol of **5a** (1 equiv.), 2-methylbenzophenone **1a** (5 equiv.), catalyst **4** (20 mol%) in 1 mL of MeOH was irradiated at 365 nm for 3h. [b]Determined by ^{1}H NMR analysis using pyrazine as internal standard.

Subsequently, the effect of different chiral Lewis base catalyst was evaluated in MeOH, confirming β-ICP **4a** as the best catalyst.

Table S-9. Screening of chiral Lewis base catalysts

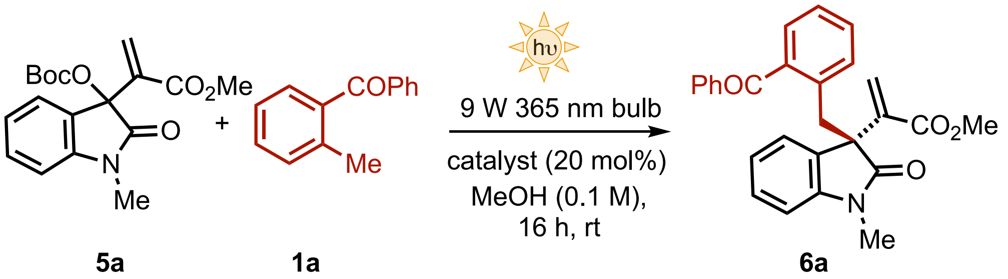

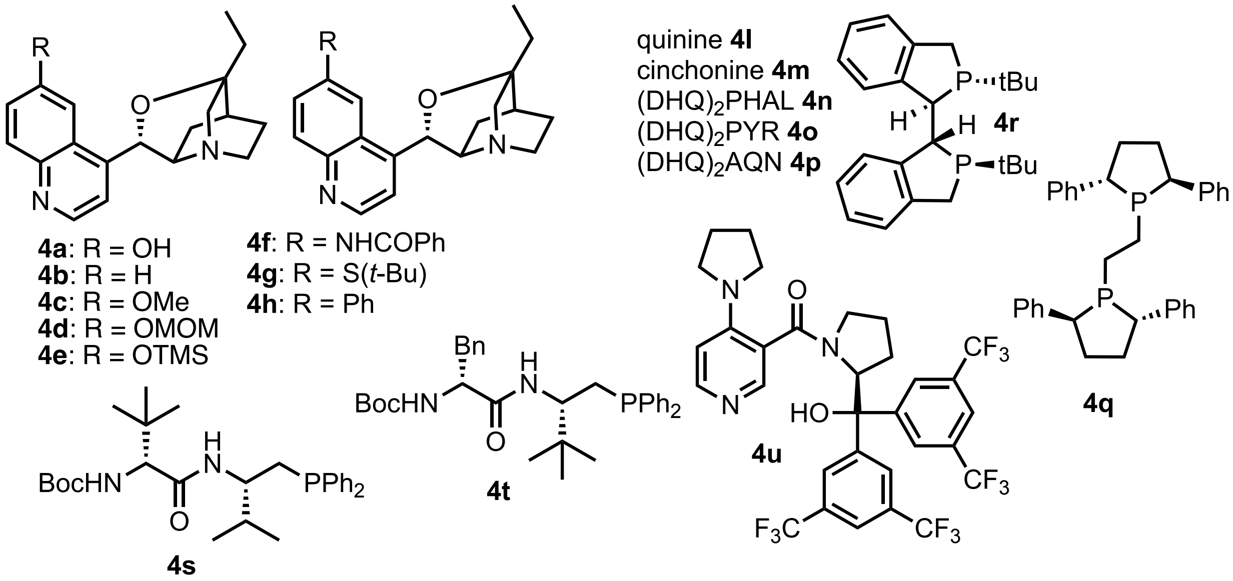

entry	catalyst **4**	yield (%) of **6a**[b]	*er* of **6a**[c]
1	**4a**	66	70:30
2	**4b**	24	65:25
3	**4c**	31	63:37
4	**4d**	55	70:30
5	**4e**	21	70:30
6	**4f**	12	58:42
7	**4g**	23	59:41
8	**4h**	10	53:46
9	**4l**	n.r.	-
10	**4m**	n.r.	-
11	**4n**	n.r.	-
12	**4o**	n.r.	-
13	**4p**	n.r.	-
14	**4q**	n.r.	-
15	**4r**	n.r.	-
-16	**4s**	n.r.	-
17	**4t**	n.r.	-
18	**4u**	4	n.d.

[a]Reaction conditions: 0.1 mmol of **5a** (1 equiv.), 2-methylbenzophenone **1a** (5 equiv.), catalyst **4** (20 mol%) in 1 mL of MeOH was irradiated at 365 nm for 3h. [b]Determined by ^{1}H NMR analysis using pyrazine as internal standard. [c]Determined by chiral HPLC analysis.

The reaction was tested in other alcoholic solvents without significant improvement.

Table S-10. Screening of solvents[a]

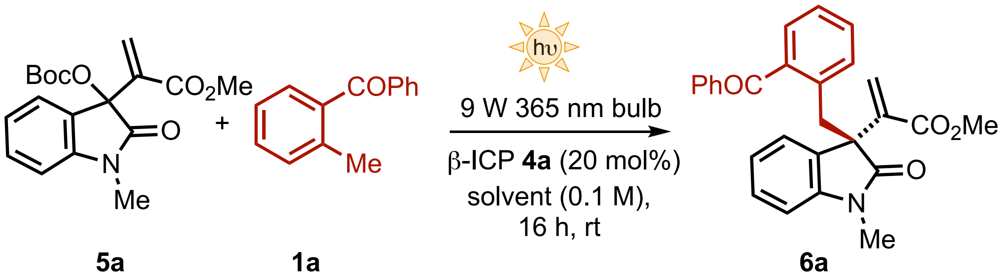

5a **1a** **6a**

entry	solvent	yield (%) of **6a**[b]	*er* of **6a**[c]
1	MeOH	66	70:30
2	*i*-PrOH	31	70:30
3	*t*-BuOH	45	71:29
4	HFIP	n.r.	n.d.

[a]Reaction conditions: 0.1 mmol of **5a** (1 equiv.), 2-methylbenzophenone **1a** (5 equiv.), β-isocupreidine **4a** (20 mol%) in 1 mL of MeOH was irradiated at 365 nm for 3h. [b]Determined by ^{1}H NMR analysis using pyrazine as internal standard. [c]Determined by chiral HPLC analysis.

Table S-11. Screening of other reaction parameters

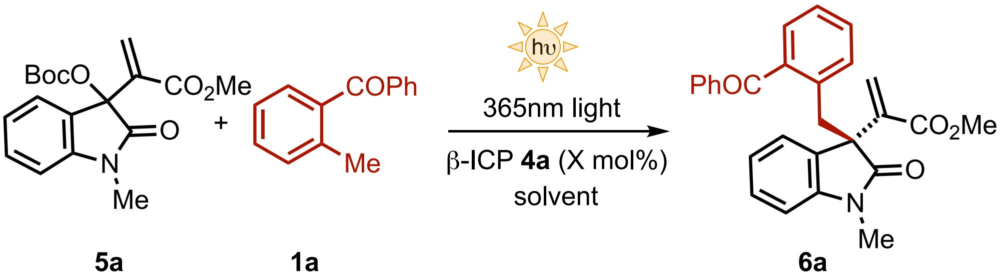

5a **1a** **6a**

entry	**4a** (mol%)	solvent	light	time (h)	yield (%) of **3**[b]	*er* of **3**[c]
1	20	MeOH	9W 365nm bulb	16	66	70:30
2	20	MeOH	HP LED @ 50 mA	16	65	70:30
3[d]	20	MeOH	HP LED @ 50 mA	16	42	72:28
4[e]	20	MeOH	HP LED @ 50 mA	16	30	75:25
5	20	MeOH	HP LED @ 350 mA	4	81	70:30
6	20	MeOH/Tol 9:1	HP LED @ 350 mA	4	70	70:30
7	20	MeOH/Chx 9:1	HP LED @ 350 mA	4	65	71:29
8	20	MeOH/DCM 9:1	HP LED @ 350 mA	4	71	71:29
9	20	MeOH/THF 9:1	HP LED @ 350 mA	4	60	70:30
10	10	MeOH	HP LED @ 350 mA	3	80 (78)	70:30
11	5	MeOH	HP LED @ 350 mA	3	45	70:30
12	1	MeOH	HP LED @ 350 mA	3	12	n.d.
13[f]	20	MeOH	HP LED @ 350 mA	3	51	70:30

[a]Reaction conditions: 0.1 mmol of **5a** (1 equiv.), 2-methylbenzophenone **1a** (5 equiv.) and catalyst **4** (x mol%) irradiated at 365 nm. [b]Determined by ^{1}H NMR analysis using pyrazine as internal standard, isolated yields are provided in parenthesis. [c]Determined by chiral HPLC analysis. [d] Reaction performed at 0ºC. [e]Reaction performed at -12ºC. [f] Using 2 equiv. of **1a**

Table S-12. Screening of aliphatic ester MBH carbonates

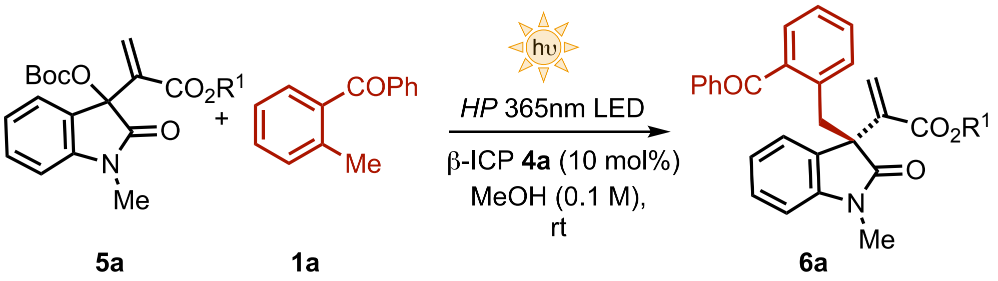

entry	R	yield (%) of **6**[b]	*er* of **6**[c]
1	Me (**5a**)	80 (78)	70:30
2	Et (**5e**)	61 (57)	70:30
3	Hex (**5m**)	33	64:36
4	*t*-Bu (**5j**)	5	85:15
5	Bn (**5l**)	34	64:36

[a]Reaction conditions: 0.1 mmol of **5a** (1 equiv.), 2-methylbenzophenone **1a** (5 equiv.) and catalyst **4** (x mol%) irradiated at 365 nm. [b]Determined by ^{1}H NMR analysis using pyrazine as internal standard, isolated yields are provided in parenthesis. [c]Determined by chiral HPLC analysis.

Results show no improvement on the enantiomeric ratios between the different aliphatic acrylates (apart from *t*-Bu, which gave only traces of product), in return yield decreases with the increasing of steric hindrance of the ester, as observed also for the linear substrate.

D.3. Optimization in microfluidic conditions

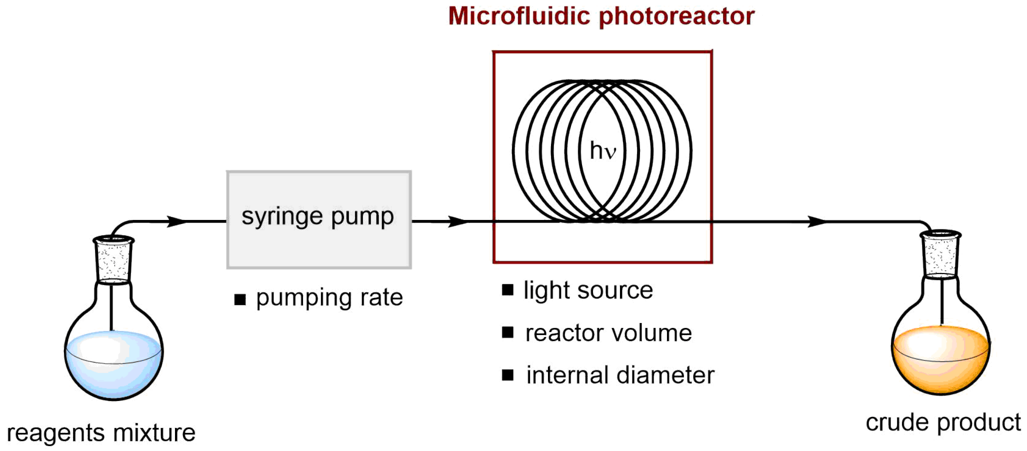

Figure S-11. General representation of a microfluidic photoreactor

Figure S-11 shows a schematic representation of the microfluidic photoreactor (MFP) and Figure S-12 shows a picture of the MFP setup used in this work . The solution containing 2-methyl benzophenone **1a,** the MBH carbonate **5a** and 10 mol% of β-ICP **4a** in MeOH (0.05 M) was properly degassed by bubbling argon for 15 min. Subsequently, the solution under argon atmosphere was introduced in continuous flow into the photoreactor via the syringe pump (Syrris Asia, see general information). The microfluidic photoreactor consists of a transparent PTFE capillary (Supelco; internal diameter: 750 μm; inner volume: 400 μL); a 9 W 365 nm bulb lamp (Figure S-4). Aluminum foil was used to avoid undesired irradiation of the tubing. To maintain a stable reaction temperature, two fans were placed in close proximity to the reactor and the temperature was controlled by a thermometer (25±2 °C).

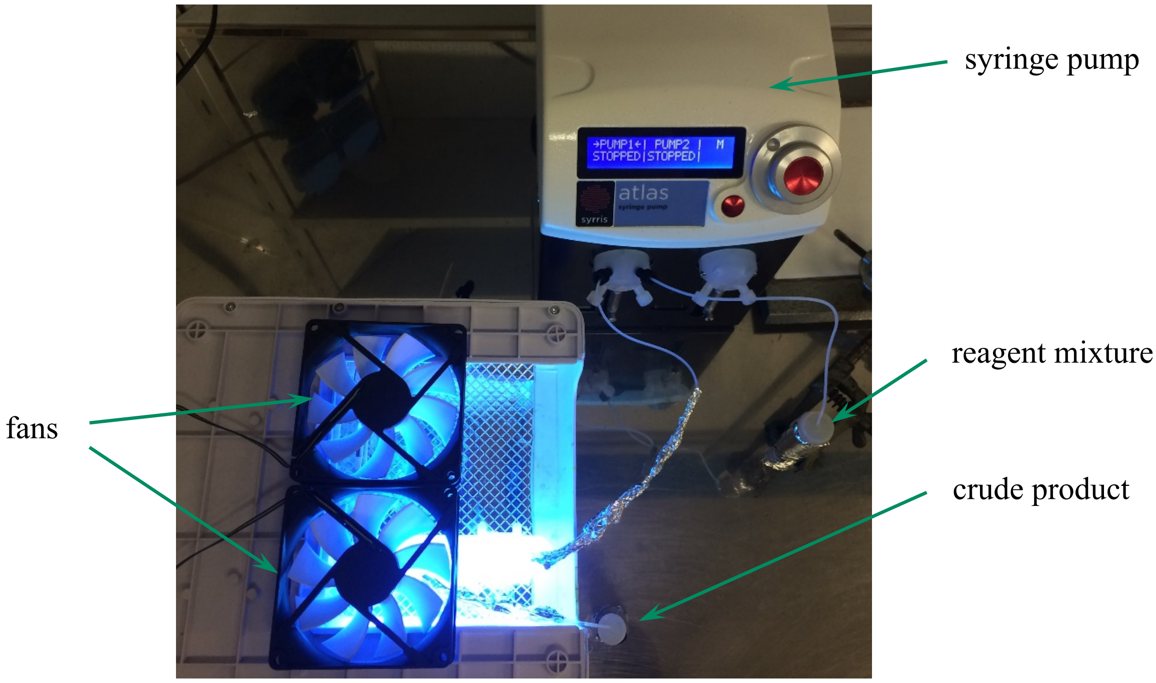

Figure S-12. Microfluidic photoreactor used in this work

To optimize the reaction in microfluidic conditions, solutions containing the starting materials and the catalyst in different concentrations were prepared and reacted in the microfluidic photoreactor at different flow rates. Subsequently, 100µl of product solution was introduced into an NMR tube, diluted with $CDCl_3$ and analyzed by ^1H NMR using pyrazine as internal standard to determine the yield of product **6a**.

Table S-13. Optimization of the asymmetric allylic benzylation of **5a** in microfluidic flow conditions.

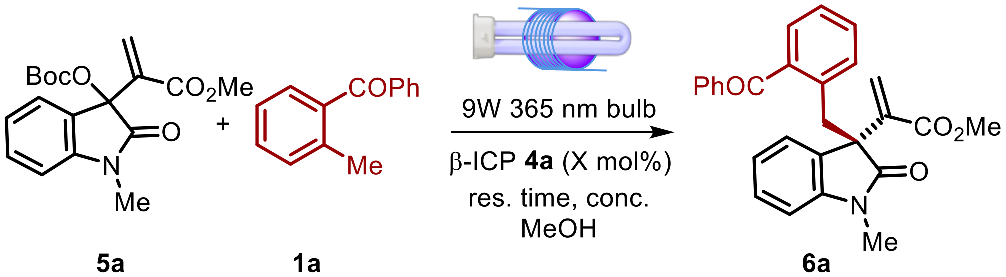

5a **1a** **6a**

entry	[**5a**] (M)	**4a** (mol %)	**1a** (equiv.)	res. time	yield of **6a**[b]
1	0.1	20	5	60 min	39%
2	0.1	20	5	40 min	52%
3	0.1	20	5	30 min	50%
4	0.05	20	5	40 min	47%
5	0.05	20	5	30 min	55%
6	0.05	20	5	15 min	46%
7	0.05	20	5	10 min	34%
8	0.05	10	5	50 min	45%
9	0.05	10	5	40 min	56%
10	0.05	10	5	30 min	69% [68%]
11	0.05	10	3	40 min	46%
12	0.05	10	3	35 min	53%

13	0.05	10	3	30 min	43%
14	0.05	10	3	20 min	37%

[a]Reaction conditions: 1 equiv. of **5a**, 2-methylbenzophenone **1a**, β-isocupreidine in methanol irradiated at 365 nm. In all the cases, product **6a** is formed in 70:30 *e.r.*. [b]Determined by ^{1}H NMR analysis using pyrazine as internal standard. Isolated yield between brackets.

Large-scale synthesis of the benzylated product **6a** was performed using the optimal conditions (entry 10). To an oven dried round-bottomed flask, the MBH carbonate **5** (1 equiv.) and β-isocupreidine **4a** (10 mol%) were added under argon atmosphere. The solids were dissolved in argon-degassed anhydrous MeOH (0.05M) and 2-methylbenzophenone **1a** (5 equiv.) was subsequently added in one portion. After further degassing with argon for 30 min, the solution was reacted using two parallel MFP setups with a flow rate of 13.3 µl/min (30 min residence time). Once all the solution was reacted, the crude product solution was treated with water and extracted three times with EtOAc. The organic layers were dried with anhydrous $MgSO_4$ and evaporated to afford the crude product, which was subsequently purified by flash chromatography to afford the pure benzylated products **6a** (1 mmol scale, 290 mg, 68% yield, 70:30 er) and **6c** (2 mmol scale, 639 mg, 72% yield, 72:28 er).

E. Isomerization studies

We decided to perform mechanistic investigations in order to understand the reasons behind the moderate asymmetric induction. Commonly, the efficient shielding of one enantiotopic face of the electrophilic double bond in the catalytic intermediate **II** is invoked as the catalytic asymmetric induction event. After extensive optimization studies, we reasoned that observed moderate enantiocontrol may be derived by a light-mediated isomerization of the catalytic intermediate **II,** where the catalyst would shield the opposite enantiotopic face and thus leading to the moderate enantiomeric ratio obtained with the large variety of catalysts tested. Therefore, the different catalytic intermediates were accurately characterized and their photoisomerization studied by means of absorbance spectroscopy and *in situ* semi-continuous ^{1}H-NMR.

E.1. Catalytic intermediate IIa derived from methyl MBH carbonate 2a and β-ICP 4a

The catalytic intermediate **IIa** was prepared by mixing equimolar amount of methyl MBH carbonate **2a** (0.05 mmol, 1 equiv.) and β-ICD **4a** (0.05 mmol, 1 equiv.) in MeOH_d$_3$ (0.5 mL, 0.1M) inside an NMR tube. The characterization confirmed the formation of the *E*-diastereoisomer.

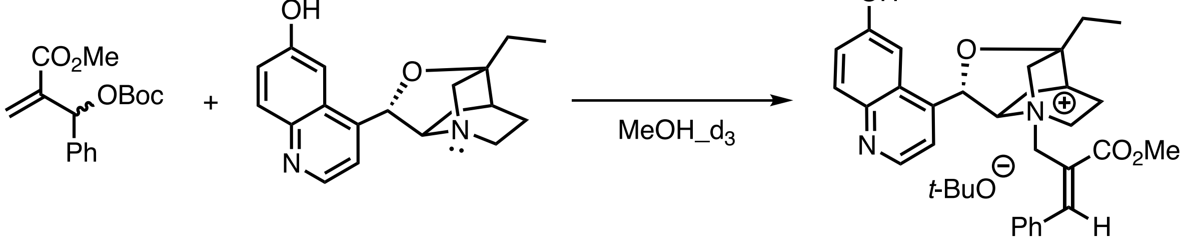

2a **4a** (*E*)-**IIa**

Figure S-13. In situ formation of the catalytic intermediate (*E*)-**IIa**

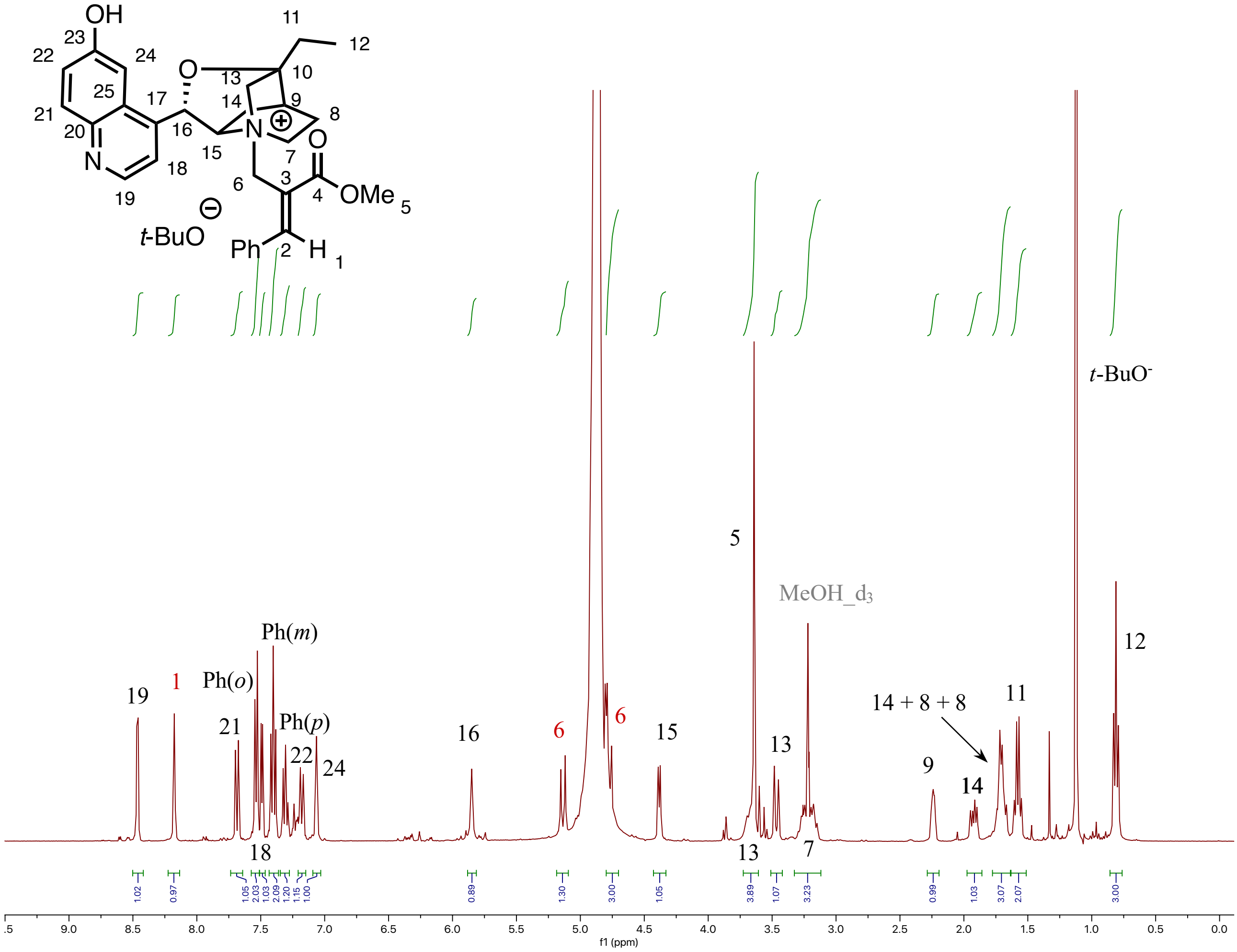

Figure S-14. ^{1}H NMR of the catalytic intermediate (*E*)-**IIa**

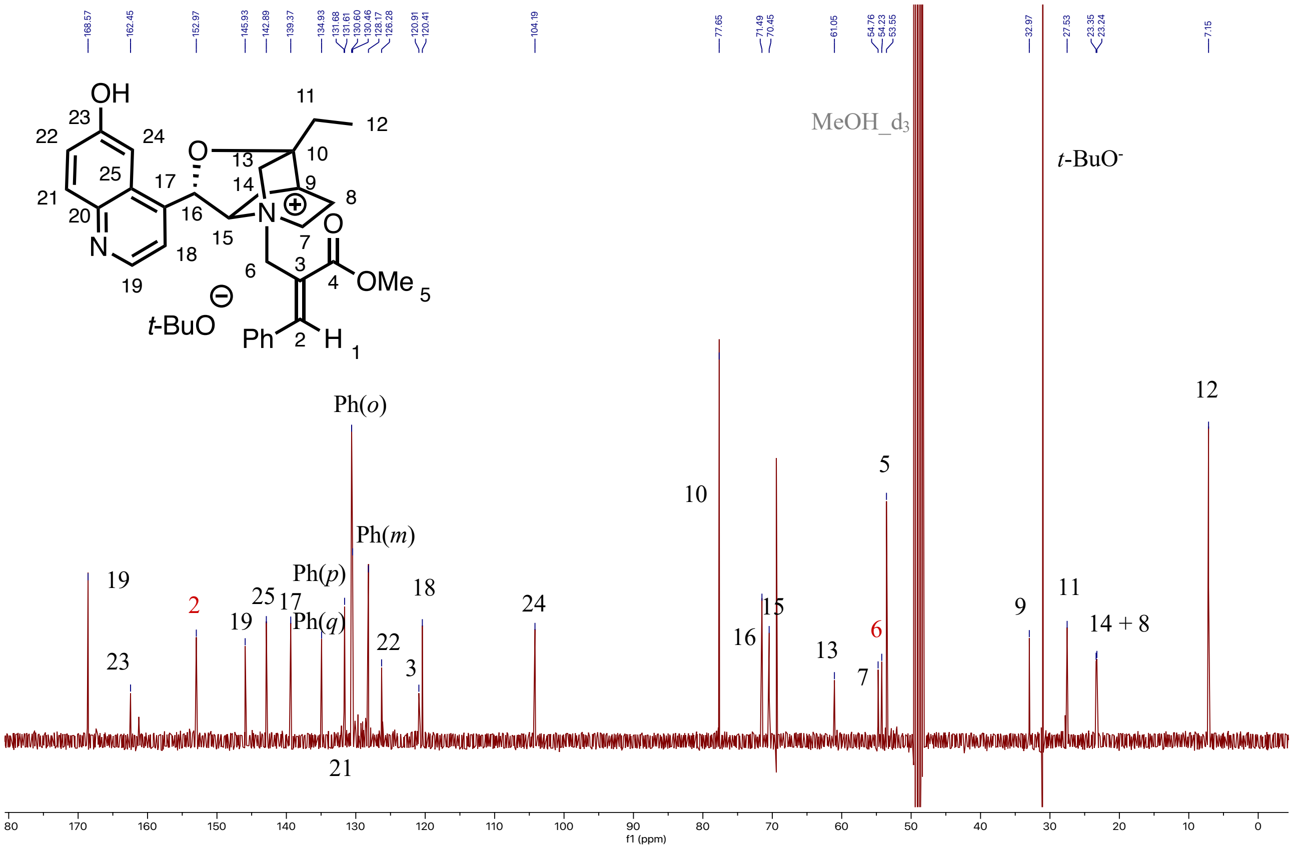

Figure S-15. 1H NMR of the catalytic intermediate (I)-**IIa**

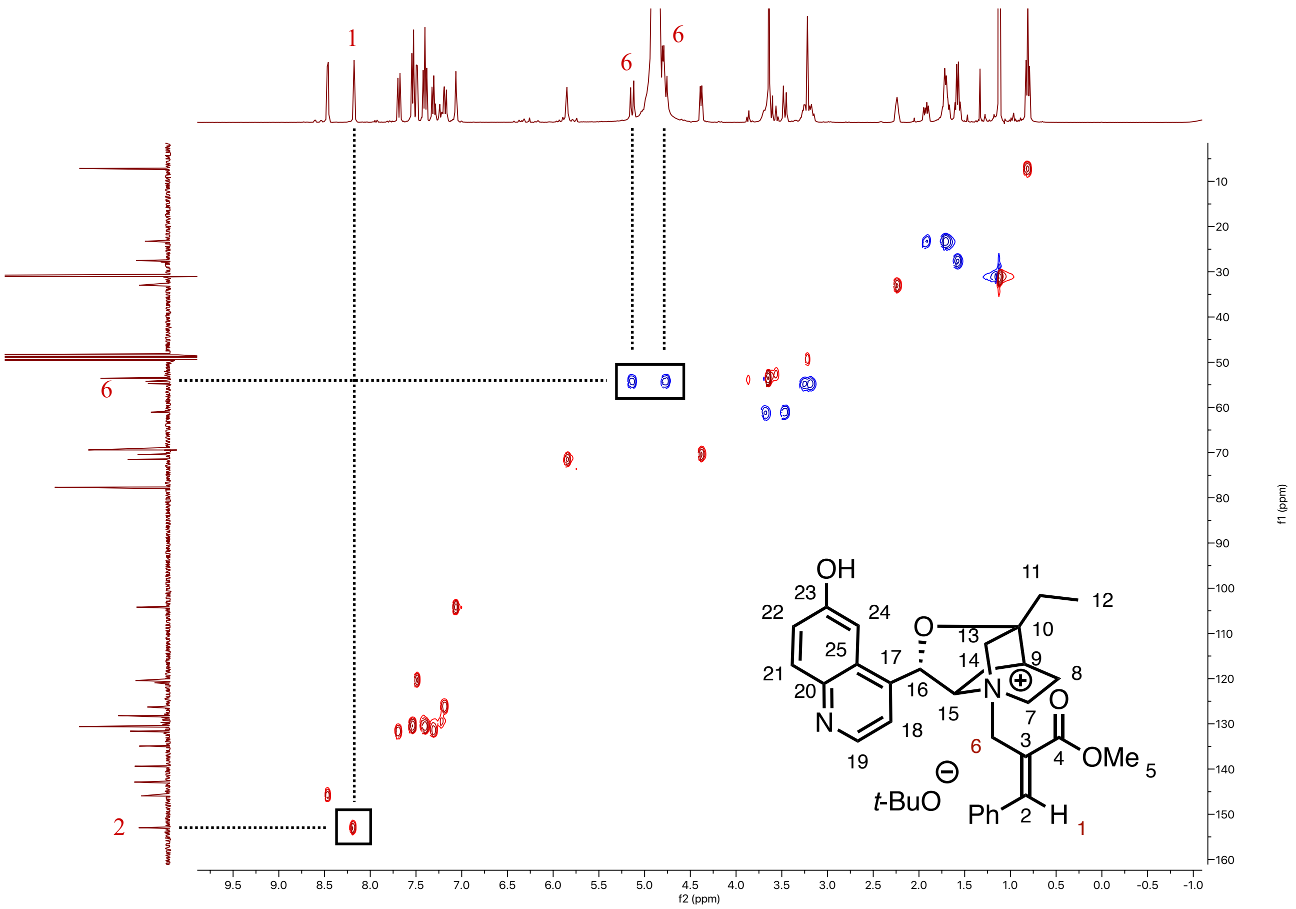

Figure S-16. HSQC of catalytic intermediate (*E*)-**IIa**

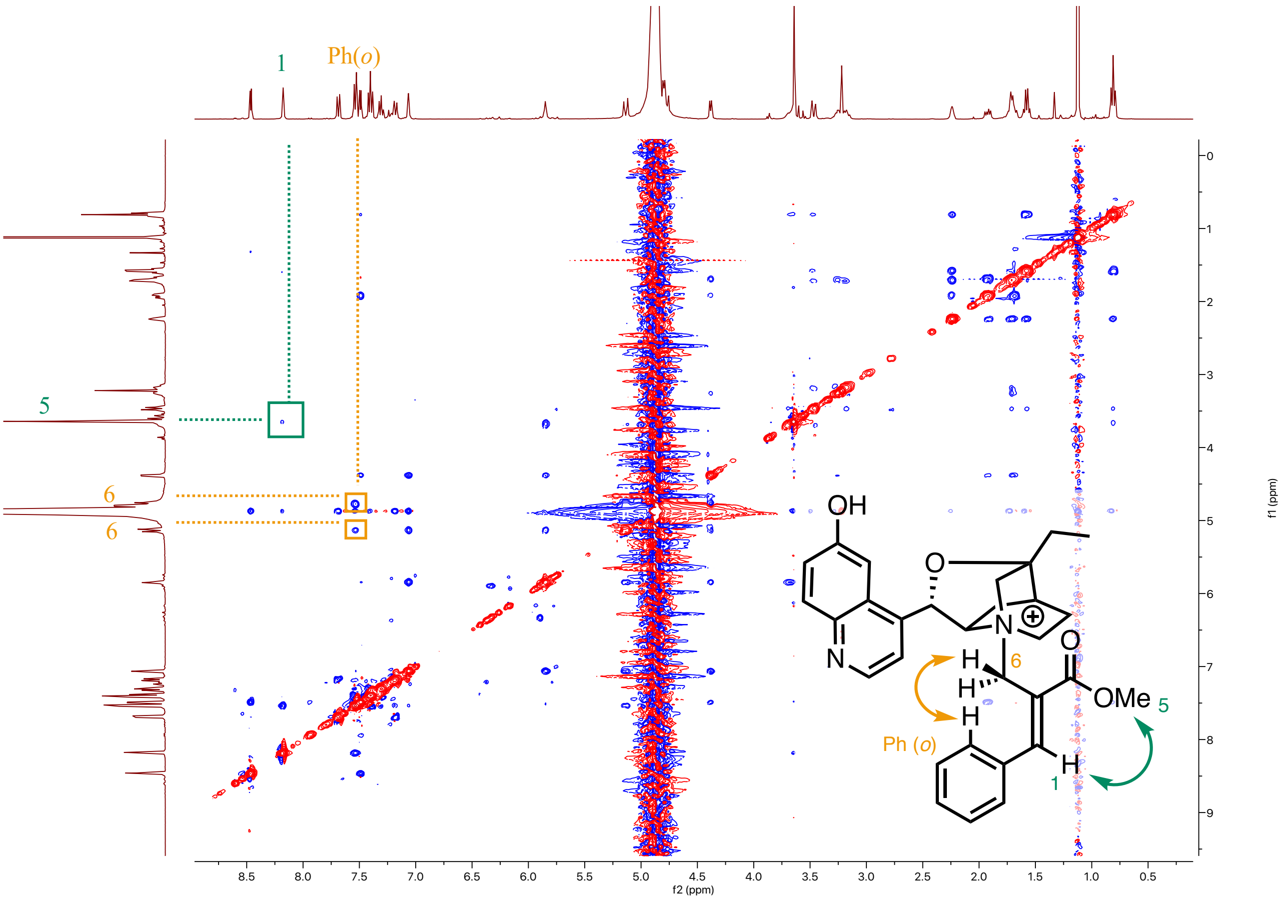

Figure S- 17. NOESY of the catalytic species (*E*)-**IIa**

Figure S-18 shows the absorbance spectrum of methyl MBH carbonate **2a**, the catalytic intermediate **IIa** and the corresponding product **3a**. As evidenced in the figure, the intermediate **IIa** starts to absorb around 440 nm. The large bathochromic shift in the absorption spectra is probably due to the conjugated nature of the system.

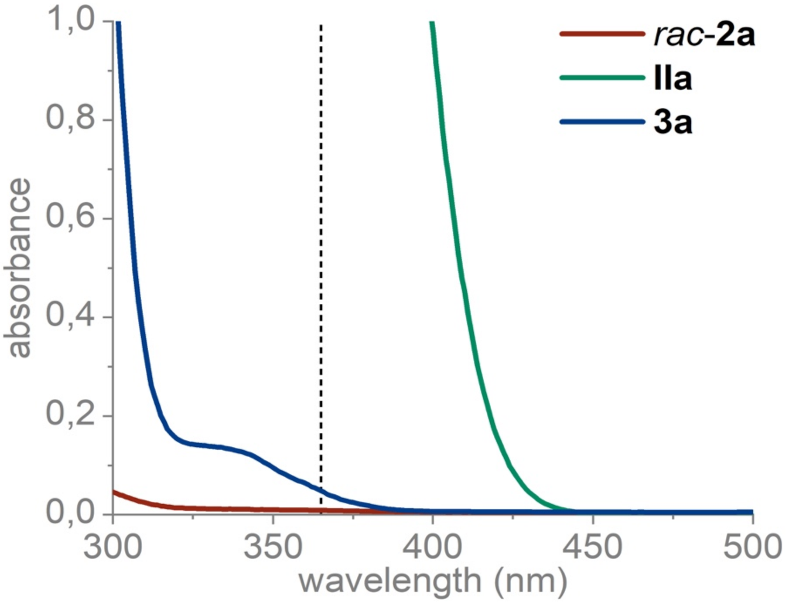

Figure S-18. Absorption spectra of methyl MBH carbonate **2a** (red line, 10^{-3}M), intermediate **IIa** (green line, 10^{-3}M) and product **3**a (blue line, 10-3M) in MeOH. The operative wavelength (365nm) is indicated with the dotted black line.

In order to study the light-mediated photoisomerization of the catalytic intermediate **IIa**, a diastereopure solution of (*E*)-**IIa** in MeOH_d3 inside an NMR tube was irradiated at 365nm with a 9W bulb. 1H NMR spectrum were acquired at regular intervals of time. As evidenced in Figure S-19 the initial *(E)* isomer converted to the corresponding *(Z)* isomer under 365nm light irradiation.

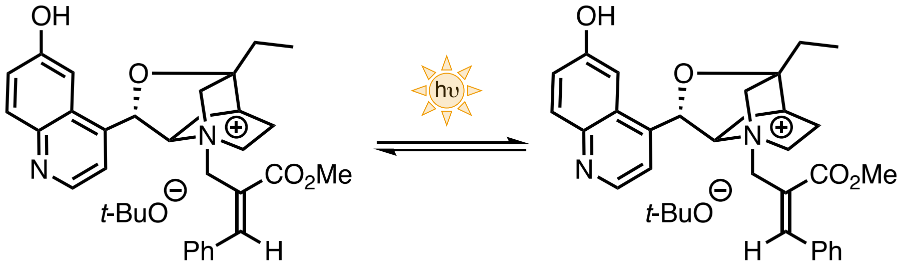

(*E*)-**IIa** (*Z*)-**IIa**

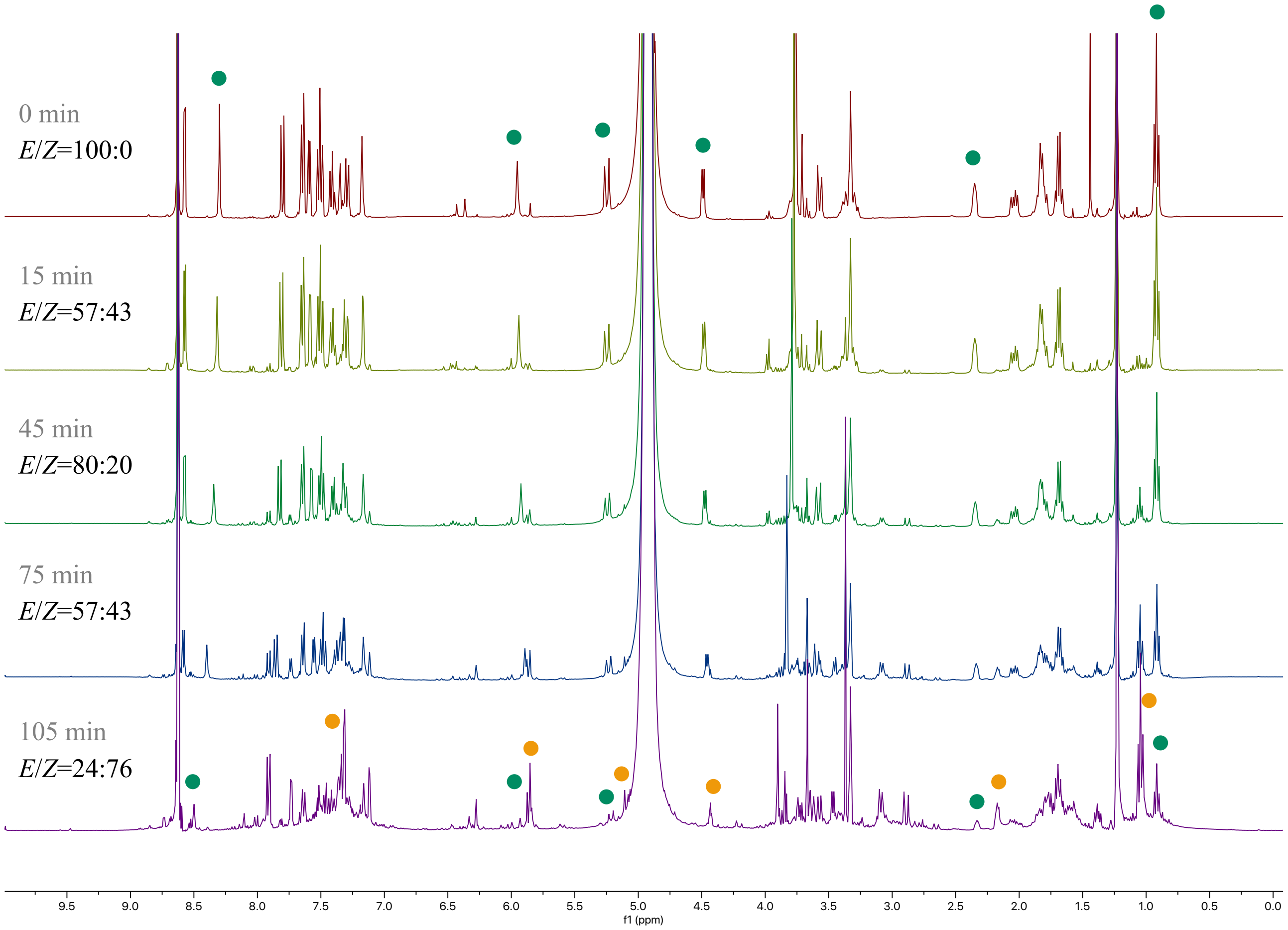

Figure S-19. Photoisomerization experiment of **IIa** followed by semi-continuous ^{1}H-NMR. The evolution of both species was quantified integrating the triplet at 0.92ppm for (*E*)-**IIa** and the at 1.05 ppm (*Z*)-**IIa**.

In order to study whether the *t*-BuO^{-} basic counteranion could influence the isomerization process, the salt **IIa'** derived from methyl MBH carbonate **2a** and DABCO bearing the non-basic bromide counteranion was synthesize as a 68:32 *E*/*Z* mixture, according the literature procedure.[2] As shown in Figure S-20, the light-triggered isomerization of the salt **IIa'** is operative at 365nm, from the starting diastereomeric mixture E/Z=68:32 to 43:57 in 140 min.

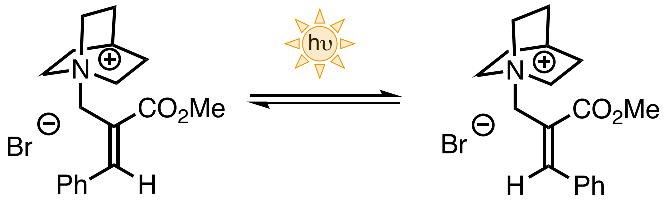

(*E*)-**IIa** (*Z*)-**IIa**

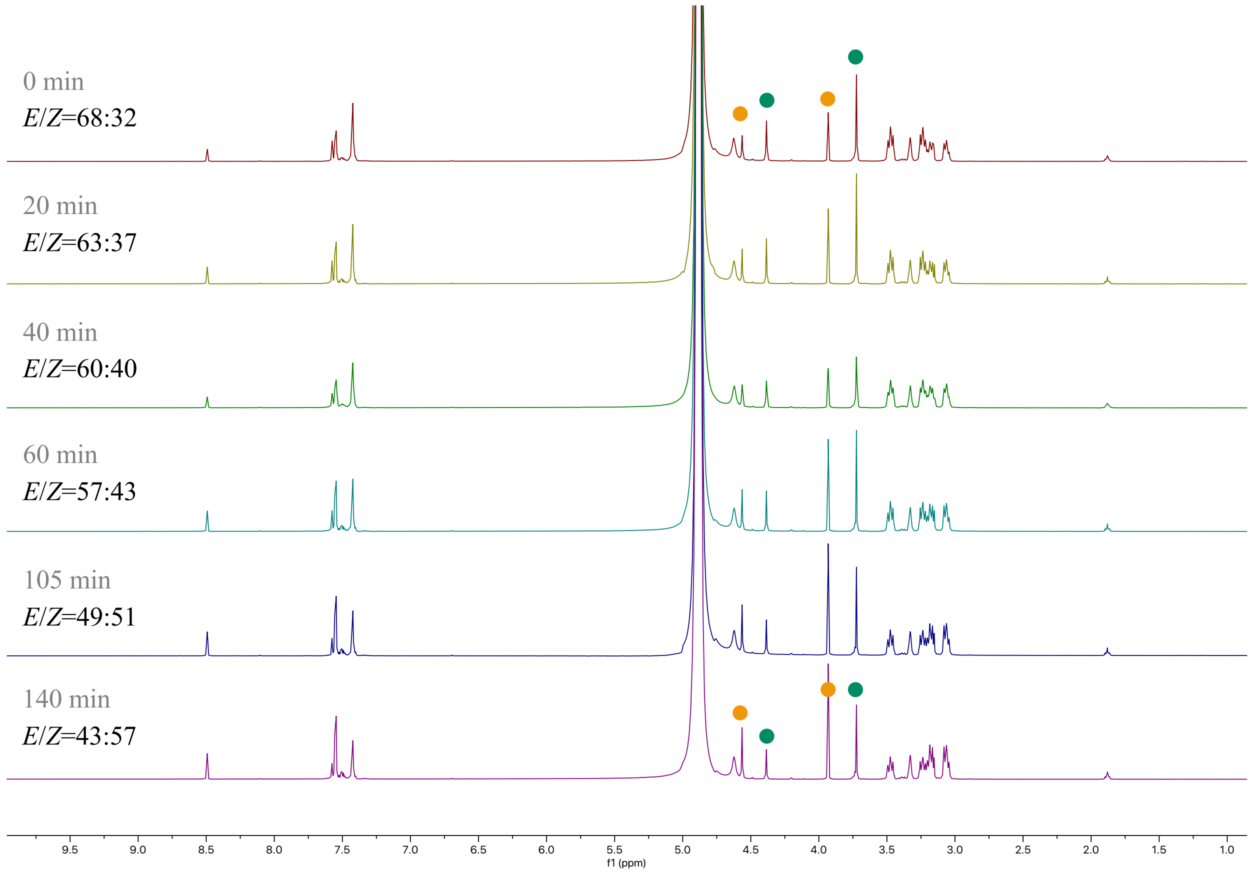

Figure S-20. Photoisomerization experiment of salt **IIa’** 1H-NMR followed by semi-continuous NMR in MeOH_d$_3$. The evolution of both species was quantified integrating the singlet at 3.72 ppm for (*E*)-**IIa’** and the at 3.93 ppm (*Z*)-**IIa’**.

E.2. Catalytic intermediate IIIg/IVg derived from 2-naphtyl MBH carbonate 2g and β-ICP 4a

The catalytic intermediate **IIIg/IVg** was prepared by mixing equimolar amount of 2-naphtyl MBH carbonate **2g** (0.05 mmol, 1 equiv.) and β-ICD **4a** (0.05 mmol, 1 equiv.) in toluene_d$_8$ (0.5 mL, 0.1M) inside an NMR tube. The characterization confirmed the formation of a diastereomeric mixture **IIIg/IVg** = 75:25

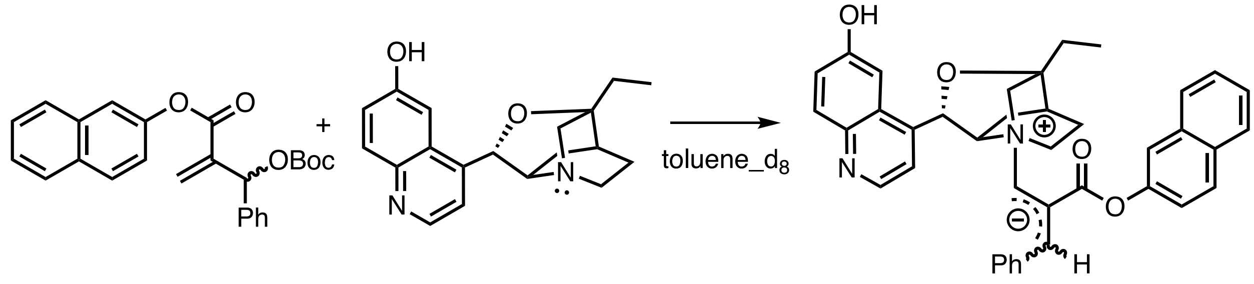

2g **4a** **IIIg/IVg**

Figure S-21. In situ formation of the catalytic intermediate **IIIg/IVg**

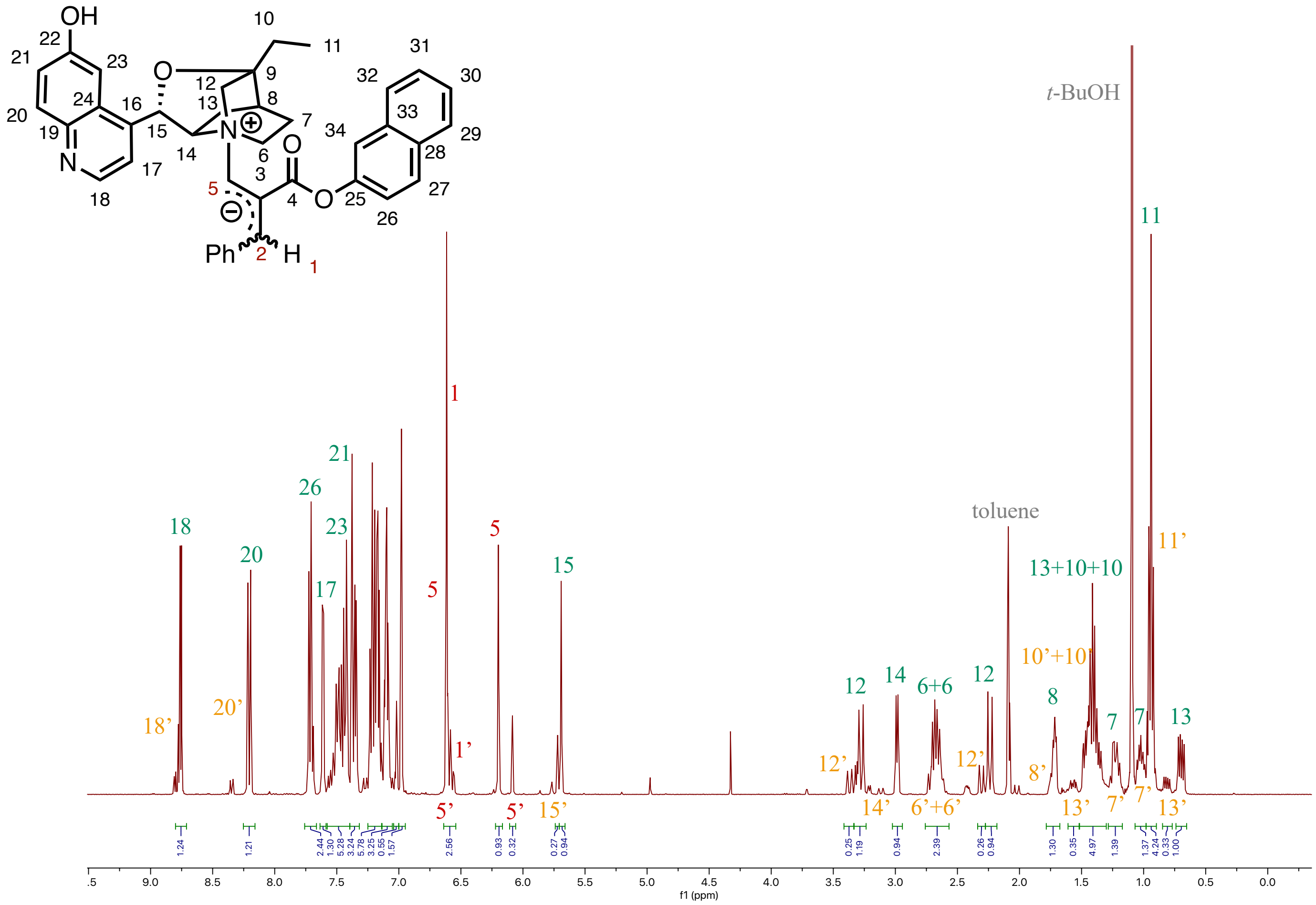

Figure S-22.^{1}H NMR of the catalytic intermediate **IIIg** (major, in green) and **IVg** (minor, in yellow).

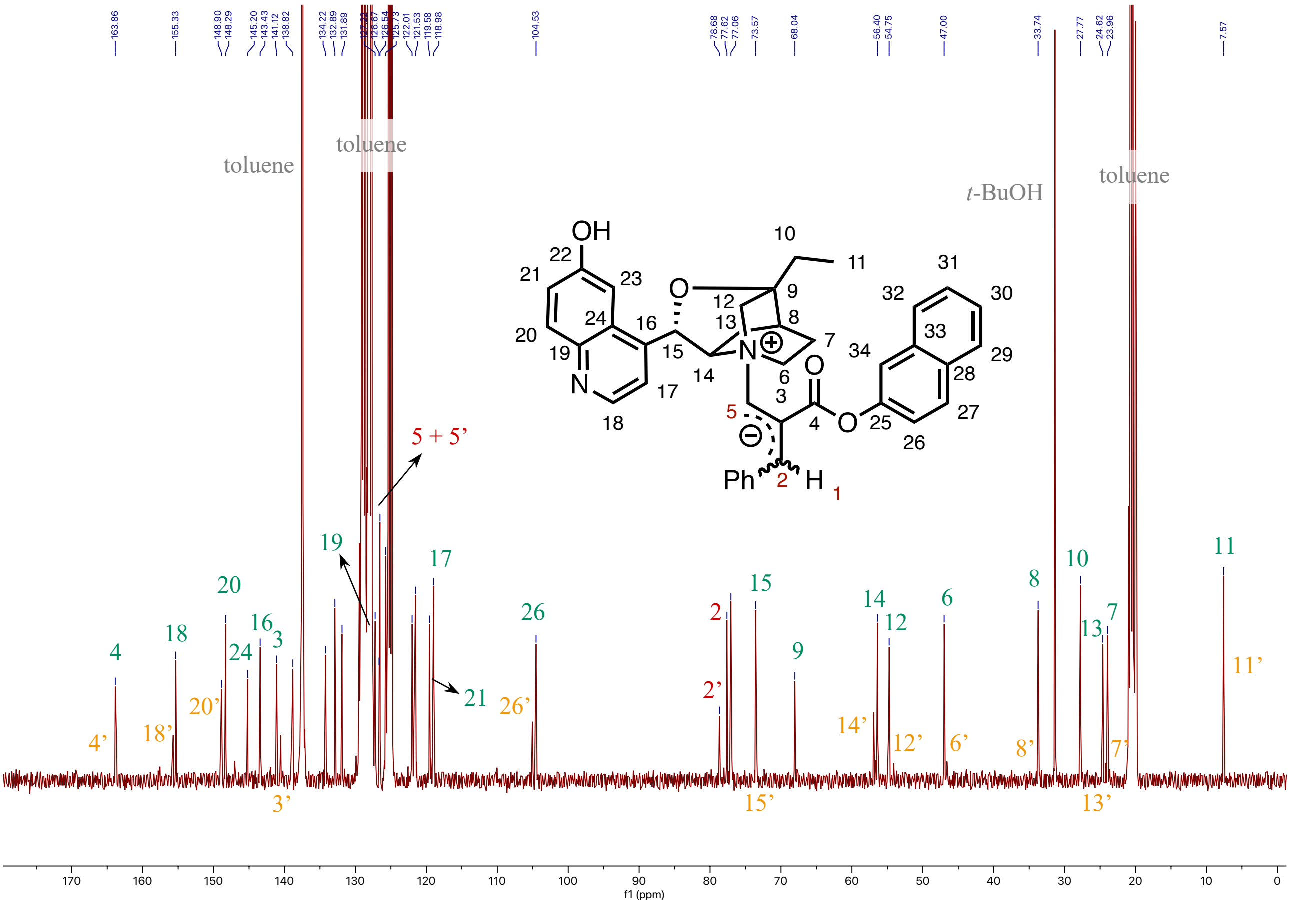

Figure S-23. ^{13}C NMR of the catalytic intermediate **IIIg** (major, in green) and **IVg** (minor, in yellow).

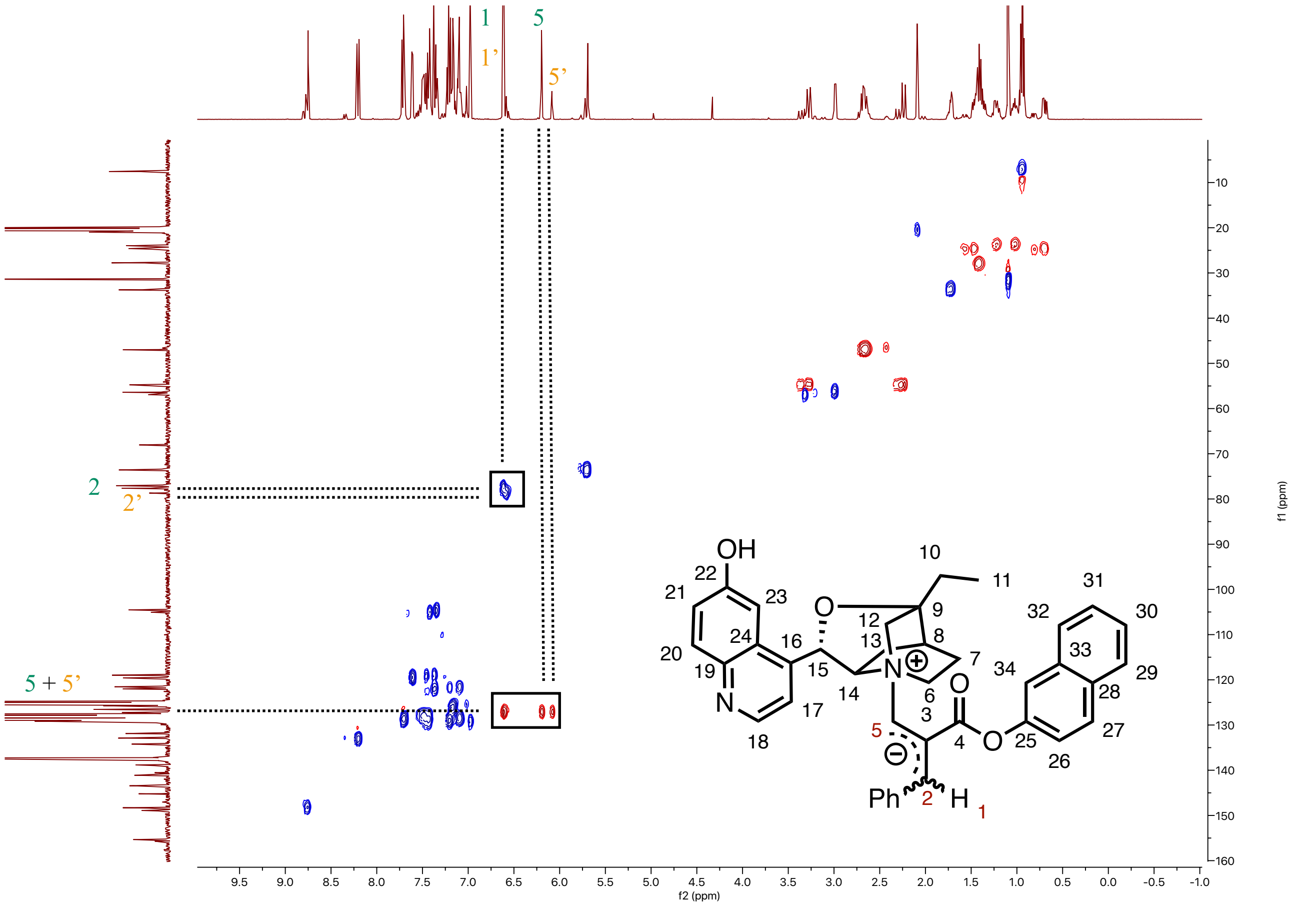

Figure S-24. HSQC of the catalytic intermediate **IIIg**/ **IVg**

The NOESY experiment of the catalytic intermediates **IIIg**/**IVg** could not be resolved.
Figure S-25 shows the absorbance spectrum of methyl MBH carbonate **2a**, the catalytic intermediate **IIa** and the corresponding product **3a**. As evidenced in the figure, the intermediate **III/IVg** starts to absorb around 380 nm.

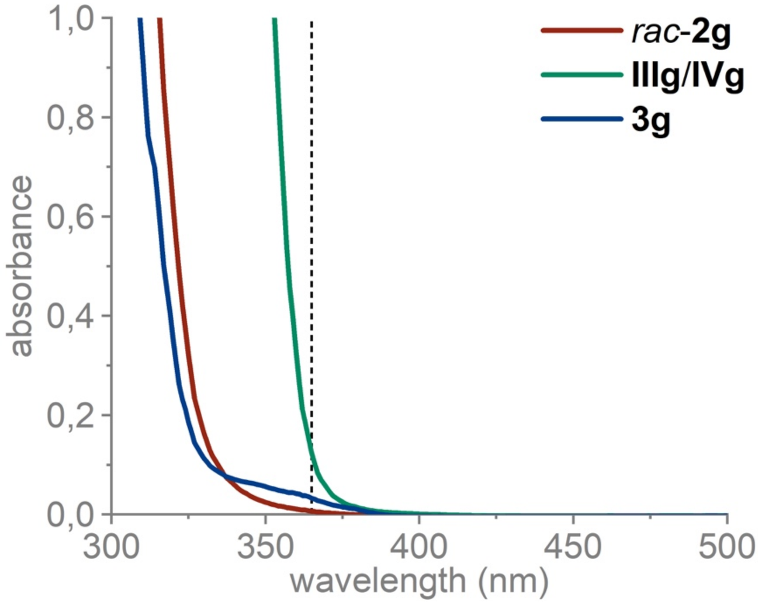

Figure S-25. Absorption spectra of 2-naphthyl MBH carbonate **2g** (red line, 10^{-3}M), intermediate **III/IVg** (green line, 10^{-3}M) and product **3a** (blue line, 10^{-3}M) in toluene. The operative wavelength used in the present work (365 nm) is shown as a dotted black line.

The change in ratio of the two diastereomers of the catalytic intermediate in this case is combination of base- and light mediated isomerization. Being toluene a non-protic solvent, it is not able to quench the strongly basic $^{t}BuO^{-}$, which is responsible for the deprotonation of α-position of the intermediate **IIg** to furnish **IIIg**. It is evidenced by sp^2 character of Cα of the intermediate which appears at 124 ppm. The scheme also explains why large bathochromic shift in absorption was absent in this case, compared with the intermediate **IIa** (see Figure S-25). The conjugate system is not present in intermediates **III/IV**.

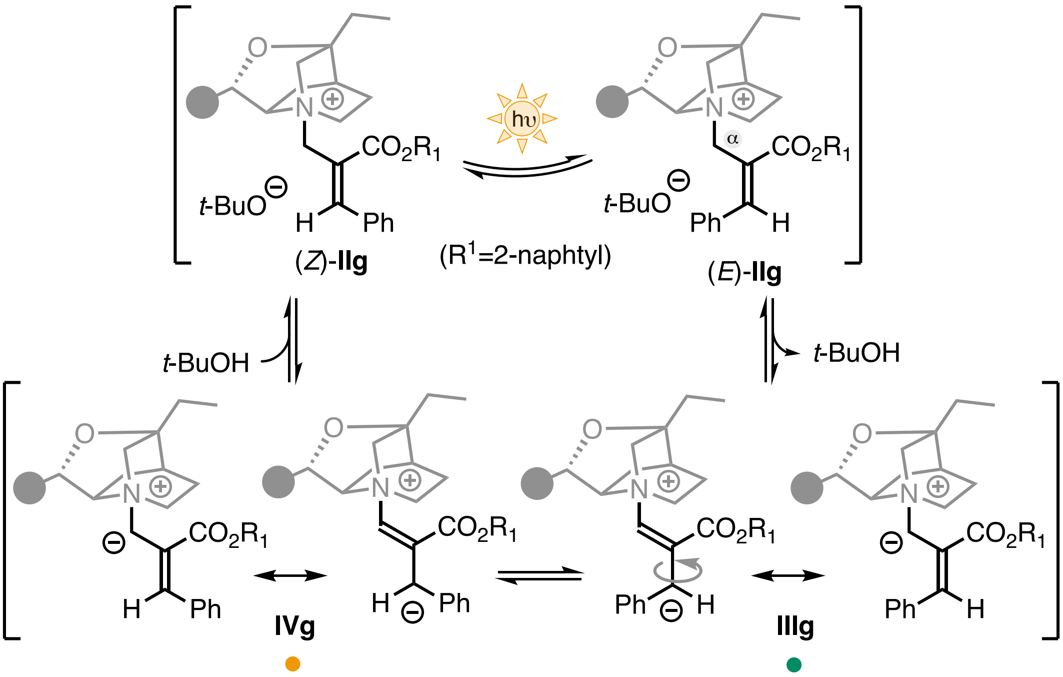

Figure S-26. Isomerization of the catalytic intermediate **III/IVg**

To study the light-mediated photoisomerization of the catalytic intermediate **III/IVg**, a solution of these intermediates in toluene_d8 formed in situ inside an NMR tube was irradiated at 365nm with a 9W bulb. ^{1}H NMR spectrum were acquired at regular intervals of time. As evidenced in Figure S-27, **IIIg** converted to **IVg** under 365nm light irradiation, albeit in much lower rate than the catalytic intermediate **IIa**.

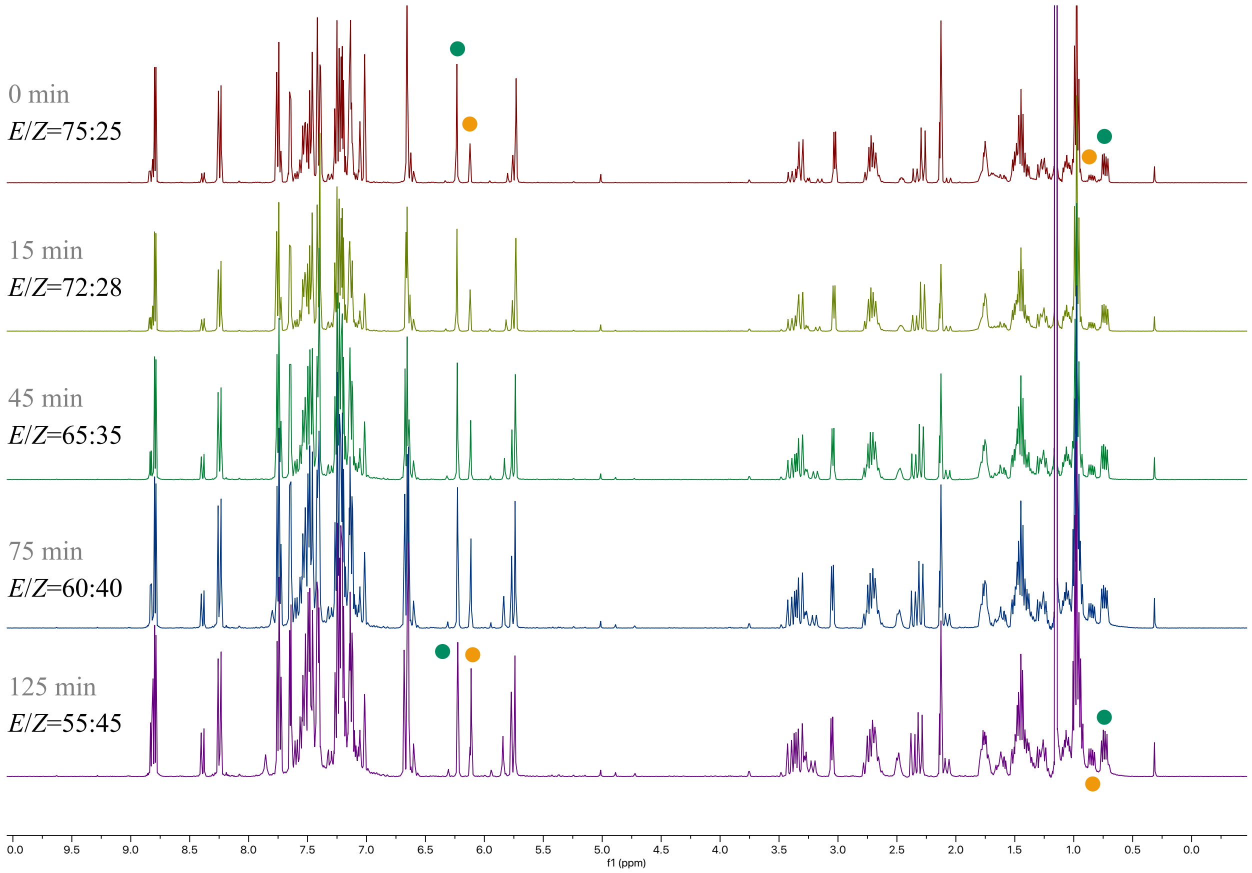

Figure S-27. Light-mediated isomerization experiment the catalytic intermediate III/IVg monitored by semi-continuous ^{1}H-NMR. The evolution of the species was quantified integrating the singlet at 6.23 ppm for **IIIg** and the at 6.12 ppm **IVg**.

E.3. Catalytic intermediate Va derived from isatin MBH carbonate 5a and β-ICP 4a

The catalytic intermediate **Va** was prepared by mixing equimolar amount of isatin-derived methyl MBH carbonate **5a** (0.05 mmol, 1 equiv.) and β-ICD **4a** (0.05 mmol, 1 equiv.) in MeOH_d3 (0.5 mL, 0.1M) inside an NMR tube. The characterization confirmed the formation of the *E*-diastereoisomer.

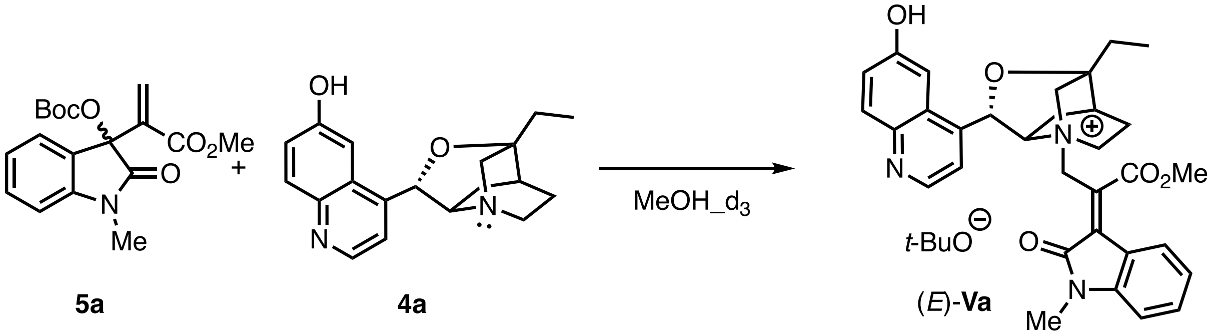

Figure S-28. In situ formation of the catalytic intermediate **Va**

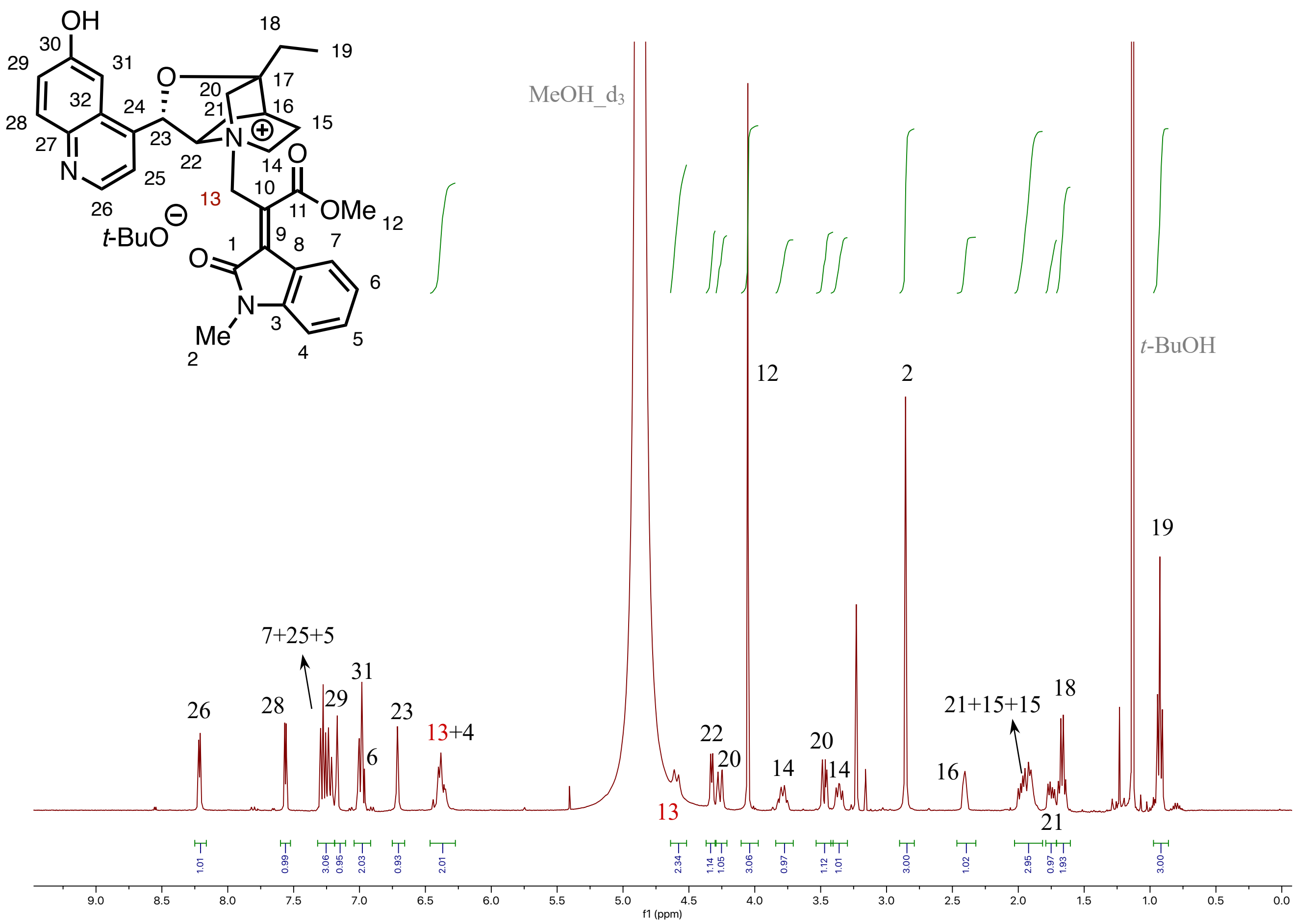

Figure S-29. ^{1}H NMR of the catalytic intermediate **V**

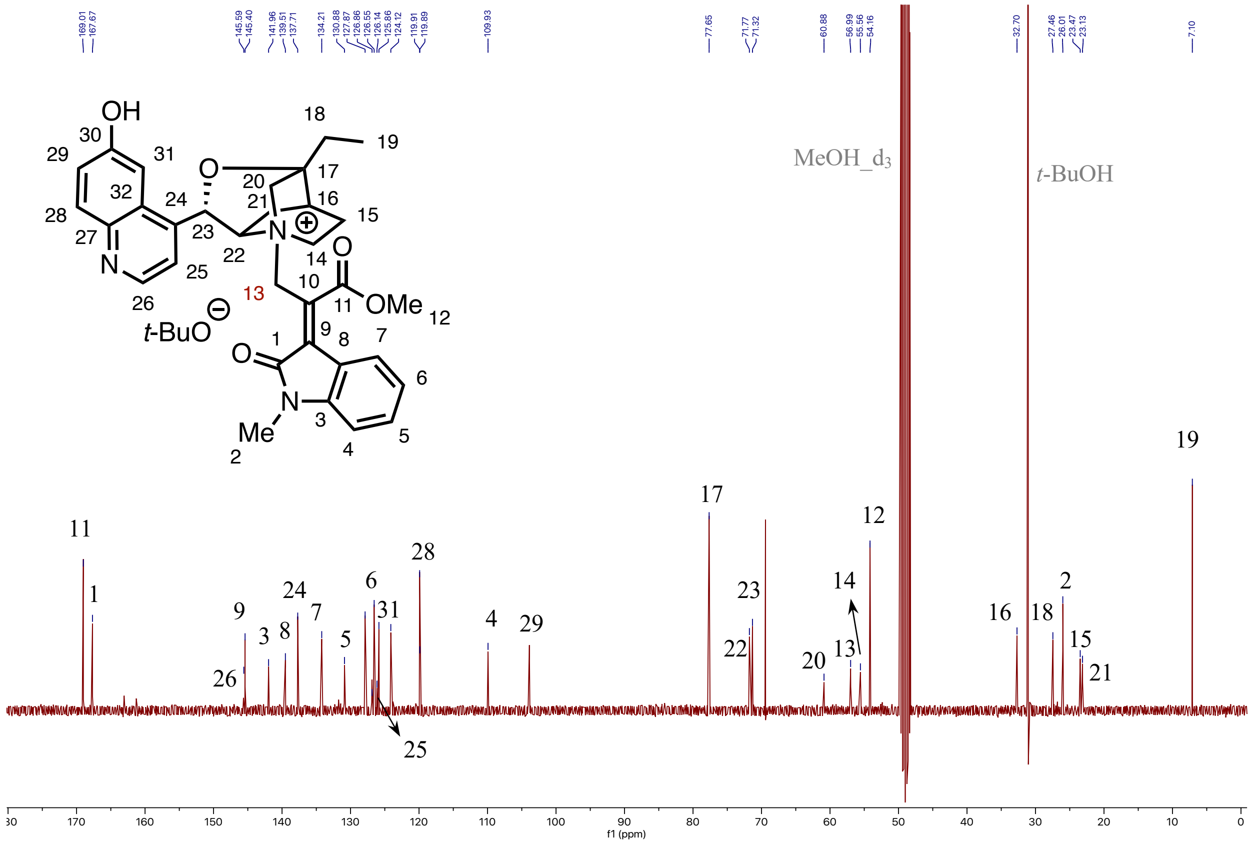

Figure S-30. ^{13}C NMR of the catalytic intermediate **Va**

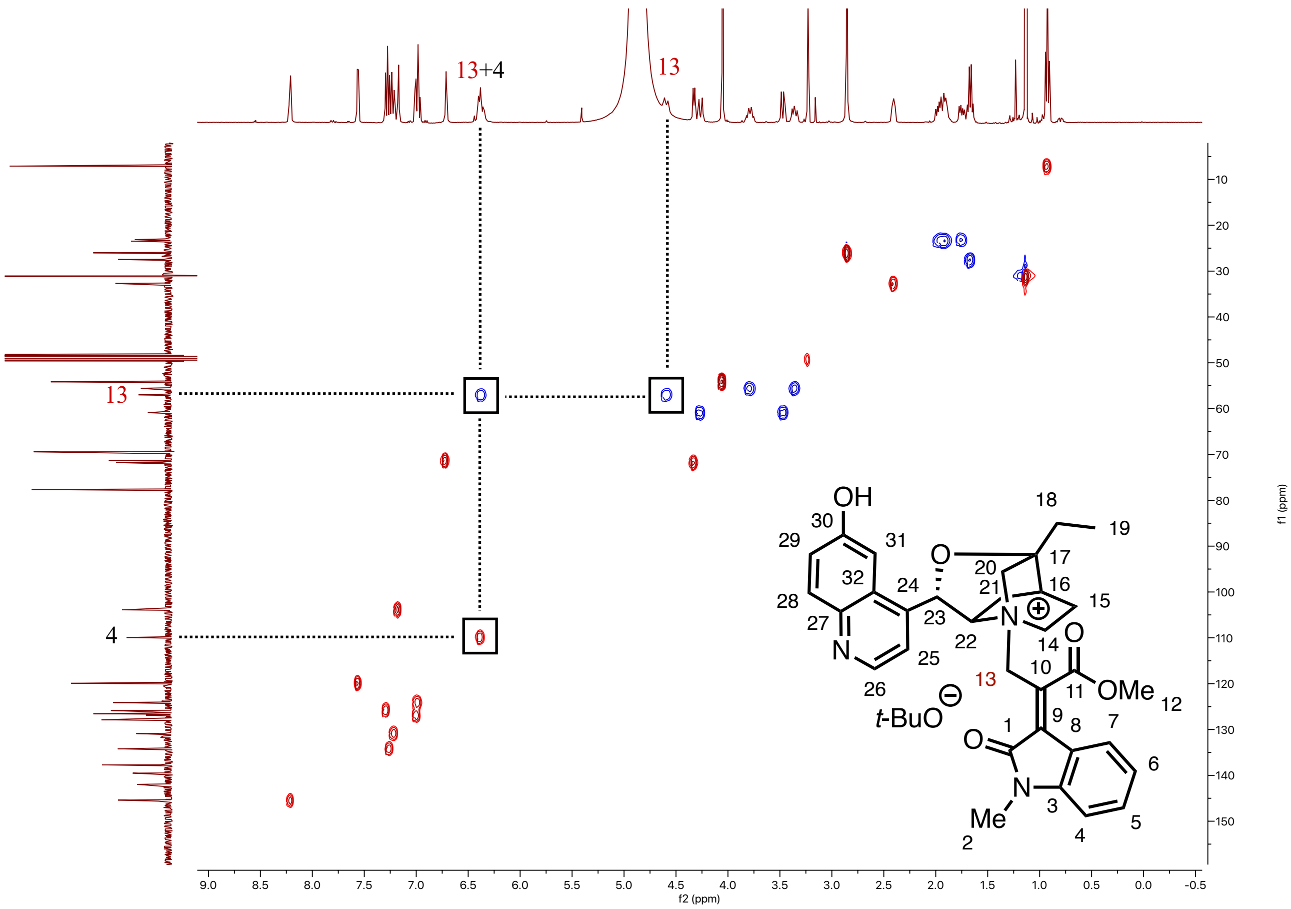

Figure S-31. HSQC of the catalytic intermediate **Va**

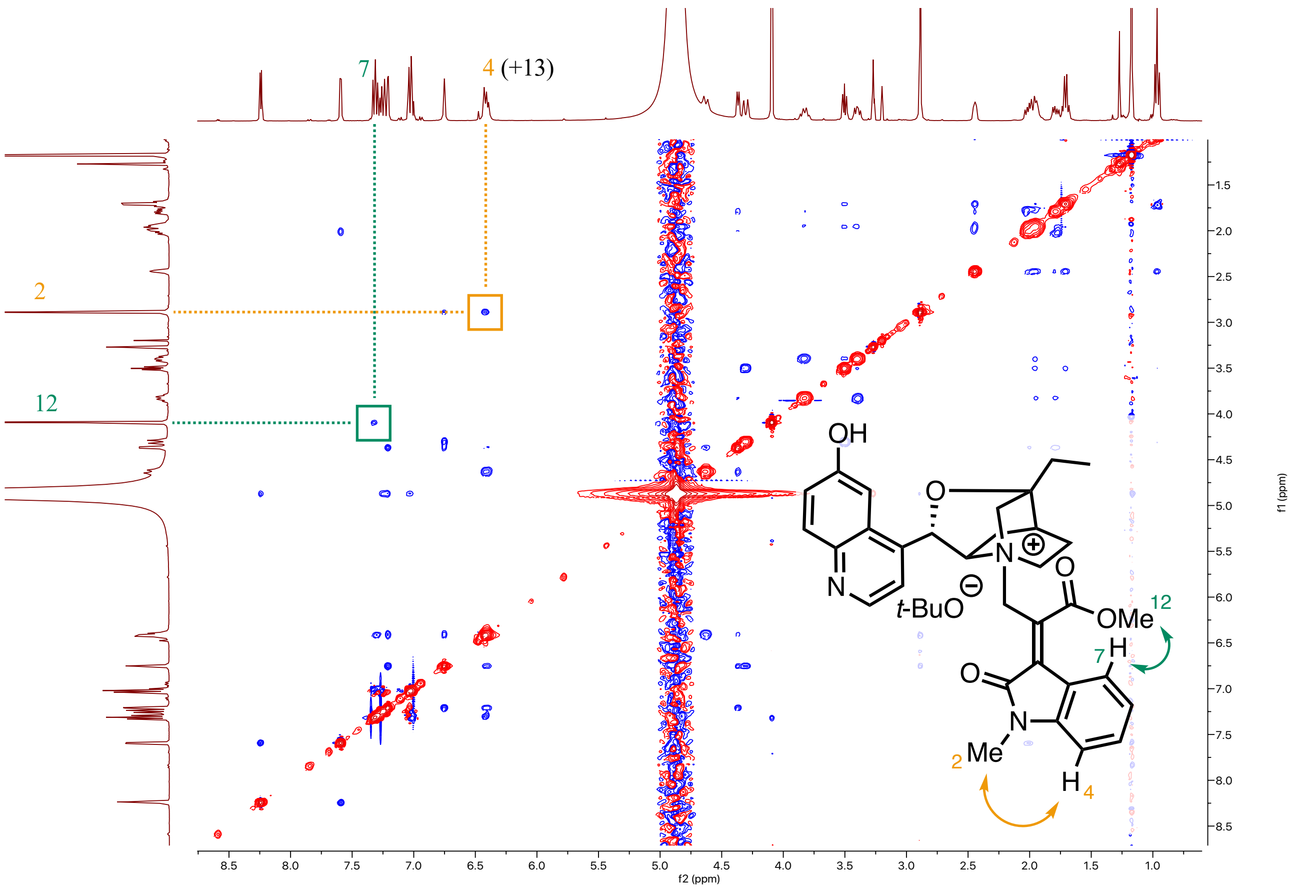

Figure S-32. NOESY of the catalytic intermediate **Va**

Although the solely catalytic intermediate detected from **5a** and catalyst **4a** is **Va**, it is in equilibrium with the corresponding allylic ylide. Complete deuteration of the C13 was observed within approximately 180 min when the **Va** was *in situ* formed with MeOH_d4, due to the high acidity of these protons.

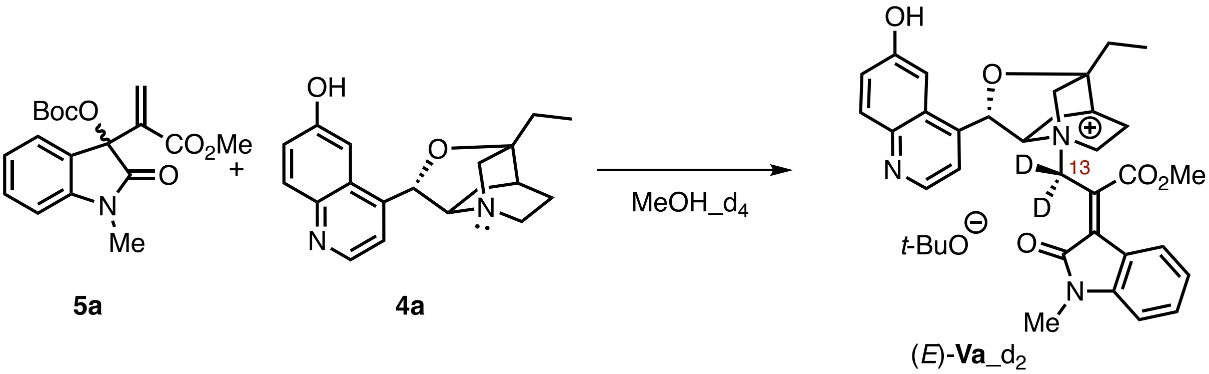

Figure S-33. In situ formation of the catalytic intermediate **Va**_d2

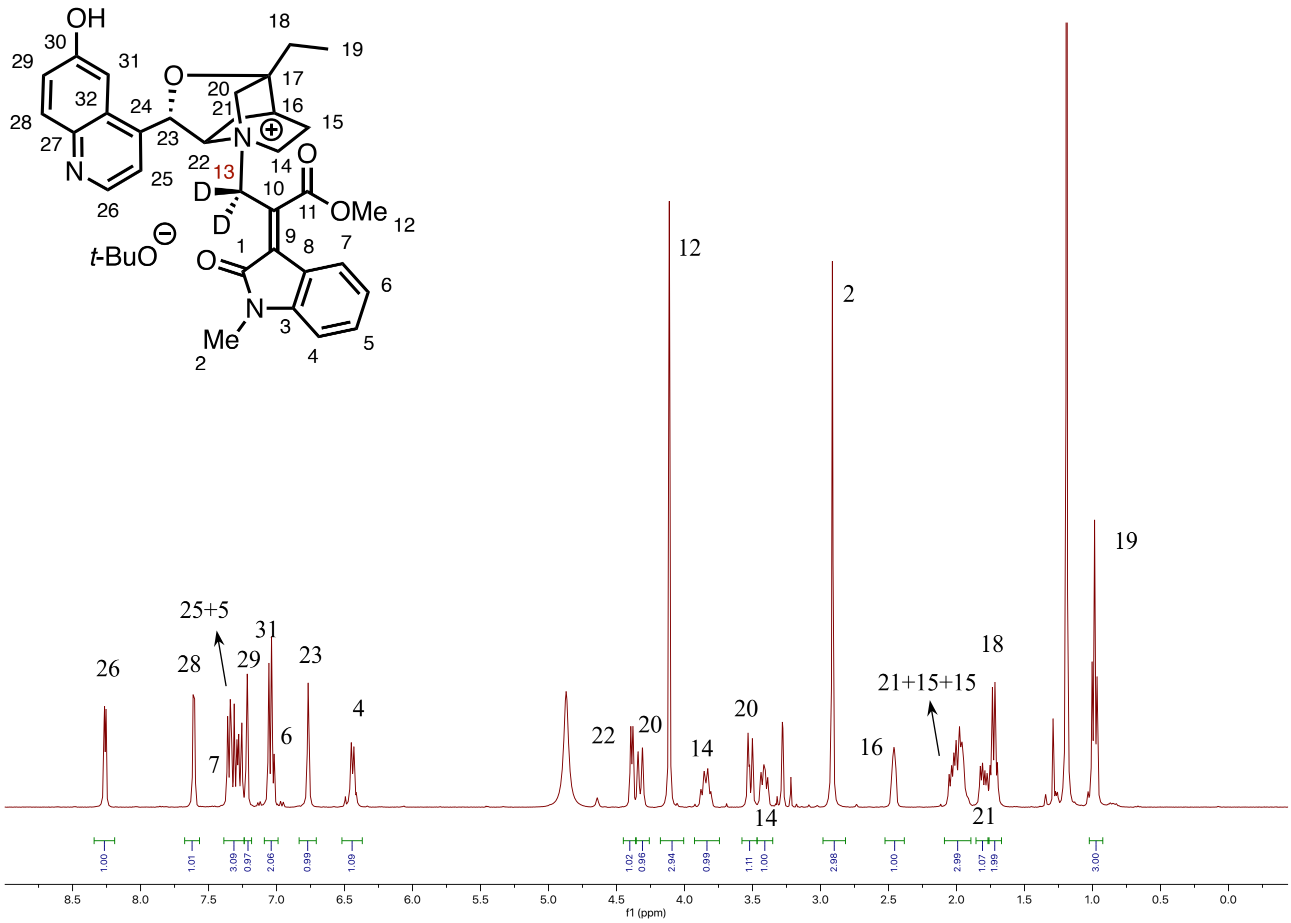

Figure S-34. ^{1}H-NMR of the catalytic intermediate **Va**_d2

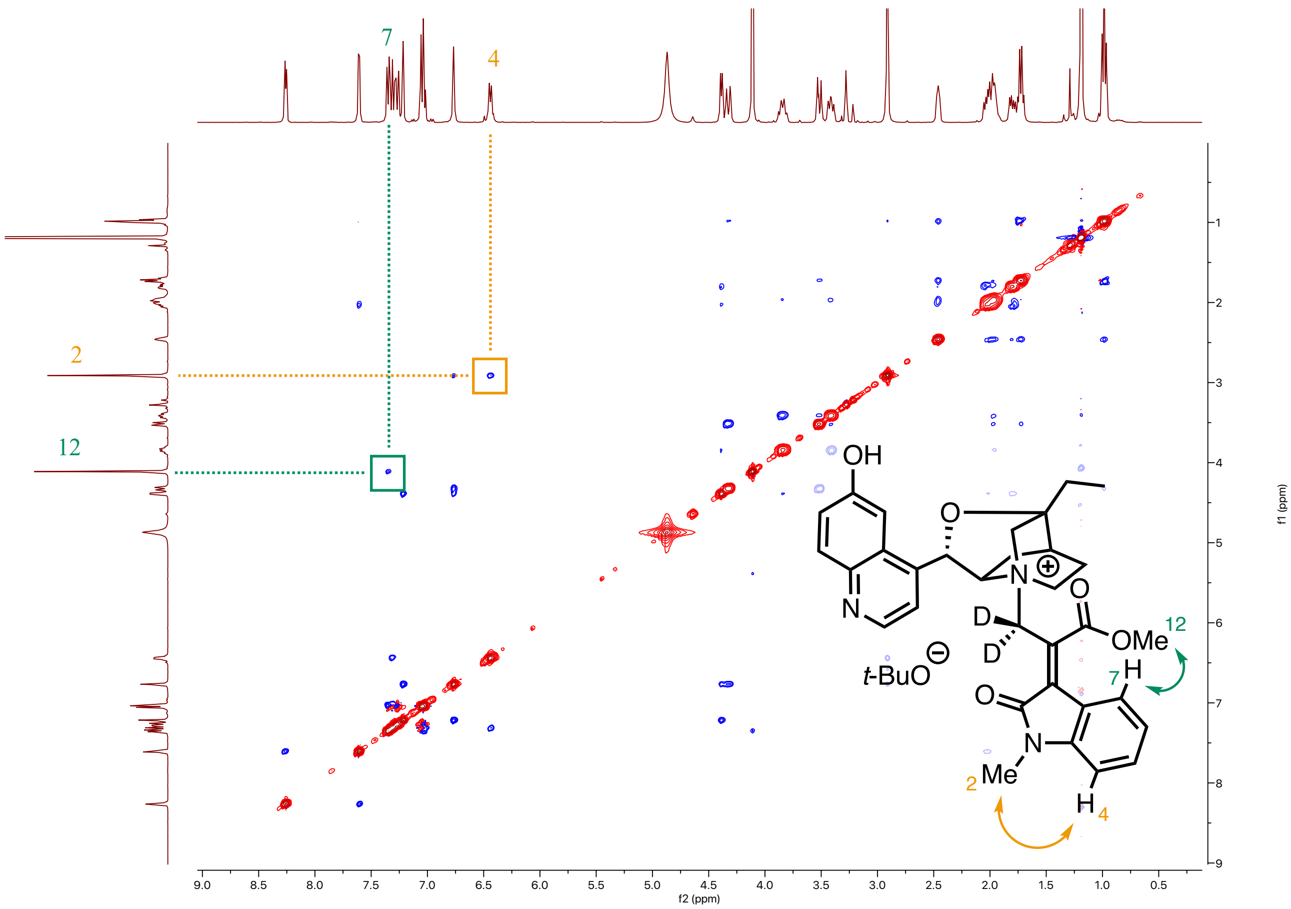

Figure S-35. NOESY of the catalytic intermediate **Va**_d₂

Figure S-36 shows the absorbance spectrum of isatin-derived methyl MBH carbonate **5a**, the catalytic intermediate (*E*)-**Va** and the corresponding product **6a**. As evidenced in the figure, the intermediate (*E*)-**Va** starts to absorb around 550 nm, and the allylic benzylated product also absorbs significantly at the operative wavelength.

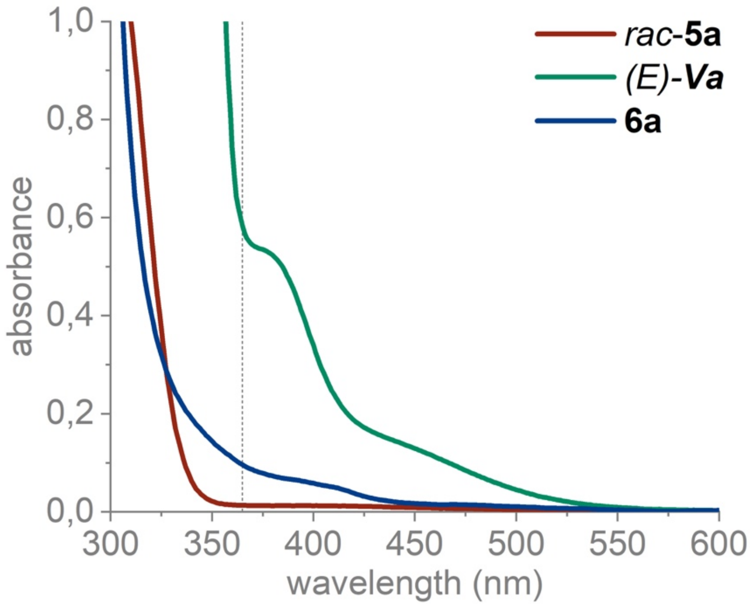

Figure S-36. Absorption spectra of isatin MBH carbonate **5a** (red line, 10^{-3}M), intermediate **Va** (green line, 10^{-3}M) and product **6a** (blue line, 10^{-3}M) in MeOH. The operative wavelength used in the present work (365 nm) is shown as a dotted black line.

To investigate the isomerization of the catalytic intermediate **Va** by semi-continuous ^{1}H-NMR experiments, a solution of **Va** (prepared by mixing MBH carbonate **5a** and β-ICD **4a** in equimolar amounts) in CD_3OH was irradiated at 365nm with the 9W bulb. Contrary to the other catalytic intermediates, **Va** quickly decomposed in the experimental conditions under UV-light irradiation and therefore the intended photoisomerization could not be quantified.
In order to study the isomerization of the isatin-derived catalytic intermediate, the bromide salt **Va'** was synthesized as shown in Figure S-37 adapting a procedure described in literature.[3]

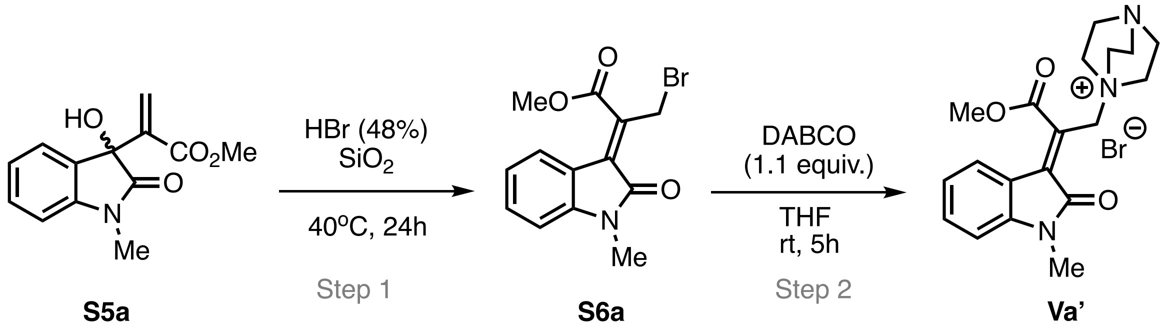

S5a **S6a** **Va'**

Figure S-37. Synthesis of the bromide salt **Va'**.

Step 1. The MBH adduct **S5a** (2 mmol) was treated with aqueous HBr 48% (1.4 mL) in the presence of silica gel (670 mg) and heated at 40 ˚C for 24h. The mixture was then filtered through a bed of Celite® and washed with CH_2Cl_2 (15 mL). The organic layer was collected, washed with brine and concentrated in vacuo to furnish the crude bromide **S6a**. Purification by column chromatography on silica using hexane/EtOac7:3 as eluent afforded the pure bromide (*E*)-**S6a** as a red solid in 25% yield. Spectroscopic data is in agreement with the literature report.[3]
Step 2. The bromide **S6a** (3 mmol) was dissolved in THF (4 mL) and DABCO (1.1 equiv.) was added to the solution. The mixture was then stirred at room temperature for 5h, the salt was purified by filtration and the filtrate was washed with Et_2O. (*E*)-**Va'** was obtained in 45% yield as an orange solid.

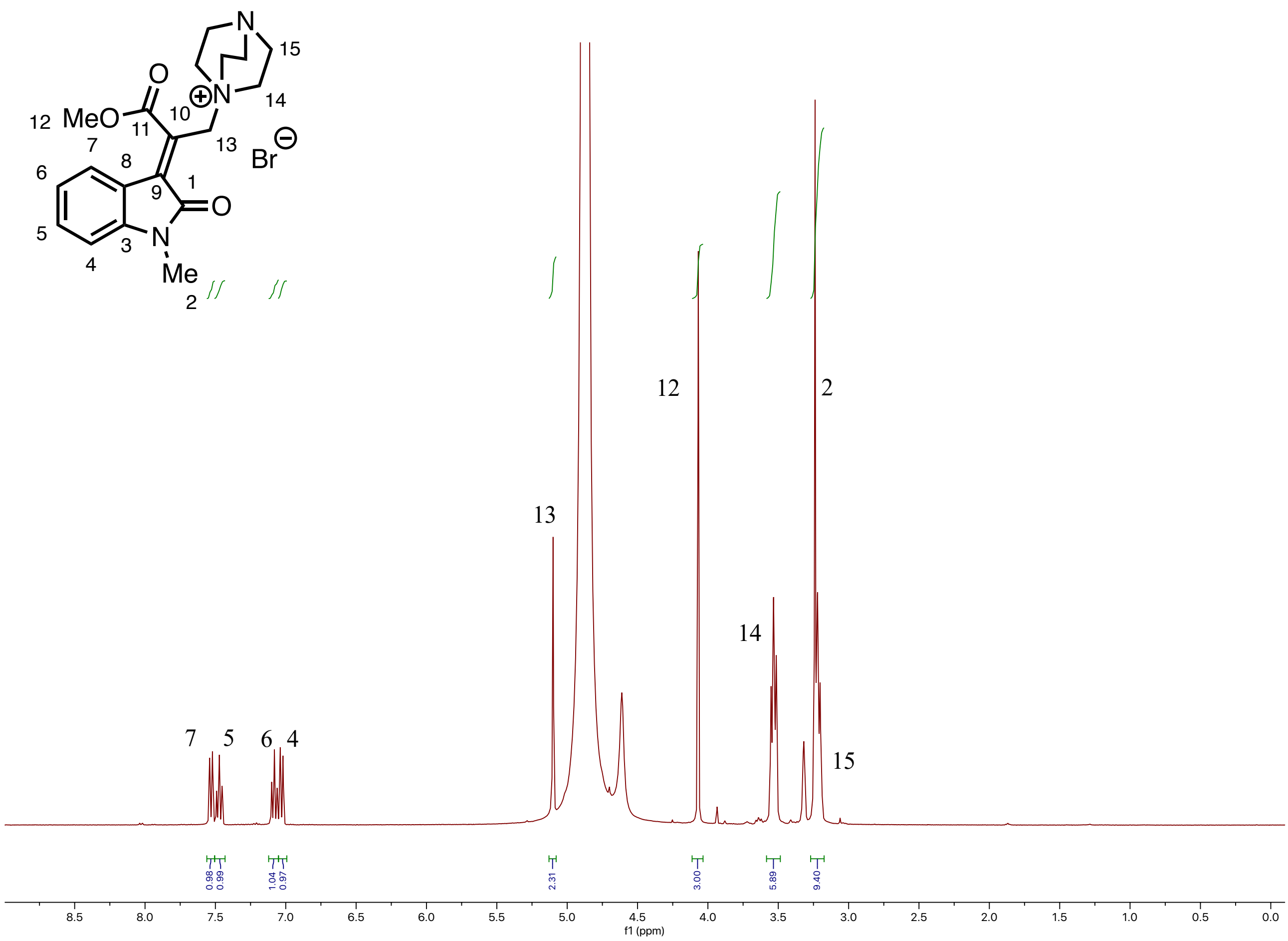

Figure S-38. ^{1}H NMR of the salt **Va'**

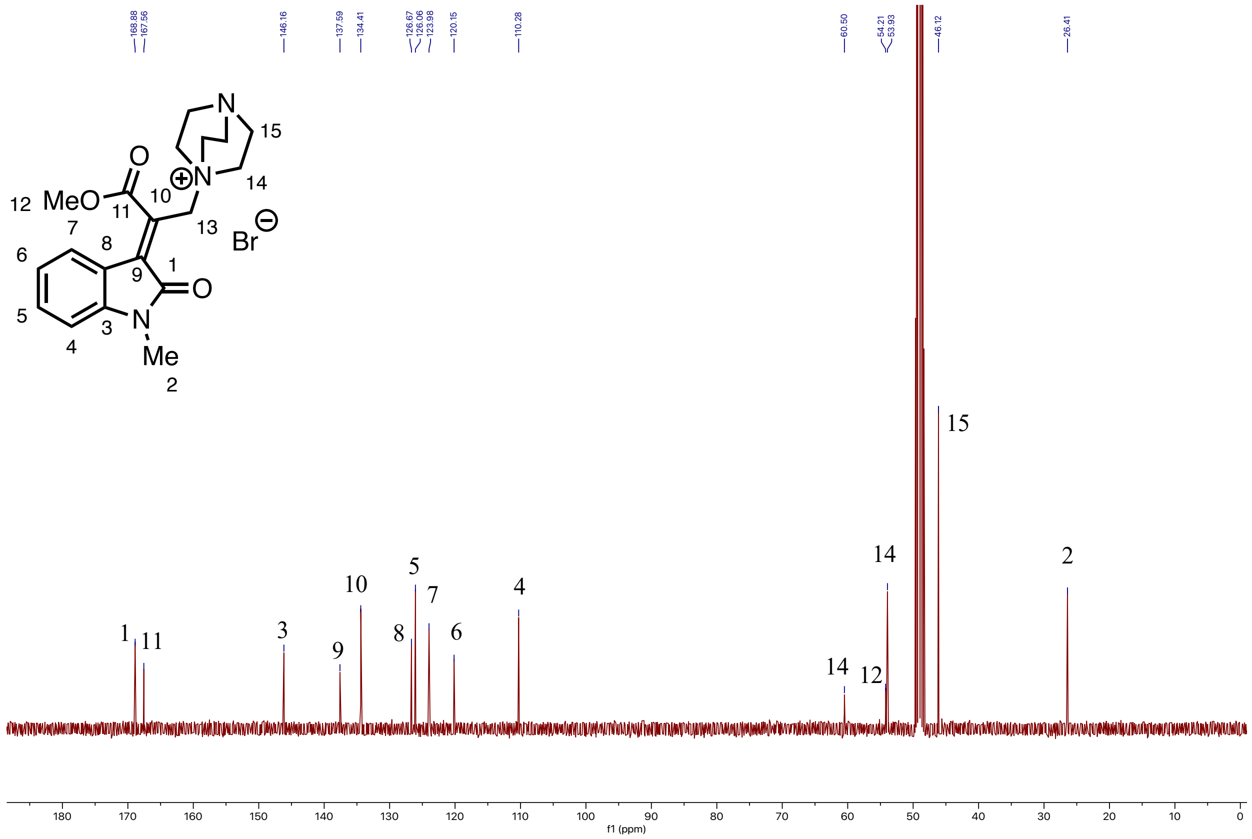

Figure S-39. ^{13}C NMR of the salt **Va'**

A solution of **Va’** in MeOH_d3 inside an NMR tube was irradiated at 365nm with a 9W bulb. ^{1}H NMR spectrum were acquired at regular intervals of time. As evidenced in Figure S-40, the initial *(E)* isomer converted smoothly to the corresponding *(Z)* isomer under 365nm light irradiation.

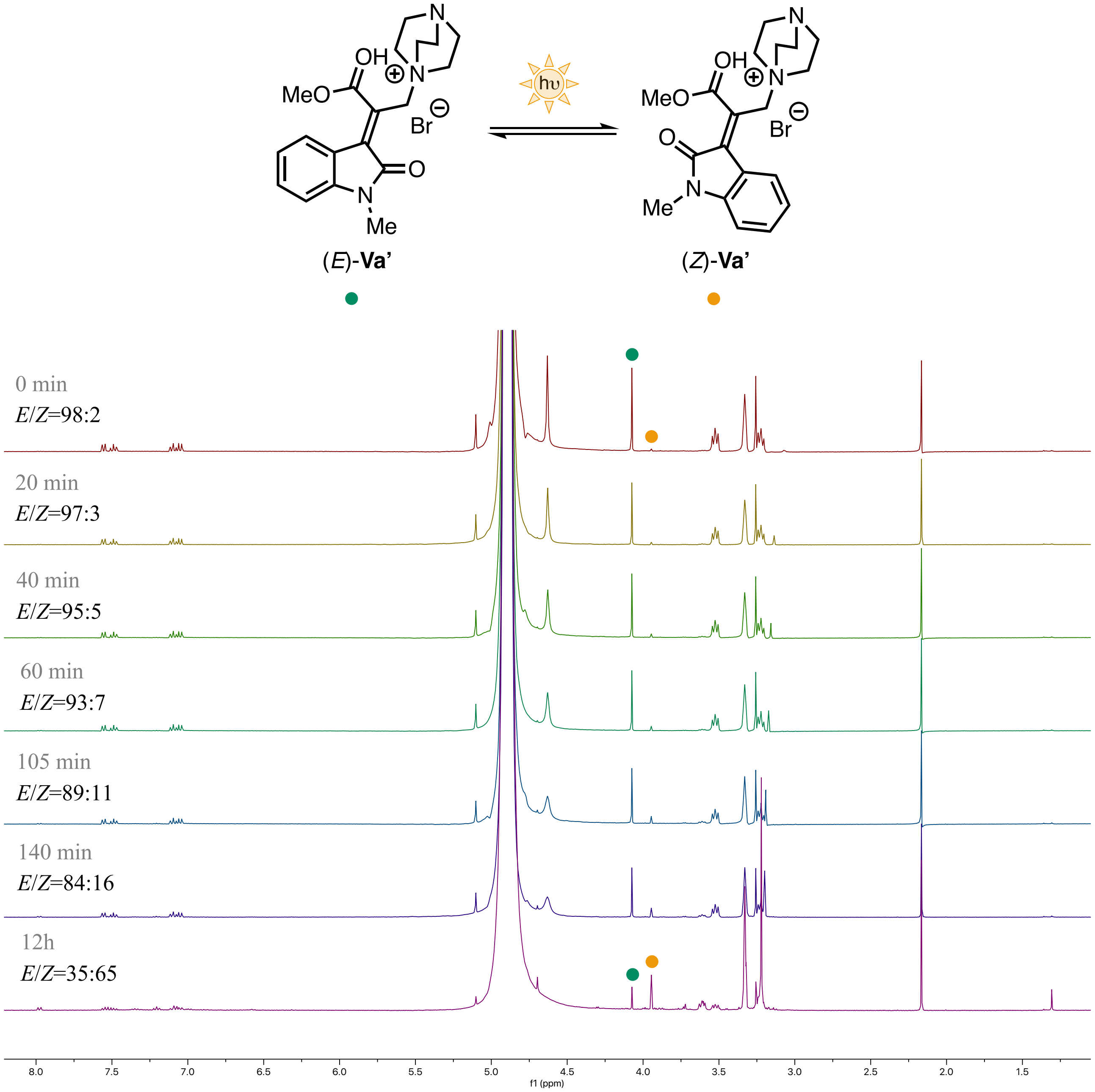

Figure S-40. Light-mediated isomerization experiment of the salt monitored by semi-continuous 1H-NMR. The evolution of the species was quantified integrating the singlet at 4.07 ppm for (*E*)-**Va’** and the at 3.95 ppm (*Z*)-**Va’**.

F. Determination of the absolute configuration

F.1. X-Ray diffraction of compound 6a

A nearly saturated solution of compound **6a** in CH_2Cl_2 was introduced into a 10 mL vial closed with a rubber septum. The septum was opened through a needle to allow the slow evaporation of the solvent. After two days, suitable crystals for single X-ray diffraction were obtained.

Data were collected using an Oxford Diffraction Gemini E diffractometer, equipped with a 2K × 2K EOS CCD area detector using a sealed–tube Enhance (Mo) source. A suitable single crystal of the compound was fastened on the top of a Lindemann glass capillary. Data were collected by means of the ω-scans technique using graphite-monochromated radiation. Detector distance has been set at 45 mm. The diffraction intensities were corrected for Lorentz/polarization effects as well as with respect to absorption. Empirical multi-scan absorption corrections using equivalent reflections were performed with the scaling algorithm SCALE3 ABSPACK. Data reduction, finalization and cell refinement were carried out through the CrysAlisPro software. Accurate unit cell parameters were obtained by least squares refinement of the angular settings of strongest reflections, chosen from the whole experiment. The structures were solved with Olex2[4] by using ShelXT[5] structure solution program by Intrinsic Phasing and refined with the ShelXL[6] refinement package using least-squares minimization. In the last cycles of refinement, non-hydrogen atoms were refined anisotropically. Hydrogen atoms were included in calculated positions, and a riding model was used for their refinement. A solvent molecule (CH2Cl2) was disordered over two sites, the occupancies of which were constrained to sum to 1.0. This disordered molecule was modelled using SADI and RIGU restraints. EADP constrain was applied to atoms C28A/C28B and Cl1A/Cl1B.

The specific refinement details are embedded in the CIF files given as Supplementary Materials and that have been deposited with the Cambridge Crystallographic Data Centre as supplementary publication (CCDC 1960705).

Table S-14. Crystal data and structure refinement for **6a**.

Identification code	6a
Empirical formula	$C_{28}H_{25}Cl_2NO_4$
Formula weight	510.39
Temperature/K	296.4(6)
Crystal system	triclinic
Space group	P-1
a/Å	8.7462(3)
b/Å	11.3434(5)
c/Å	14.7196(5)
α/°	74.989(3)
β/°	74.857(3)
γ/°	69.550(4)
Volume/Å^3	1297.89(9)
Z	2
ρ_{calc}g/cm^3	1.306
μ/mm^{-1}	0.284
F(000)	532.0

Crystal size/mm^3	0.45 × 0.25 × 0.2
Radiation	MoKα (λ = 0.71073)
2Θ range for data collection/°	5.062 to 58.536
Index ranges	$-11 \leq h \leq 11$, $-15 \leq k \leq 15$, $-19 \leq l \leq 20$
Reflections collected	40698
Independent reflections	6349 [R_{int} = 0.0560, R_{sigma} = 0.0403]
Data/restraints/parameters	6349/278/334
Goodness-of-fit on F^2	1.039
Final R indexes [I>=2σ (I)]	R_1 = 0.0747, wR_2 = 0.1998
Final R indexes [all data]	R_1 = 0.1113, wR_2 = 0.2292
Largest diff. peak/hole / e Å^{-3}	0.39/-0.38
CCDC	1960705

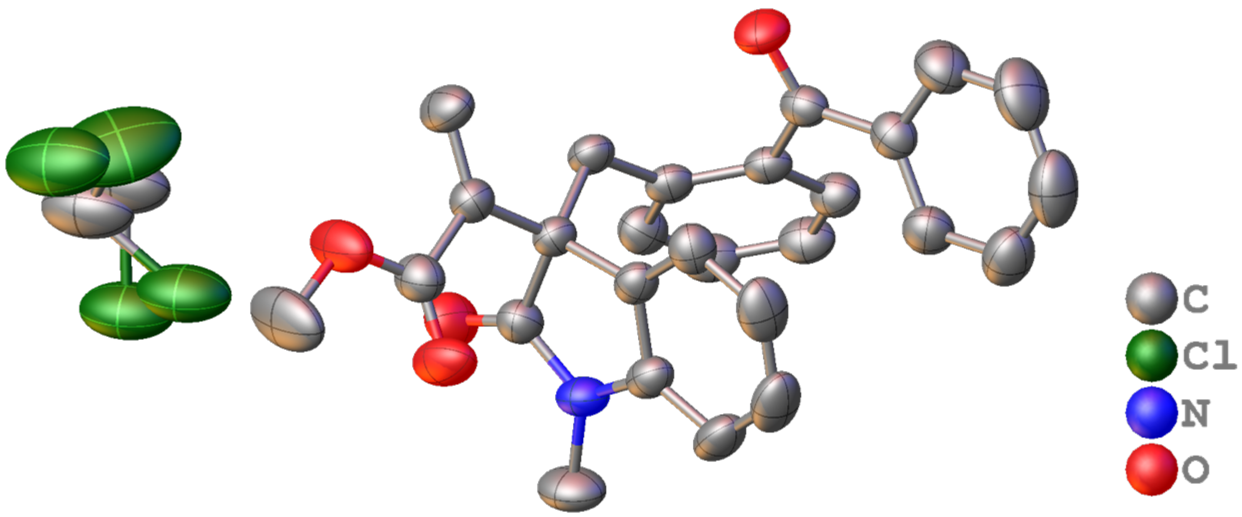

Figure S-41. Crystal structure of compound **6a**. Thermal ellipsoids drawn at the 50% probability level. H atoms omitted for clarity.

F.2. Absolute configuration of compound 6a

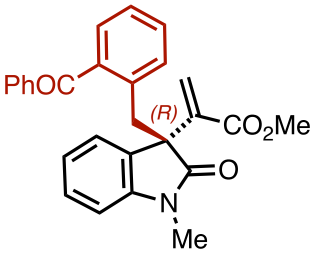

Despite many attempts, enantiopure single crystals suitable for X-ray diffraction[7] cannot be obtained. Also, in cases with high enantiomeric excess, all the single crystals were composed by the racemic mixture, as in the case of compound **6a**. For this reason, the absolute configuration of compound **6a** was determined by the theoretical simulations of chiro-optical spectra (electronic circular dichroism, ECD).[8]

Conformational analysis

In order to assign the absolute configuration of compound **6a**, a complete conformational analysis was performed in order to find all the low-energy conformations. A full scan of the potential energy surface (PES) was performed using molecular mechanics and the MMFF force field using Macromodel software (Maestro 11.8.012), the MMFF force field and the systematic torsional sampling (SPMC) method to explore the PES. All the energy minima enclosed in the lowest 10 kcal/mol range (52 conformations) were then optimized using DFT calculations at the B3LYP/6-31G(d) level of theory including the solvent with the SMD approach[9]. Frequency analysis was performed to confirm they corresponded to energy minima (no imaginary frequencies were observed), and to evaluate the ZPE correction to Free Energy. After this step, the energy was calculated as single-point energy at the higher SMD-B3LYP/6-311++G(2d,2p) level of theory, with the D3 empirical dispersion correction proposed by Grimme.[10]

The final energies for the evaluation of the conformational ratio were then obtained by adding the thermal correction to free energy from the lower level calculations to the electronic energies at the higher level (Table S-15). After the second step, 10 conformations were found to be enclosed in a 3 kcal/mol range, and 7 within the first 1.0 kcal/mol threshold. The 3D shape of the best seven conformations is shown in Figure S-42.

Table S-15. Relative energies of the conformations of **6a**.

	SMD-B3LYP/6-31G(d)		SMD-B3LYP/6-311++G(2d,2p)			
**Conf. #**	**G°(a.u.)**	**G_corr. (a.u.)**	**EE (a.u.)**	**EE+G_corr (a.u.)**	**Rel. G° (kcal/mol)**	Pop%
**10**	-1398.036893	0.379068	-1398.901981	-1398.522913	0.00	27
**7**	-1398.035488	0.378392	-1398.900946	-1398.522554	0.23	18
**1**	-1398.036970	0.378780	-1398.901163	-1398.522383	0.33	15
**4**	-1398.035672	0.378726	-1398.900966	-1398.522240	0.42	13
**18**	-1398.034015	0.378534	-1398.900700	-1398.522166	0.47	12
**8**	-1398.034075	0.377841	-1398.899708	-1398.521867	0.66	9
**2**	-1398.034616	0.378615	-1398.899954	-1398.521339	0.99	6
**20**	-1398.033734	0.378684	-1398.897331	-1398.518647	2.68	<1
**5**	-1398.034643	0.379191	-1398.897519	-1398.518328	2.88	<1
**21**	-1398.034776	0.378459	-1398.896692	-1398.518233	2.94	<1

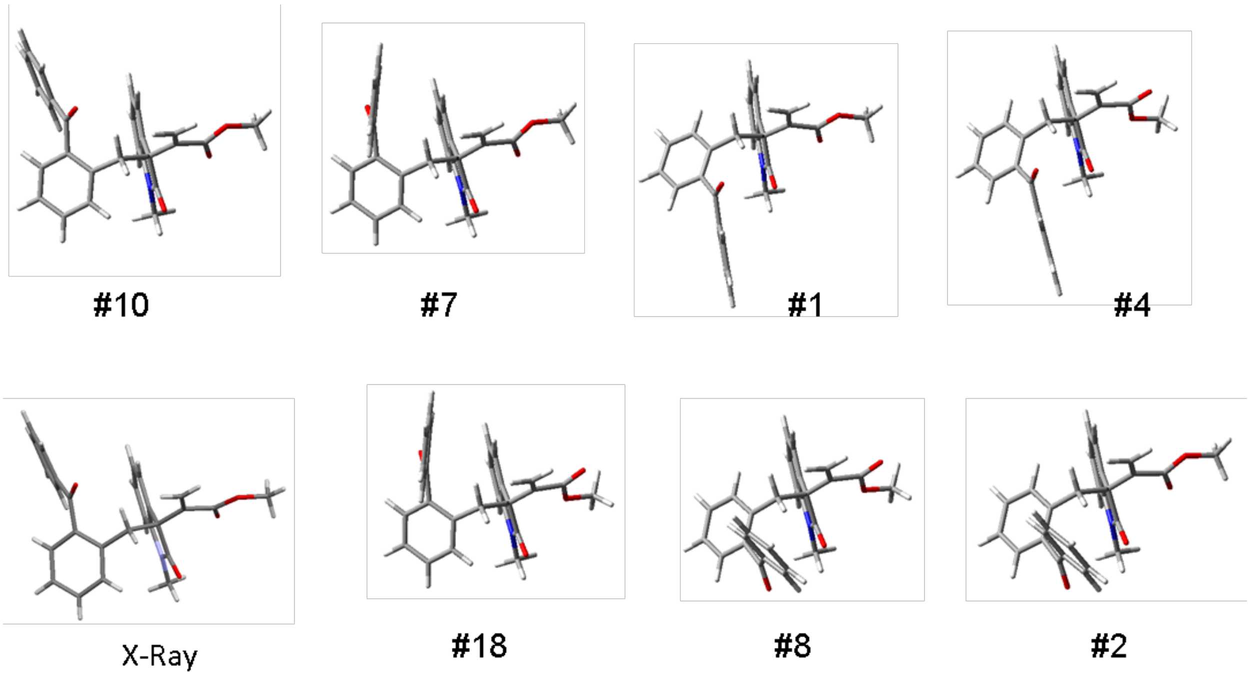

Figure S-42. The best seven conformations of **6a**. Geometry optimization at the SMD-B3LYP/6-31G(d) level. On the bottom left is reported the X-Ray structure of racemic **6a**, corresponding to conformation #10.

All the low-energy conformations have the same conformation in the proximity of the stereogenic carbon, i.e. the CH_2 in close proximity to the terminal CH_2 of the vinyl group, and the aromatic ring of benzophenone in *anti* disposition with respect to the quaternary carbon of the vinyl group. This in agreement with the NOESY spectrum obtained in **6a**, that shows a large cross peak enhancement for one of the vinyl hydrogens on saturation of the two diastereotopic hydrogens of the CH_2 (Figure S-43). It has to be noted that the best calculated conformation (#10) is that experimentally found in the solid state (Figure S-42). This is a good proof of the accuracy of the conformational search.

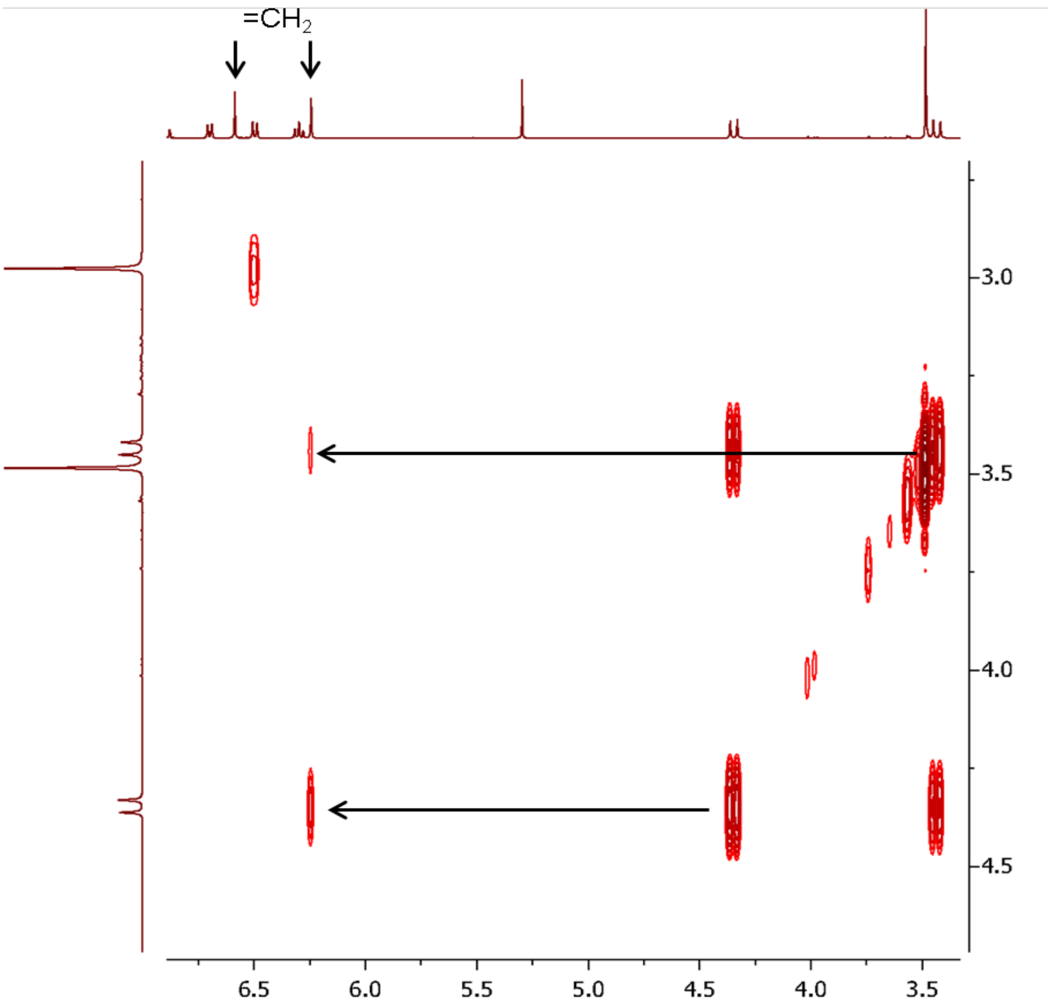

Figure S-43. Section of the NOESY spectrum of **6a** (400 MHz in $CDCl_3$), showing the NOE enhancement on the vinyl CH.

Absolute configuration

Due to the presence of good UV chromophores, in the present case the theoretical calculation of the ECD spectra of **6a** was selected for the absolute configuration assignment. The ECD spectrum of a 41% e.e. of **6a** was acquired in HPLC-grade acetonitrile solution ($1 \cdot 10^{-4}$ M) with a cell path of 0.2 cm in the 190-400 nm region by the average of 16 scans at 50 nm/min scan rate (Figure S-44 show the specific ECD spectra derived from the 41% e.e. sample). The experimental ECD spectrum exhibits two weak negative Cotton effects centred at 300 and 251 nm, a strong positive branch at 217 and a negative one at 190 nm.

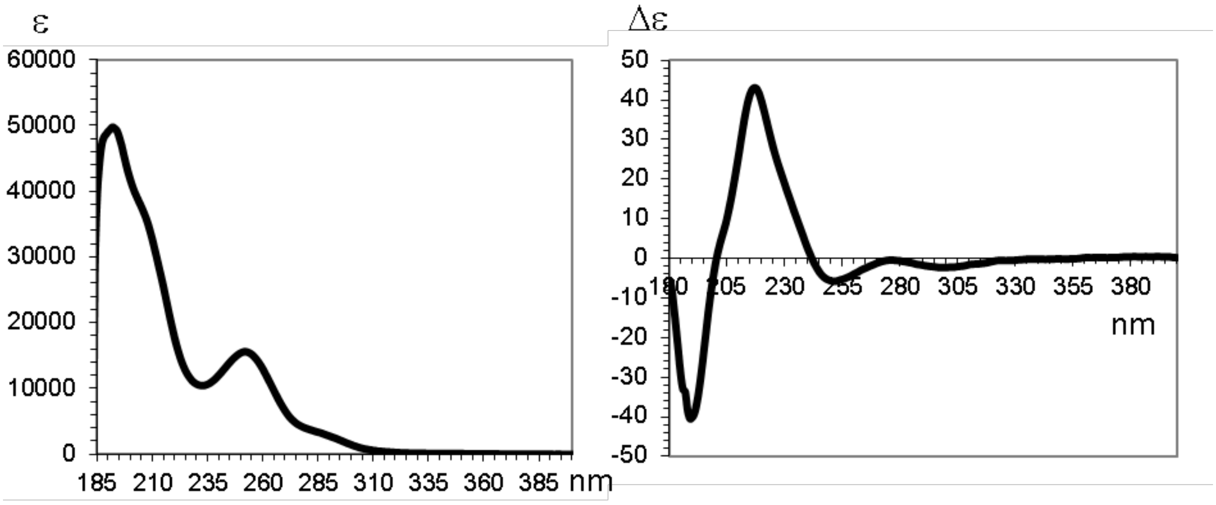

Figure S-44. UV and ECD spectrum of **6a** in acetonitrile. The intensity of the ECD spectrum has been scaled to the specific value by considering that the sample had 41% ee

The TD-DFT simulations of the ECD spectra were performed using the geometries of the best seven conformations (Figure S-42), and assuming the *R* absolute configuration. Calculations were performed with ωB97X-D that includes empirical dispersion,[11] and with CAM-B3LYP[12] that includes long range correction using the Coulomb Attenuating Method. All the calculations employed the 6-311++G(2d,p) basis set, that is known to yield good performances at a reasonable computational cost. [13]. For each conformation, the first 50 excited states were calculated, and the spectra were obtained using a 0.25 eV line width at half height. The simulation of the weighted spectrum was obtained by using the populations obtained from Boltzmann distribution using the population of Table S-15. The red shift to be applied to CAM-B3LYP simulations was evaluated on the UV spectrum as +11 nm (11 nm for ωB97X-D simulations). Scale factors were applied to the UV simulated spectrum (0.7 and 0.65 for CAM-B3LYP and ωB97X-D, respectively) and to the ECD simulation (0.5 and 0.6) to match the experimental spectra. The results of the TD-DFT calculations shown in Figure S-45 and Figure S-46. The two simulations are in a very good agreement with the experimental spectrum; it is thus safe to assign the *R* absolute configuration to **6a**.

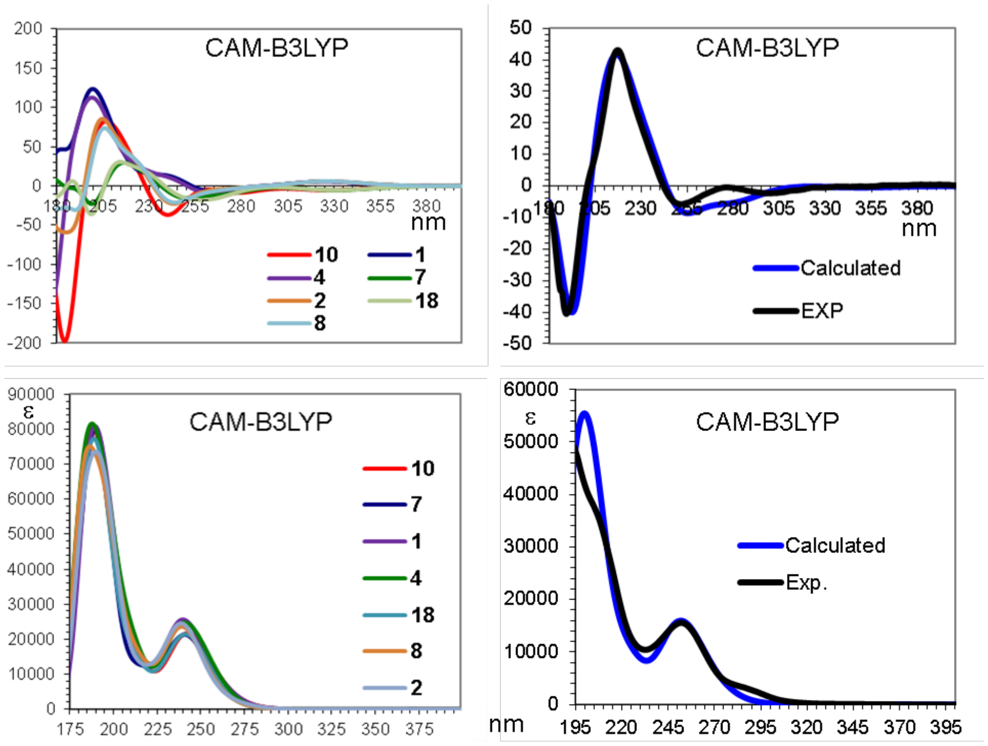

Figure S-45. Left: TD-DFT simulated spectra calculated for the best seven conformations of **6a** (S absolute configuration) using CAM-B3LYP/6-311++G(2d,p) basis set. On the left the spectra of the seven conformation are presented. On the right is shown the comparison with the experimental spectra (black traces) after Boltzmann averaging

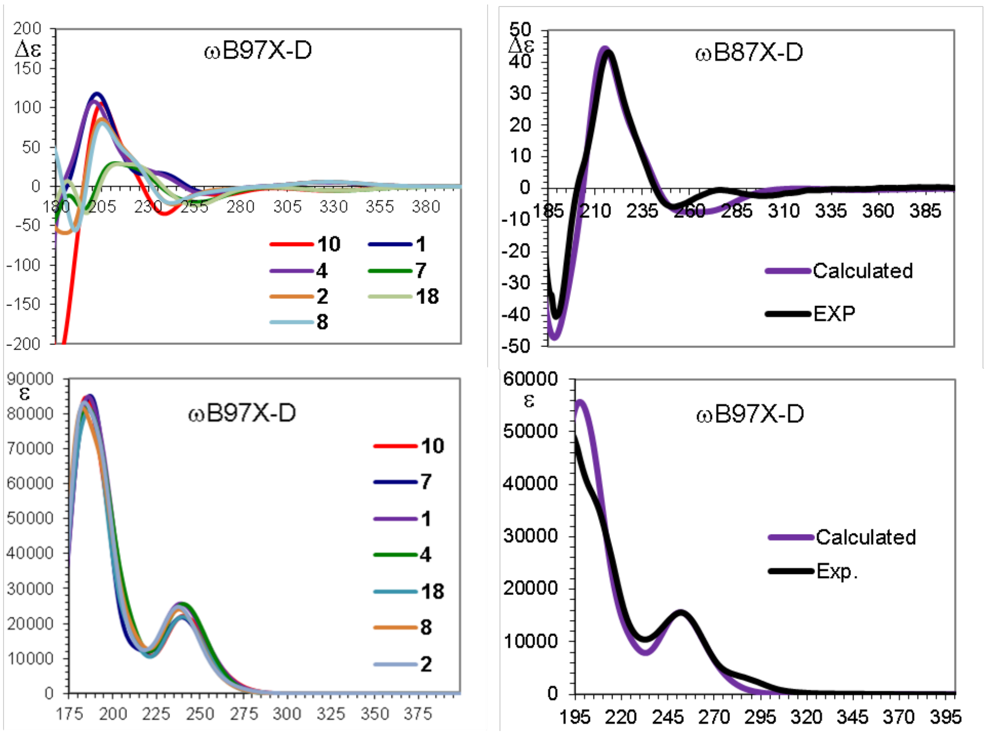

Figure S-46. Left: TD-DFT simulated spectra calculated for the best seven conformations of XX (S absolute configuration) using ωB97X-D /6-311++G(2d,p) basis set. On the left the spectra of the seven conformation are presented. On the right is shown the comparison with the experimental spectra (black traces) after Boltzmann averaging

Geometries of the optimized conformations

Compound 6a, conformation #1

0 1

C	-2.22985000	0.90323500	-0.04817700
C	-1.80719700	1.14507300	1.26873500
C	-2.40766200	2.11574900	2.06492300
C	-3.46643000	2.84871800	1.50833300
C	-3.90484700	2.61093900	0.20348300
C	-3.28283600	1.62808600	-0.58468000
C	-0.50016400	-0.60749300	0.57463400
O	0.31711300	-1.51637900	0.60476300
N	-0.75680700	0.27812500	1.59938000
C	-0.14133800	0.19002100	2.90983100
C	-1.35934100	-0.18806000	-0.65075600
C	-2.18927100	-1.36011100	-1.17999600
C	-2.24628500	-1.73606600	-2.46445700
C	-2.99716800	-2.09227300	-0.15139500
O	-2.92706100	-1.87345300	1.04634100
O	-3.80967200	-3.02538300	-0.67698000
C	-4.59252700	-3.78276300	0.26710300
C	-0.38718600	0.34752600	-1.76036600
C	0.59865600	1.42490900	-1.33875300
C	1.95856300	1.15939800	-1.03373200
C	0.15368200	2.75260700	-1.25647700
C	2.80191800	2.21741900	-0.64950300
C	1.00183300	3.79581600	-0.88288200
C	2.33516400	3.52834100	-0.57594300
C	2.60179000	-0.18645600	-1.25390800
O	2.38288000	-0.81560600	-2.28722600
C	3.57602800	-0.72224900	-0.24814900
C	3.59006700	-0.31051400	1.09450600
C	4.47695100	-1.71637900	-0.66700200
C	4.49071000	-0.87903200	1.99626800
C	5.38356300	-2.27394200	0.23056000
C	5.39142700	-1.85641400	1.56596800
H	-2.07648000	2.29851500	3.08239200
H	-3.95373100	3.61168600	2.10973100
H	-4.73139000	3.18598500	-0.20439500
H	-3.62513600	1.43751100	-1.59893000
H	0.33611400	1.13889000	3.17605700
H	-0.88902000	-0.05909900	3.67188200
H	0.61182400	-0.59807400	2.87298300
H	-2.87644600	-2.56299000	-2.77241100
H	-1.67753900	-1.24508100	-3.24562100
H	-5.17767700	-4.47896200	-0.33463900
H	-3.94179700	-4.32929700	0.95487100
H	-5.25361500	-3.12256700	0.83464700
H	-1.00895700	0.74874700	-2.56727700
H	0.15694900	-0.50709500	-2.15859500

H	-0.88005000	2.97317100	-1.50412900
H	3.84624100	2.01219500	-0.43591200
H	0.62074200	4.81268900	-0.84084400
H	3.01064600	4.33069200	-0.29263900
H	2.88891800	0.44119800	1.44031000
H	4.45554400	-2.03810200	-1.70338200
H	4.48766300	-0.55941700	3.03471900
H	6.08292200	-3.03404600	-0.10703800
H	6.09634100	-2.29335600	2.26835800

Compound 6a, conformation #2

0 1

C	-2.06587800	0.57686100	-0.60224600
C	-2.29206400	1.26642800	0.60041400
C	-3.25678300	2.26386900	0.70332100
C	-4.01276800	2.55706300	-0.44154700
C	-3.80804200	1.87012600	-1.64039600
C	-2.82579200	0.86898600	-1.72435000
C	-0.69457400	-0.28708600	1.13957300
O	0.07522800	-0.96397300	1.80467900
N	-1.43696700	0.77923000	1.59843000
C	-1.44302700	1.21466300	2.98169700
C	-0.93607200	-0.41745400	-0.38992200
C	-1.33384700	-1.84605800	-0.76520800
C	-0.73021900	-2.58911900	-1.70200400
C	-2.49088500	-2.41016100	0.00349900
O	-2.99042900	-1.86399400	0.97271000
O	-2.92497400	-3.58849600	-0.47612400
C	-4.01332100	-4.19925200	0.24536100
C	0.39522100	-0.00301400	-1.11305800
C	0.92762800	1.38975500	-0.81542100
C	1.94106400	1.66251600	0.13458100
C	0.37716900	2.47682400	-1.51555800
C	2.31612500	2.99438500	0.38965000
C	0.77810500	3.79030600	-1.27794600
C	1.74845200	4.05465600	-0.30992100
C	2.69602200	0.63826700	0.94933600
O	2.78553300	0.79125200	2.16376900
C	3.44169600	-0.47407600	0.27811500
C	3.80764800	-0.42035000	-1.07652100
C	3.85320800	-1.56872700	1.05731800
C	4.56871500	-1.44522700	-1.64066800
C	4.59867400	-2.59805500	0.48904500
C	4.96029000	-2.53672600	-0.86157500
H	-3.42726900	2.79331700	1.63553800
H	-4.77436400	3.33058900	-0.38709000
H	-4.41073100	2.10882600	-2.51217900
H	-2.66684700	0.33066600	-2.65549900
H	-0.64720700	0.68015400	3.50234000
H	-1.25750900	2.29184000	3.04280600

H	-2.40431800	0.98937300	3.45847500
H	-1.07828700	-3.59195400	-1.92320100
H	0.12092300	-2.23678700	-2.27306000
H	-4.22083300	-5.13237700	-0.27928500
H	-3.72327800	-4.40120000	1.27991600
H	-4.89393400	-3.55173200	0.23127500
H	0.21631400	-0.07579900	-2.19026200
H	1.13632900	-0.75976500	-0.85350200
H	-0.38453600	2.28241200	-2.26424700
H	3.08139800	3.18392900	1.13634800
H	0.33260100	4.60212500	-1.84670400
H	2.06701500	5.07380000	-0.10885400
H	3.51103000	0.42760700	-1.68605800
H	3.57455600	-1.59909600	2.10579200
H	4.85640100	-1.39027300	-2.68697400
H	4.90114800	-3.44739900	1.09564900
H	5.54684300	-3.33759100	-1.30392300

Compound 6a, conformation #4

0 1

C	-2.31145900	0.71057800	-0.12649800
C	-1.94441300	0.85402300	1.22093100
C	-2.62662500	1.70981700	2.08022900
C	-3.71147600	2.42761900	1.55496100
C	-4.09557600	2.28555100	0.21942500
C	-3.39072800	1.41851500	-0.63227000
C	-0.50300600	-0.74007200	0.41503000
O	0.37064800	-1.59507200	0.39026500
N	-0.85340000	0.02129700	1.50908100
C	-0.26106700	-0.13745500	2.82395600
C	-1.35304700	-0.26193900	-0.79505300
C	-2.07924500	-1.42248300	-1.48201800
C	-2.04523300	-1.66459600	-2.79911000
C	-2.93441000	-2.36109800	-0.67380300
O	-3.61282800	-3.25599300	-1.14572900
O	-2.86623100	-2.11539400	0.64558800
C	-3.64966100	-2.97659800	1.49602400
C	-0.37864700	0.44897400	-1.80339100
C	0.51556000	1.53797100	-1.23350600
C	1.87870800	1.32980200	-0.89916600
C	-0.02121300	2.81898100	-1.03745600
C	2.63365700	2.39437900	-0.37483600
C	0.74017900	3.87024900	-0.52524100
C	2.07658400	3.65822400	-0.18987400
C	2.61951700	0.05799000	-1.22685400
O	2.47878000	-0.47492100	-2.32578800
C	3.59341200	-0.51471300	-0.24129000
C	3.53264200	-0.24489900	1.13567100
C	4.57386700	-1.40007500	-0.72110900
C	4.43779700	-0.84550300	2.01177700

C	5.48445600	-1.98885000	0.15216000
C	5.41729300	-1.71309800	1.52225200
H	-2.33718200	1.81789700	3.12078500
H	-4.26248600	3.10202400	2.20518400
H	-4.94368700	2.84612800	-0.16352200
H	-3.68977600	1.30322400	-1.67119000
H	0.14861400	0.81398800	3.17903200
H	-1.00724200	-0.49441000	3.54318000
H	0.54162900	-0.87141800	2.74283700
H	-2.61508500	-2.49360500	-3.20651200
H	-1.47222300	-1.07020200	-3.49999300
H	-3.47042300	-2.62284700	2.51158500
H	-4.71065800	-2.89619100	1.24609600
H	-3.32474500	-4.01484200	1.39070100
H	-0.99777700	0.89063900	-2.59079000
H	0.23711000	-0.32263500	-2.26169200
H	-1.05796500	2.99728400	-1.30556900
H	3.68134000	2.23619300	-0.13893900
H	0.28964200	4.85099900	-0.39784200
H	2.68562800	4.46850600	0.20109100
H	2.77092600	0.42118800	1.52616400
H	4.61047600	-1.61293600	-1.78478500
H	4.37700900	-0.63652400	3.07633100
H	6.24521200	-2.66338300	-0.23140400
H	6.12531600	-2.17487700	2.20535600

Compound 6a, conformation #5

0 1

C	-1.49294900	0.01198400	-0.85780400
C	-2.13595600	1.23493200	-1.11153600
C	-2.30824400	1.72483800	-2.40234300
C	-1.82884900	0.94176800	-3.46237100
C	-1.20538600	-0.28562700	-3.22800000
C	-1.03548300	-0.75852700	-1.91620500
C	-2.29388100	0.99106600	1.16176900
O	-2.58589600	1.21734600	2.32851500
N	-2.56020900	1.81326700	0.09296700
C	-3.30935200	3.05034900	0.19801600
C	-1.46735000	-0.22642600	0.64640300
C	-2.16026500	-1.53440200	1.04477700
C	-1.62268900	-2.49145500	1.81254100
C	-3.57084100	-1.69191800	0.56132400
O	-4.26836600	-0.76438800	0.18760000
O	-4.00118600	-2.96530500	0.59313700
C	-5.37389300	-3.17486600	0.20473800
C	-0.03598400	-0.14985000	1.28471300
C	0.84723700	1.01703000	0.86950300
C	2.06010600	0.85172300	0.15231300
C	0.51225300	2.31387300	1.29158200
C	2.83249400	1.98038000	-0.18015900

C	1.27939900	3.42611900	0.94636700
C	2.43650100	3.26387600	0.18440300
C	2.57177300	-0.49298400	-0.28477100
O	1.82441700	-1.34277700	-0.76578200
C	4.03179300	-0.80607600	-0.13054100
C	4.84009300	-0.20492900	0.84859800
C	4.58414700	-1.80081100	-0.95641300
C	6.17402900	-0.58965700	0.99356900
C	5.91995200	-2.16914600	-0.82349300
C	6.71783300	-1.56493300	0.15461300
H	-2.80487900	2.67225100	-2.58715600
H	-1.95225200	1.29888600	-4.48152500
H	-0.84817100	-0.88019500	-4.06432700
H	-0.54115400	-1.70593500	-1.73365800
H	-4.26574100	2.97033600	-0.33151900
H	-2.73587900	3.88244800	-0.22376100
H	-3.49704700	3.23832500	1.25606700
H	-2.18416200	-3.38833300	2.05043500
H	-0.62724700	-2.42037700	2.23497600
H	-5.54092700	-4.24831000	0.29926000
H	-6.04922900	-2.62551900	0.86608500
H	-5.53262900	-2.85532200	-0.82848400
H	0.47278400	-1.08382100	1.05811200
H	-0.18798500	-0.10514400	2.36985200
H	-0.36297200	2.45511300	1.91730100
H	3.74983900	1.84525000	-0.74442100
H	0.97534800	4.41444900	1.28083900
H	3.03822900	4.12209400	-0.10105100
H	4.42462100	0.54760000	1.51063100
H	3.95307200	-2.27184300	-1.70342900
H	6.78728300	-0.12731300	1.76214400
H	6.34072300	-2.92806900	-1.47754500
H	7.75916100	-1.85643500	0.26307000

Compound 6a, conformation #7

0 1

C	-1.19304900	-0.17884700	0.86519300
C	-1.97075800	-1.34855900	0.87974100
C	-2.25759100	-2.02547900	2.06045700
C	-1.75020200	-1.49127200	3.25450400
C	-0.98763800	-0.32174600	3.25725000
C	-0.70344600	0.34292700	2.05237300
C	-1.98086600	-0.70337100	-1.32048100
O	-2.24187600	-0.68528100	-2.51580700
N	-2.38724700	-1.66221200	-0.42182100
C	-3.25274300	-2.77307100	-0.76804100
C	-1.05295900	0.29797000	-0.57211100
C	-1.54401600	1.73488800	-0.76889400
C	-0.81810500	2.73225900	-1.29106800
C	-2.96391900	1.98938300	-0.36082600
O	-3.77437700	1.10525400	-0.14115600

O	-3.26376100	3.29655200	-0.26909700
C	-4.63206700	3.60582100	0.06395900
C	0.40488500	0.13359200	-1.12560300
C	1.05250800	-1.23299700	-0.96315500
C	2.05745600	-1.49886500	-0.00472100
C	0.64600600	-2.28913700	-1.79632200
C	2.56577500	-2.80213000	0.13530700
C	1.17793600	-3.57179800	-1.67190600
C	2.13704000	-3.83564300	-0.69234200
C	2.66079500	-0.47776500	0.93017200
O	2.65183200	-0.69071600	2.13947900
C	3.36170200	0.72793500	0.38470700
C	3.77103900	0.81609000	-0.95606400
C	3.67235400	1.77666200	1.26787900
C	4.47419600	1.93496100	-1.40407000
C	4.36190800	2.89818900	0.81576000
C	4.76589500	2.97835900	-0.52178300
H	-2.85900700	-2.92908700	2.06377200
H	-1.96189900	-1.99905800	4.19186900
H	-0.60951300	0.07697600	4.19435300
H	-0.11225000	1.25484300	2.05416300
H	-4.21954700	-2.68424900	-0.25938400
H	-2.78672500	-3.72348700	-0.48750600
H	-3.40931000	-2.75035600	-1.84742800
H	-1.23875100	3.72651200	-1.39399400
H	0.20057500	2.60221900	-1.63743800
H	-5.31147600	3.21032300	-0.69582100
H	-4.89214400	3.18961100	1.04066300
H	-4.68473700	4.69464800	0.08960500
H	1.01551900	0.89248000	-0.63662300
H	0.36948400	0.38315900	-2.19202900
H	-0.10212600	-2.09759500	-2.55947500
H	3.32173400	-2.98956600	0.89239000
H	0.84263800	-4.36131300	-2.33920900
H	2.55396400	-4.83270400	-0.58031400
H	3.55229100	0.00644100	-1.64508000
H	3.36281900	1.69689500	2.30514900
H	4.79526500	1.99108700	-2.44047400
H	4.58811300	3.70933700	1.50244800
H	5.30845200	3.85175100	-0.87374300

Compound 6a, conformation #8

0 1

C	-2.07344100	0.34299900	-0.74061400
C	-2.42370100	0.99132900	0.45504500
C	-3.47234900	1.90401600	0.51416900
C	-4.18514700	2.15154100	-0.66857200
C	-3.85857500	1.50132500	-1.86093900
C	-2.79245900	0.58717900	-1.90044200
C	-0.72689200	-0.42773900	1.06489300
O	0.06040000	-1.04687800	1.76470600

N	-1.58956000	0.55686600	1.49543900
C	-1.70088700	0.97682200	2.87953400
C	-0.87362800	-0.55185700	-0.47730100
C	-1.10586600	-2.00095900	-0.91255200
C	-0.37165400	-2.64971600	-1.82604000
C	-2.24372900	-2.78807200	-0.31948800
O	-2.58495800	-3.89593600	-0.69347500
O	-2.84740200	-2.14053400	0.69141600
C	-3.94403500	-2.82945800	1.32515800
C	0.45216200	0.00706200	-1.11417500
C	0.85071000	1.42343200	-0.73195500
C	1.81191000	1.72817200	0.26178100
C	0.22853900	2.49657900	-1.39217000
C	2.06323400	3.07056700	0.59922800
C	0.50863200	3.82413100	-1.07287900
C	1.42505300	4.11587500	-0.06112900
C	2.63466600	0.72649600	1.03761500
O	2.68887800	0.81582500	2.26040600
C	3.48348400	-0.28069800	0.32374300
C	3.95828900	-1.38740000	1.04789100
C	3.88074800	-0.11356100	-1.01278800
C	4.79750600	-2.31871500	0.44235000
C	4.73545900	-1.03920100	-1.61311000
C	5.19021100	-2.14443400	-0.88953300
H	-3.73755800	2.40404200	1.44047600
H	-5.01032500	2.85862500	-0.64913300
H	-4.43047300	1.70210800	-2.76249300
H	-2.53762200	0.07807200	-2.82667700
H	-2.67397700	0.69269000	3.29659300
H	-1.58084400	2.06211600	2.95997700
H	-0.90772100	0.48225500	3.44197700
H	-0.62561800	-3.67151300	-2.08951300
H	0.47993300	-2.20965500	-2.33029400
H	-3.60095100	-3.76956600	1.76456400
H	-4.73770500	-3.03007800	0.60099600
H	-4.30073600	-2.15389100	2.10283700
H	0.33034400	-0.03450800	-2.20097200
H	1.24266500	-0.69423600	-0.84650900
H	-0.49171100	2.28057500	-2.17503500
H	2.78922500	3.28241100	1.37844700
H	0.01142600	4.62565600	-1.61265300
H	1.64864500	5.14569500	0.20349600
H	3.65420700	-1.50527700	2.08308300
H	3.53527900	0.74604800	-1.57877700
H	5.14896500	-3.17898100	1.00559300
H	5.04671300	-0.89646200	-2.64428000
H	5.84996200	-2.86831500	-1.36052300

Compound 6a, conformation #10

0 1

C	-0.86960400	0.54443800	0.46813500
C	-1.01710000	-0.11652400	1.69911700
C	-0.47140200	0.38986700	2.87449400
C	0.22633300	1.60475800	2.79581500
C	0.36866200	2.28017000	1.58183300
C	-0.18185300	1.74641700	0.40415200
C	-2.24456000	-1.38525900	0.23610100
O	-2.98925200	-2.25871900	-0.18732300
N	-1.78668100	-1.27604200	1.52976000
C	-2.19594800	-2.16130900	2.60315800
C	-1.57358800	-0.26461000	-0.60983400
C	-2.62984700	0.55274900	-1.35895400
C	-2.65237300	0.74884700	-2.68383900
C	-3.71162200	1.14183900	-0.50452700
O	-3.90658900	0.82454500	0.65669300
O	-4.45882600	2.05534800	-1.14831300
C	-5.54773300	2.62296700	-0.39306900
C	-0.57891600	-0.93471300	-1.61851100
C	0.52862700	-1.79958100	-1.03437400
C	1.87397300	-1.36993100	-0.89357300
C	0.21429200	-3.10978300	-0.64194900
C	2.83278800	-2.25736100	-0.37012700
C	1.17472300	-3.98101600	-0.12737800
C	2.49509600	-3.55472600	0.00747800
C	2.37197500	-0.04568700	-1.40877700
O	1.98321100	0.39570500	-2.48956800
C	3.40096900	0.71913600	-0.62928500
C	3.56438000	0.58351000	0.75929300
C	4.19025900	1.65404200	-1.32184300
C	4.50332400	1.36263300	1.43722100
C	5.13662800	2.41968000	-0.64661100
C	5.29468900	2.27551800	0.73626700
H	-0.59071900	-0.12516800	3.82256900
H	0.65740900	2.02553200	3.70043000
H	0.90825700	3.22217400	1.54536200
H	-0.07446300	2.27278400	-0.54065000
H	-2.78577400	-2.96717100	2.16405700
H	-1.31979400	-2.58371400	3.10594300
H	-2.80640200	-1.62413200	3.33835900
H	-3.43197200	1.34832300	-3.14079800
H	-1.91412000	0.32453500	-3.35437300
H	-6.03097700	3.32948400	-1.06854600
H	-6.25248100	1.84416300	-0.08974500
H	-5.17099400	3.14132500	0.49251900
H	-0.13920500	-0.14862600	-2.22860800
H	-1.18423200	-1.56460000	-2.27973400
H	-0.80818800	-3.45672400	-0.75594200
H	3.86500000	-1.93318500	-0.28682200
H	0.89054400	-4.99131900	0.15478500

H	3.25825700	-4.22472100	0.39336700
H	2.94665800	-0.11409200	1.31450700
H	4.05254200	1.76385700	-2.39283700
H	4.61491300	1.25678500	2.51279000
H	5.75035700	3.12984600	-1.19415600
H	6.03077800	2.87547200	1.26482200

Compound 6a, conformation #18

0 1

C	-1.17766200	-0.04988500	-0.84669200
C	-2.05375300	1.04784300	-0.87485000
C	-2.39501600	1.68576600	-2.06257500
C	-1.84059600	1.18475900	-3.25020200
C	-0.97965600	0.08576900	-3.23939300
C	-0.64150600	-0.53943600	-2.02735600
C	-2.00919900	0.42970700	1.33422400
O	-2.27028400	0.40134800	2.52931000
N	-2.50209200	1.33470300	0.42331400
C	-3.44957800	2.38025800	0.75967700
C	-0.99715000	-0.49420300	0.59644900
C	-1.33373400	-1.97274500	0.81767300
C	-0.49758700	-2.87721800	1.34448500
C	-2.68707300	-2.50105600	0.42273900
O	-2.97935900	-3.68250600	0.37593300
O	-3.56170400	-1.52060300	0.14173900
C	-4.88904200	-1.93923500	-0.23507200
C	0.44001300	-0.17474100	1.14354700
C	0.94511600	1.24894300	0.96742500
C	1.90630100	1.60956300	-0.00490100
C	0.44613700	2.26270800	1.80298200
C	2.28001400	2.95643800	-0.15544100
C	0.84579200	3.59151100	1.66800600
C	1.76102300	3.94546800	0.67497200
C	2.59799900	0.64945500	-0.94336800
O	2.54818500	0.85101900	-2.15351900
C	3.42909700	-0.47191300	-0.40088800
C	3.85918500	-0.50861000	0.93581600
C	3.83962900	-1.48713300	-1.28245800
C	4.68072800	-1.54458300	1.38166300
C	4.64828500	-2.52695600	-0.83240400
C	5.07196900	-2.55637900	0.50113600
H	-3.07166000	2.53442000	-2.07623000
H	-2.09259000	1.66291700	-4.19313100
H	-0.56614600	-0.28830000	-4.17167300
H	0.02705500	-1.39619200	-2.01868500
H	-4.40228800	2.22073800	0.24213300
H	-3.05138900	3.36174200	0.48141400
H	-3.61375500	2.34744800	1.83764100
H	-0.82069500	-3.90840100	1.44615600
H	0.50346000	-2.64255300	1.68479500

H	-4.85220300	-2.55880600	-1.13468700
H	-5.35761000	-2.49924000	0.57824100
H	-5.43955000	-1.01856600	-0.42962200
H	1.12251100	-0.87172200	0.65808700
H	0.43312300	-0.41662100	2.21209400
H	-0.26940200	2.00036900	2.57628800
H	3.00372700	3.21548100	-0.92281900
H	0.44246000	4.34628400	2.33786000
H	2.07520200	4.97858800	0.55467800
H	3.56343700	0.27753500	1.62332700
H	3.51262400	-1.44659100	-2.31662600
H	5.01623000	-1.56062700	2.41486200
H	4.95193600	-3.31370600	-1.51759000
H	5.70703200	-3.36574500	0.85139600

Compound 6a, conformation #20

0 1

C	1.49806000	0.18349100	-0.79823800
C	2.21046200	-0.99461700	-1.07741200
C	2.42440600	-1.43819600	-2.37851100
C	1.91453000	-0.65295300	-3.42272900
C	1.22237800	0.53164600	-3.16273000
C	1.01139400	0.95758200	-1.84083500
C	2.32139500	-0.81159700	1.20580200
O	2.60793400	-1.05682300	2.36995300
N	2.65584100	-1.58030100	0.11687000
C	3.45611100	-2.78730500	0.19913200
C	1.43849800	0.37466400	0.71160600
C	2.03286400	1.71354200	1.16667400
C	1.42953800	2.58181900	1.98915700
C	3.39870500	2.12756700	0.68813700
O	3.82009000	3.26990300	0.71048100
O	4.12974500	1.08595800	0.25641500
C	5.45902700	1.38995700	-0.21212900
C	0.00129300	0.18676300	1.31946700
C	-0.80451400	-1.01094500	0.83991000
C	-2.01273500	-0.88548800	0.10687800
C	-0.40223200	-2.30308400	1.21505700
C	-2.71188800	-2.04156300	-0.28758300
C	-1.09730500	-3.44199400	0.80940200
C	-2.24794500	-3.31444800	0.03120400
C	-2.59579600	0.44513400	-0.28163700
O	-1.89255000	1.35680000	-0.71335600
C	-4.07363800	0.66639200	-0.13881500
C	-4.86049400	-0.02575200	0.79673600
C	-4.67036500	1.66549200	-0.92781600
C	-6.21679100	0.27392400	0.93480200
C	-6.02732100	1.94942900	-0.80241800
C	-6.80360500	1.25461600	0.13195000
H	2.97362400	-2.35195200	-2.58281100

H	2.06856900	-0.97409400	-4.44962700
H	0.84273100	1.12914900	-3.98696600
H	0.46291100	1.87056600	-1.63869000
H	4.40874500	-2.65678700	-0.32669500
H	2.91860200	-3.63398400	-0.24077100
H	3.64974800	-2.98831400	1.25368600
H	1.92667400	3.51219700	2.24513500
H	0.45525900	2.41135900	2.43039000
H	5.88133600	0.43525000	-0.52643600
H	6.05718700	1.82500300	0.59260900
H	5.41666500	2.08403900	-1.05540400
H	-0.55657400	1.09866300	1.12066100
H	0.13709200	0.10524500	2.40444200
H	0.46803500	-2.42046200	1.85256400
H	-3.62569100	-1.93576200	-0.86379900
H	-0.74258300	-4.42441200	1.10959300
H	-2.79348800	-4.19297000	-0.30145400
H	-4.41221200	-0.78315900	1.43114100
H	-4.05633600	2.20685400	-1.64060500
H	-6.81376600	-0.25854200	1.67009100
H	-6.48159100	2.71289700	-1.42814600
H	-7.86176100	1.48016000	0.23469300

Compound 6a, conformation #21

0 1

C	-2.24937900	0.94800000	0.31199100
C	-1.69409900	1.84394500	1.24188000
C	-2.27191300	3.07761800	1.52612300
C	-3.45126000	3.41332800	0.84824900
C	-4.02052700	2.53675900	-0.07787600
C	-3.42142400	1.29635400	-0.34931100
C	-0.25855100	0.05664200	1.27896200
O	0.70649000	-0.63883000	1.57011600
N	-0.52244200	1.30279100	1.79221800
C	0.31944000	1.97001600	2.76826000
C	-1.37122600	-0.30094500	0.25539400
C	-2.13100800	-1.55397500	0.73143800
C	-2.00019400	-2.07721600	1.95923900
C	-3.10389300	-2.18123500	-0.22386600
O	-3.47915800	-1.67981200	-1.27208600
O	-3.53801800	-3.38395800	0.19254100
C	-4.50582700	-4.03376700	-0.65571400
C	-0.67436900	-0.57931300	-1.12789900
C	0.28356500	0.49201500	-1.62088700
C	1.69507600	0.37949900	-1.53580400
C	-0.24885200	1.65565300	-2.19641800
C	2.50451900	1.42575600	-2.01368800
C	0.56397800	2.68429700	-2.67415900
C	1.95061700	2.57056200	-2.58317700
C	2.39064700	-0.88299900	-1.09515400

O	2.01269700	-1.97667700	-1.51026100
C	3.60086000	-0.79948000	-0.21358100
C	3.86054000	0.29827900	0.62286700
C	4.48060900	-1.89529700	-0.19521100
C	4.98031700	0.29978400	1.45566900
C	5.60423400	-1.88765400	0.62703700
C	5.85601700	-0.78884400	1.45577600
H	-1.82726000	3.75740900	2.24605200
H	-3.92501900	4.37031600	1.05043900
H	-4.93550800	2.81364400	-0.59425000
H	-3.86755700	0.61443300	-1.06419400
H	-0.25284100	2.20013900	3.67332700
H	1.13895800	1.29551100	3.01886800
H	0.72471900	2.90058200	2.35629300
H	-2.57585500	-2.94524400	2.25991200
H	-1.32181400	-1.66550400	2.69775300
H	-4.08281100	-4.22120200	-1.64604100
H	-4.73866200	-4.97578900	-0.15832400
H	-5.40637100	-3.42154500	-0.75183100
H	-1.46608100	-0.71237600	-1.86651900
H	-0.15064900	-1.52968900	-1.03256000
H	-1.32698800	1.74931600	-2.28285600
H	3.58433200	1.32555500	-1.96418200
H	0.11231100	3.56526400	-3.12255500
H	2.59759600	3.35810200	-2.95950400
H	3.18056300	1.14297700	0.63706800
H	4.27021400	-2.74530400	-0.83656600
H	5.16781800	1.15032400	2.10536000
H	6.28356000	-2.73586200	0.62496100
H	6.73096700	-0.78306700	2.1005090

F.3. Absolute configuration of compound 3g

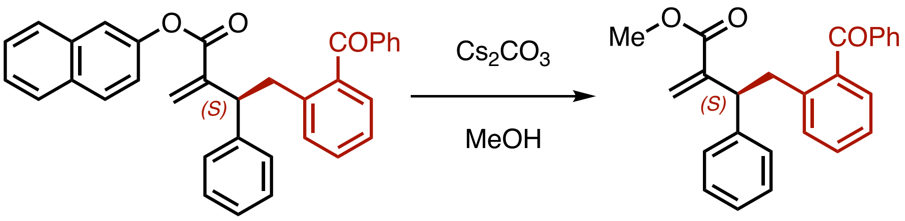

Figure S-47. Obtention of compound **3a** by trans-esterification of **3g**

All compounds **3** contain an aromatic ester moiety that could complicate the conformational analysis at a great extent, because of its conformational freedom. Moreover, the presence of a strong UV chromophore such as the 1- and 2-naphthyl rings makes more difficult the simulation of the ECD spectrum because different orientations of the naphthyl ring would lead to very different ECD spectra for different conformations. For this reason, the absolute configuration of compounds **3** was determined on compound **3a**, that was obtained by trans-esterification with MeOH starting from compound **3g**. The methyl ester largely reduces the conformational space to be explored in the conformational analysis step. Moreover, the phenyl ring directly bonded to the stereogenic carbon is C_2-symmetrical, and also this feature effectively reduces the number of conformations to be considered.

Conformational analysis

Table S-16. Results of DFT optimization for compound **3a**. The conformation number is that of the full conformational search.

	Opt. SMD-B3LYP/6-31G(d)		s.p. SMD-B3LYP/6-311++G(2d,2p)			
**Conf. #**	**G°(a.u.)**	**G_corr. (a.u.)**	**EE (a.u.)**	**EE+G_corr (a.u.)**	**Rel. G° (kcal/mol)**	Pop%
**3**	-1191.255095	0.347959	-1192.016710	-1191.668751	**0.000**	15
**10**	-1191.255842	0.347967	-1192.016528	-1191.668561	**0.119**	13
**12**	-1191.255270	0.348047	-1192.016591	-1191.668544	**0.130**	12
**33**	-1191.253948	0.347154	-1192.015475	-1191.668321	**0.270**	10
**11**	-1191.254083	0.348446	-1192.016545	-1191.668099	**0.409**	8
**38**	-1191.252943	0.347670	-1192.015618	-1191.667948	**0.504**	7
**25**	-1191.254238	0.348204	-1192.016060	-1191.667856	**0.562**	6
**13**	-1191.255174	0.347020	-1192.014534	-1191.667514	**0.776**	4
**7**	-1191.256004	0.348090	-1192.015383	-1191.667293	**0.915**	3
**5**	-1191.255499	0.346424	-1192.013695	-1191.667271	**0.929**	3
**14**	-1191.252940	0.348345	-1192.015560	-1191.667215	**0.964**	3
**9**	-1191.254339	0.347620	-1192.014546	-1191.666926	**1.145**	2
**35**	-1191.253588	0.346714	-1192.013590	-1191.666876	**1.177**	2
**18**	-1191.254013	0.348098	-1192.014970	-1191.666872	**1.179**	2
**4**	-1191.255034	0.348710	-1192.015505	-1191.666795	**1.227**	2
**26**	-1191.252263	0.348986	-1192.015732	-1191.666746	**1.258**	2
**19**	-1191.251873	0.348932	-1192.015597	-1191.666665	**1.309**	2
**23**	-1191.250733	0.348584	-1192.015140	-1191.666556	**1.377**	1
**8**	-1191.252262	0.347742	-1192.014242	-1191.666500	**1.413**	1
**31**	-1191.252976	0.348207	-1192.014691	-1191.666484	**1.423**	1

The conformational search was performed as described for compound **6a**, yielding 64 conformation within the 10 kcal/mol energy range. After optimization with SMD-B3LYP/6-31G(d) and single point calculation with GD3 correction at the SMD-B3LYP/6-311++G(2d,2p) level, 20 conformations were found to be enclosed in a 1.5 kcal/mol window (Table S-16 and Figure S-48).

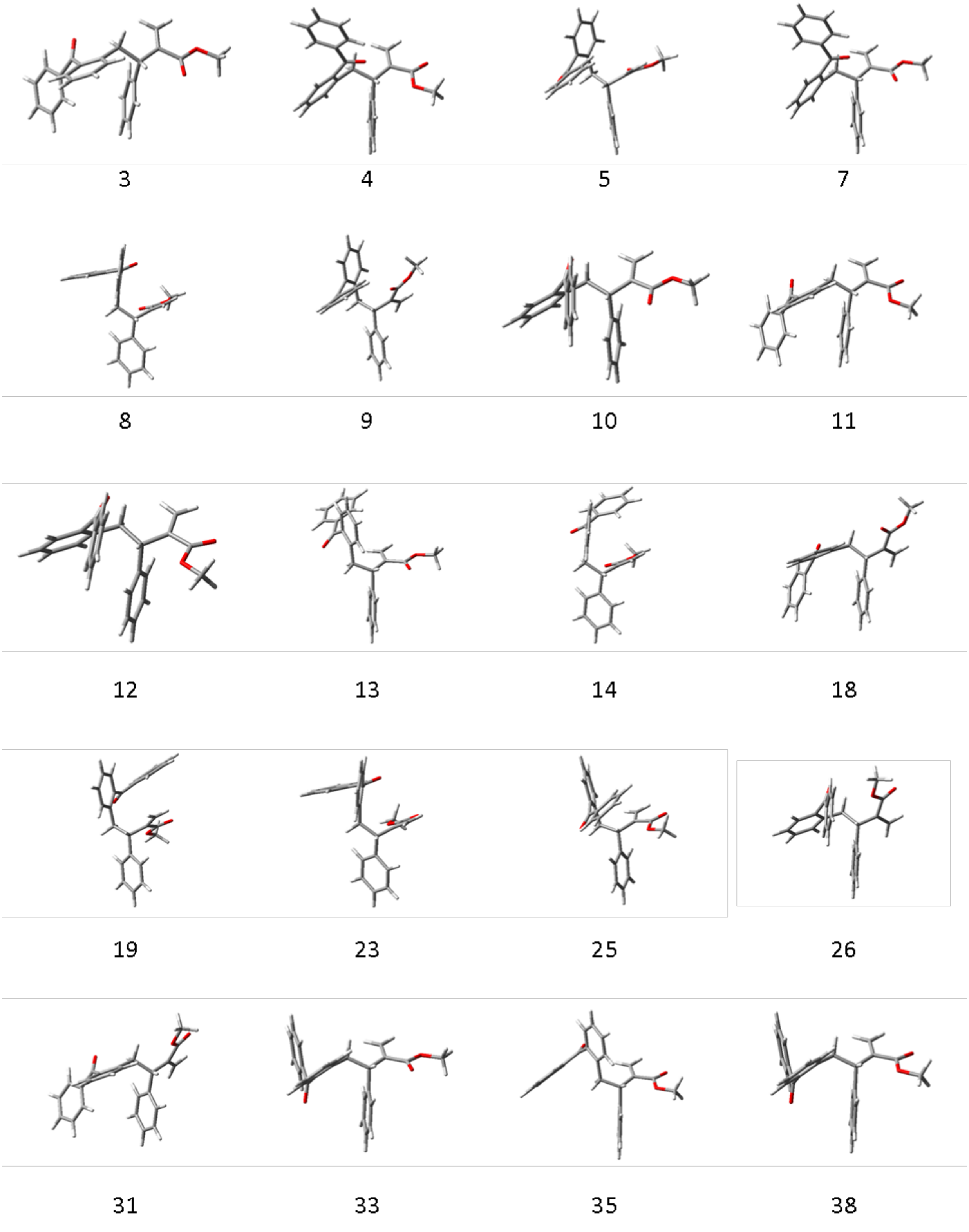

Figure S-48. The best 20 conformations of **3a**. Geometry optimization at the SMD-B3LYP/6-31G(d) level.

Absolute configuration

The large number of conformations to be considered is due to the presence of the tertiary stereogenic carbon that causes higher flexibility of **3a** with respect to **6a.** The choice to limit the energy range to 1.5 kcal/mol instead of the usual 3 kcal/mol range was taken because such a large number of conformations within 1.5 kcal/mol imply that the population of higher energy conformations is negligible (the populations of the first 1 kcal/mol range already sums up to 84%). The ECD spectrum of **3a** was recorded in acetonitrile, showing a shape similar to that of **6a** (Figure S-49). Two weak band were observed at 336 and 288 nm, while two intense bands were observed at 215 and 197 nm.

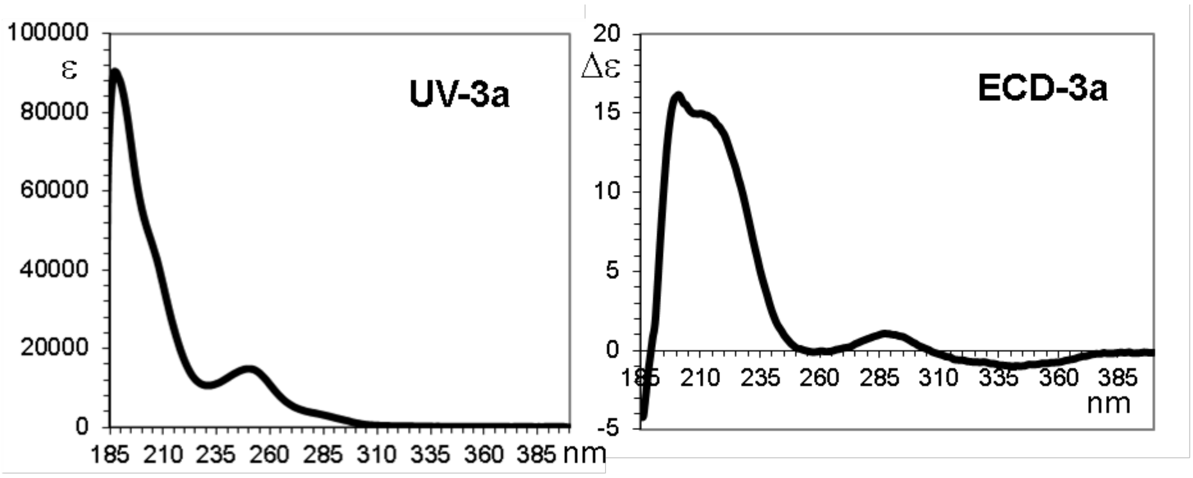

Figure S-49. UV and ECD spectra of **3a** in acetonitrile. The intensity of the ECD spectrum has been scaled to the specific value by considering that the sample had 60% ee.

The ECD calculated spectra for the 20 conformations were obtained with CAM-B3LYP and ωB97X-D functional with the 6-311++G(2d,p) basis set, by generating the first 50 UV/CD transitions and assuming the *S* absolute configuration (Figure S-50 and Figure S-51). Red shifts of 5 and 5 nm were applied to CAM-B3LYP and ωB97X-D, respectively. Vertical scale factors for ECD were 0.55 and 0.57, 0.88 and 0.85 for UV. The agreement with the experimental ECD spectrum is very good for both the calculation methods, and the assignment of the *S* absolute configuration to **3a** is reliable.

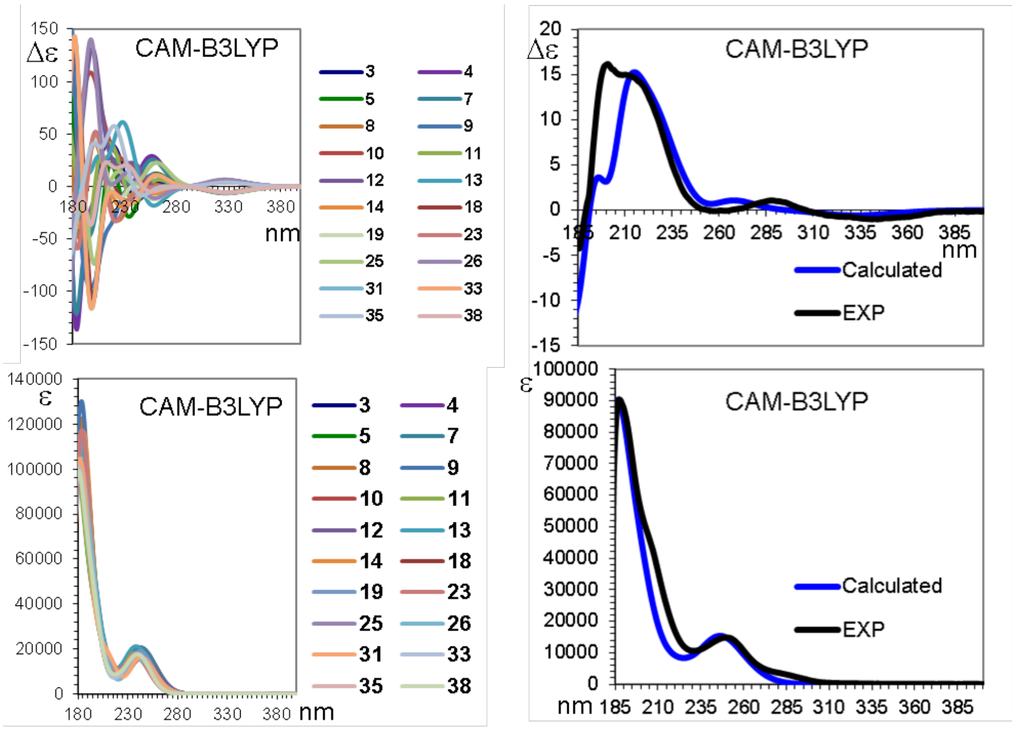

Figure S-50. Left: TD-DFT simulated spectra calculated for the 20 conformations of 3a (S absolute configuration) using CAM-B3LYP/6-311++G(2d,p). Right: comparison with the experimental spectra (black traces) after Boltzmann averaging.

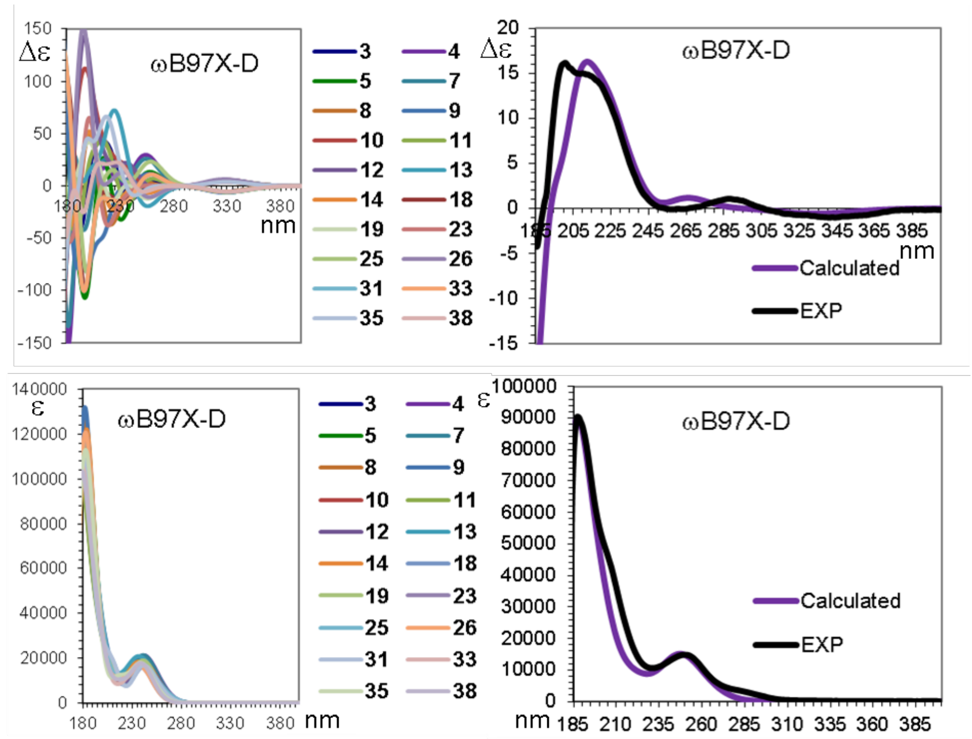

Figure S-51. Left: TD-DFT simulated spectra calculated for the 20 conformations of 3a (S absolute configuration) using ωB97X-D/6-311++G(2d,p). Right: comparison with the experimental spectra (black traces) after Boltzmann averaging.

Geometries of the optimized conformations

Compound 3a, conformation #3

0 1

C	1.82999800	0.60053500	0.19651100
C	3.04691600	0.15570800	0.99835000
C	3.09869400	0.05678900	2.33362100
C	4.25139000	-0.16509600	0.16600200
O	4.34015500	0.09011900	-1.02342900
O	5.23939500	-0.75898100	0.86228200
C	6.43980500	-1.05354500	0.12263000
C	0.80225400	1.38282700	1.05914800
C	-0.26663900	2.08349800	0.23776000
C	-1.63785300	1.73292700	0.25881700
C	0.13116900	3.12740300	-0.61171300
C	-2.54824700	2.42693900	-0.55991400
C	-0.77960100	3.82027200	-1.40828000
C	-2.13067000	3.47029700	-1.38214600
C	-2.19512100	0.71574700	1.21732300
O	-1.86974200	0.73482500	2.40336500
C	-3.18495600	-0.29878000	0.73083000
C	-3.28616800	-0.67279300	-0.61933700
C	-4.00242200	-0.93625900	1.68075400
C	-4.19079000	-1.66154900	-1.00954000
C	-4.91443500	-1.91142700	1.28777500
C	-5.00965200	-2.27684600	-0.05993800
C	1.19079400	-0.59032500	-0.52338300
C	0.99158400	-0.56062200	-1.91043800
C	0.39364800	-1.63613000	-2.57338800
C	-0.01380000	-2.76338200	-1.85604800
C	0.18019800	-2.80499500	-0.47239000
C	0.77797000	-1.72834400	0.18588700
H	2.20274200	1.27500700	-0.58065800
H	3.99948000	-0.27105700	2.84149000
H	2.24888600	0.30410100	2.96202900
H	6.22645600	-1.74476400	-0.69723500
H	6.88108300	-0.13695600	-0.27833100
H	7.11756300	-1.51664700	0.84071600
H	1.35344400	2.14514800	1.62409100
H	0.34691200	0.71792200	1.79219400
H	1.18160000	3.40771500	-0.63922800
H	-3.60021300	2.15965500	-0.52871600
H	-0.43470700	4.63198500	-2.04344500
H	-2.85324000	4.00505500	-1.99227500
H	-2.64623000	-0.20851400	-1.36209400
H	-3.91296000	-0.65035500	2.72402800
H	-4.25453900	-1.95126500	-2.05474700
H	-5.55034500	-2.38935300	2.02806300
H	-5.71853600	-3.04123600	-0.36697800
H	1.31134200	0.31068000	-2.47652200

H	0.25116700	-1.59339900	-3.65038700
H	-0.47549900	-3.60267700	-2.36967900
H	-0.13054700	-3.67834700	0.09564400
H	0.93329000	-1.77749300	1.26093900

Compound 3a, conformation #4

0 1

C	-1.42009900	-0.38387000	0.44980700
C	-2.00835500	-1.62340400	1.11478600
C	-1.89695300	-1.93056500	2.41413000
C	-2.78660100	-2.59037600	0.26719100
O	-3.43059900	-3.53231400	0.69645400
O	-2.68383700	-2.31487000	-1.04706600
C	-3.39418200	-3.19936500	-1.93459000
C	-0.30046500	0.26882400	1.31410800
C	0.48422200	1.35322100	0.60018800
C	1.67119400	1.10102300	-0.13262800
C	0.04606400	2.68194300	0.69873700
C	2.34876500	2.16490500	-0.75430100
C	0.71601800	3.72983900	0.06596700
C	1.86917800	3.47058000	-0.67519900
C	2.20543000	-0.28996500	-0.31948500
O	1.45110200	-1.22234400	-0.59470800
C	3.67639100	-0.54531500	-0.18319300
C	4.20829700	-1.69562900	-0.79210300
C	4.52217200	0.27791500	0.57862100
C	5.55925800	-2.00413700	-0.66148100
C	5.87304200	-0.04238100	0.72274500
C	6.39476500	-1.17773200	0.09857900
C	-2.52453500	0.60880600	0.07931500
C	-2.63140300	1.09631400	-1.23035800
C	-3.61807000	2.02427800	-1.57409200
C	-4.51831600	2.48080000	-0.60873900
C	-4.42404400	2.00071300	0.70070600
C	-3.43766600	1.07172800	1.03892600
H	-0.94447300	-0.70876400	-0.47772100
H	-2.35968200	-2.83115400	2.80618200
H	-1.35001700	-1.31036600	3.11611700
H	-3.01763500	-4.22148100	-1.84029400
H	-4.46535300	-3.18374600	-1.71651400
H	-3.20608700	-2.81747600	-2.93861100
H	0.37383600	-0.52742500	1.64029900
H	-0.74760500	0.70343800	2.21505200
H	-0.84178600	2.89627100	1.28728500
H	3.25206100	1.96062500	-1.32099300
H	0.33927200	4.74515300	0.15788500
H	2.39814800	4.27609800	-1.17676100
H	3.54827100	-2.33485900	-1.36999300
H	4.12377600	1.15698200	1.07438300
H	5.96328800	-2.88796200	-1.14765500
H	6.51673500	0.59462800	1.32307100

H	7.44861700	-1.42094600	0.20519300
H	-1.93551600	0.74435400	-1.98804700
H	-3.68299200	2.38817800	-2.59656100
H	-5.28807300	3.20100300	-0.87379800
H	-5.12167900	2.34641900	1.45959100
H	-3.38108000	0.69835700	2.05862800

Compound 3a, conformation #5

0 1

C	-1.02793800	-0.38429400	-0.03782600
C	-1.62854800	0.52593500	1.03396400
C	-1.00937800	0.72212900	2.20721100
C	-2.92503600	1.21847800	0.75072700
O	-3.42628300	1.32754400	-0.35740200
O	-3.49782700	1.73245400	1.85634200
C	-4.72420100	2.45995400	1.64996000
C	-0.66651000	0.34108100	-1.36886500
C	0.33443300	1.46861500	-1.19798300
C	1.72371300	1.25349600	-1.02135900
C	-0.13381600	2.78942400	-1.17045300
C	2.58294800	2.35022100	-0.84012100
C	0.72799300	3.87529300	-1.00319800
C	2.09582600	3.65687200	-0.84237000
C	2.33986400	-0.11651400	-1.12542500
O	2.05396800	-0.85785300	-2.06378700
C	3.32901600	-0.56590300	-0.09272700
C	3.41545300	0.02034300	1.18112400
C	4.15953400	-1.65792300	-0.40091900
C	4.31674300	-0.47557200	2.12463300
C	5.06740400	-2.14208600	0.53615500
C	5.14668000	-1.55183700	1.80285300
C	-1.88265900	-1.63663400	-0.25957300
C	-2.93287300	-1.69491800	-1.18809400
C	-3.67986000	-2.86511500	-1.34977900
C	-3.39437700	-3.99858600	-0.58547600
C	-2.35225400	-3.95340200	0.34393900
C	-1.60701800	-2.78351400	0.50176000
H	-0.08459600	-0.73751900	0.39275700
H	-1.42620800	1.36042300	2.97894200
H	-0.05628400	0.24580300	2.42314600
H	-4.55929100	3.31550800	0.98937900
H	-5.48957900	1.80873400	1.21952400
H	-5.02997500	2.79988100	2.64003400
H	-1.57275300	0.74861400	-1.81832000
H	-0.26468100	-0.41176800	-2.04979300
H	-1.19981000	2.96600600	-1.28885600
H	3.64831000	2.17648900	-0.71972100
H	0.32928400	4.88639000	-1.00024900
H	2.77964800	4.49194200	-0.71816500
H	2.77064100	0.85294600	1.44187700
H	4.08160600	-2.11297400	-1.38320600

H	4.36964600	-0.02139000	3.11020600
H	5.71220100	-2.97966900	0.28430200
H	5.85230200	-1.93264100	2.53646400
H	-3.17743700	-0.82305600	-1.78443700
H	-4.48774900	-2.88845000	-2.07716400
H	-3.97565400	-4.90798400	-0.71451200
H	-2.11509700	-4.82909100	0.94306700
H	-0.79422800	-2.75860200	1.22455400

Compound 3a, conformation #7

0 1

C	1.41387200	0.26644100	0.30611600
C	2.08547400	1.51590200	0.86264000
C	2.04039500	1.91587300	2.14073700
C	2.82364000	2.32716300	-0.15929700
O	2.74181900	2.14007400	-1.36160100
O	3.58750800	3.29771500	0.37893600
C	4.28826000	4.13709600	-0.55842700
C	0.28499900	-0.26326200	1.23706800
C	-0.57598800	-1.34680000	0.61545200
C	-1.77030800	-1.07808500	-0.09931800
C	-0.20346000	-2.68812300	0.78664500
C	-2.52000300	-2.14138700	-0.63307800
C	-0.94487900	-3.73643800	0.24022400
C	-2.10545800	-3.46312400	-0.48410700
C	-2.23753800	0.32512800	-0.36006400
O	-1.44414900	1.19759900	-0.71118100
C	-3.68913900	0.66623600	-0.20419300
C	-4.55435300	-0.06136500	0.62965000
C	-4.17800900	1.80296500	-0.87184400
C	-5.88221400	0.33906000	0.78699100
C	-5.50691500	2.19082900	-0.72723500
C	-6.36209700	1.45942300	0.10489400
C	2.45750300	-0.80393400	-0.02137300
C	2.52272700	-1.36350500	-1.30465400
C	3.45544000	-2.35879900	-1.60884000
C	4.34254600	-2.81154000	-0.62976300
C	4.28975700	-2.25988100	0.65364800
C	3.35743000	-1.26399100	0.95217700
H	0.93921000	0.55824400	-0.63320000
H	2.54594200	2.81773300	2.46910800
H	1.49388000	1.36221700	2.89781400
H	4.83948900	4.85535400	0.04972000
H	4.97902800	3.54416300	-1.16400500
H	3.58354400	4.65711800	-1.21315200
H	-0.33583800	0.58925900	1.52469900
H	0.73095800	-0.66239100	2.15496300
H	0.68994200	-2.91178200	1.36319200
H	-3.42863400	-1.92551900	-1.18679200
H	-0.61770800	-4.76258200	0.38602000
H	-2.68993600	-4.26909700	-0.91890500

H	-4.18803800	-0.92762900	1.17060700
H	-3.50257900	2.36864500	-1.50586200
H	-6.54064200	-0.22394100	1.44276600
H	-5.87822600	3.06292100	-1.25862800
H	-7.39848400	1.76478400	0.22222800
H	1.83695100	-1.01519700	-2.07307000
H	3.48863500	-2.77782500	-2.61152900
H	5.06994500	-3.58453300	-0.86397200
H	4.97771100	-2.60215300	1.42286400
H	3.33333400	-0.83618100	1.95162300

Compound 3a, conformation #8

0 1

C	1.54846100	0.98569700	-0.37423500
C	1.43771500	0.97666300	1.14868000
C	1.53407100	2.10371000	1.86835100
C	1.19876600	-0.33744200	1.82410400
O	0.93521400	-1.37361100	1.23653700
O	1.31209000	-0.26911700	3.16472600
C	1.05255300	-1.49078000	3.88085300
C	0.21009700	0.63546900	-1.08705000
C	-0.87794300	1.66464900	-0.83886000
C	-1.99047800	1.42848500	-0.00527400
C	-0.75452500	2.93418500	-1.42734300
C	-2.91628600	2.45606500	0.24316400
C	-1.68943200	3.94273300	-1.20095900
C	-2.77670500	3.70523400	-0.35601200
C	-2.24750100	0.11827900	0.70266500
O	-2.22427200	0.08722800	1.93011600
C	-2.61316200	-1.09761900	-0.08703900
C	-2.69014800	-2.33324900	0.57935800
C	-2.92154500	-1.03612900	-1.45586700
C	-3.05744800	-3.48440600	-0.11074300
C	-3.29686900	-2.19017400	-2.14467400
C	-3.36249600	-3.41449200	-1.47517900
C	2.74116300	0.15154500	-0.85614100
C	2.60925700	-1.07613700	-1.51829400
C	3.73671700	-1.78411500	-1.94595100
C	5.01775900	-1.27922400	-1.71606600
C	5.16352700	-0.05620500	-1.05522400
C	4.03555600	0.64831800	-0.63270100
H	1.78005100	2.02388900	-0.63847300
H	1.45194100	2.10384900	2.95010400
H	1.69245100	3.06443000	1.38498300
H	1.20571000	-1.24875800	4.93325800
H	0.02411500	-1.82252300	3.71450500
H	1.74513100	-2.27672700	3.56795400
H	-0.11355000	-0.35969300	-0.78220000
H	0.41414100	0.59348100	-2.16373000
H	0.09651100	3.12960000	-2.07613000
H	-3.75834800	2.26021100	0.90154900

H	-1.56825800	4.91089900	-1.67973300
H	-3.50934500	4.48499800	-0.16672900
H	-2.45493600	-2.37189600	1.63796300
H	-2.87831100	-0.08707700	-1.98044300
H	-3.10768400	-4.43669800	0.41013300
H	-3.53912600	-2.13281200	-3.20223200
H	-3.65143800	-4.31323000	-2.01363600
H	1.62480300	-1.49227200	-1.70321100
H	3.60933800	-2.73411800	-2.45922400
H	5.89318800	-1.83071300	-2.04924300
H	6.15452800	0.35213900	-0.87289200
H	4.15820500	1.60065600	-0.12099000

Compound 3a, conformation #9

0 1

C	-1.53209700	0.01857000	-0.14060600
C	-1.86833400	1.33184300	0.57112700
C	-3.02566900	1.98881600	0.41855200
C	-0.81128000	1.86651100	1.48724700
O	0.13998800	1.21346700	1.88587700
O	-1.01829100	3.14662500	1.84662100
C	-0.05874400	3.70579900	2.76458600
C	-1.03170100	0.28455000	-1.59340300
C	0.25927100	1.07861000	-1.66413700
C	1.53375100	0.49929600	-1.44523400
C	0.19714300	2.45663200	-1.91136300
C	2.68245500	1.30637100	-1.48367200
C	1.34568700	3.25023400	-1.96114200
C	2.59692200	2.67288800	-1.74975500
C	1.72333800	-0.98441600	-1.27355200
O	1.16664400	-1.77818800	-2.02995100
C	2.63400600	-1.49072100	-0.19716300
C	3.17657600	-2.78018500	-0.33594200
C	2.91387700	-0.74883100	0.96244200
C	4.00147800	-3.30874300	0.65273100
C	3.72759900	-1.28845500	1.96019000
C	4.27817100	-2.56266200	1.80423600
C	-2.68269900	-0.98346600	-0.08761500
C	-2.79807100	-1.82231700	1.03284500
C	-3.83806500	-2.74632000	1.13943500
C	-4.78844500	-2.85298000	0.11968200
C	-4.68483500	-2.02811900	-1.00145500
C	-3.64100200	-1.10278900	-1.10459500
H	-0.70029700	-0.42470900	0.41453100
H	-3.22148800	2.92727100	0.92599100
H	-3.81937100	1.59649300	-0.21171500
H	-0.04925600	3.14461500	3.70286000
H	0.94189800	3.69914300	2.32426400
H	-0.38787500	4.73060500	2.94002600
H	-1.80477500	0.83540800	-2.13873000
H	-0.90114500	-0.67920400	-2.08864800

H	-0.77544700	2.91418800	-2.07372900
H	3.65624200	0.85222500	-1.32599200
H	1.25891100	4.31397000	-2.16659100
H	3.49951800	3.27618700	-1.79085000
H	2.94650500	-3.35215300	-1.22940100
H	2.47128600	0.23192000	1.09953200
H	4.42789100	-4.30077000	0.53073500
H	3.93005300	-0.71385300	2.85994300
H	4.91861500	-2.97664300	2.57862400
H	-2.06036800	-1.74788600	1.82909400
H	-3.90259000	-3.38692500	2.01550700
H	-5.59802800	-3.57406800	0.19753600
H	-5.41486900	-2.10400000	-1.80357300
H	-3.57813200	-0.47774400	-1.99041700

Compound 3a, conformation #10

0 1

C	1.22475800	0.01019900	-0.61395800
C	2.20936600	-1.01547300	-1.16237000
C	2.78200300	-0.96564800	-2.37263500
C	2.50371900	-2.15101500	-0.22953600
O	1.87252000	-2.37050600	0.79074200
O	3.53495100	-2.91840800	-0.63133700
C	3.84070300	-4.04932200	0.20674700
C	0.57805700	0.87200700	-1.73381400
C	-0.56561800	1.74785200	-1.25685000
C	-1.84734200	1.23679000	-0.93209900
C	-0.34912700	3.12326800	-1.10189600
C	-2.85330200	2.10942300	-0.48466800
C	-1.35757500	3.98593800	-0.66680100
C	-2.61979500	3.47880800	-0.36157200
C	-2.20957200	-0.20800500	-1.15005200
O	-1.91165800	-0.77405400	-2.20032800
C	-2.96566600	-0.95190900	-0.09080500
C	-3.63714100	-2.13073700	-0.46023400
C	-2.97872900	-0.55127500	1.25572800
C	-4.32147400	-2.88202500	0.49063500
C	-3.65276500	-1.31426000	2.21067900
C	-4.32916100	-2.47533100	1.82988400
C	1.88564400	0.87187200	0.46582000
C	1.33556000	0.95028600	1.75211200
C	1.91769800	1.75604300	2.73453000
C	3.06559500	2.49668800	2.44476900
C	3.62635900	2.42367400	1.16631700
C	3.04201700	1.61659400	0.18818900
H	0.42941600	-0.55768900	-0.12036400
H	3.48039000	-1.72883700	-2.69901100
H	2.56810900	-0.16845800	-3.07747800
H	4.68614000	-4.54529800	-0.27144700
H	4.11418900	-3.71958300	1.21271300
H	2.98621100	-4.72907000	0.26552200

H	0.22089000	0.19981600	-2.51664300
H	1.34241400	1.51716700	-2.17893300
H	0.63311200	3.52600700	-1.33536800
H	-3.83720000	1.71228900	-0.25334400
H	-1.15419000	5.04915900	-0.56964700
H	-3.41664500	4.13824400	-0.02916500
H	-3.61537000	-2.44089800	-1.50009800
H	-2.45128700	0.34605400	1.56183600
H	-4.84742900	-3.78502700	0.19272300
H	-3.64860900	-1.00139500	3.25109100
H	-4.85947700	-3.06421200	2.57358400
H	0.44583100	0.37163200	1.98760900
H	1.47446800	1.80168000	3.72624200
H	3.52134100	3.12264600	3.20763800
H	4.52241900	2.99256000	0.93064100
H	3.49350500	1.56032000	-0.79944700

Compound 3a, conformation #11

0 1

C	1.88657300	0.51349300	0.39587100
C	3.01282300	0.04320300	1.30998100
C	2.95336800	-0.00087700	2.64773100
C	4.29723200	-0.42302100	0.68445000
O	5.20906800	-0.96438600	1.28548400
O	4.34723400	-0.15980400	-0.63557500
C	5.55040900	-0.56768900	-1.31484100
C	0.82346700	1.35446200	1.15745000
C	-0.16106000	2.05985600	0.24044400
C	-1.54262800	1.75677900	0.18044800
C	0.33002200	3.05728700	-0.61591500
C	-2.37004200	2.44896400	-0.72348100
C	-0.49942300	3.74895200	-1.49789800
C	-1.86062800	3.44498300	-1.55267500
C	-2.19983600	0.79941600	1.13729000
O	-1.94916700	0.85256700	2.34034300
C	-3.19819700	-0.19466500	0.62644700
C	-3.22919200	-0.61935300	-0.71210300
C	-4.09975500	-0.76052300	1.54511300
C	-4.14707400	-1.58777800	-1.12183500
C	-5.02441400	-1.71505200	1.13158900
C	-5.04895400	-2.13159500	-0.20437200
C	1.25516400	-0.66863600	-0.34547100
C	1.14269700	-0.65545700	-1.74255800
C	0.54896700	-1.72061200	-2.42546500
C	0.05799900	-2.82080500	-1.71833300
C	0.16459800	-2.84550700	-0.32491700
C	0.75868100	-1.77918200	0.35341100
H	2.33865500	1.15945100	-0.36290100
H	3.80051900	-0.36384800	3.22177000
H	2.07886100	0.31743500	3.20479500
H	6.41966800	-0.05089100	-0.89923200

H	5.69265100	-1.64816400	-1.22847800
H	5.40534900	-0.28658000	-2.35844100
H	1.35670700	2.11740800	1.73854600
H	0.29607400	0.72617400	1.87399100
H	1.38891400	3.30245100	-0.58044500
H	-3.43067400	2.21897600	-0.75540000
H	-0.08356700	4.52394400	-2.13641700
H	-2.52067600	3.97925900	-2.23031300
H	-2.52525600	-0.21048000	-1.42910500
H	-4.06478200	-0.43558600	2.58014700
H	-4.15640000	-1.91752300	-2.15699800
H	-5.72513700	-2.13719200	1.84684000
H	-5.76786400	-2.87994700	-0.52721400
H	1.52742900	0.19456800	-2.30060800
H	0.47499900	-1.69120600	-3.50977300
H	-0.40067000	-3.65220700	-2.24725800
H	-0.21172700	-3.69761000	0.23565100
H	0.84506400	-1.81531200	1.43665700

Compound 3a, conformation #12

0 1

C	1.26686400	-0.15346300	-0.73181900
C	2.08682200	-1.24110000	-1.41756800
C	2.60230100	-1.15530000	-2.65112800
C	2.36021900	-2.51462300	-0.66822500
O	3.12965600	-3.38669300	-1.03324000
O	1.63817300	-2.60465500	0.46508300
C	1.83603800	-3.79902700	1.24592700
C	0.72778300	0.90116600	-1.74023100
C	-0.26399400	1.88042600	-1.14070900
C	-1.59964700	1.53032500	-0.81923100
C	0.15593700	3.18833600	-0.86554000
C	-2.45600800	2.49142200	-0.25631300
C	-0.70481000	4.14092800	-0.31596000
C	-2.02093200	3.79405400	-0.01438300
C	-2.17504000	0.18134500	-1.15902100
O	-1.98033000	-0.31904500	-2.26532400
C	-3.01604800	-0.54383100	-0.15160000
C	-3.86339800	-1.56765300	-0.61072100
C	-2.94492300	-0.28251600	1.22700900
C	-4.63596800	-2.30090100	0.28507500
C	-3.70886600	-1.02936300	2.12541600
C	-4.55877900	-2.03366800	1.65678700
C	2.06277800	0.49921800	0.40229600
C	1.54082700	0.55318200	1.70163300
C	2.24721700	1.17567800	2.73429000
C	3.49322900	1.75413400	2.48250900
C	4.02622900	1.70364600	1.19130800
C	3.31734900	1.07984200	0.16255500
H	0.40329500	-0.64457900	-0.27200700
H	3.18735600	-1.97428900	-3.05829100

H	2.45923600	-0.28691800	-3.28492700
H	1.55611200	-4.68509500	0.66992000
H	2.87956200	-3.88297100	1.56093600
H	1.18537600	-3.69051500	2.11418100
H	0.25822400	0.36937800	-2.57010300
H	1.56888800	1.47072600	-2.14836500
H	1.18134600	3.46619700	-1.09543700
H	-3.48250100	2.22058100	-0.02815900
H	-0.34531800	5.14917300	-0.12773700
H	-2.70474100	4.52609300	0.40614000
H	-3.90689700	-1.77216700	-1.67577000
H	-2.28368000	0.49192600	1.60131900
H	-5.29665700	-3.08191000	-0.08137700
H	-3.63945300	-0.82607000	3.19046300
H	-5.15833900	-2.60912900	2.35712100
H	0.57429600	0.09970500	1.90732100
H	1.82373500	1.20560600	3.73518600
H	4.04535900	2.23736700	3.28442700
H	4.99703400	2.14731400	0.98448900
H	3.74670500	1.03918300	-0.83562700

Compound 3a, conformation #13

0 1

C	1.75742100	-0.04398300	-0.52829100
C	1.34144100	0.82577500	0.65082600
C	0.28303100	0.60175500	1.44105600
C	2.24098300	1.99798600	0.90183300
O	3.14740800	2.32929200	0.15547700
O	1.95101000	2.66132300	2.03742000
C	2.76631700	3.81499800	2.31771900
C	0.59559600	-0.84282800	-1.18195800
C	-0.47252700	0.01552600	-1.84530500
C	-1.85326400	-0.08159900	-1.54604900
C	-0.07811100	0.95979500	-2.80556000
C	-2.77249300	0.76511200	-2.19236800
C	-0.99757600	1.78259100	-3.45567000
C	-2.35543200	1.68801600	-3.14800900
C	-2.40492100	-1.15238900	-0.64539400
O	-2.03664300	-2.31899700	-0.77274000
C	-3.43408600	-0.80024100	0.38525700
C	-4.23669000	-1.83193100	0.90304100
C	-3.58342500	0.50224300	0.89034300
C	-5.18157500	-1.56420800	1.88928700
C	-4.52055700	0.76572900	1.89069000
C	-5.32437600	-0.26352400	2.38620200
C	2.89337100	-0.99648300	-0.14039600
C	4.05654700	-1.05622600	-0.92082900
C	5.08550000	-1.94870300	-0.60992600
C	4.96655300	-2.79933500	0.49160200
C	3.81199200	-2.74901700	1.27749600
C	2.78661500	-1.85391900	0.96441500

H	2.17121300	0.63260900	-1.28043600
H	0.05350400	1.25096100	2.27919000
H	-0.38505000	-0.23704300	1.27899600
H	2.66558000	4.56080800	1.52435600
H	2.39105000	4.21377100	3.26090900
H	3.81682700	3.52914600	2.41850300
H	0.15338700	-1.52149400	-0.45239800
H	1.04725300	-1.48410600	-1.94970200
H	0.97506600	1.04688700	-3.05928200
H	-3.82948400	0.67700200	-1.95968200
H	-0.65173100	2.49349700	-4.20150500
H	-3.08291400	2.32085900	-3.64856300
H	-4.10962900	-2.83805400	0.51605600
H	-2.95714000	1.30539200	0.51596800
H	-5.80614000	-2.36581700	2.27412800
H	-4.62209300	1.77415400	2.28213300
H	-6.05888900	-0.05529800	3.15961300
H	4.15727600	-0.39619700	-1.77944900
H	5.98031600	-1.97577500	-1.22685600
H	5.76666500	-3.49276200	0.73741500
H	3.70983100	-3.40500600	2.13848200
H	1.89889000	-1.81732800	1.59079900

Compound 3a, conformation #14

0 1

C	-1.85856900	0.73990300	-0.36747000
C	-1.06142400	-0.24593900	-1.21826800
C	-0.71679400	0.03375100	-2.48380100
C	-0.65585900	-1.54267100	-0.59007500
O	-0.80289700	-1.80905500	0.59091300
O	-0.10506000	-2.40520200	-1.46738300
C	0.33063500	-3.66464500	-0.92140200
C	-1.03169800	1.34327600	0.80441800
C	0.12616100	2.20313400	0.32849600
C	1.48411200	1.83800800	0.47388800
C	-0.16200900	3.40848200	-0.33017500
C	2.49344400	2.66801400	-0.04614800
C	0.84368100	4.23794300	-0.82538700
C	2.18205600	3.86643300	-0.68426400
C	1.91243500	0.64237200	1.28370600
O	1.49438900	0.49461000	2.42980900
C	2.88285500	-0.33721000	0.70011500
C	3.11195300	-0.43914100	-0.68218100
C	3.54667200	-1.21843100	1.57194800
C	3.99251300	-1.40111200	-1.18006800
C	4.43344000	-2.16888300	1.07469700
C	4.65773700	-2.26206700	-0.30406300
C	-3.20517000	0.14893800	0.06637600
C	-4.20369400	-0.01939800	-0.90664500
C	-5.45594100	-0.53871200	-0.57573800
C	-5.73643800	-0.90094500	0.74480500

C	-4.75314500	-0.73810600	1.72242000
C	-3.49865900	-0.22044700	1.38570000
H	-2.09884200	1.56910300	-1.04238300
H	-0.14941700	-0.66199000	-3.09281200
H	-0.99645000	0.97799200	-2.94407400
H	0.74183100	-4.21996000	-1.76520900
H	1.09871800	-3.51045800	-0.15903500
H	-0.51247400	-4.20756500	-0.48567900
H	-0.68191800	0.54983900	1.46159900
H	-1.71144200	1.97338900	1.39128700
H	-1.20204700	3.70395300	-0.44900800
H	3.53365700	2.37937700	0.07510800
H	0.58215800	5.17033300	-1.31893900
H	2.97622200	4.50364600	-1.06333100
H	2.59100700	0.21938100	-1.36932400
H	3.35753800	-1.14027000	2.63790000
H	4.15815900	-1.47791800	-2.25111300
H	4.94999500	-2.83856800	1.75692300
H	5.34800600	-3.00599900	-0.69292300
H	-3.99481500	0.26243600	-1.93666400
H	-6.21276500	-0.65580200	-1.34750400
H	-6.71118700	-1.30339300	1.00833500
H	-4.95850400	-1.01577100	2.75340400
H	-2.74863800	-0.10985700	2.16143600

Compound 3a, conformation #18

0 1

C	-1.85918900	-0.31509900	0.27775500
C	-3.06787300	-0.85791600	-0.48522200
C	-3.13281700	-2.04159600	-1.10821800
C	-4.25740800	0.05575300	-0.47444500
O	-4.33436600	1.06172400	0.21342400
O	-5.24682000	-0.34275500	-1.29480800
C	-6.42958100	0.48015800	-1.30167000
C	-1.17976200	0.81567900	-0.55839200
C	-0.23624800	1.68814700	0.25270400
C	1.16055800	1.76791000	0.03582900
C	-0.78363100	2.44737800	1.29863300
C	1.94841100	2.58859200	0.86513100
C	0.00241400	3.26883200	2.10535400
C	1.37941100	3.34231000	1.88815000
C	1.83369000	1.10245800	-1.13278800
O	1.33018700	1.15286100	-2.25408400
C	3.15017900	0.41156700	-0.93832600
C	3.96640600	0.21094500	-2.06524600
C	3.56682900	-0.09054200	0.30574800
C	5.18196100	-0.45712500	-1.94783400
C	4.77881300	-0.77359100	0.41875600
C	5.59035800	-0.95191600	-0.70405400
C	-0.88962600	-1.39322200	0.74255300
C	0.03881800	-1.99915800	-0.11902600

C	0.90151900	-2.99720500	0.34273100
C	0.85556500	-3.40709900	1.67733500
C	-0.06042500	-2.81023100	2.54682500
C	-0.92201300	-1.81492500	2.08052300
H	-2.26872500	0.15748500	1.17584400
H	-4.02795900	-2.35492000	-1.63448100
H	-2.29816900	-2.73533100	-1.10290700
H	-6.88683200	0.50633500	-0.30886000
H	-6.18787100	1.49785100	-1.61972000
H	-7.10555500	0.01054600	-2.01706100
H	-1.97496800	1.45829400	-0.95295000
H	-0.67538200	0.38416000	-1.42153100
H	-1.85607300	2.39965500	1.47104600
H	3.01686400	2.65520500	0.68434000
H	-0.46060100	3.85187100	2.89716600
H	2.00418900	3.98267200	2.50449600
H	3.63451800	0.59125200	-3.02618100
H	2.93806900	0.03472700	1.18096300
H	5.81162300	-0.59543100	-2.82259500
H	5.08786700	-1.16669900	1.38334800
H	6.53735600	-1.47717900	-0.61236600
H	0.09493400	-1.69268300	-1.15965100
H	1.61223200	-3.45153700	-0.34294000
H	1.52902300	-4.18095100	2.03651800
H	-0.10370900	-3.11599700	3.58919700
H	-1.63101000	-1.35458400	2.76521500

Compound 3a, conformation #19

0 1

C	1.79614100	0.79986200	0.37918800
C	1.00147400	-0.10538500	1.31864600
C	0.61521800	0.30836200	2.53413000
C	0.61778600	-1.50634900	0.93662300
O	0.08984600	-2.30923400	1.68669600
O	0.91777700	-1.79188800	-0.34351800
C	0.57508000	-3.11556500	-0.79644200
C	0.99241900	1.25530600	-0.87275600
C	-0.20689800	2.11979800	-0.52587600
C	-1.54877000	1.67981700	-0.61311400
C	0.02818100	3.41977300	-0.05247500
C	-2.59293600	2.54018600	-0.22704600
C	-1.01253100	4.27283400	0.31332000
C	-2.33408100	3.83229900	0.22399000
C	-1.92491400	0.35326200	-1.21788600
O	-1.45052100	0.00580300	-2.29748300
C	-2.91730400	-0.52390700	-0.51696300
C	-3.18352300	-0.41650900	0.85821800
C	-3.56180000	-1.52658400	-1.26326500
C	-4.08084600	-1.29234100	1.47140300
C	-4.46642800	-2.39033400	-0.65256300
C	-4.72718600	-2.27473700	0.71776900

C	3.17354900	0.20890300	0.05093200
C	4.11086800	0.08577500	1.08963500
C	5.38833300	-0.42369200	0.85461200
C	5.75735400	-0.82218700	-0.43343800
C	4.83607500	-0.70455800	-1.47542900
C	3.55594900	-0.19549200	-1.23487100
H	1.99410700	1.70539300	0.96481700
H	0.05624400	-0.34603600	3.19620600
H	0.84843800	1.30645700	2.89427300
H	0.90355600	-3.16136100	-1.83508700
H	1.09328000	-3.87116900	-0.20015700
H	-0.50421900	-3.27563300	-0.73227500
H	0.69084400	0.39415200	-1.46475800
H	1.67252100	1.85156400	-1.49307200
H	1.05509000	3.77055100	0.02161200
H	-3.62041700	2.19729900	-0.30618700
H	-0.79117800	5.27778700	0.66311600
H	-3.15595900	4.48712900	0.49977500
H	-2.67666200	0.33644200	1.45267300
H	-3.34561900	-1.60955500	-2.32370700
H	-4.27347100	-1.20787300	2.53734900
H	-4.96846200	-3.15430700	-1.23998100
H	-5.43064700	-2.95136000	1.19569600
H	3.83352300	0.39473100	2.09525100
H	6.09588600	-0.50562700	1.67605900
H	6.75206700	-1.21787400	-0.62176100
H	5.10992200	-1.01033800	-2.48225700
H	2.85709700	-0.11906200	-2.06120000

Compound 3a, conformation #23

0 1

C	1.59306200	0.97142000	-0.45985800
C	1.54455100	1.30202600	1.03099000
C	1.71531400	2.55450000	1.47706200
C	1.31874800	0.24674100	2.07454100
O	1.41101300	0.43073800	3.27562600
O	1.00073600	-0.94839200	1.54215200
C	0.76349500	-2.01255800	2.48376100
C	0.22279200	0.53358400	-1.05076200
C	-0.85705100	1.59024900	-0.90103900
C	-1.97185800	1.42940400	-0.05332400
C	-0.73130400	2.80127000	-1.60107500
C	-2.89615200	2.47570300	0.10361800
C	-1.66415000	3.82783500	-1.46354000
C	-2.75147100	3.66841300	-0.60057200
C	-2.23412800	0.18034100	0.75705700
O	-2.18758000	0.23832100	1.98276100
C	-2.63195600	-1.08422600	0.06410600
C	-2.76568500	-2.25637700	0.82956900
C	-2.91170200	-1.12856600	-1.31164100
C	-3.15755100	-3.44942200	0.22966100

C	-3.31019800	-2.32431900	-1.91029300
C	-3.43051500	-3.48533000	-1.14282700
C	2.74416700	0.01284800	-0.79234200
C	2.55413400	-1.27673400	-1.30588200
C	3.64650100	-2.09434300	-1.61337200
C	4.94989300	-1.63866500	-1.41015600
C	5.15371500	-0.35430400	-0.89720900
C	4.06135400	0.45859500	-0.59367000
H	1.84620800	1.92037700	-0.94705200
H	1.68596500	2.77872000	2.53905000
H	1.88486900	3.38227800	0.79432900
H	0.49731500	-2.88126400	1.88131000
H	1.66754300	-2.21709500	3.06395200
H	-0.05283200	-1.74966900	3.16028500
H	-0.09058100	-0.40582400	-0.59539600
H	0.37881100	0.33485800	-2.11826200
H	0.11878300	2.93721700	-2.26591000
H	-3.74027900	2.33959300	0.77437500
H	-1.54082000	4.75011100	-2.02516900
H	-3.48132400	4.46420400	-0.47937000
H	-2.55933600	-2.21248400	1.89396500
H	-2.82735600	-0.22906800	-1.91275100
H	-3.25319200	-4.35173200	0.82745500
H	-3.52855800	-2.34856700	-2.97428600
H	-3.73859200	-4.41644000	-1.61101900
H	1.55152700	-1.65684400	-1.47134600
H	3.47347800	-3.09092600	-2.01217300
H	5.79787800	-2.27497600	-1.64991700
H	6.16307500	0.01657600	-0.73693500
H	4.23004100	1.45712600	-0.19626800

Compound 3a, conformation #25

0 1

C	-1.28619600	0.50120200	-0.06539200
C	-1.48266900	0.85873000	1.40217700
C	-1.77490600	2.07936100	1.87062100
C	-1.38178200	-0.23706400	2.42591100
O	-1.58147000	-0.09603700	3.62023700
O	-1.03345900	-1.41854200	1.87975700
C	-0.91127500	-2.52453900	2.79463400
C	-0.81690800	1.68106500	-0.96716100
C	0.55780300	2.23861200	-0.64003800
C	1.74831100	1.48120100	-0.77618100
C	0.66987100	3.55431000	-0.17238200
C	2.98496100	2.05502600	-0.43803500
C	1.90510700	4.12496800	0.14326600
C	3.07109300	3.37276200	0.01051600
C	1.75386600	0.10789200	-1.39222700
O	1.13375900	-0.10660600	-2.43269500
C	2.54440500	-0.99151900	-0.75151300
C	2.90257200	-0.97108700	0.60671900

C	2.88190000	-2.11087200	-1.53254100
C	3.58813800	-2.04877300	1.16956400
C	3.57946300	-3.17744900	-0.97332000
C	3.93265000	-3.14882400	0.38080100
C	-2.54087700	-0.14060600	-0.66765800
C	-2.41201500	-1.25843200	-1.50558500
C	-3.53083500	-1.83604700	-2.10909100
C	-4.80281000	-1.30241000	-1.88465800
C	-4.94321200	-0.18784600	-1.05452400
C	-3.82142800	0.38658900	-0.45074100
H	-0.50597300	-0.26266300	-0.10346000
H	-1.92141200	2.23658200	2.93495000
H	-1.87737300	2.94378500	1.22453200
H	-0.13376100	-2.32437700	3.53672300
H	-1.86164600	-2.70949500	3.30229700
H	-0.63648700	-3.38222700	2.17988600
H	-1.55111700	2.49215800	-0.92670100
H	-0.81618600	1.30834200	-1.99447000
H	-0.23398200	4.14766700	-0.06182200
H	3.89208400	1.46942000	-0.55468200
H	1.95110200	5.15289200	0.49316700
H	4.03909500	3.80328700	0.25117400
H	2.63183700	-0.12350000	1.22768900
H	2.59335400	-2.12537000	-2.57879600
H	3.85226900	-2.02861300	2.22323900
H	3.84685200	-4.03279400	-1.58784500
H	4.47288800	-3.98378100	0.81903300
H	-1.42419300	-1.67474700	-1.68957700
H	-3.40878200	-2.70434600	-2.75188500
H	-5.67576000	-1.75218800	-2.35059900
H	-5.92794700	0.23530600	-0.87189500
H	-3.94685600	1.24666400	0.20167600

Compound 3a, conformation #26

0 1

C	-1.32052300	0.84848200	-0.04549400
C	-1.67568100	2.33757800	-0.07817100
C	-2.89528700	2.85504700	0.11637400
C	-0.57780400	3.32705100	-0.35691800
O	-0.75256000	4.48428500	-0.69794700
O	0.64309100	2.79761200	-0.16049300
C	1.76224500	3.66611900	-0.41707300
C	-1.04308100	0.35416300	-1.50547300
C	-0.52434100	-1.06962400	-1.59723500
C	0.81337900	-1.42546600	-1.29834000
C	-1.40952400	-2.09601000	-1.95496900
C	1.20870700	-2.77236400	-1.35036000
C	-1.00469800	-3.43044900	-2.02689800
C	0.31166700	-3.77356300	-1.72130500
C	1.88749400	-0.39816100	-1.05145400
O	2.04737000	0.52870900	-1.84201100

C	2.79123500	-0.54618600	0.13363200
C	4.01593200	0.14466900	0.13177800
C	2.43559100	-1.30668600	1.25967100
C	4.87118500	0.06803200	1.22736300
C	3.28799300	-1.37050100	2.36327300
C	4.50690800	-0.68867200	2.34719600
C	-2.33707800	-0.00230000	0.70538800
C	-2.02976100	-0.46248700	1.99432200
C	-2.93635800	-1.23749500	2.72190000
C	-4.17580900	-1.56792300	2.16990100
C	-4.49682700	-1.11812600	0.88661900
C	-3.58597000	-0.34543000	0.16194600
H	-0.37409300	0.76546900	0.49799500
H	-3.05105200	3.92756700	0.05177900
H	-3.75735200	2.24039100	0.35047600
H	2.64845300	3.06582500	-0.21488600
H	1.75779000	3.99879800	-1.45826900
H	1.73037400	4.53620800	0.24423200
H	-0.32135300	1.03753800	-1.95798600
H	-1.96855400	0.43211200	-2.08497400
H	-2.43950300	-1.83922800	-2.18623000
H	2.23865200	-3.03261800	-1.12343100
H	-1.71838300	-4.19628400	-2.31906000
H	0.64133600	-4.80751900	-1.77273000
H	4.28530700	0.73051900	-0.74137400
H	1.48795100	-1.83495700	1.28109300
H	5.82056700	0.59637900	1.21259000
H	3.00007400	-1.95272100	3.23424600
H	5.17257100	-0.74489200	3.20440900
H	-1.06643100	-0.21072900	2.43254800
H	-2.67173200	-1.58372200	3.71790400
H	-4.88358800	-2.17178500	2.73187600
H	-5.45763500	-1.37057800	0.44495800
H	-3.85417300	-0.00882100	-0.83559900

Compound 3a, conformation #28

0 1

C	-1.04236500	-0.27402800	0.09094300
C	-1.52168500	0.58624500	1.25905900
C	-0.79593900	0.71977100	2.37819800
C	-2.83241200	1.31866200	1.22446100
O	-3.29121400	1.96027100	2.15388700
O	-3.46540000	1.19659300	0.04229500
C	-4.74152000	1.85819000	-0.06290700
C	-0.70637800	0.54367500	-1.18920500
C	0.37339300	1.58935100	-0.98206100
C	1.75329300	1.27611900	-0.90317200
C	-0.00521400	2.92923000	-0.82368000
C	2.68978400	2.30068800	-0.68488800
C	0.93236700	3.94366500	-0.61974700
C	2.28941200	3.63005700	-0.55483400

C	2.27268400	-0.11493400	-1.15323100
O	1.87159000	-0.76590300	-2.11630200
C	3.30688000	-0.69767800	-0.23800200
C	3.51198300	-0.23033400	1.07085500
C	4.05505400	-1.79608900	-0.69733500
C	4.44880400	-0.84884100	1.90046500
C	4.99922000	-2.40209800	0.12610500
C	5.19687800	-1.92984300	1.42881800
C	-1.98247600	-1.46069900	-0.16227500
C	-2.69030900	-1.64525200	-1.35712400
C	-3.51423300	-2.76084800	-1.53779200
C	-3.64796000	-3.71229200	-0.52552000
C	-2.94763900	-3.54016800	0.67200300
C	-2.12541100	-2.42683400	0.84734500
H	-0.09771600	-0.71175500	0.43664500
H	-1.14860600	1.33005600	3.20408800
H	0.16353600	0.22388000	2.49492500
H	-5.43881300	1.46496800	0.68151000
H	-4.62785700	2.93647300	0.07668100
H	-5.09914600	1.64114600	-1.06983000
H	-1.60794700	1.04326700	-1.55023500
H	-0.38841300	-0.16168400	-1.95911900
H	-1.06185900	3.18099800	-0.87085100
H	3.74671100	2.05501800	-0.64109900
H	0.60051300	4.97318500	-0.51401500
H	3.03168200	4.40891300	-0.40404900
H	2.93130200	0.60511400	1.44761100
H	3.88554000	-2.15885100	-1.70615500
H	4.59309700	-0.48629500	2.91446400
H	5.58043800	-3.24306000	-0.24233100
H	5.93086100	-2.40574600	2.07370400
H	-2.60672600	-0.91980900	-2.15950900
H	-4.05238500	-2.88235500	-2.47470500
H	-4.28865500	-4.57881500	-0.66725400
H	-3.03833900	-4.27433500	1.46880700
H	-1.58431400	-2.30229100	1.78281600

Compound 3a, conformation #29

0 1

C	-1.75969700	-0.04870500	-0.34866500
C	-2.08109300	-0.72564500	0.97767000
C	-1.24648800	-0.81412200	2.02228400
C	-3.44404800	-1.33092000	1.15723700
O	-3.87058500	-1.79490300	2.20093400
O	-4.16301300	-1.32200200	0.01696300
C	-5.48372200	-1.89046600	0.10040400
C	-0.24580700	0.17452000	-0.61452300
C	0.61990700	-1.07688300	-0.68930700
C	1.92421300	-1.13579300	-0.14010900
C	0.16900900	-2.18989800	-1.41460900
C	2.70903600	-2.29007400	-0.31285700

C	0.94523300	-3.33924700	-1.56405800
C	2.22137300	-3.39682300	-1.00315800
C	2.50325400	-0.02895700	0.69907700
O	1.87278700	0.43744600	1.64862000
C	3.87311600	0.48885700	0.39190700
C	4.48058700	0.31912800	-0.86352000
C	4.54708100	1.22031900	1.38622000
C	5.73853200	0.86812500	-1.11586700
C	5.80764600	1.75446500	1.13715900
C	6.40561400	1.58010400	-0.11632700
C	-2.49345900	1.28964500	-0.48698400
C	-3.18883800	1.59166600	-1.66623600
C	-3.82215300	2.82579200	-1.83049700
C	-3.76986600	3.78158700	-0.81270700
C	-3.07981500	3.49207800	0.36728300
C	-2.44816500	2.25640000	0.52772700
H	-2.14848300	-0.69193400	-1.14272500
H	-1.57404300	-1.30139000	2.93624800
H	-0.23495300	-0.42053000	1.99897200
H	-5.43046500	-2.94622200	0.37976900
H	-6.08947800	-1.34971800	0.83242500
H	-5.91048600	-1.78398500	-0.89747500
H	0.15275600	0.86968500	0.12484500
H	-0.17775200	0.68806200	-1.58320500
H	-0.81330900	-2.16051200	-1.87710800
H	3.70493900	-2.32357100	0.11920700
H	0.55293200	-4.18481600	-2.12299100
H	2.83463400	-4.28723200	-1.10911900
H	3.96612600	-0.22592800	-1.64787500
H	4.06938200	1.35572300	2.35132800
H	6.19650400	0.73993300	-2.09270300
H	6.32573500	2.30776600	1.91566800
H	7.38824600	2.00091400	-0.31242000
H	-3.23568100	0.85144500	-2.46202800
H	-4.35864300	3.03836000	-2.75196100
H	-4.26430600	4.74158800	-0.93680900
H	-3.03433100	4.22748500	1.16680100
H	-1.92152100	2.03964900	1.45374400

Compound 3a, conformation #31

0 1

C	-1.91733500	-0.45450800	0.11287700
C	-3.00757900	-1.06350900	-0.77262000
C	-2.92917100	-2.24265900	-1.40248600
C	-4.28375300	-0.29275300	-0.97060300
O	-5.16417700	-0.59343300	-1.75760800
O	-4.37008200	0.77963600	-0.15709500
C	-5.57320400	1.56488100	-0.27432700
C	-1.21605300	0.71164000	-0.65299800
C	-0.34780000	1.59357700	0.22965100
C	1.05165500	1.74182700	0.07111200

C	-0.96664300	2.29251500	1.27751900
C	1.77025400	2.56790000	0.95586300
C	-0.24898700	3.11958300	2.14056500
C	1.13020700	3.26116000	1.97966500
C	1.79805500	1.14636900	-1.09066500
O	1.33559000	1.21323400	-2.22863100
C	3.13533500	0.50606300	-0.86719300
C	3.99952100	0.37585800	-1.96847300
C	3.52857900	-0.01689700	0.37593600
C	5.23770600	-0.24404300	-1.82622700
C	4.76396600	-0.65199600	0.51311800
C	5.62213900	-0.76054900	-0.58363800
C	-0.94168600	-1.48485700	0.66934100
C	-1.09002900	-1.92410300	1.99368500
C	-0.22805700	-2.87675700	2.54136700
C	0.80559300	-3.41180900	1.76907900
C	0.96792500	-2.98324300	0.44946500
C	0.10407200	-2.02820700	-0.09388600
H	-2.43178500	-0.01323600	0.97136900
H	-3.74944900	-2.58732900	-2.02446700
H	-2.06414400	-2.89067600	-1.31336400
H	-5.67746800	1.95783300	-1.28900600
H	-6.44987600	0.96165100	-0.02392000
H	-5.45835300	2.38122300	0.43931100
H	-2.00026600	1.33968000	-1.09143100
H	-0.64532100	0.30885500	-1.48821300
H	-2.04121300	2.19470000	1.40972900
H	2.84053800	2.68744700	0.81838400
H	-0.76726200	3.65394100	2.93246400
H	1.70186400	3.90711600	2.64014300
H	3.68600700	0.77202000	-2.92917400
H	2.86444500	0.05449600	1.23079600
H	5.90384700	-0.32765800	-2.68067500
H	5.05494400	-1.06202700	1.47628900
H	6.58692700	-1.24839100	-0.47267100
H	-1.89091900	-1.51145600	2.60338800
H	-0.36304700	-3.19730400	3.57139800
H	1.47988200	-4.15219200	2.19165400
H	1.77077900	-3.38923500	-0.16067200
H	0.25095900	-1.70786300	-1.12138900

Compound 3a, conformation #33

0 1

C	-1.40970600	0.02953200	-0.64780700
C	-2.28500800	-1.21409600	-0.73716400
C	-1.83474800	-2.47429200	-0.80412400
C	-3.75659100	-0.93125800	-0.76989500
O	-4.22849600	0.18201600	-0.92983800
O	-4.51561000	-2.03262300	-0.61460400
C	-5.93998200	-1.82911300	-0.68380800
C	0.05161200	-0.23606700	-1.10463700

C	0.86550400	1.03351400	-1.28865700
C	1.96777900	1.38028600	-0.47811500
C	0.48100600	1.93422000	-2.29604300
C	2.61532500	2.61481100	-0.66417100
C	1.14693700	3.14165600	-2.49685000
C	2.21930700	3.48960000	-1.67124900
C	2.50973100	0.52573000	0.64196100
O	2.58252600	0.99784900	1.77349100
C	3.04205300	-0.84433700	0.35933300
C	3.31414400	-1.69427500	1.44555800
C	3.32941800	-1.28287200	-0.94360300
C	3.84757400	-2.96210400	1.23215700
C	3.87529500	-2.54950900	-1.15473700
C	4.13077100	-3.39153700	-0.06950200
C	-1.47136300	0.63909900	0.75565200
C	-1.15375200	-0.12334100	1.88986600
C	-1.18435700	0.44384500	3.16549600
C	-1.53951200	1.78563500	3.32996700
C	-1.86204600	2.55344300	2.20886500
C	-1.82874300	1.98240400	0.93389300
H	-1.84683300	0.76433700	-1.33103300
H	-2.51734600	-3.31536700	-0.86323100
H	-0.77458600	-2.70718200	-0.80910700
H	-6.38164000	-2.81702700	-0.54831500
H	-6.27117300	-1.15413700	0.10999400
H	-6.22422500	-1.41667900	-1.65576500
H	0.01211000	-0.76997400	-2.06224700
H	0.53462200	-0.90578100	-0.39078000
H	-0.36211700	1.67827500	-2.93358800
H	3.44782500	2.87265600	-0.01561300
H	0.82730000	3.81006300	-3.29200800
H	2.74201100	4.43167200	-1.81211300
H	3.09811700	-1.34520800	2.45032600
H	3.13846500	-0.63099000	-1.79013000
H	4.04605900	-3.61645600	2.07663900
H	4.10229100	-2.87764700	-2.16530100
H	4.55123200	-4.37980200	-0.23578600
H	-0.88581100	-1.17102900	1.77588700
H	-0.93451800	-0.16405400	4.03157600
H	-1.56653800	2.22696300	4.32293200
H	-2.14238800	3.59752200	2.32433500
H	-2.08410800	2.58607000	0.06662400

Compound 3a, conformation #35

0 1

C	1.42406800	0.05142500	-0.34232700
C	1.89447100	0.79394300	0.90170000
C	1.13389500	1.09330100	1.96300200
C	3.33551000	1.21388100	0.97812600
O	3.87272600	1.68843800	1.96407000
O	3.98670500	1.02042200	-0.18542800

C	5.37687000	1.39771800	-0.20046500
C	-0.11642500	0.02794700	-0.53904500
C	-0.78397200	1.38379600	-0.72519600
C	-1.96495800	1.73426500	-0.03770000
C	-0.23641000	2.32813100	-1.60777100
C	-2.53675800	3.00469100	-0.20949100
C	-0.82126200	3.58012700	-1.79666400
C	-1.97513100	3.92607700	-1.09036500
C	-2.65292800	0.81543000	0.94608000
O	-2.66529600	1.11275200	2.13838200
C	-3.36156500	-0.40391600	0.45371400
C	-3.86046200	-1.31906600	1.39786700
C	-3.57174300	-0.64418800	-0.91407000
C	-4.54596100	-2.45611600	0.98180900
C	-4.26574800	-1.78139300	-1.32877900
C	-4.75026000	-2.68880200	-0.38349800
C	1.95672900	-1.38539200	-0.37048100
C	2.52679500	-1.89977900	-1.54360400
C	2.97085000	-3.22252100	-1.60588500
C	2.85202800	-4.05529300	-0.49022300
C	2.28587100	-3.55389300	0.68454000
C	1.84315300	-2.23011500	0.74294900
H	1.86147200	0.55824200	-1.20686500
H	1.56678800	1.60775800	2.81567100
H	0.07879000	0.85107800	2.01948400
H	5.48714300	2.46602300	0.00410400
H	5.93818500	0.82410300	0.54190500
H	5.73090700	1.16581900	-1.20554100
H	-0.57245100	-0.51229200	0.29551900
H	-0.30746100	-0.58482000	-1.42953800
H	0.66728500	2.08140700	-2.15830600
H	-3.43530000	3.26057700	0.34577900
H	-0.37217600	4.28534100	-2.49111100
H	-2.43313500	4.90235000	-1.22340700
H	-3.69866600	-1.12280700	2.45302000
H	-3.20402600	0.06117900	-1.65238000
H	-4.92324400	-3.16200100	1.71666900
H	-4.42938700	-1.95784300	-2.38821400
H	-5.28786800	-3.57576800	-0.70806100
H	2.62436100	-1.25683300	-2.41559600
H	3.41272100	-3.60036600	-2.52456100
H	3.19986400	-5.08407500	-0.53504800
H	2.19073900	-4.19222100	1.55945500
H	1.41242700	-1.84929000	1.66548300

Compound 3a, conformation #38

0 1

C	-1.41312000	-0.16871100	-0.68391600
C	-2.13638300	-1.48362100	-0.95198700
C	-1.54057400	-2.64761700	-1.24370900
C	-3.63744100	-1.50306400	-0.87903600

O	-4.32389700	-2.50959800	-0.91555400
O	-4.15967900	-0.26530800	-0.78452500
C	-5.59651100	-0.19040100	-0.71022400
C	0.05742800	-0.18879000	-1.19237300
C	0.70691400	1.18397600	-1.20578300
C	1.74515200	1.57113100	-0.33124200
C	0.21952800	2.14058200	-2.11230600
C	2.22926700	2.89153500	-0.35857400
C	0.72519600	3.43817200	-2.15622000
C	1.73490500	3.82041200	-1.26900000
C	2.37894800	0.67062500	0.70105900
O	2.38618200	1.02311000	1.87769100
C	3.07711000	-0.58672100	0.28630400
C	3.42812100	-1.51601200	1.28099000
C	3.44203500	-0.83780800	-1.04649800
C	4.11563400	-2.67955200	0.94779800
C	4.14238900	-1.99861900	-1.37675200
C	4.47598400	-2.92198600	-0.38269200
C	-1.49569700	0.21316900	0.79676600
C	-1.11270900	-0.69018200	1.79954200
C	-1.16074300	-0.32647900	3.14703400
C	-1.59569900	0.94936900	3.51595900
C	-1.98154700	1.85654900	2.52649000
C	-1.93266500	1.48871000	1.17951300
H	-1.94191200	0.60878100	-1.24387400
H	-2.13547000	-3.54091000	-1.40814100
H	-0.46394500	-2.74828900	-1.32923800
H	-6.05009800	-0.61191800	-1.61123800
H	-5.96360500	-0.72714900	0.16857500
H	-5.82918900	0.87197700	-0.63004400
H	0.05276600	-0.58145200	-2.21645800
H	0.63633800	-0.88876100	-0.58757000
H	-0.57693000	1.85500100	-2.79577000
H	3.01370600	3.17593100	0.33699100
H	0.33019200	4.14897500	-2.87729800
H	2.13322100	4.83110700	-1.28761100
H	3.15039100	-1.31103100	2.31000700
H	3.19086900	-0.12183700	-1.82265400
H	4.37409100	-3.39732400	1.72154700
H	4.42848300	-2.18094900	-2.40891500
H	5.01683800	-3.82818700	-0.64232600
H	-0.78064500	-1.68867700	1.52577900
H	-0.86084500	-1.04167300	3.90896800
H	-1.63570800	1.23235800	4.56465100
H	-2.32396800	2.85116900	2.80129600
H	-2.23856200	2.19923600	0.41555000

G. NMR spectra & HPLC traces

2g, ^{1}H NMR, 400 MHz, $CDCl_3$

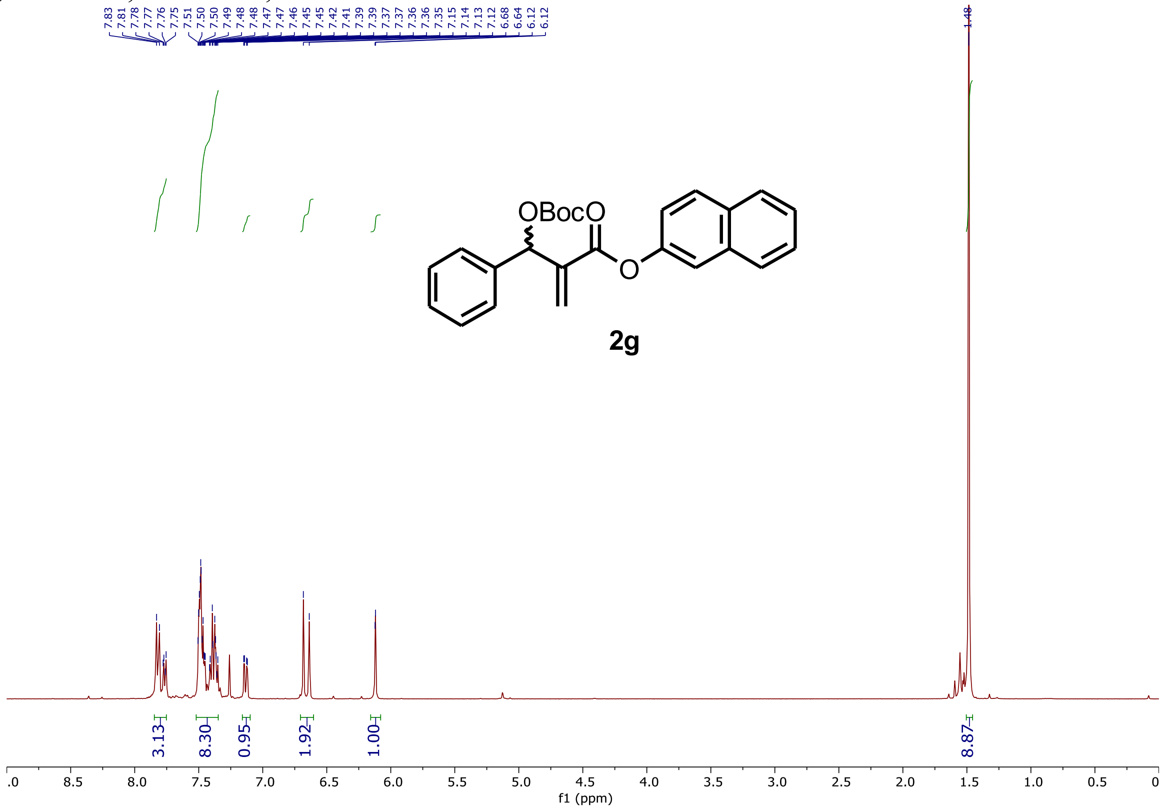

2g, ^{13}C NMR, 101 MHz, $CDCl_3$

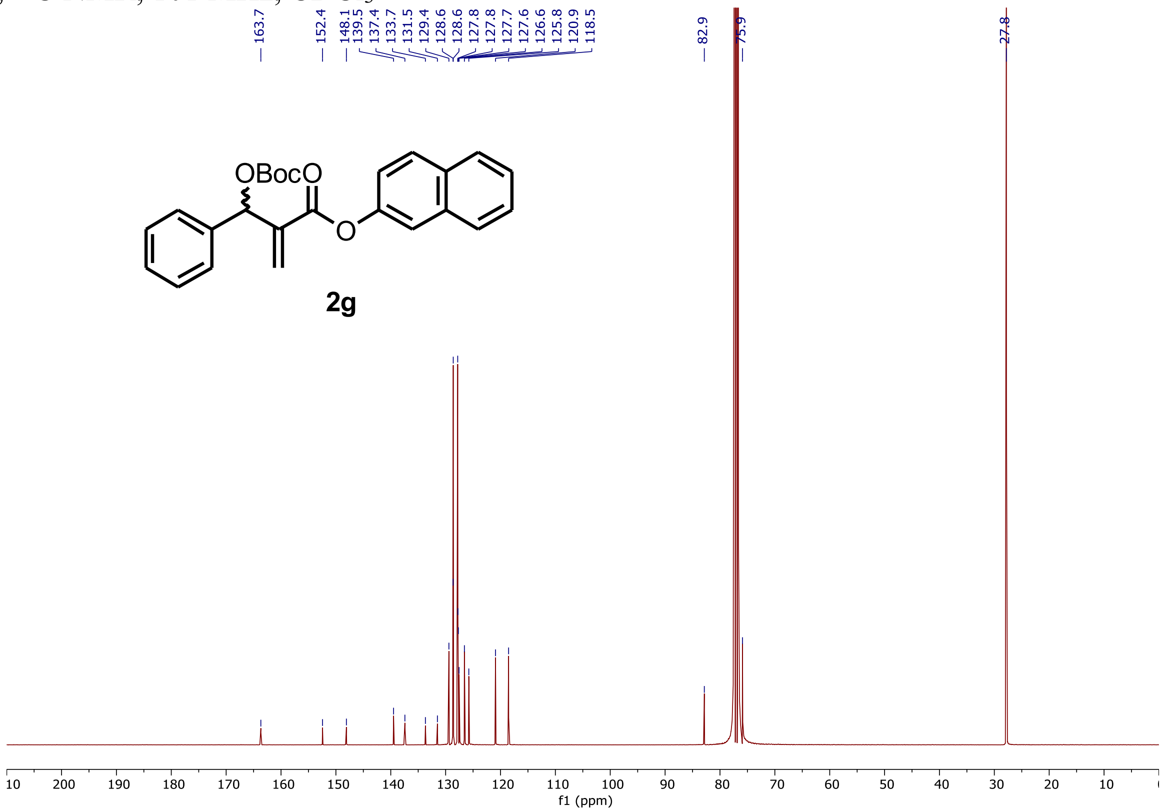

2f, ^{1}H NMR, 200 MHz, $CDCl_3$

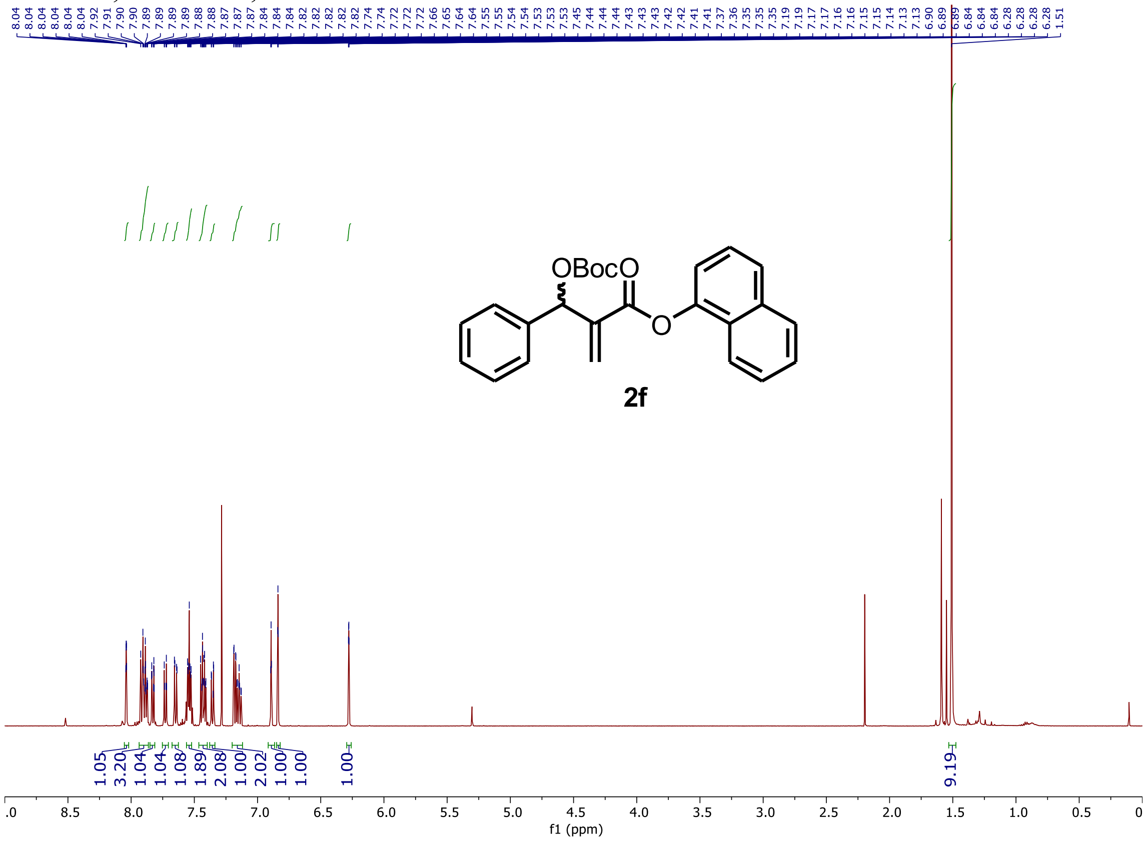

2f, ^{13}C NMR, 101 MHz, $CDCl_3$

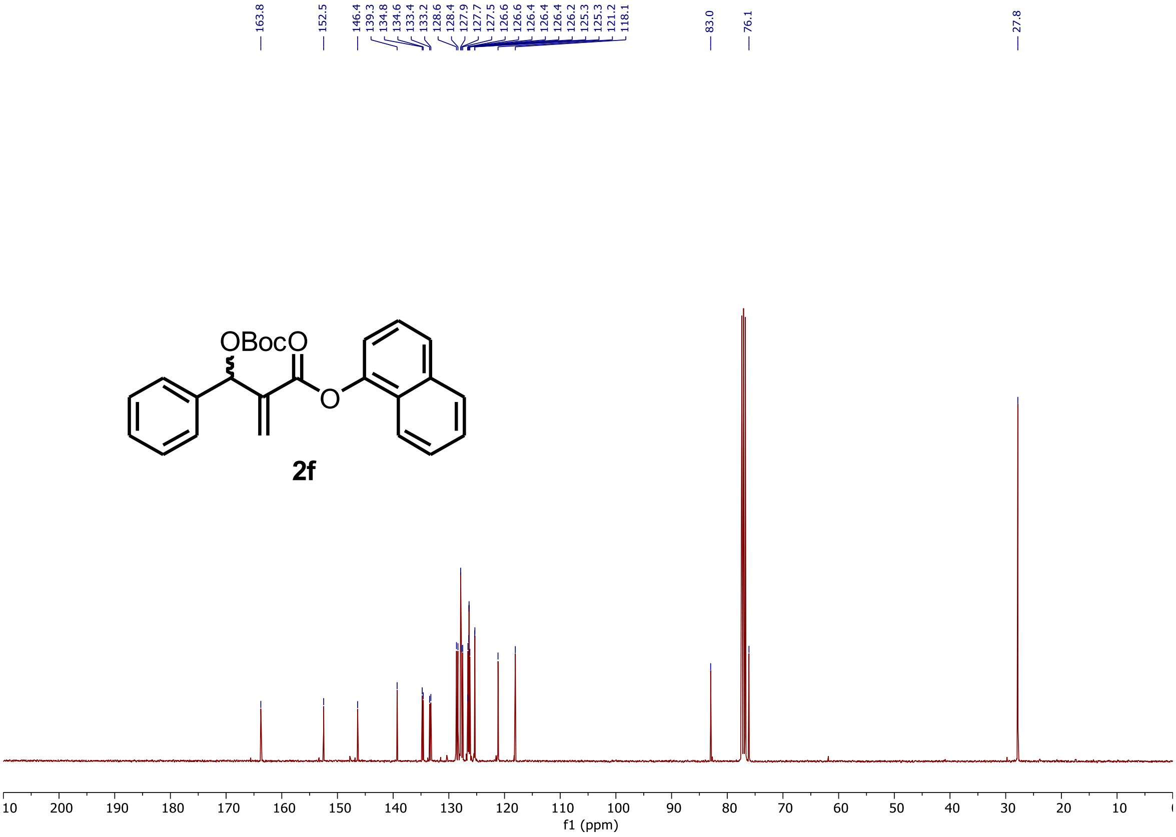

2h, ^{1}H NMR, 400 MHz, $CDCl_3$

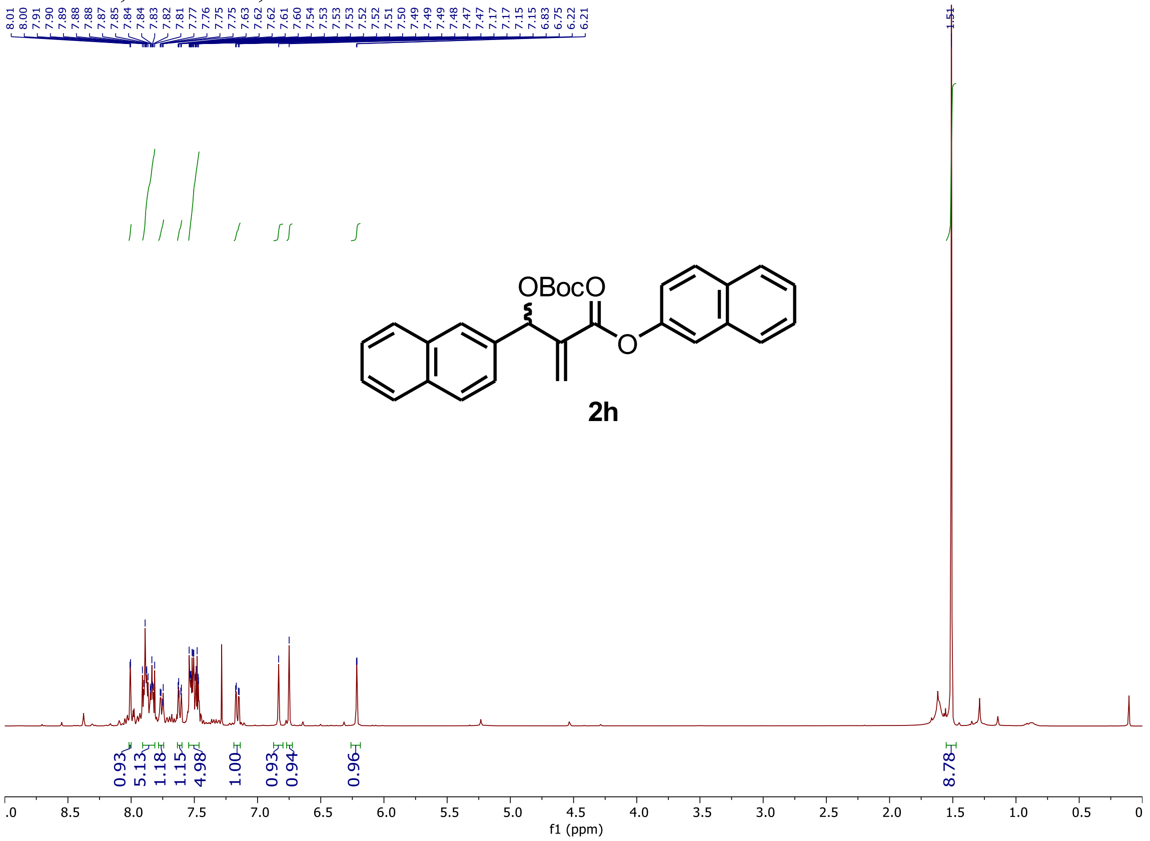

2h, ^{13}C NMR, 101 MHz, $CDCl_3$

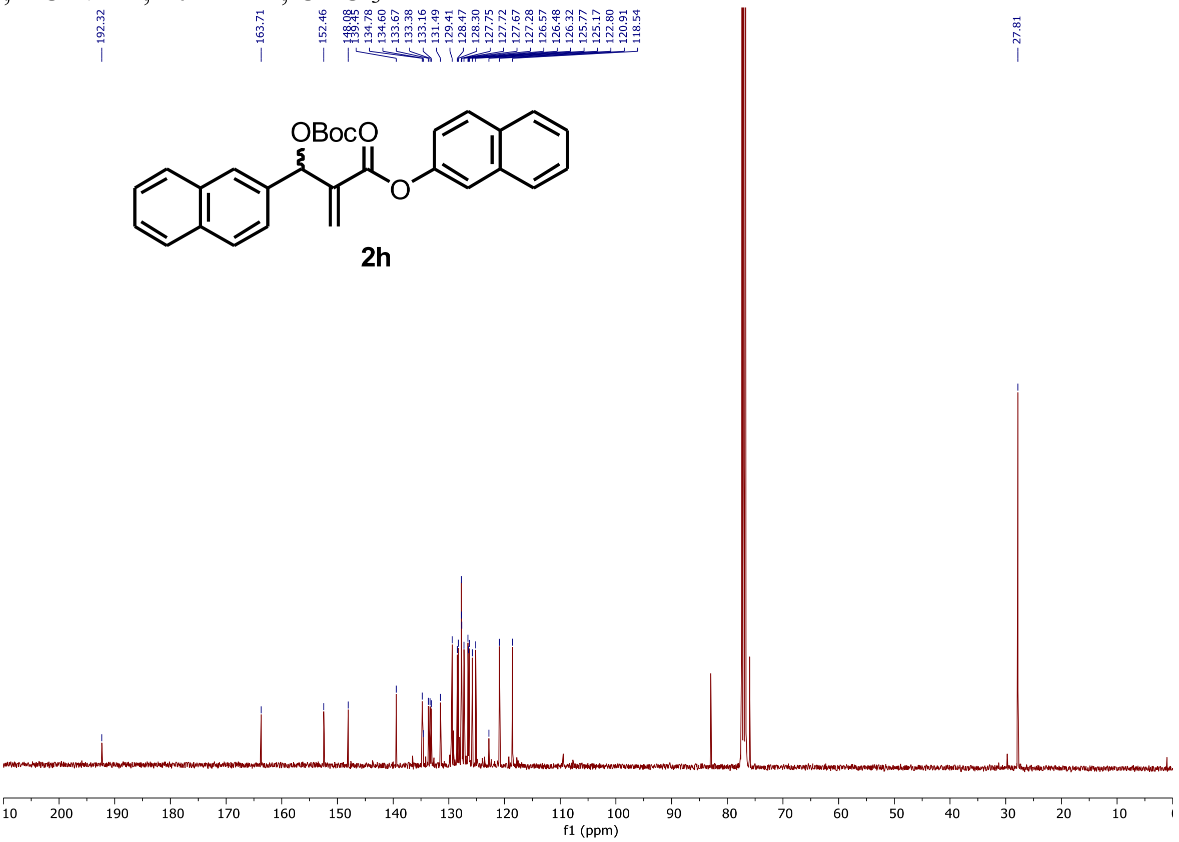

2j, ^{1}H NMR, 200 MHz, $CDCl_3$

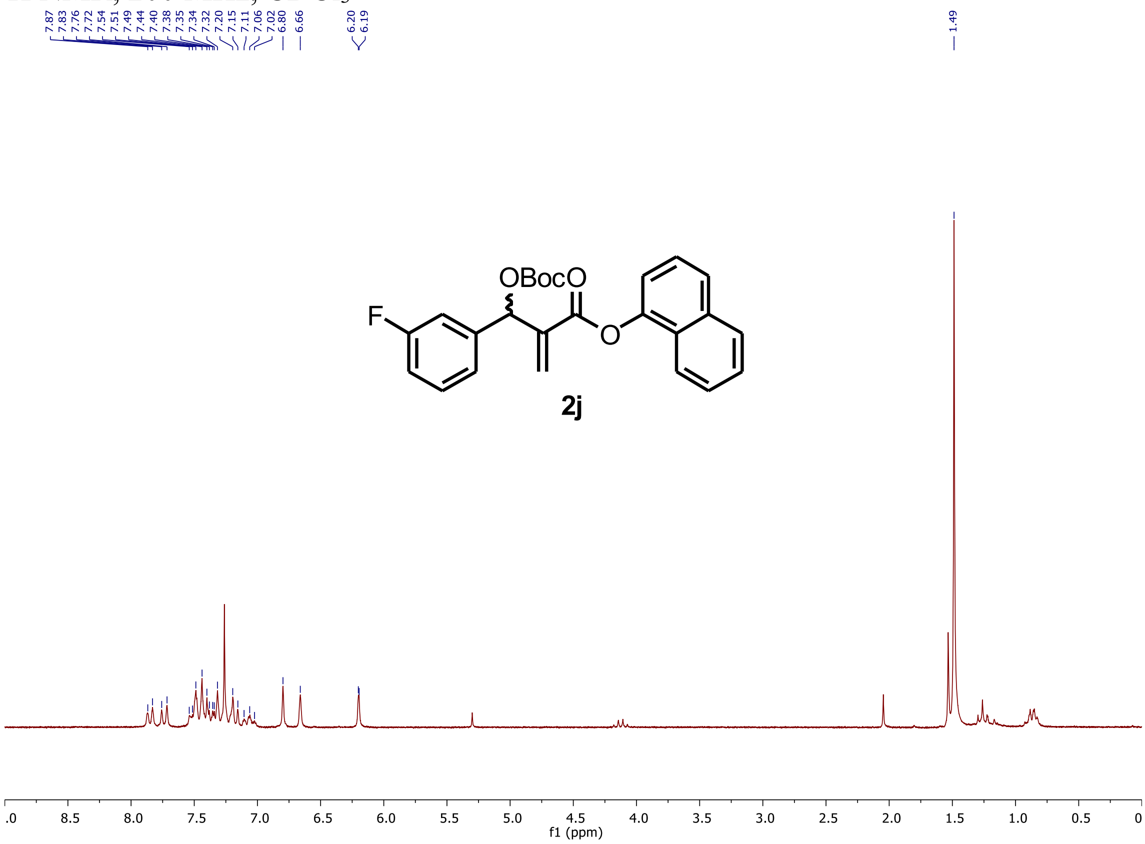

2j, ^{13}C NMR, 101 MHz, $CDCl_3$

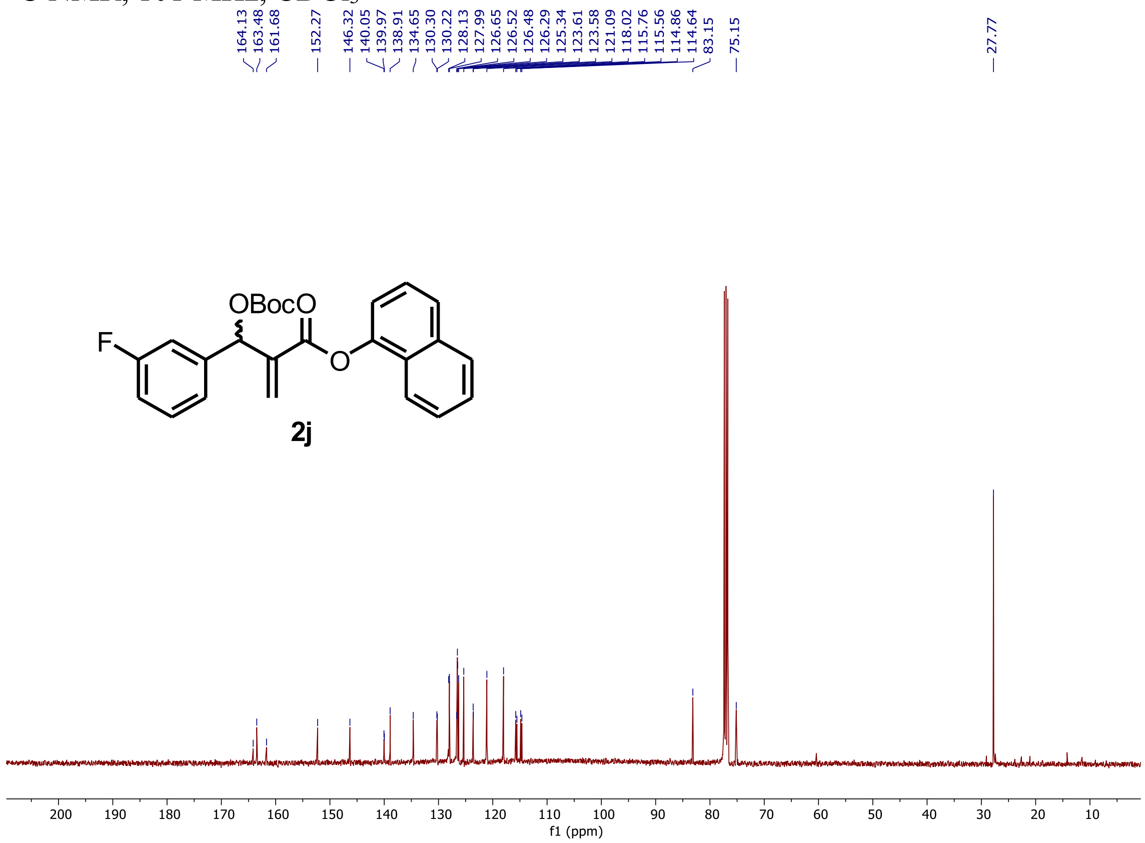

2j, ^{19}F NMR, 376 MHz, $CDCl_3$

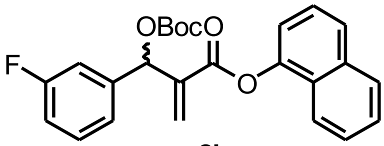

2j

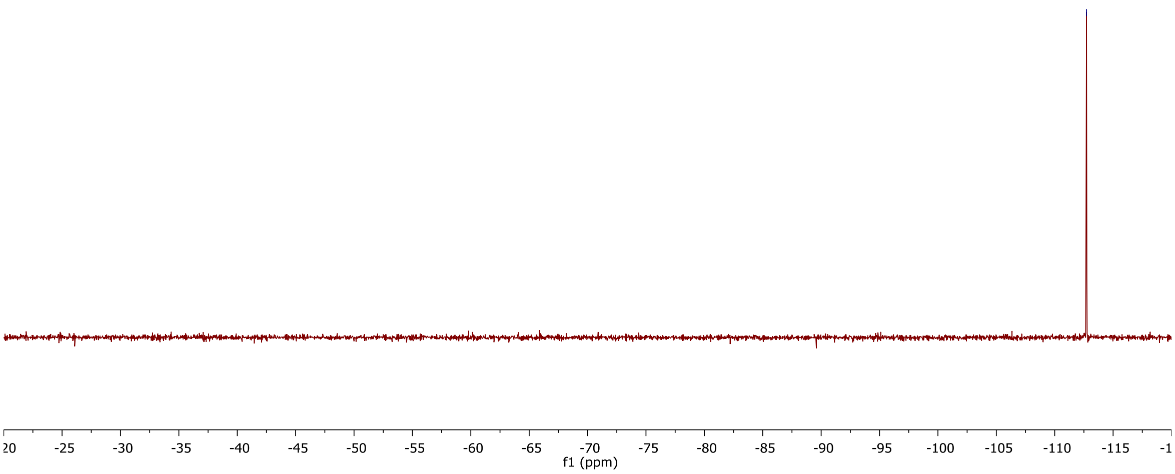

2k, ^{1}H NMR, 200 MHz, $CDCl_3$

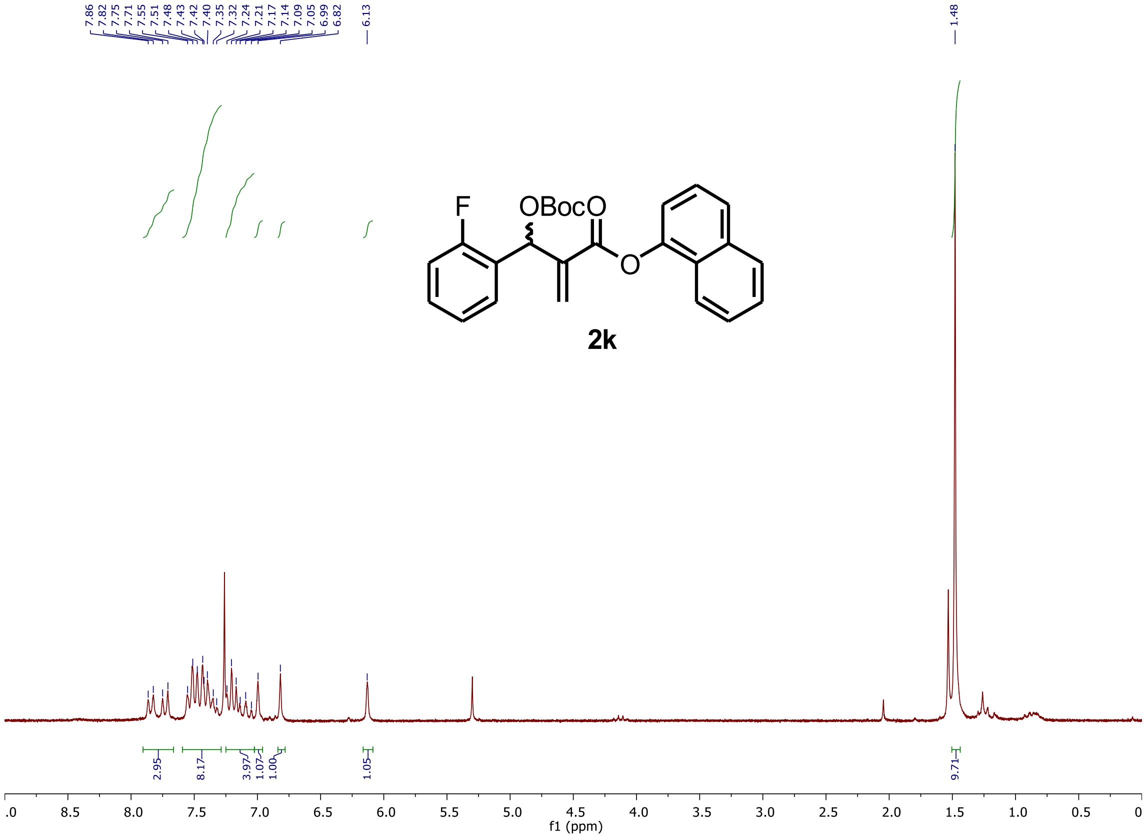

2k, ^{13}C NMR, 101 MHz, $CDCl_3$

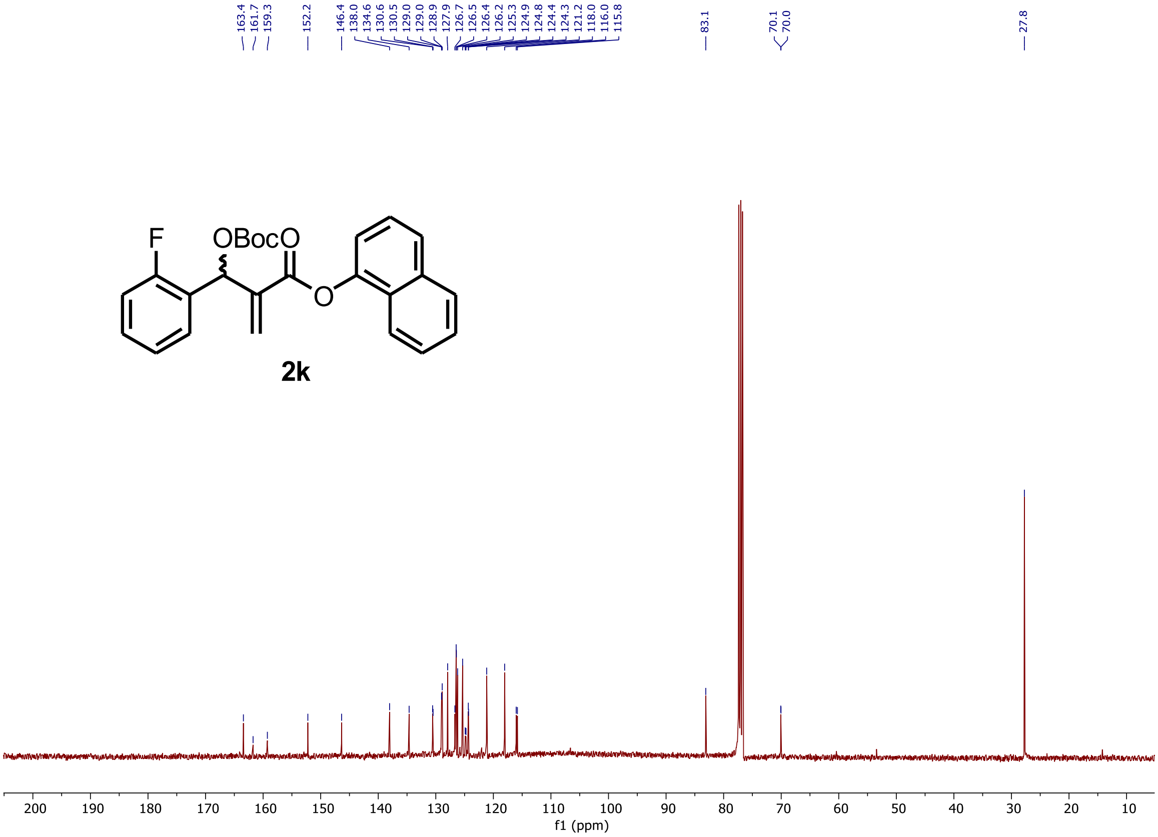

2k, ^{19}F NMR, 376 MHz, $CDCl_3$

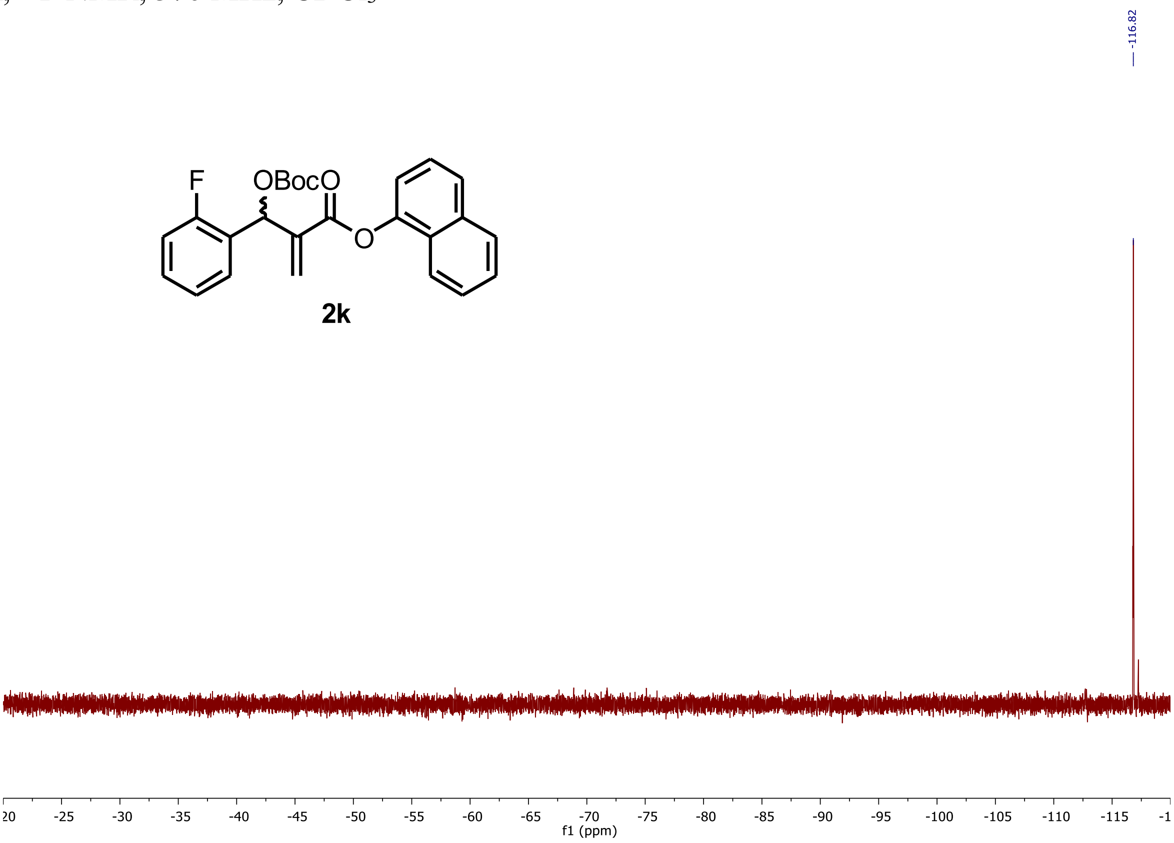

3g, ¹H NMR, 400 MHz, CDCl₃

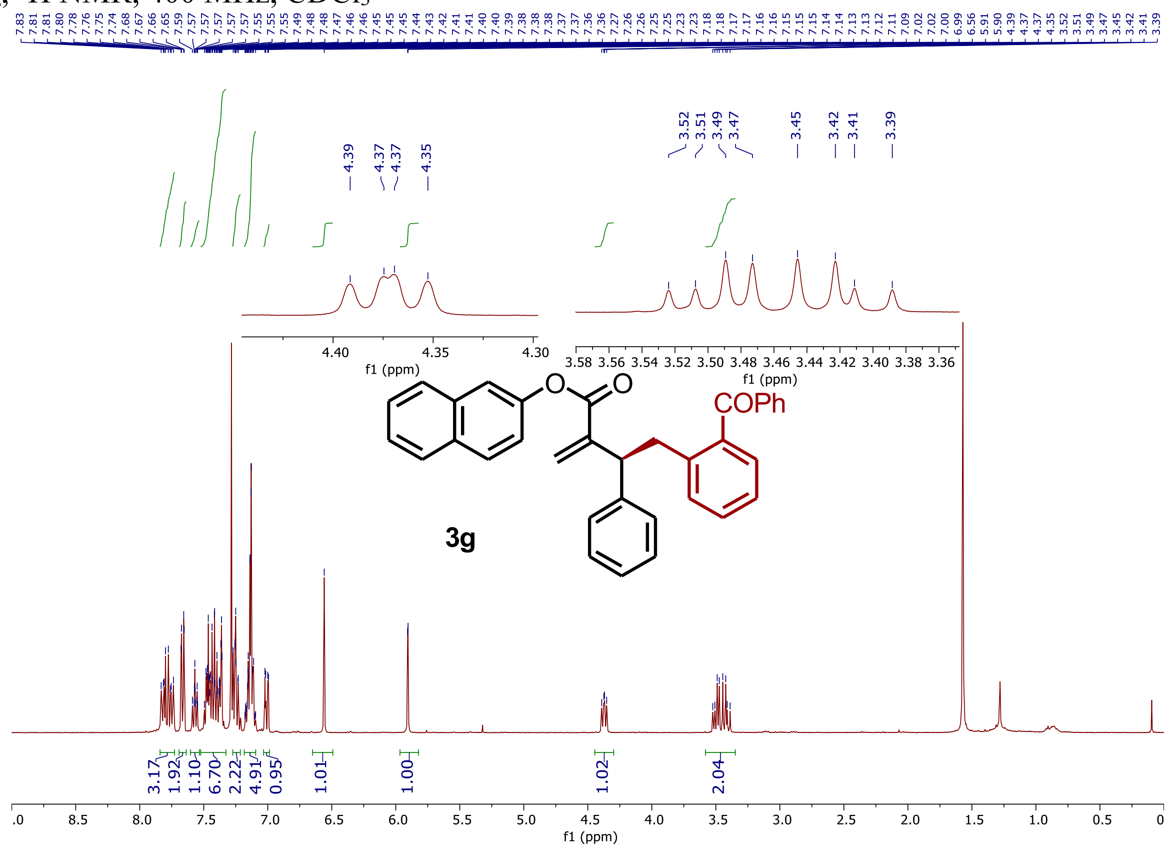

3g, ¹³C NMR, 101 MHz, CDCl₃

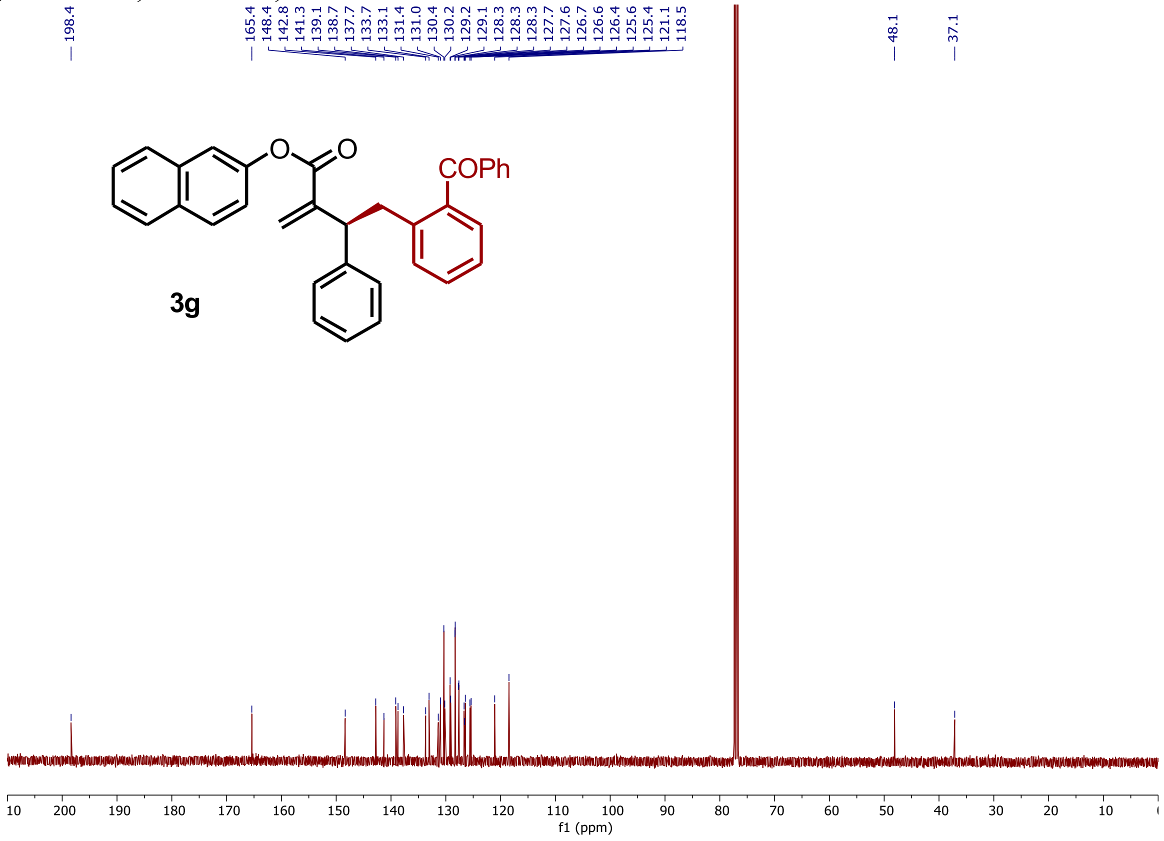

3f, ^{1}H NMR, 400 MHz, $CDCl_3$

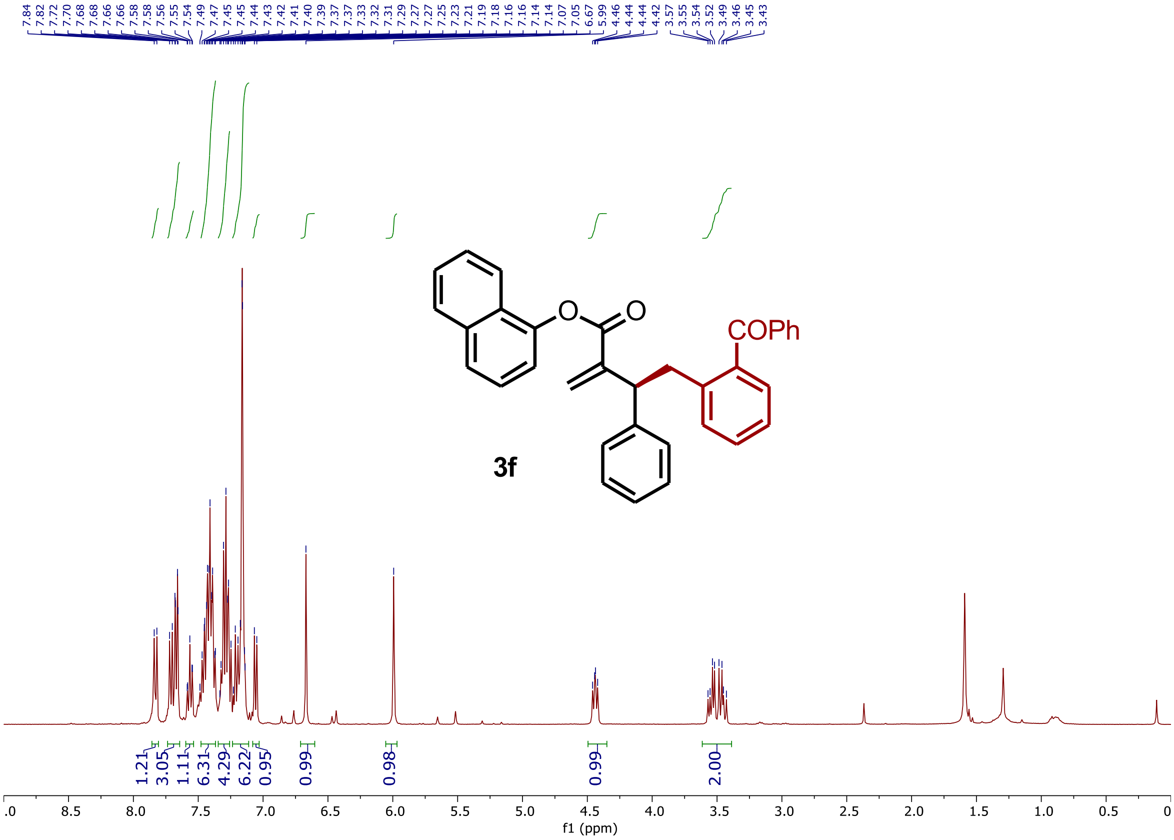

3f, ^{13}C NMR, 101 MHz, $CDCl_3$

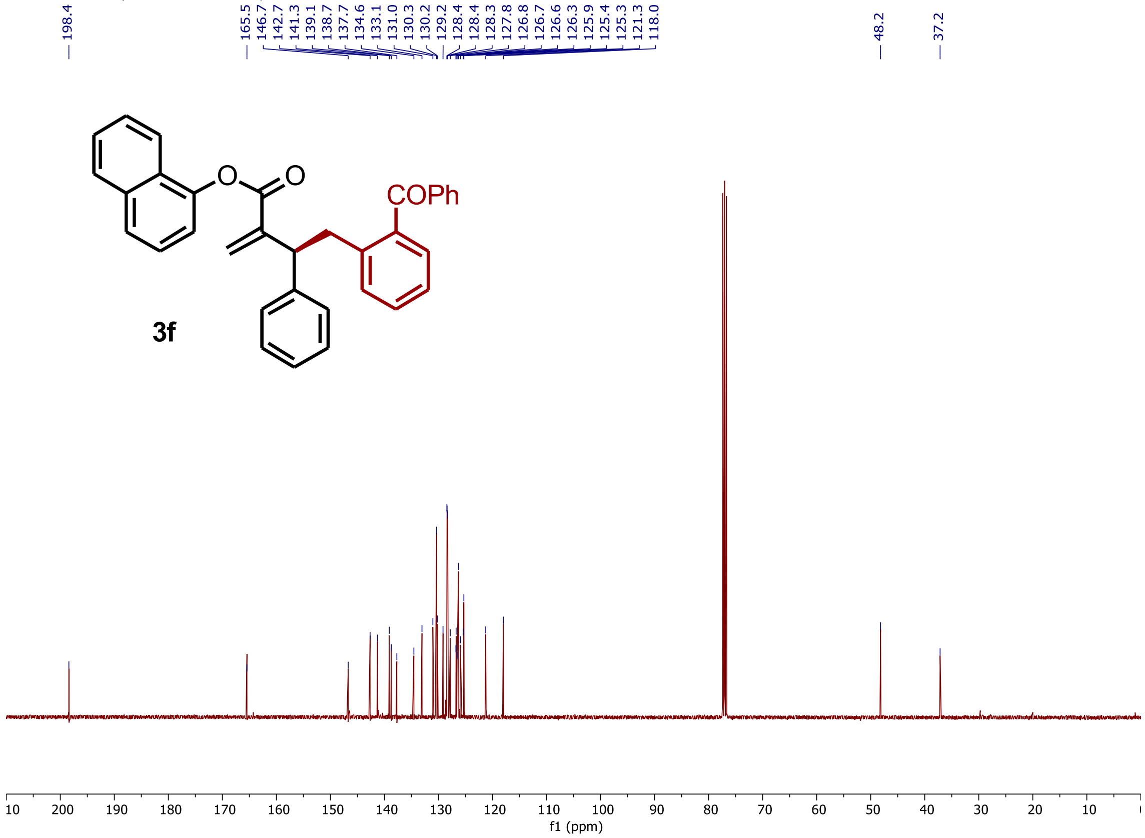

3h, ^{1}H NMR, 400 MHz, CDCl$_3$

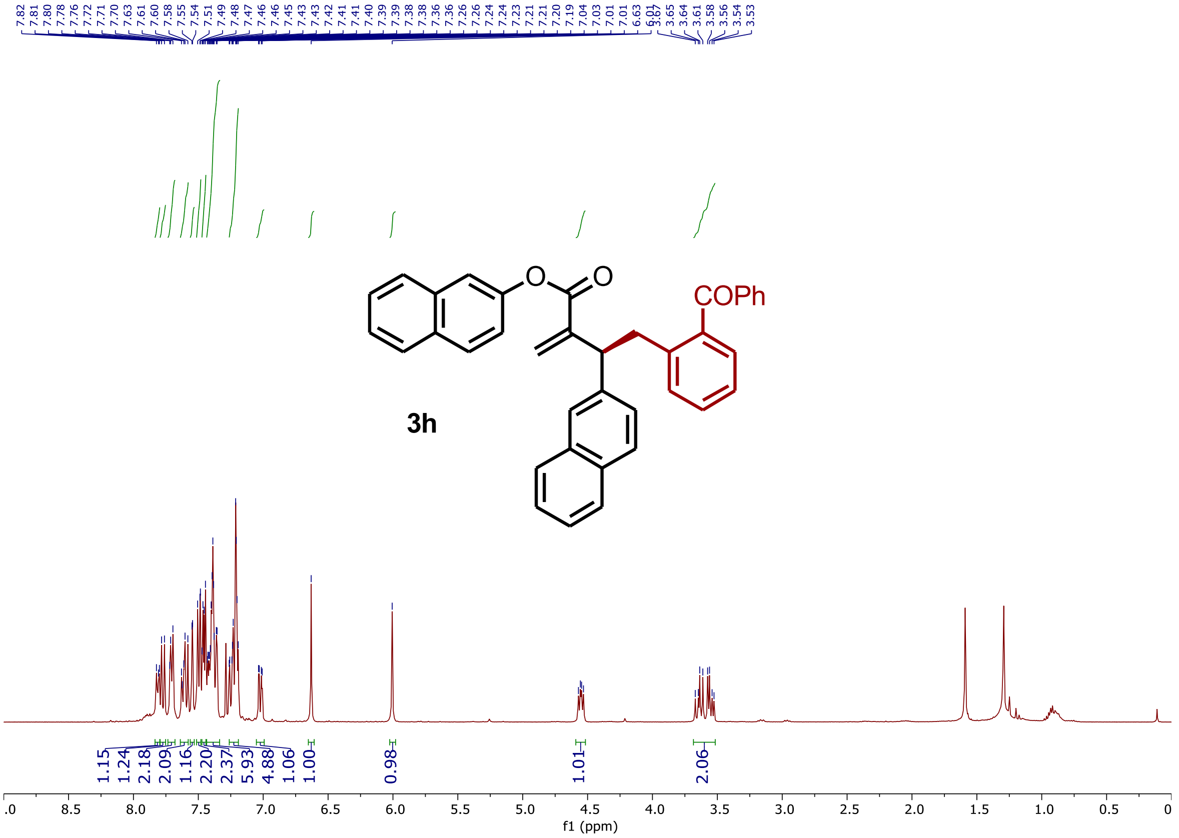

3h, ^{13}C NMR, 101 MHz, CDCl$_3$

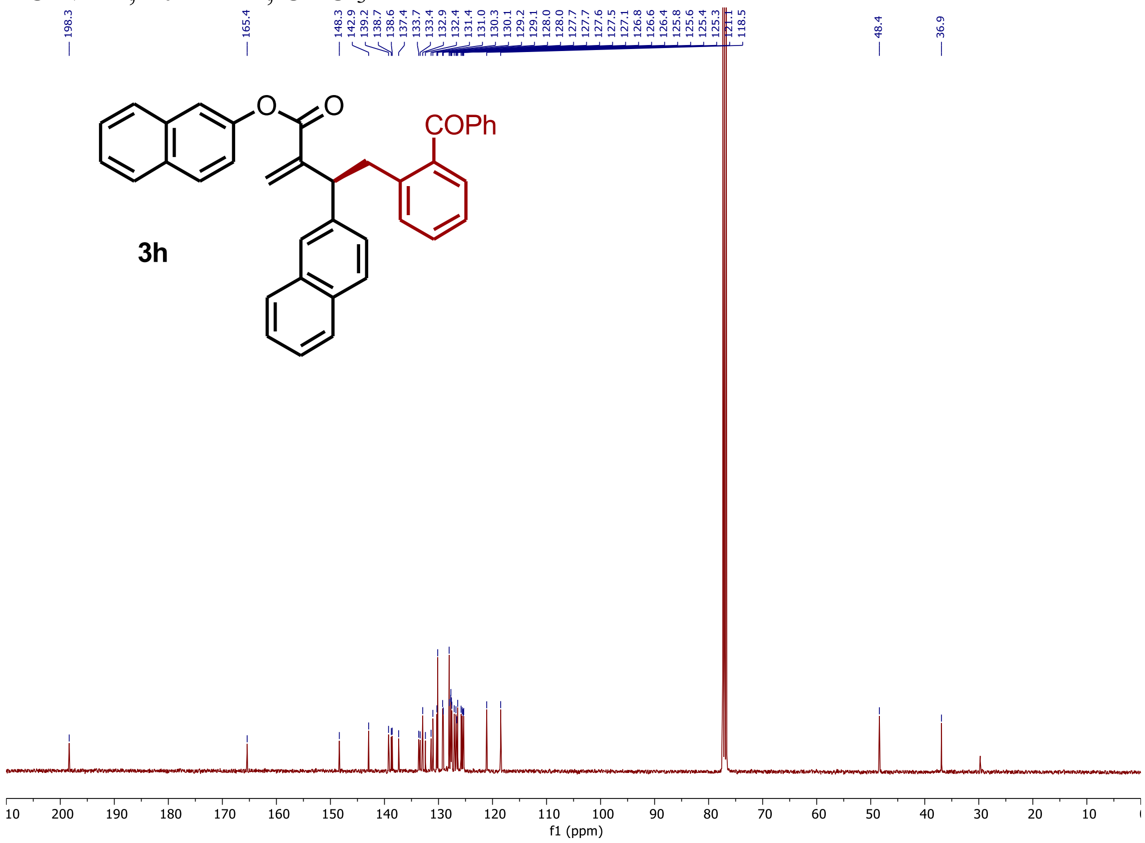

3i, ^{1}H NMR, 400 MHz, $CDCl_3$

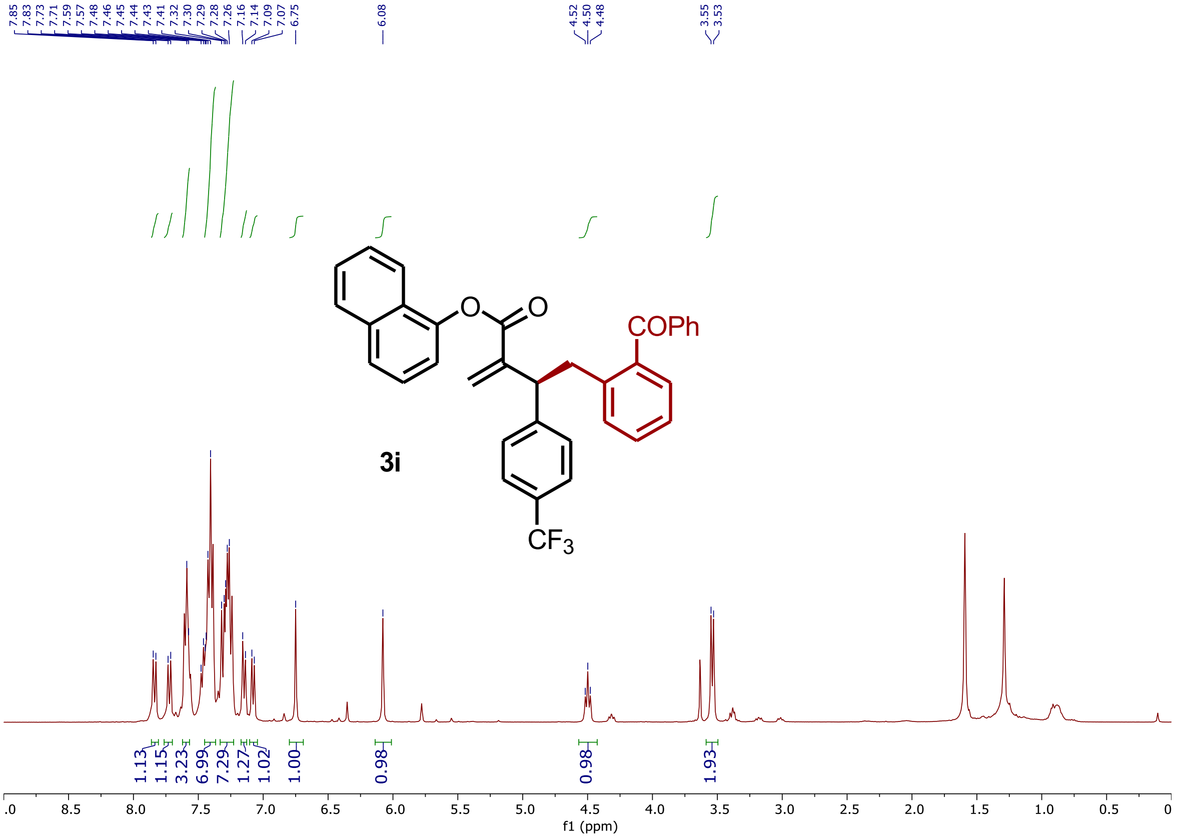

3i, ^{13}C NMR, 101 MHz, $CDCl_3$

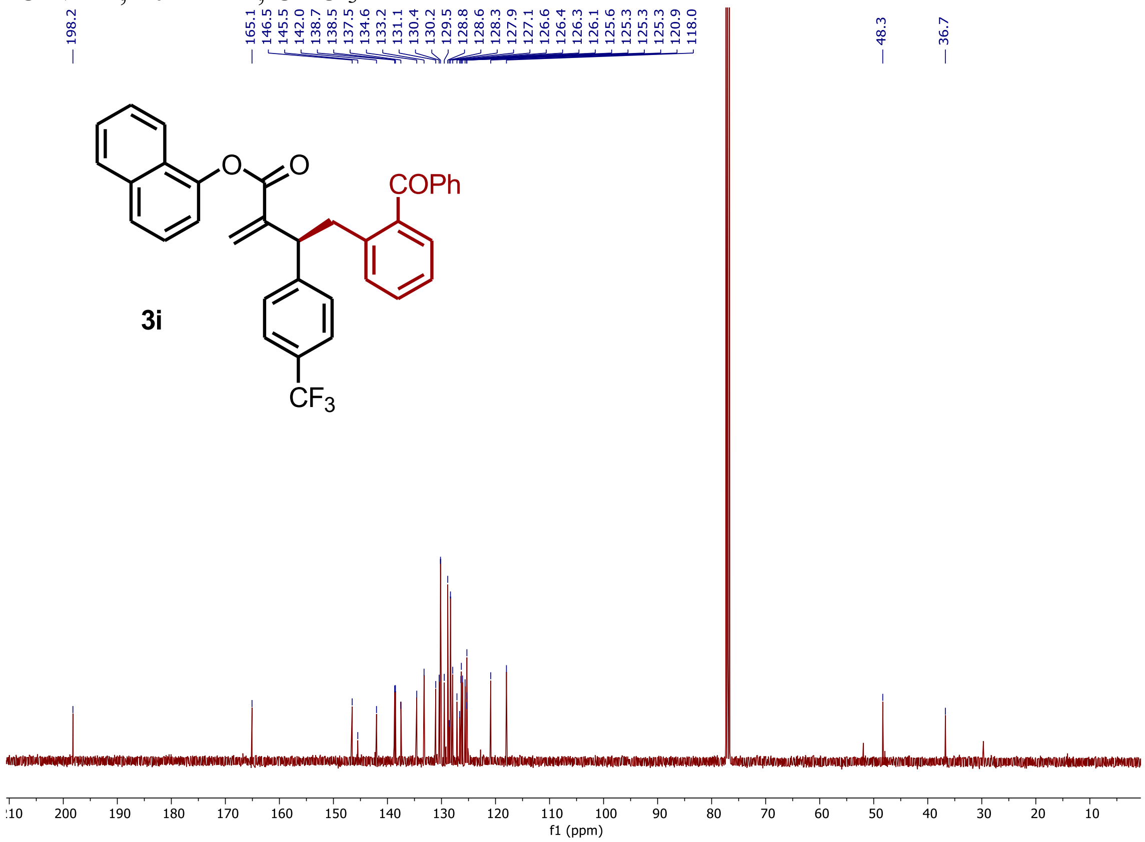

3i, ^{19}F NMR, 376 MHz, $CDCl_3$

-62.68

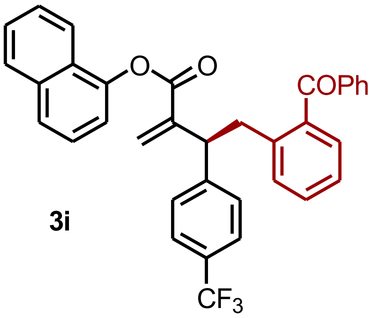

3i

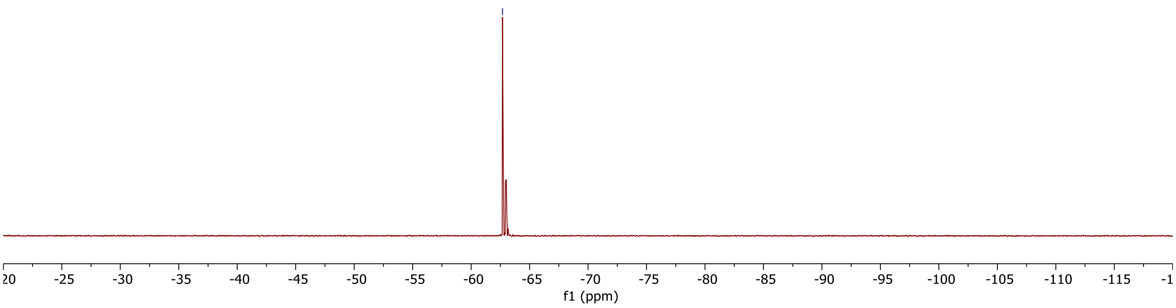

3j, ^{1}H NMR, 400 MHz, $CDCl_3$

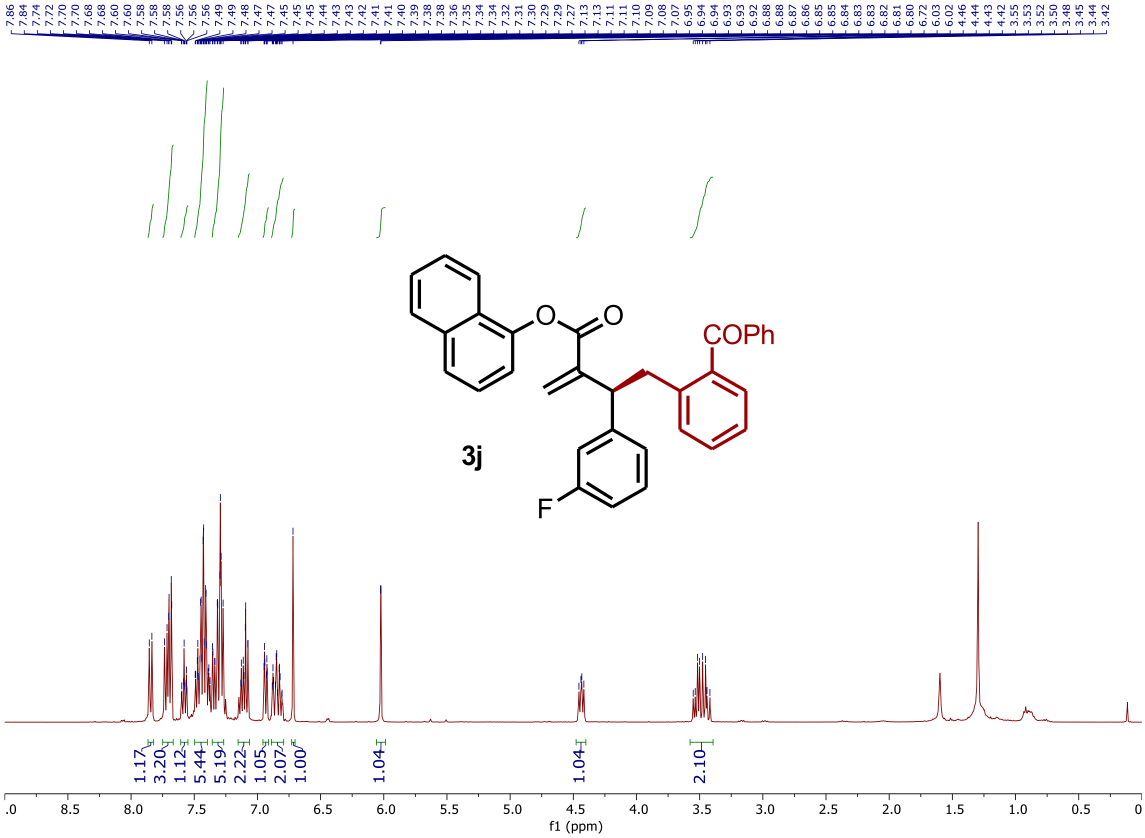

3j, ^{13}C NMR, 101 MHz, $CDCl_3$

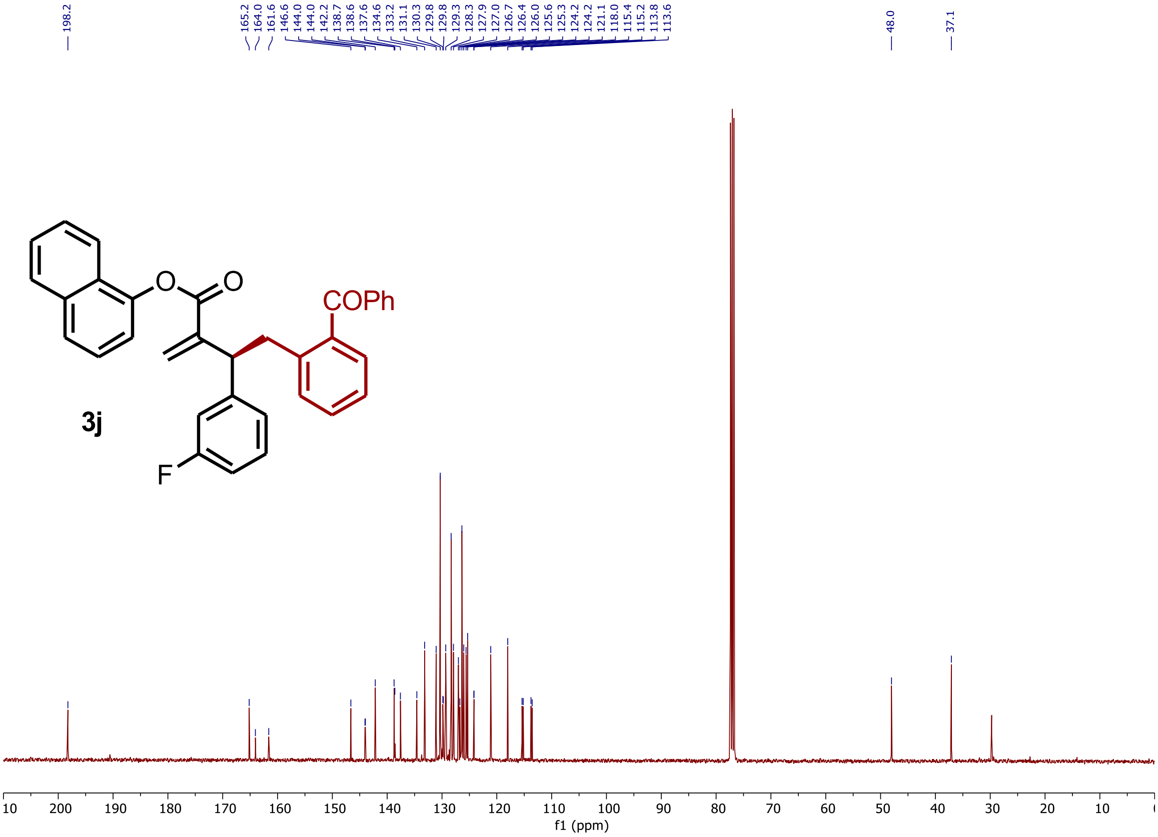

3j, ^{19}F NMR, 376 MHz, $CDCl_3$

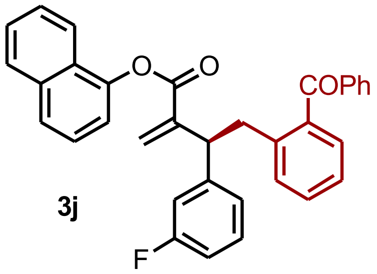

3j

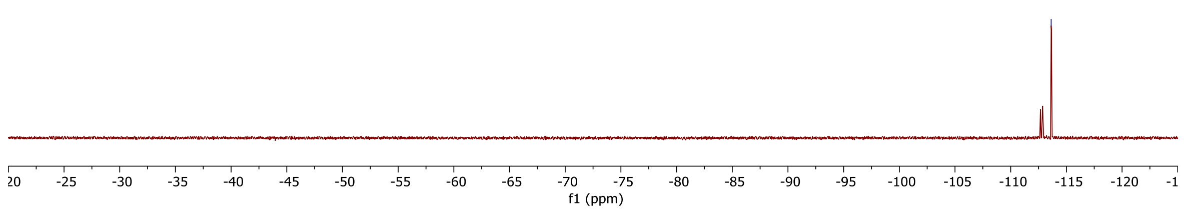

3k, ^{1}H NMR, 500 MHz, CDCl$_3$

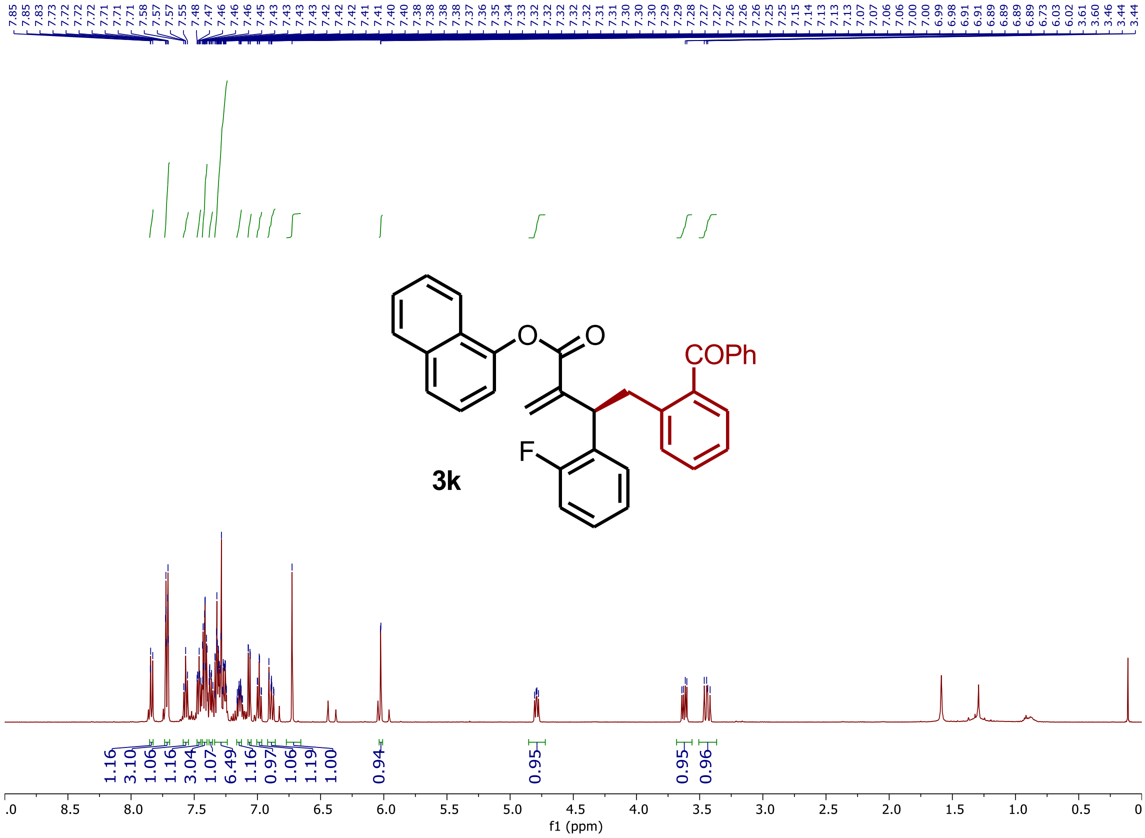

3k, ^{13}C NMR, 101 MHz, CDCl$_3$

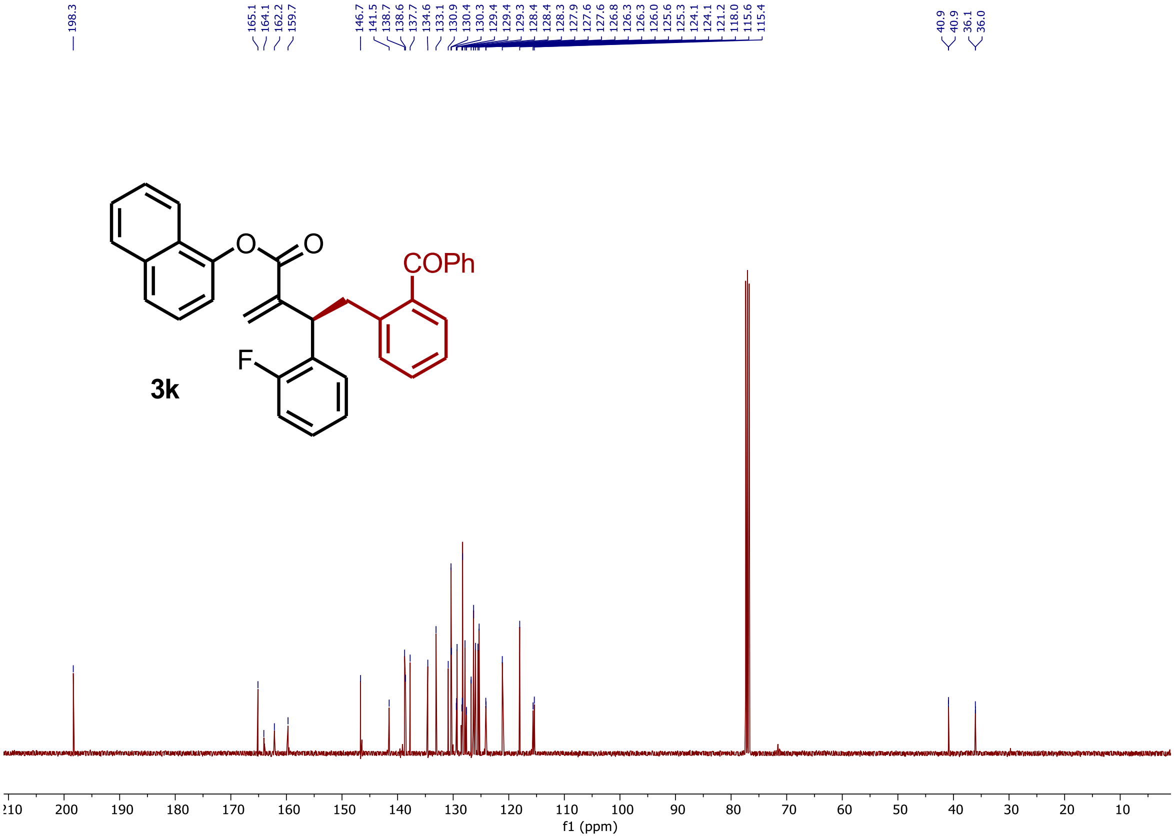

3k, ^{19}F NMR, 376 MHz, $CDCl_3$

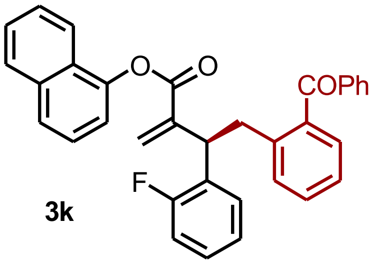

3k

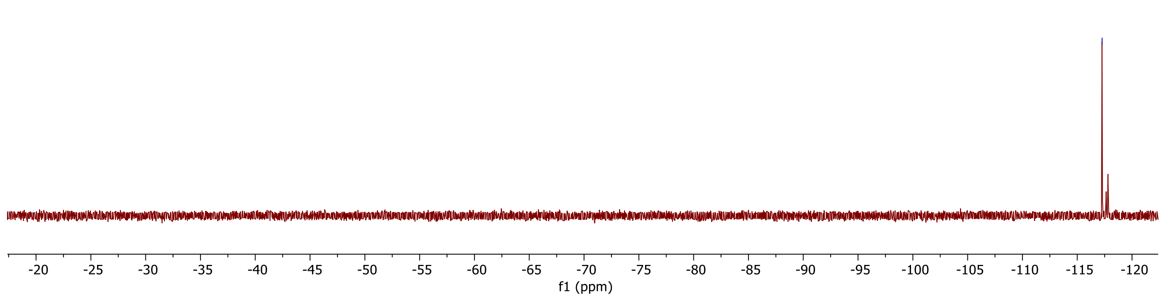

3l, ^{1}H NMR, 400 MHz, $CDCl_3$

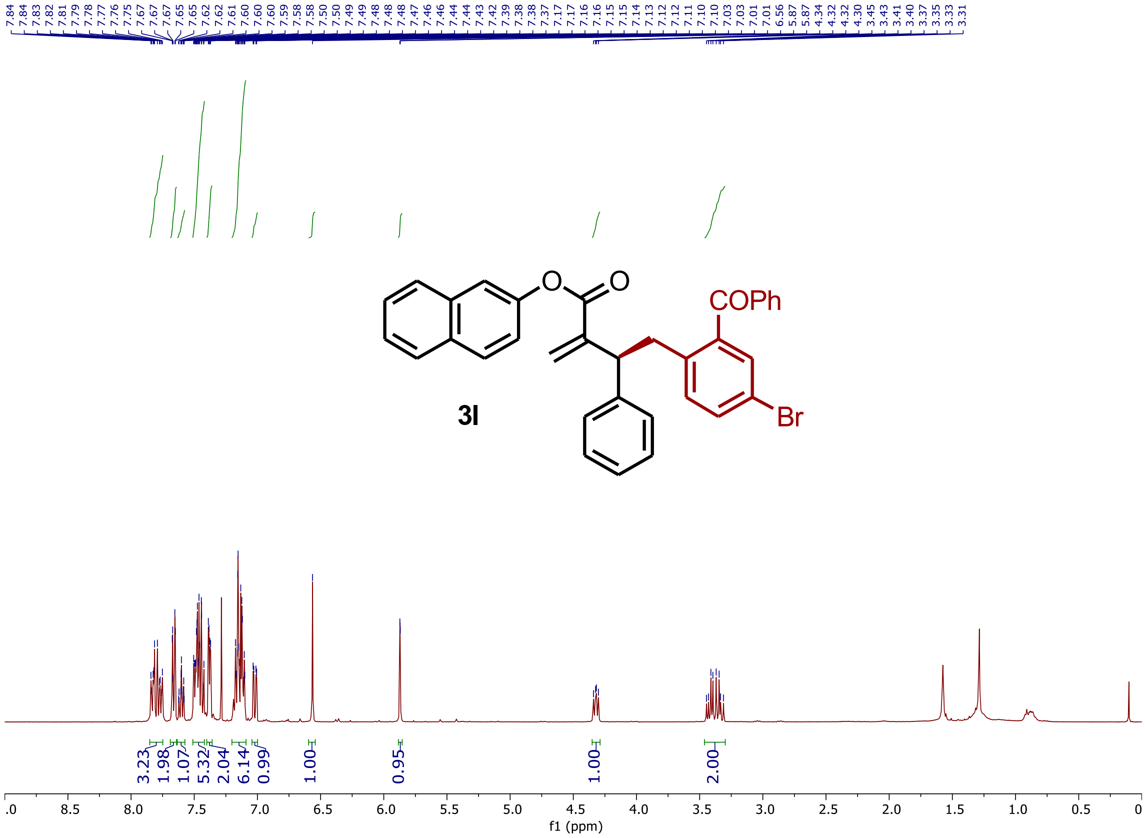

3l, ^{13}C NMR, 101 MHz, $CDCl_3$

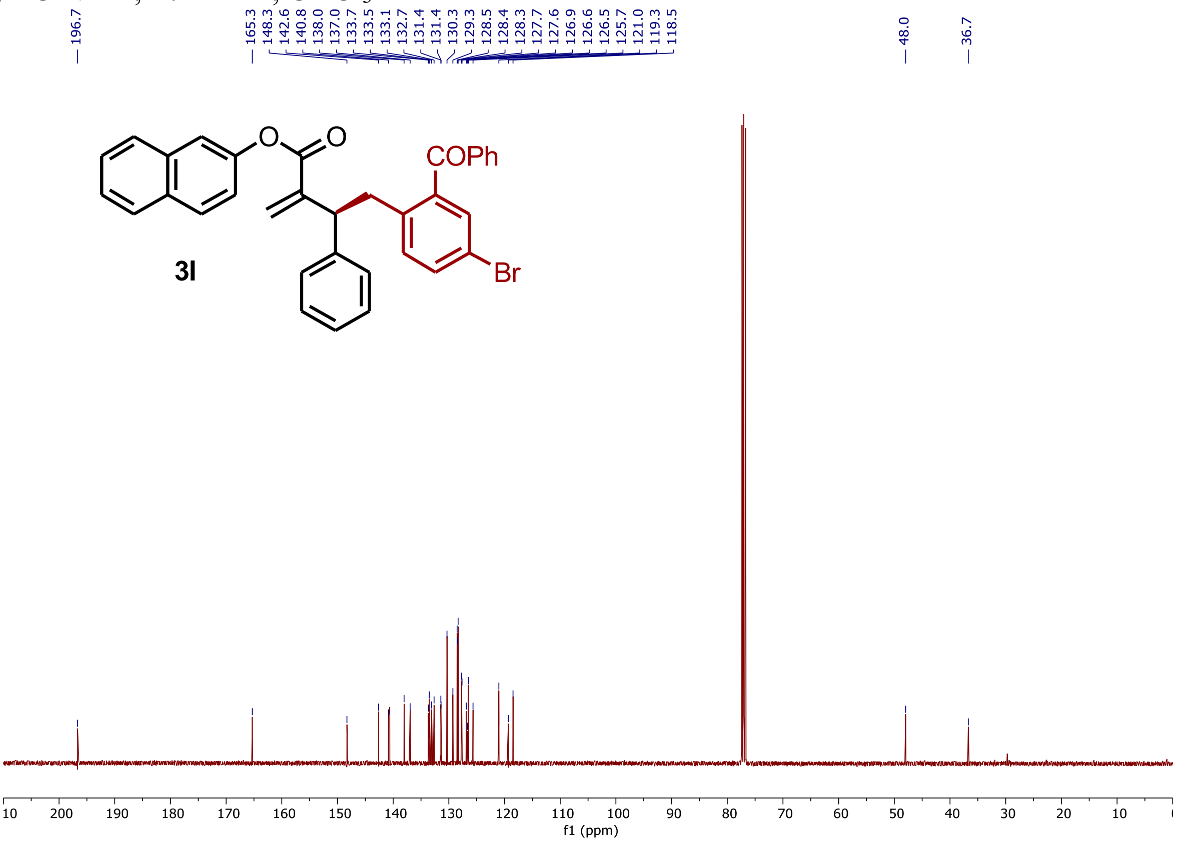

3m, ^{1}H NMR, 400 MHz, $CDCl_3$

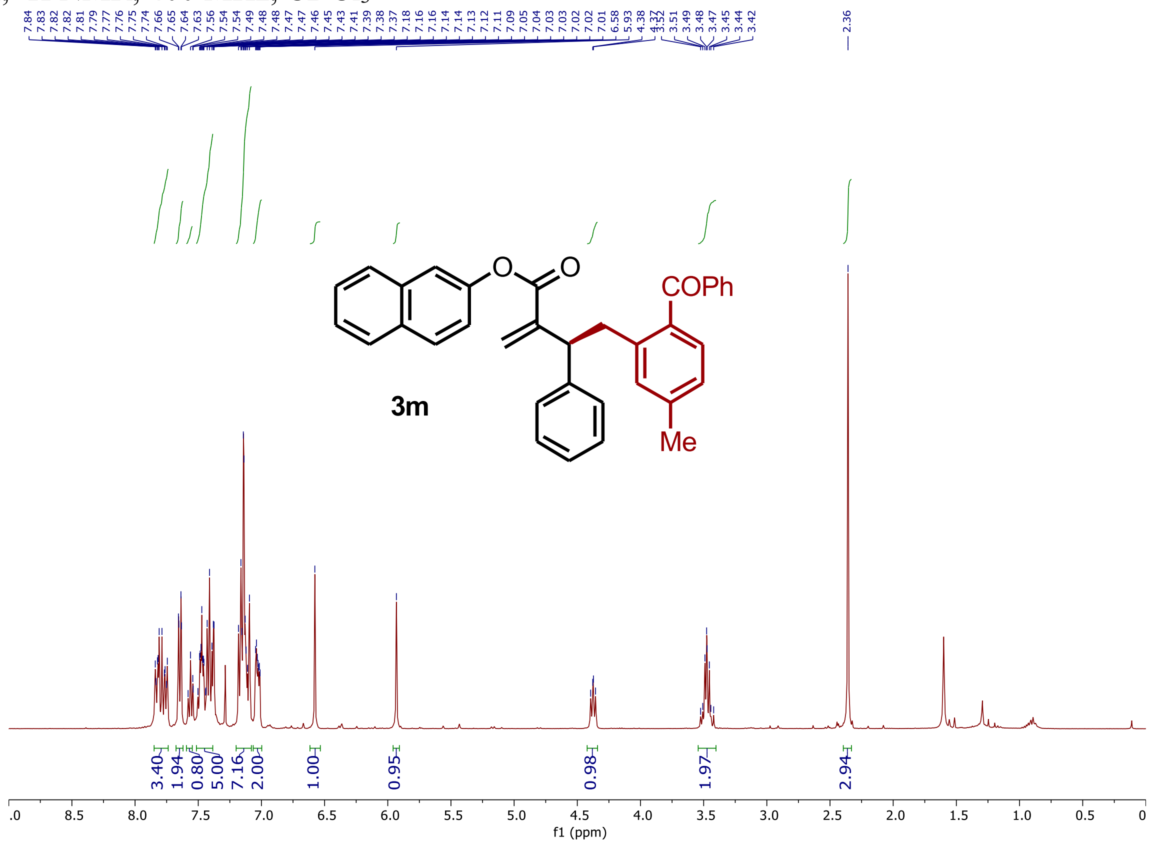

3m, ^{13}C NMR, 101 MHz, $CDCl_3$

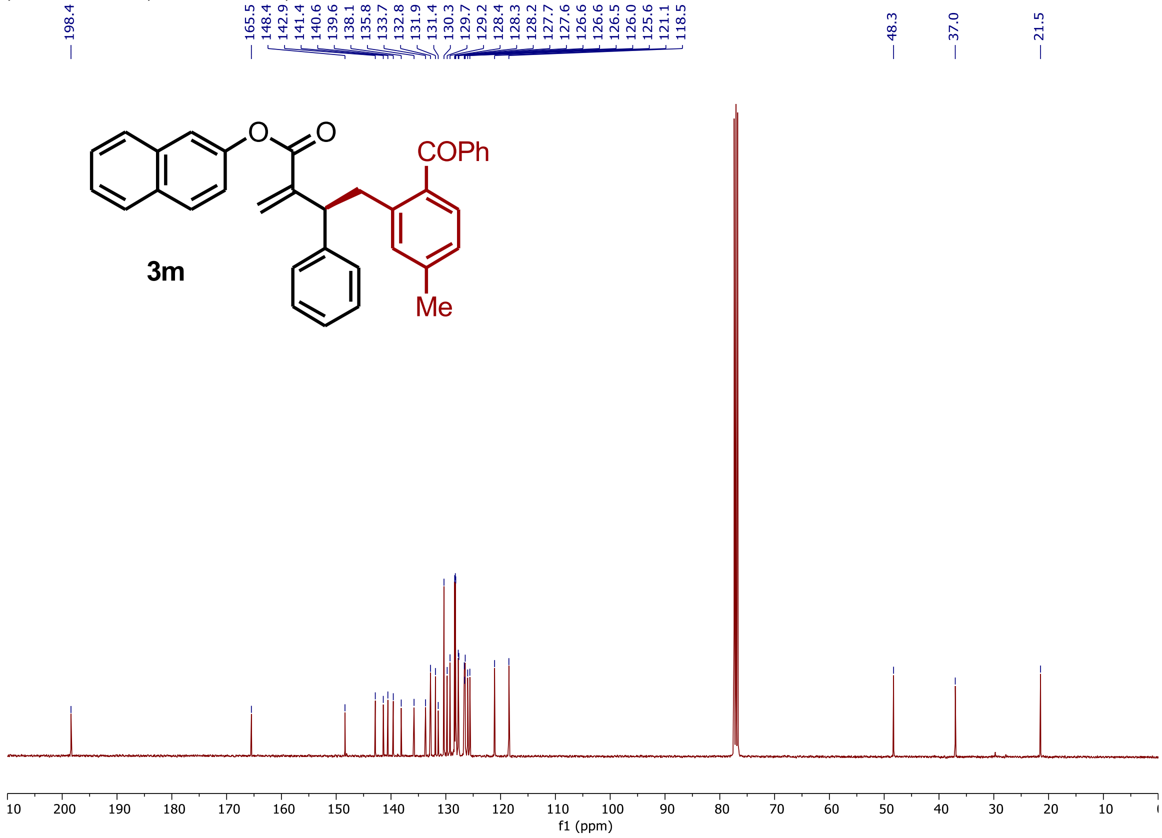

3n, ^{1}H NMR, 400 MHz, $CDCl_3$

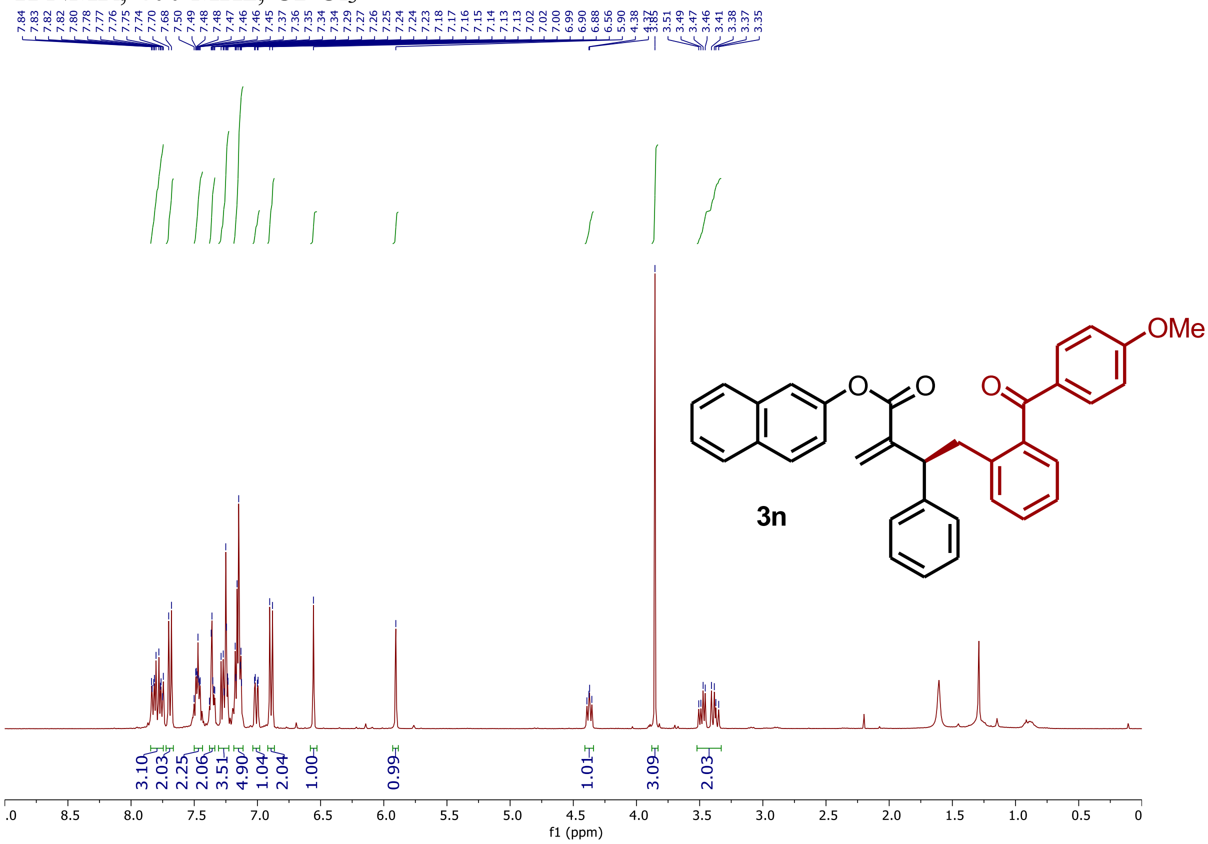

3n, ^{13}C NMR, 101 MHz, $CDCl_3$

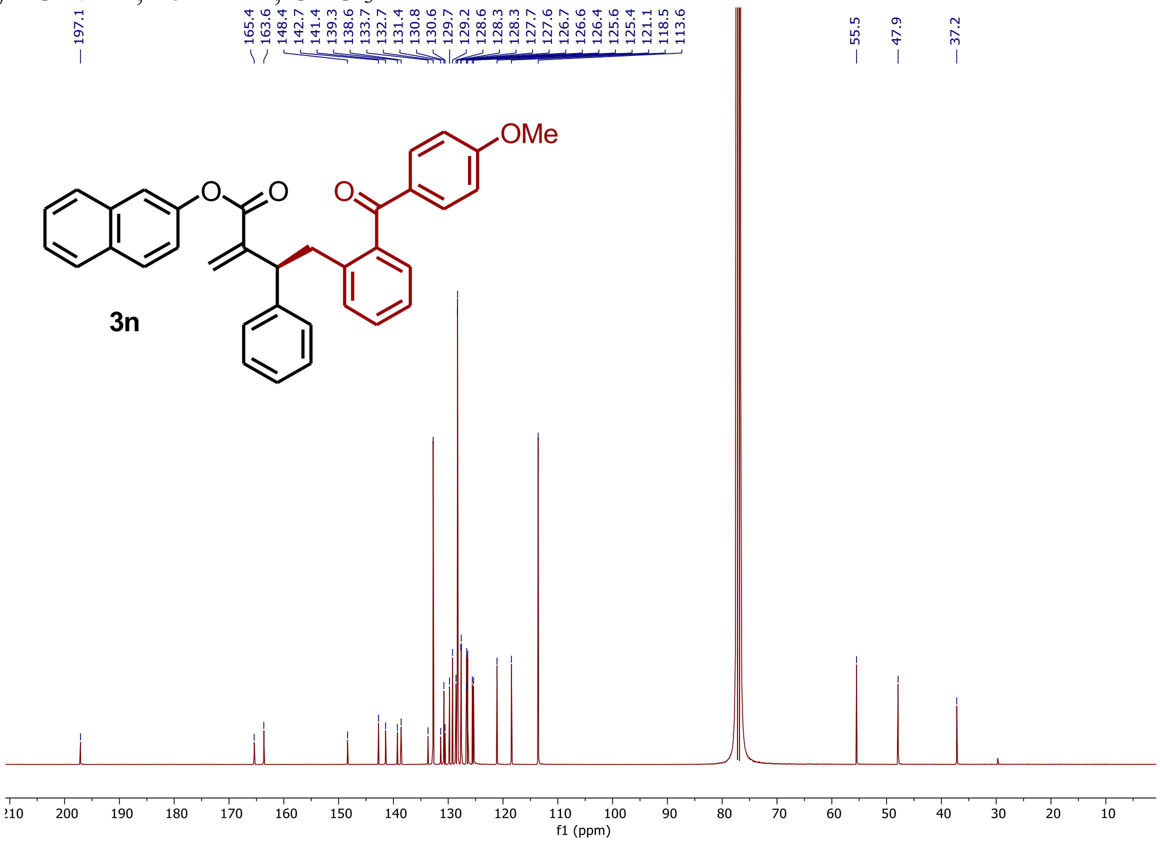

3o, ^{1}H NMR, 400 MHz, CDCl$_3$

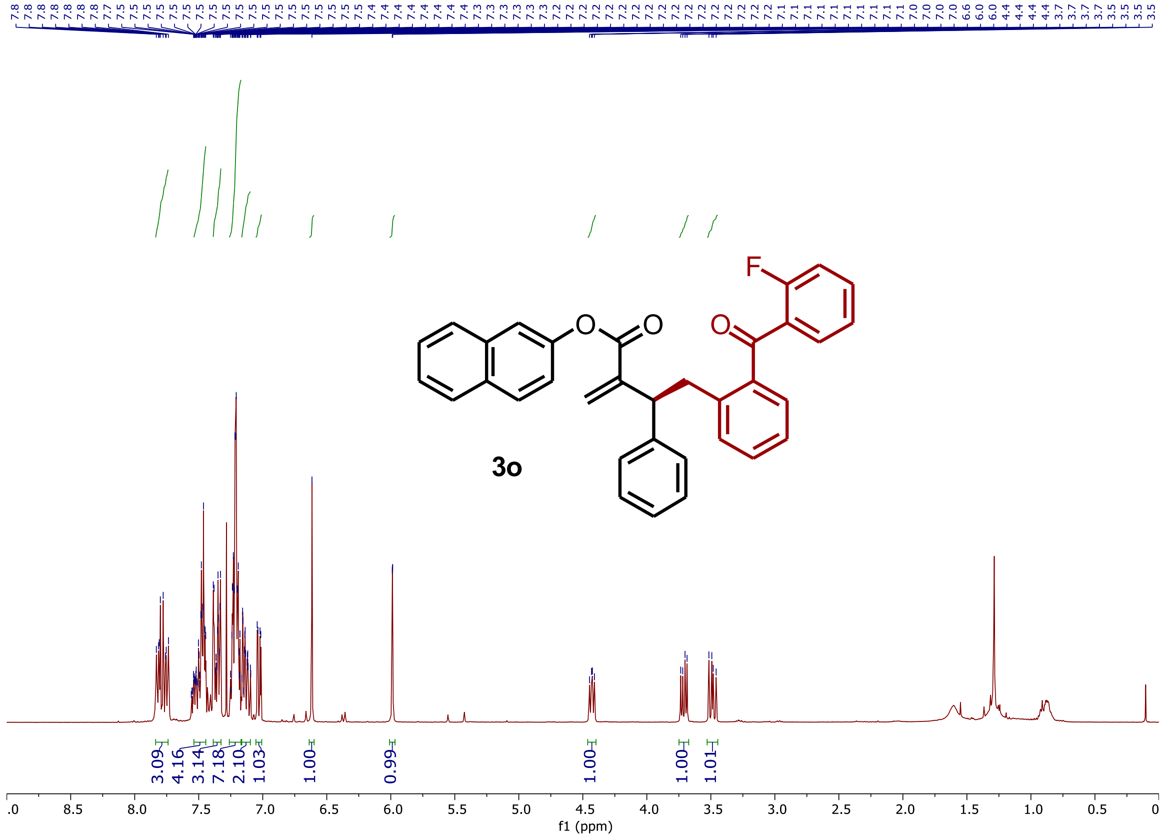

3o, ^{13}C NMR, 101 MHz, CDCl$_3$

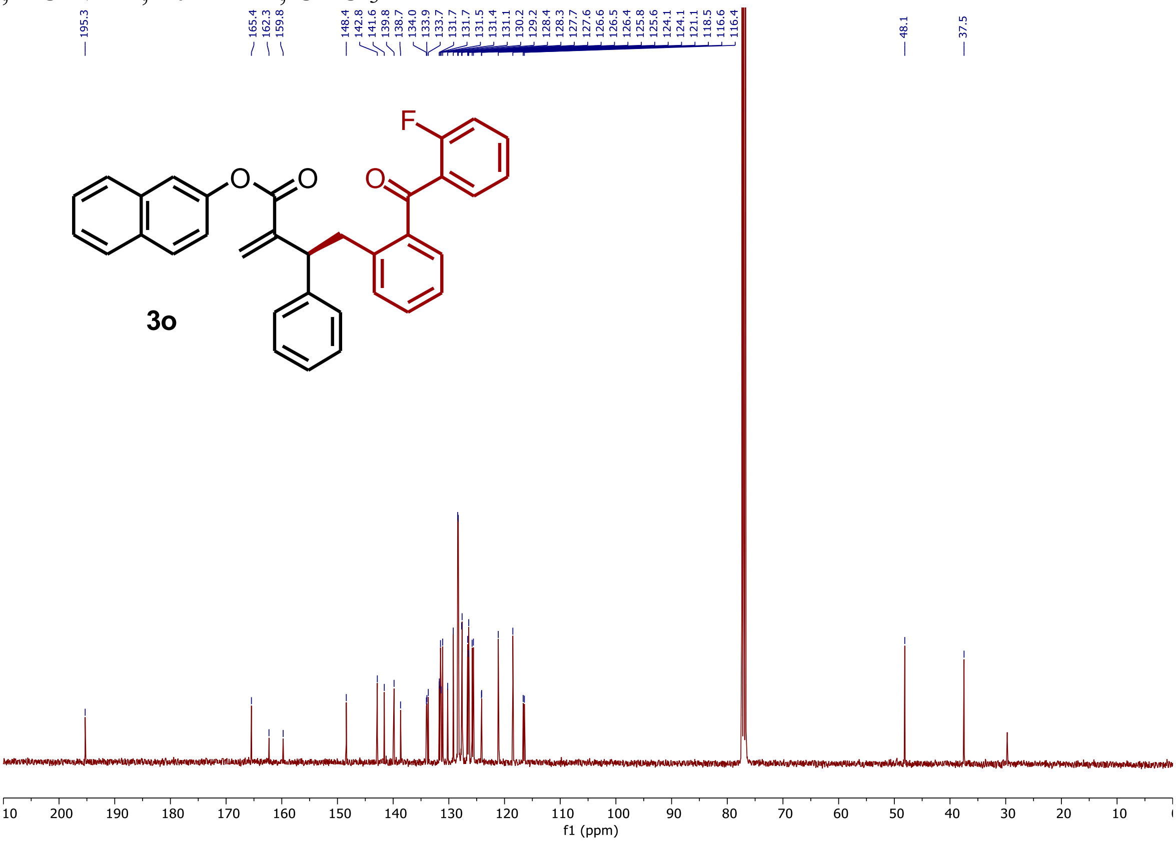

3o, ^{19}F NMR, 376 MHz, $CDCl_3$

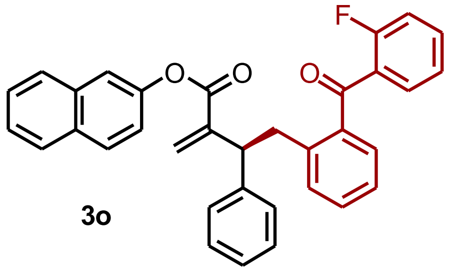

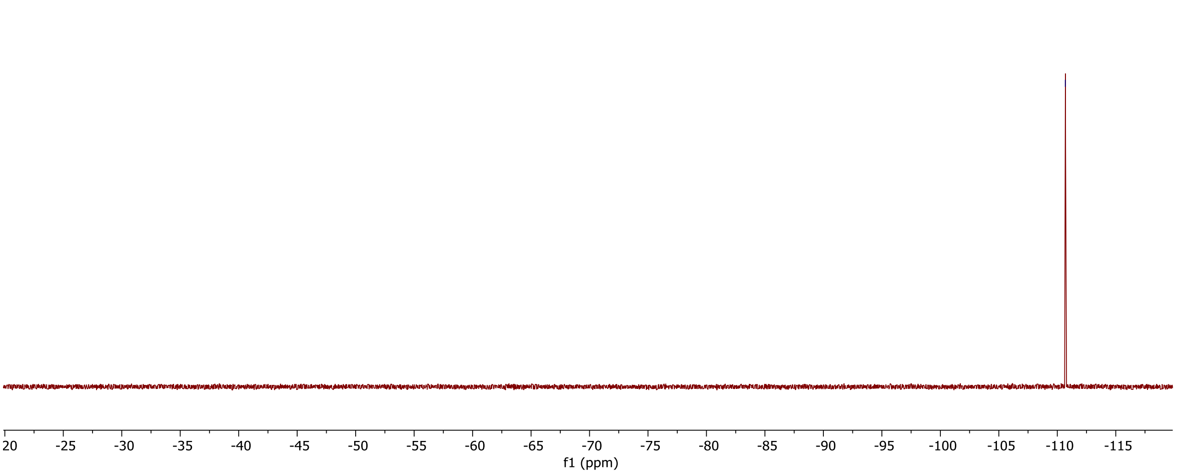

6a, ^{1}H NMR, 400 MHz, $CDCl_3$

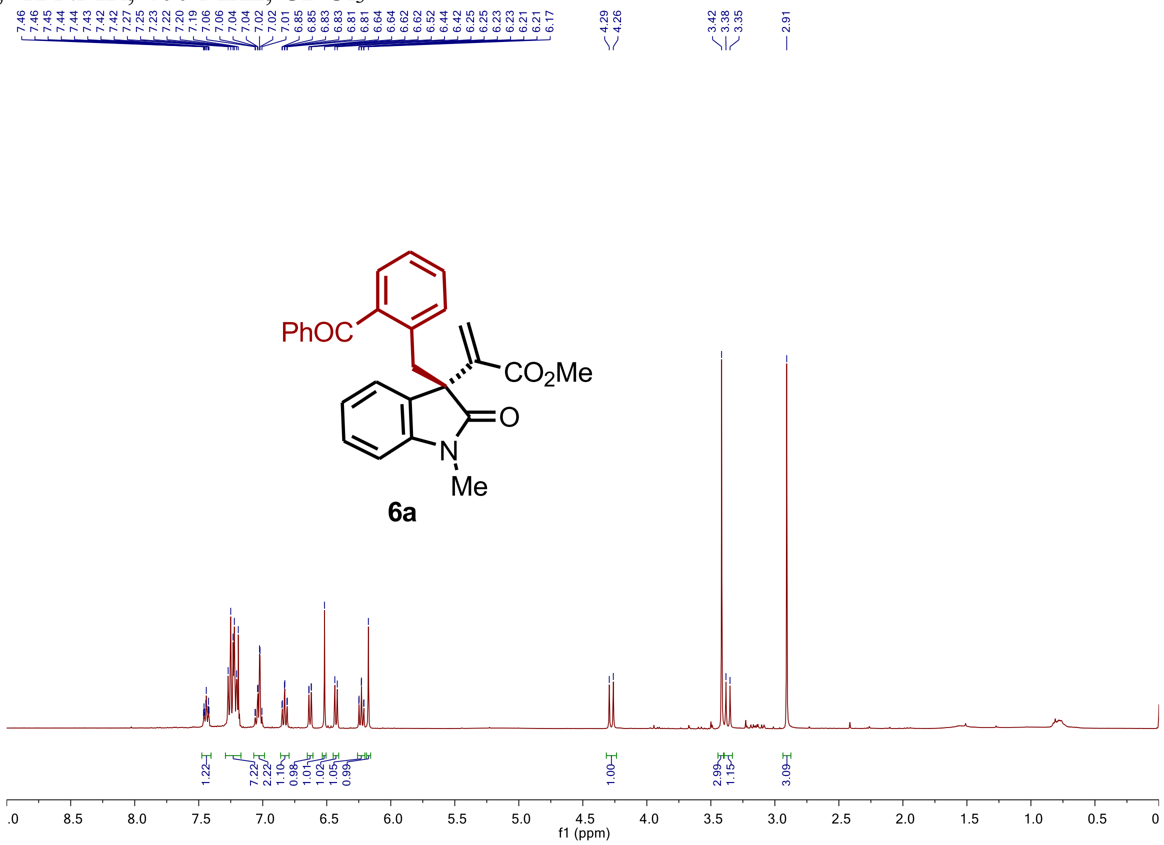

6a, ^{13}C NMR, 101 MHz, $CDCl_3$

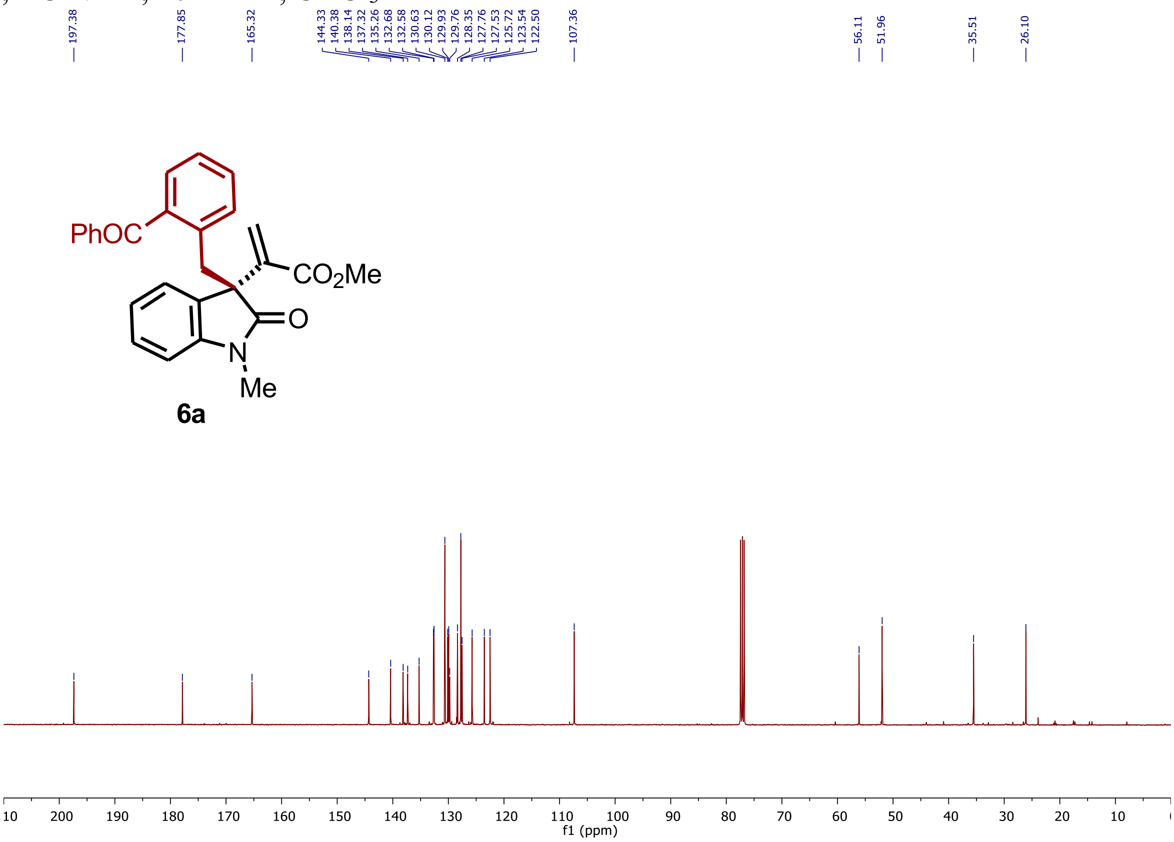

6b, ^{1}H NMR, 400 MHz, $CDCl_3$

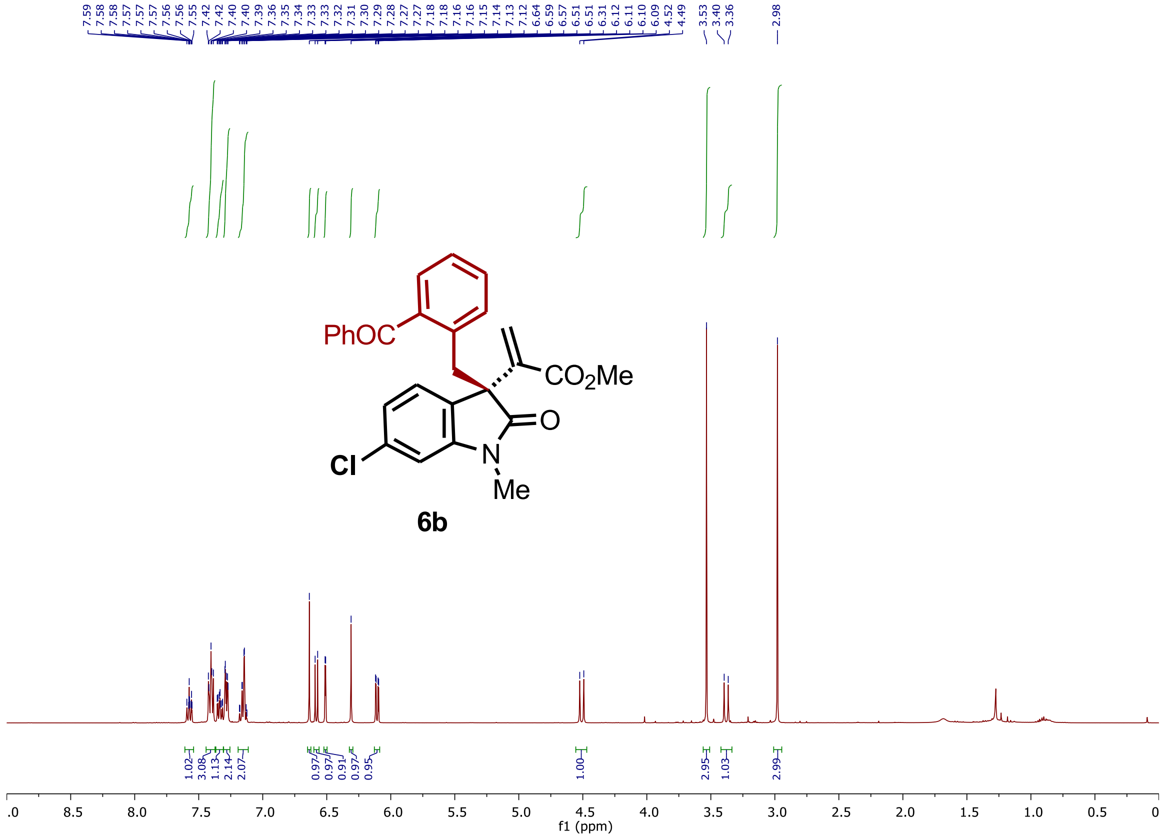

6b, ^{13}C NMR, 101 MHz, $CDCl_3$

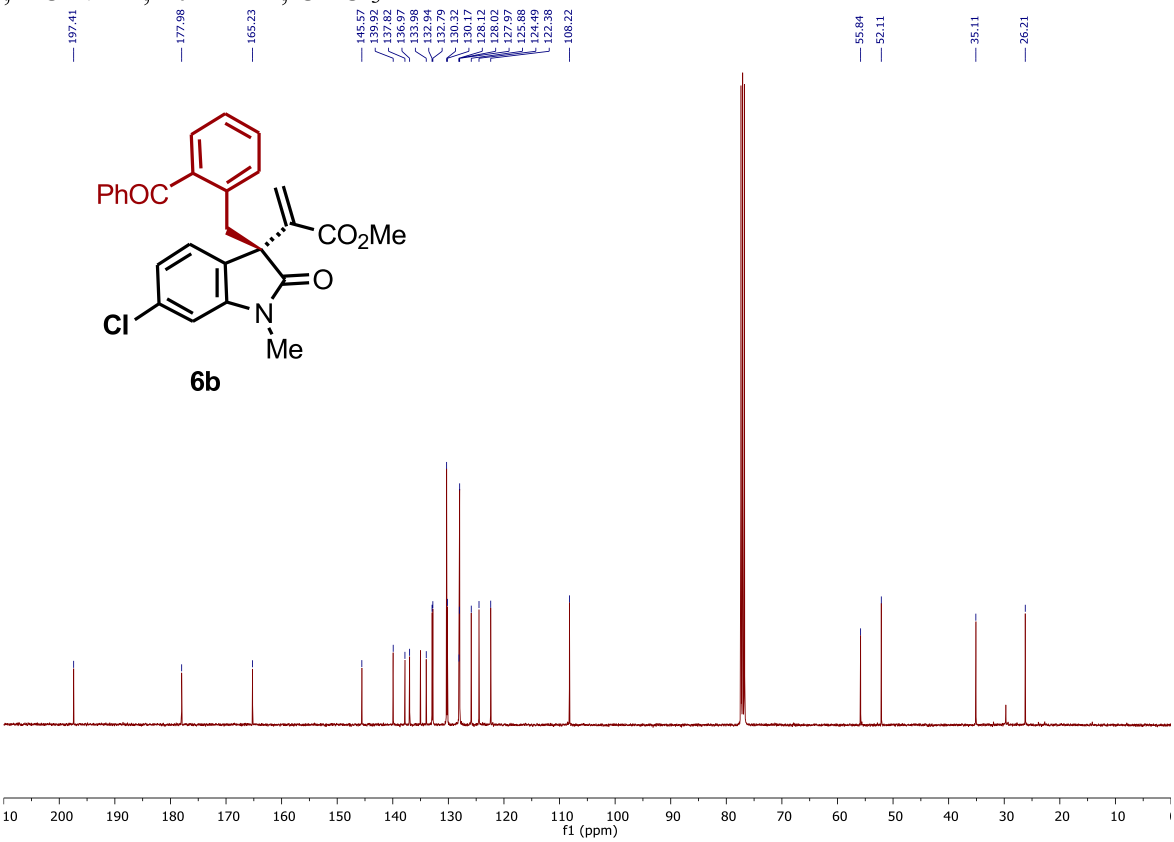

6c, ^{1}H NMR, 200 MHz, $CDCl_3$

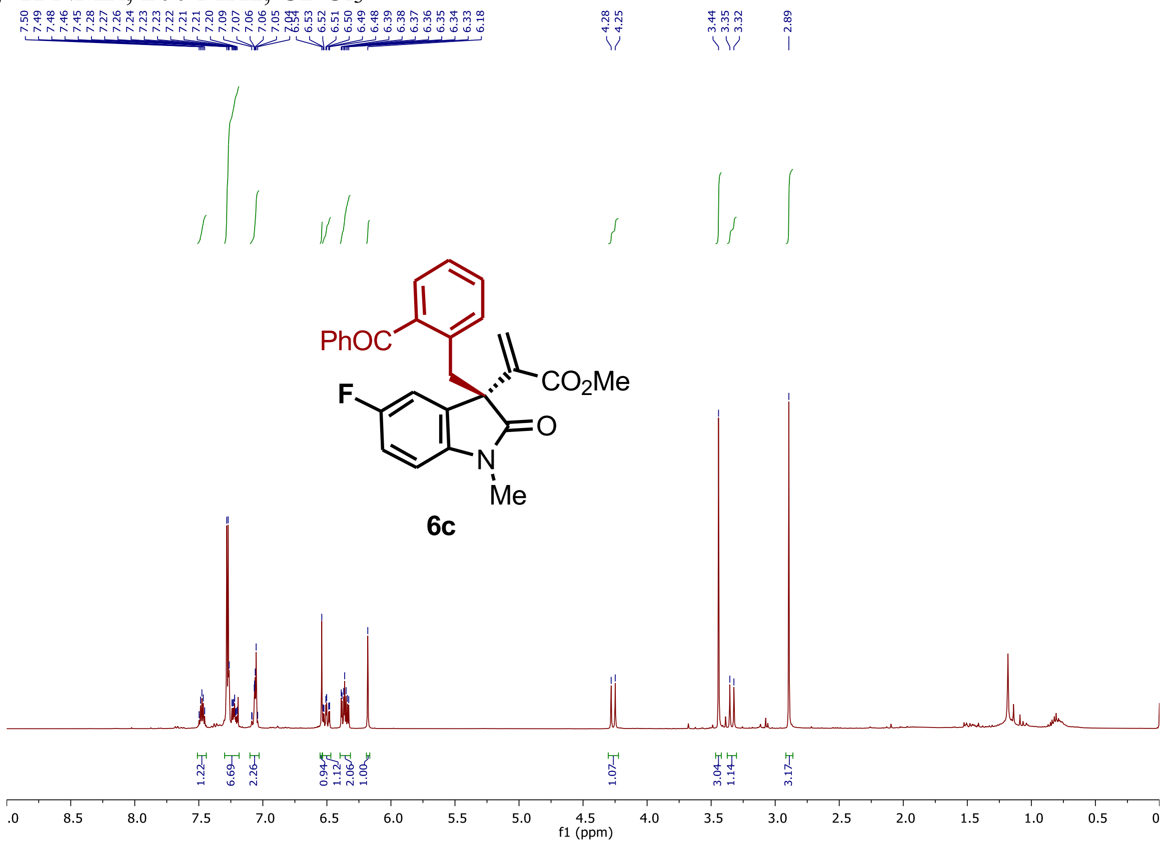

6c, ^{13}C NMR, 101 MHz, $CDCl_3$

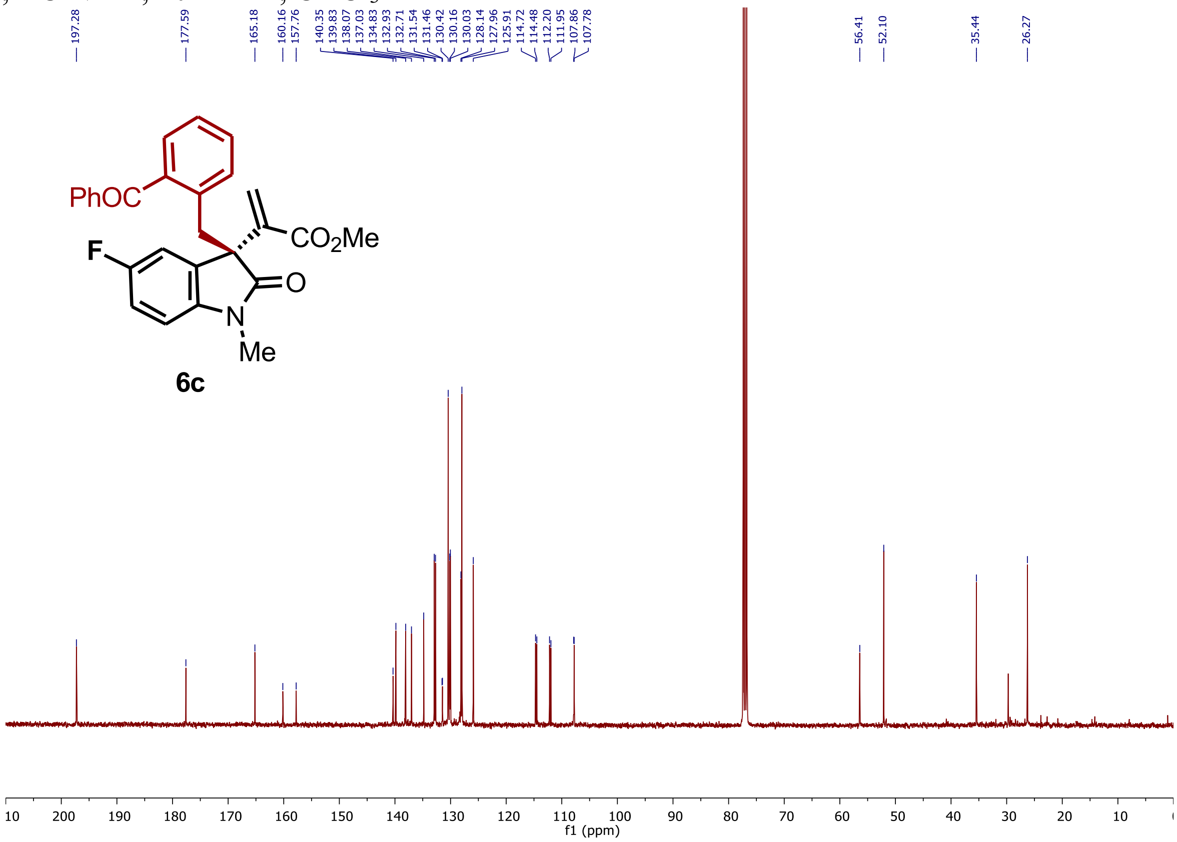

6c, ^{19}F NMR, 376 MHz, $CDCl_3$

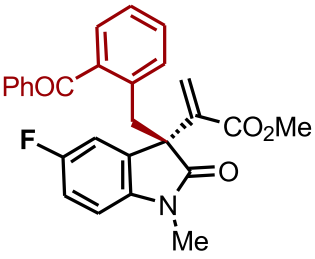

6c

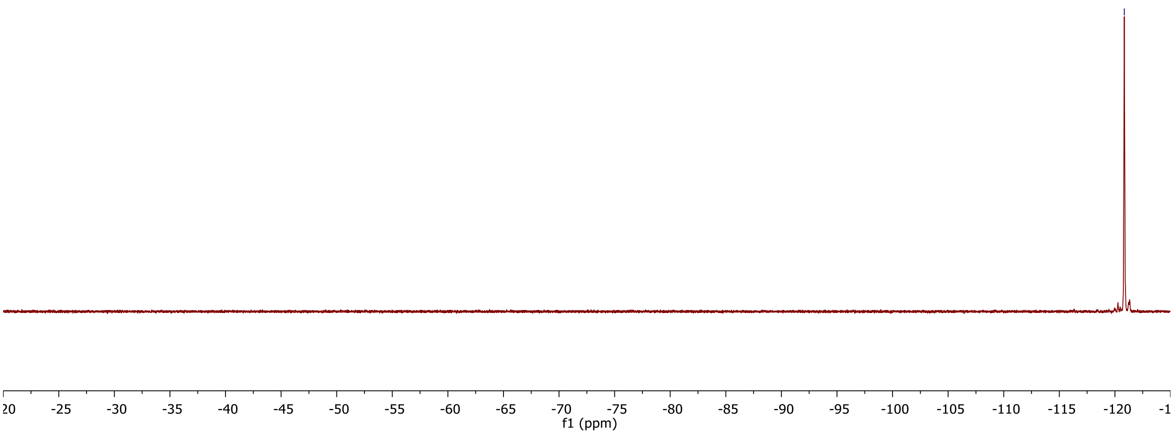

6d, ^{1}H NMR, 400 MHz, $CDCl_3$

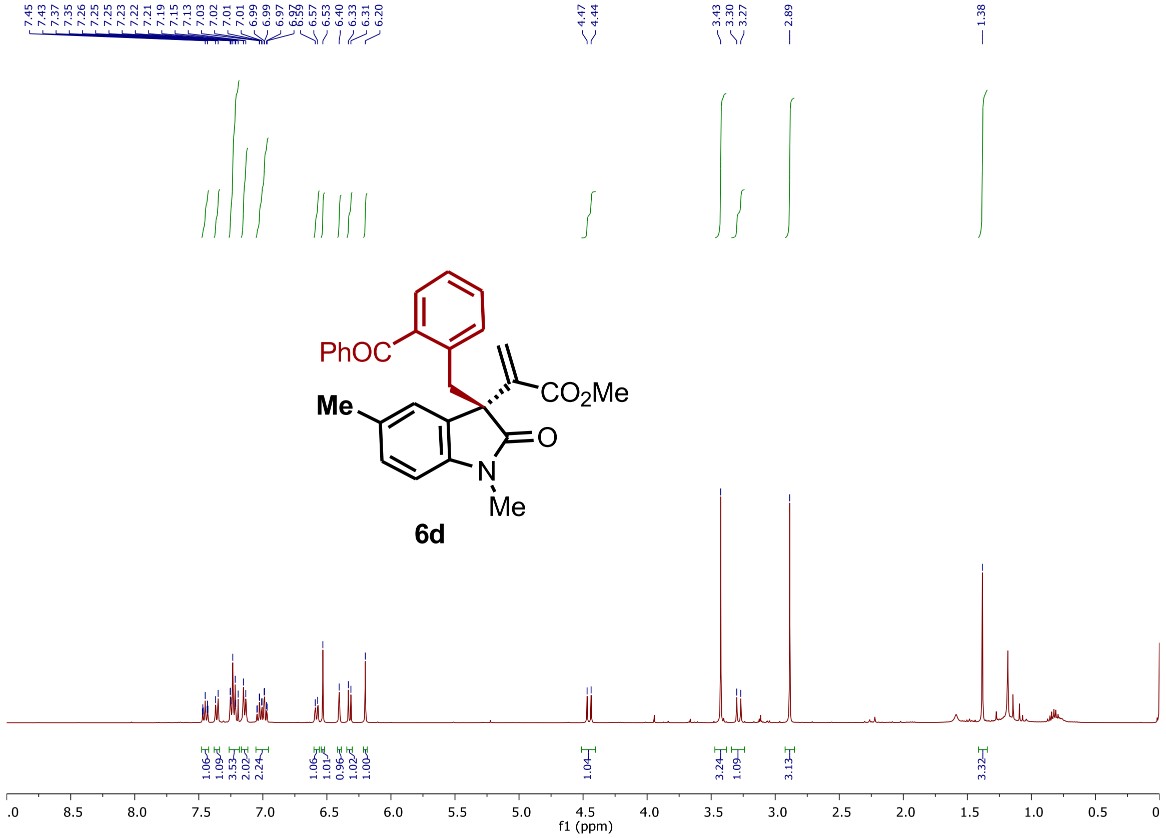

6d, ^{13}C NMR, 101 MHz, $CDCl_3$

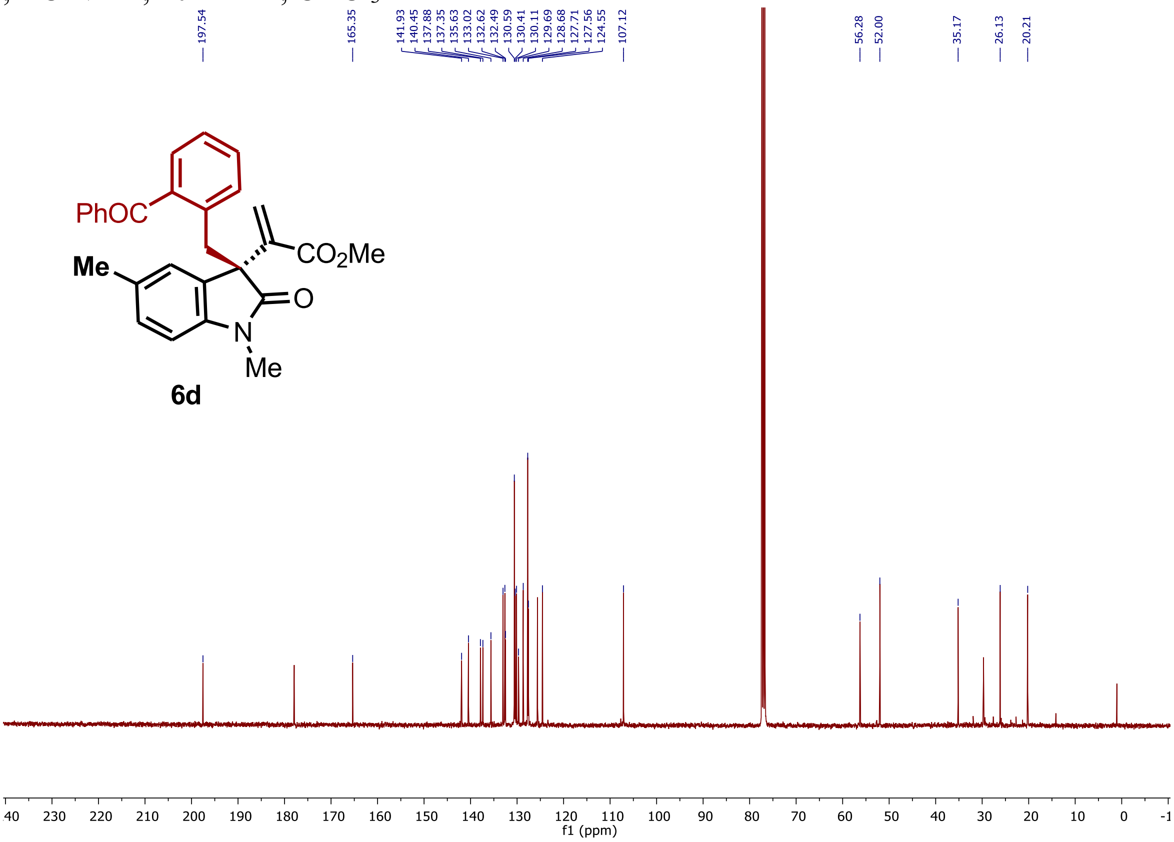

6e, ^{1}H NMR, 400 MHz, $CDCl_3$

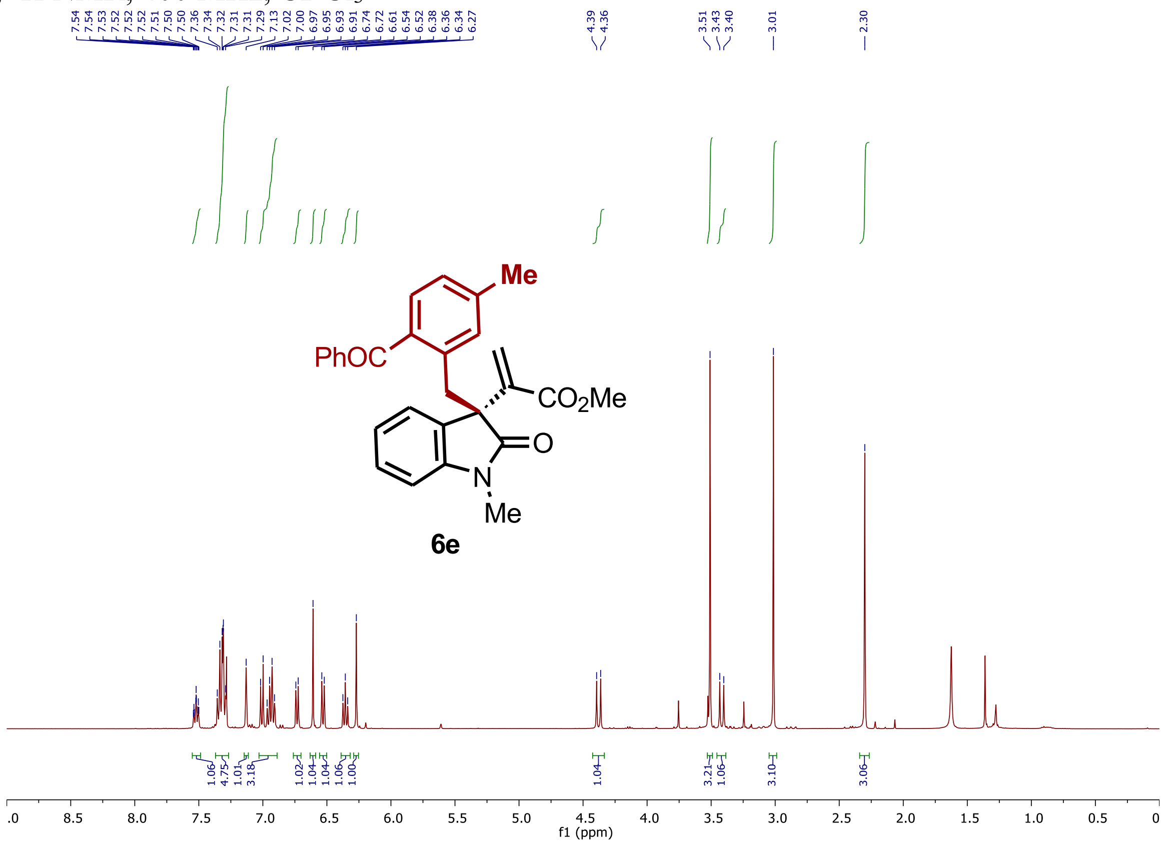

6e, ^{13}C NMR, 101 MHz, $CDCl_3$

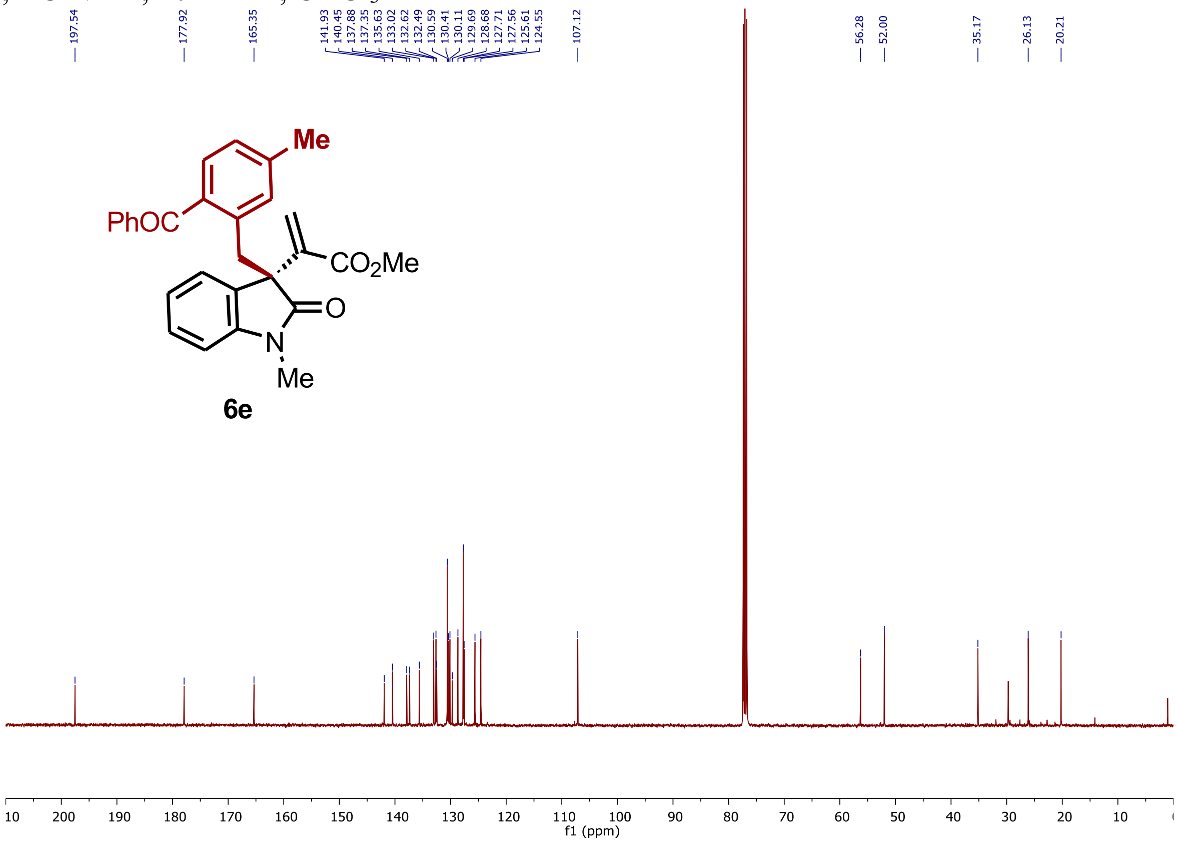

6g, ^{1}H NMR, 400 MHz, $CDCl_3$

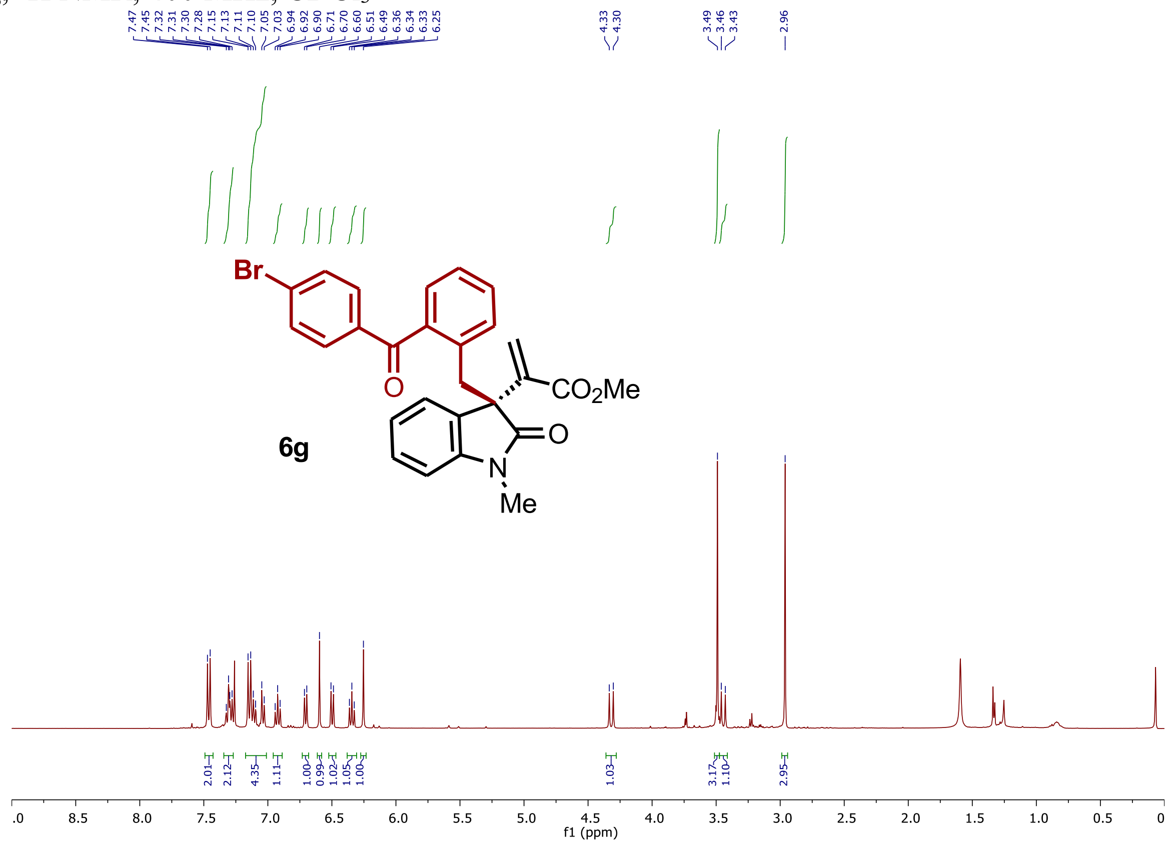

6g, ^{13}C NMR, 101 MHz, $CDCl_3$

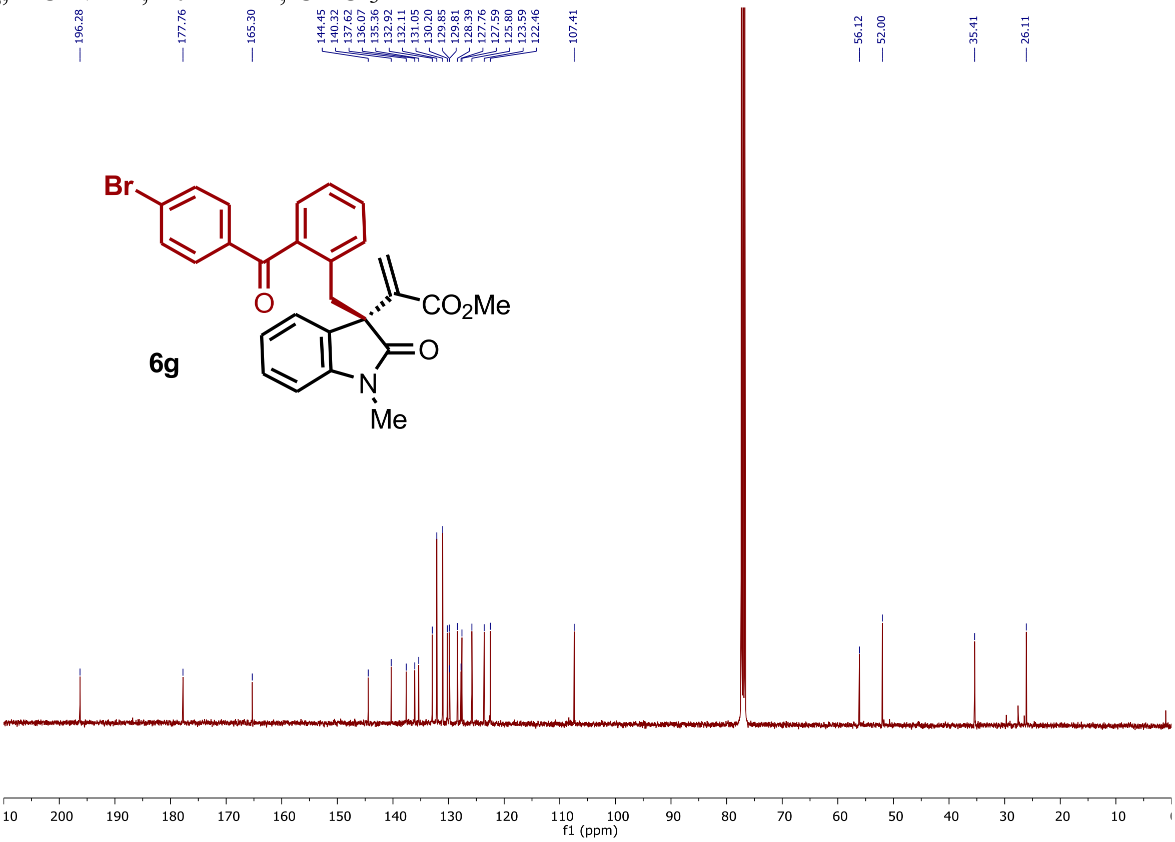

6f, ^{1}H NMR, 400 MHz, $CDCl_3$

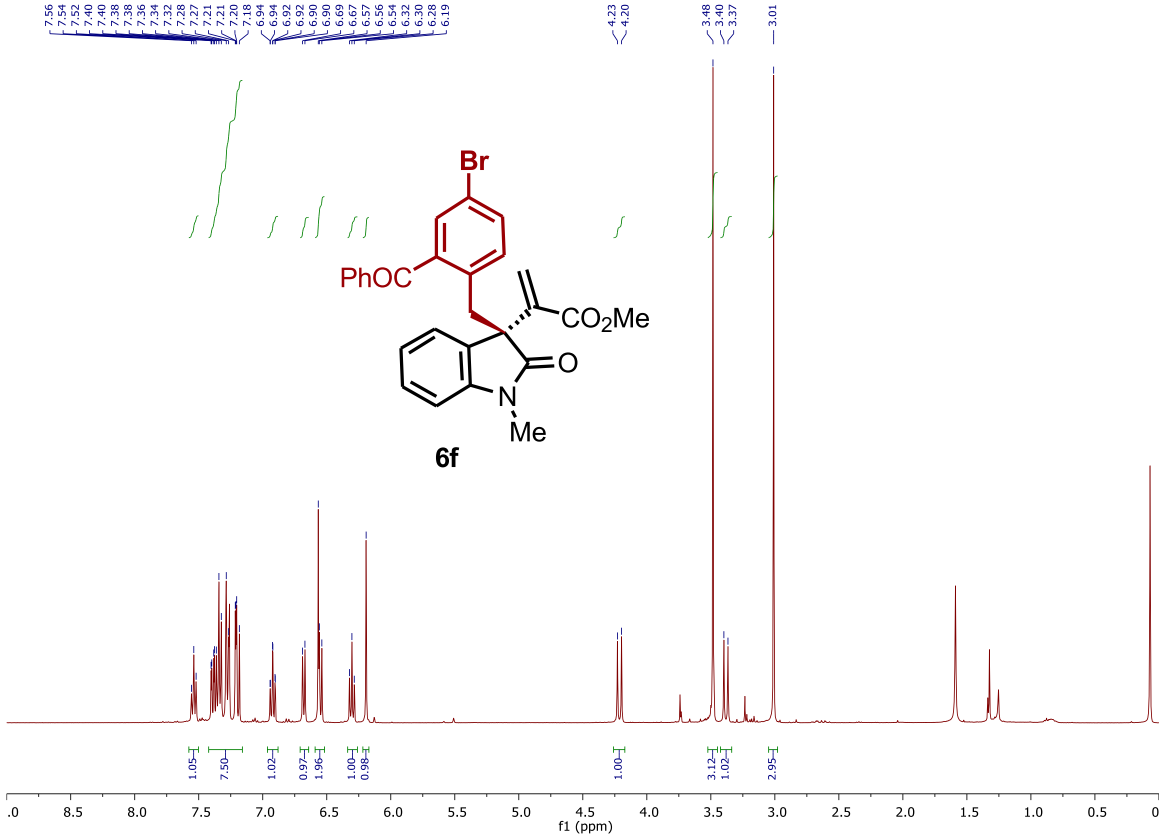

6f, ^{13}C NMR, 101 MHz, $CDCl_3$

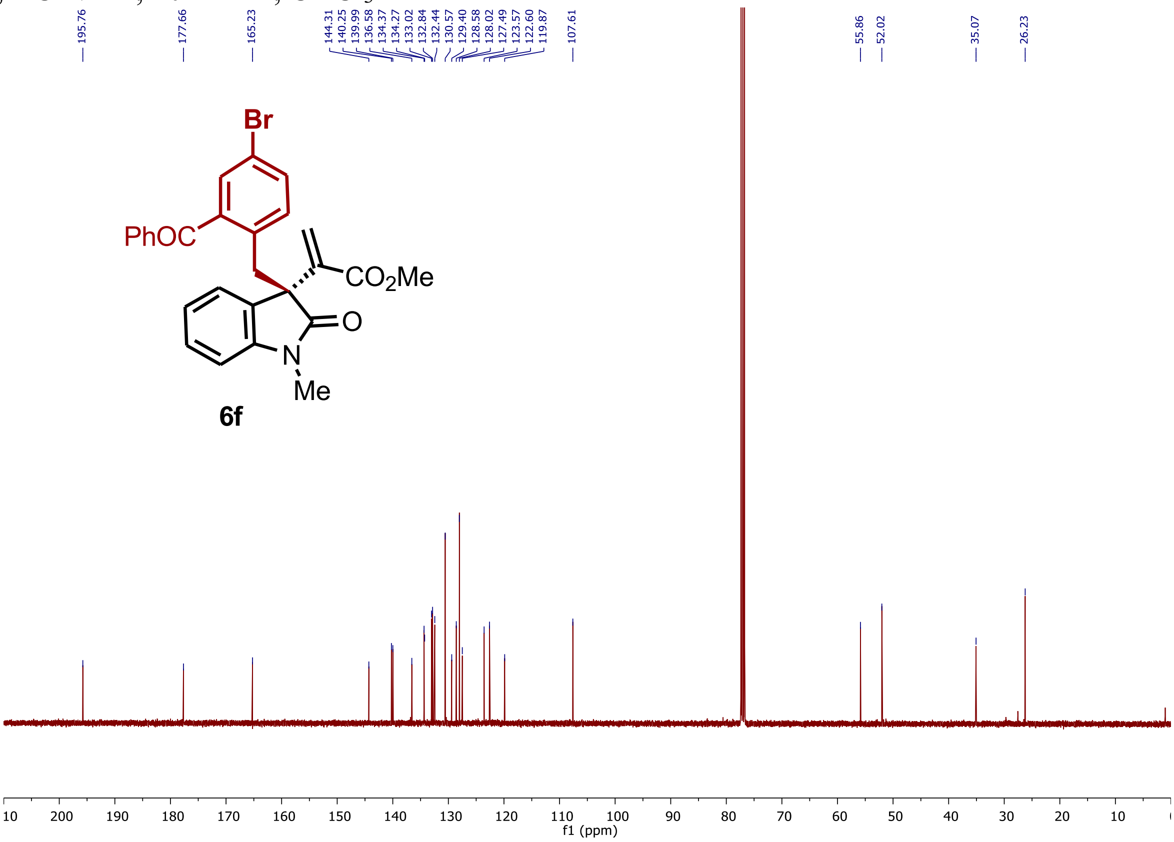

6h, ^{1}H NMR, 400 MHz, $CDCl_3$

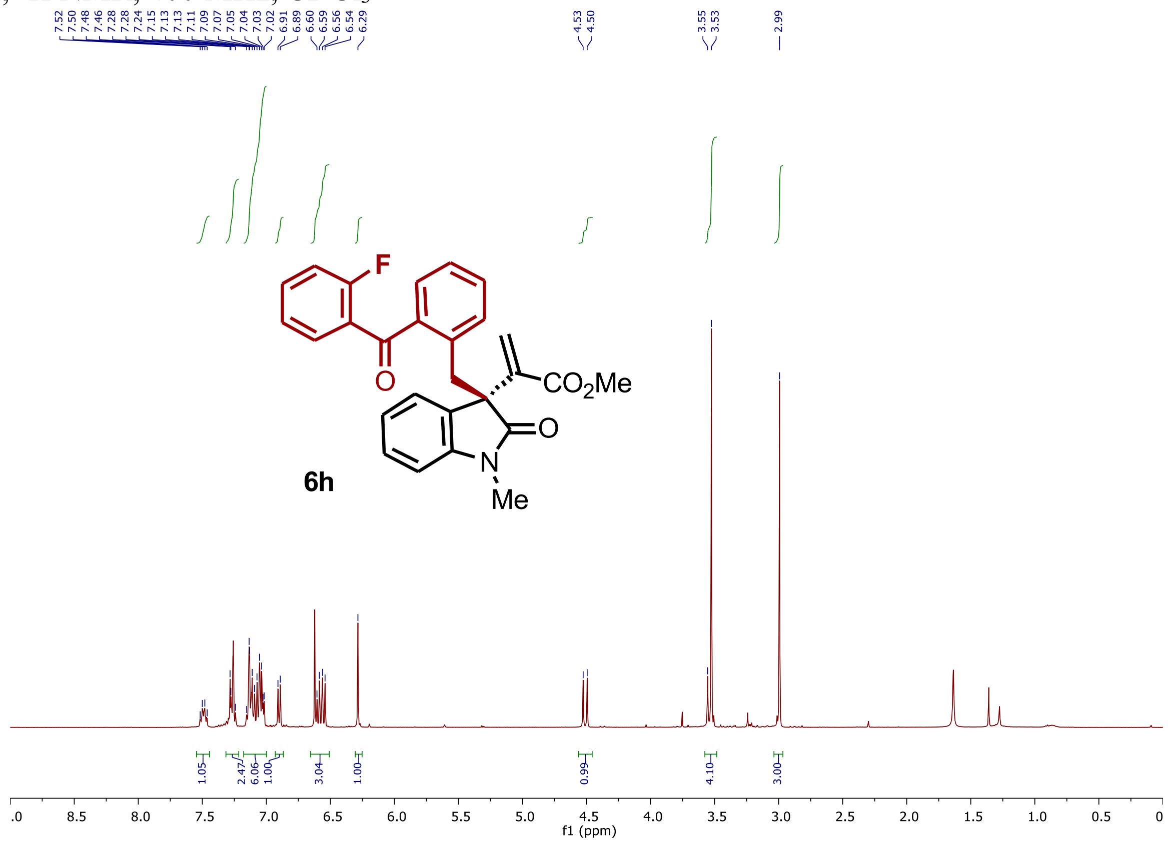

6h, ^{13}C NMR, 101 MHz, $CDCl_3$

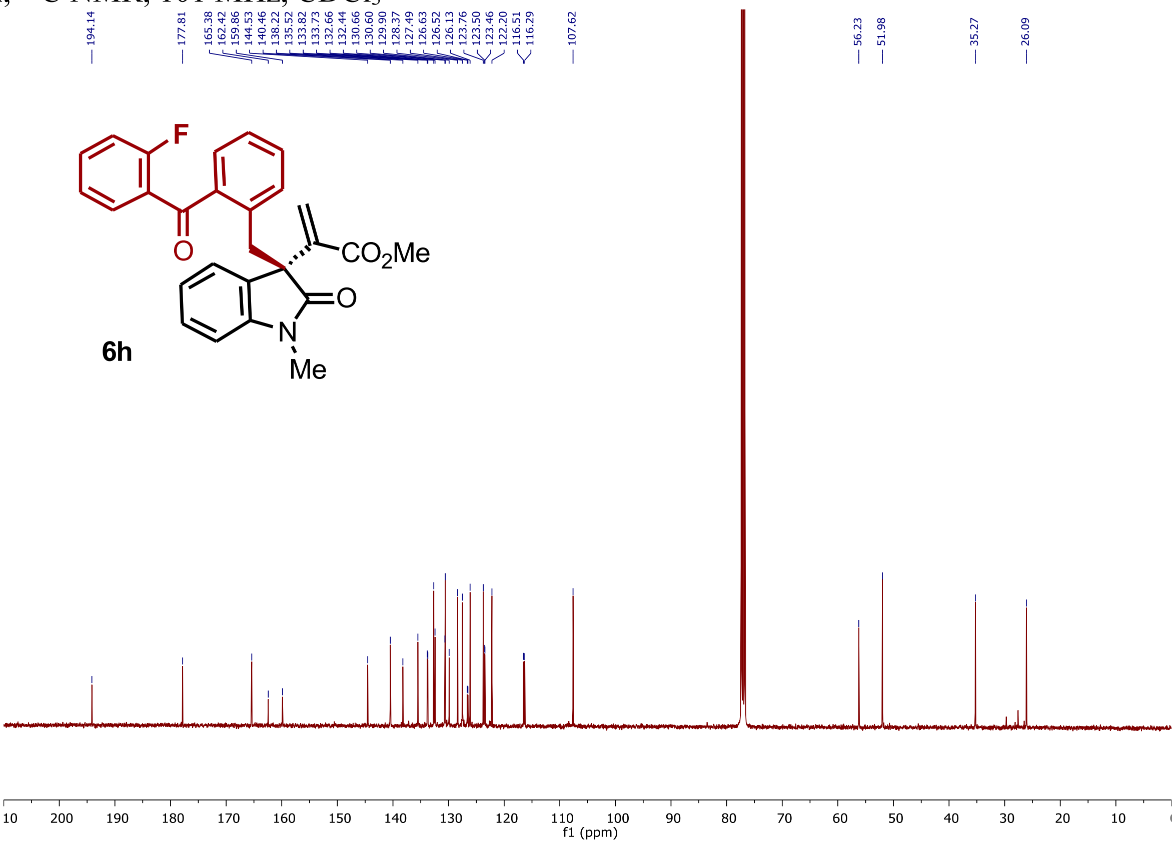

6h, ^{19}F NMR, 376 MHz, $CDCl_3$

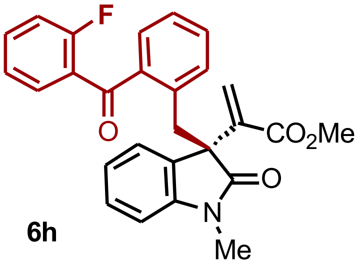

6h

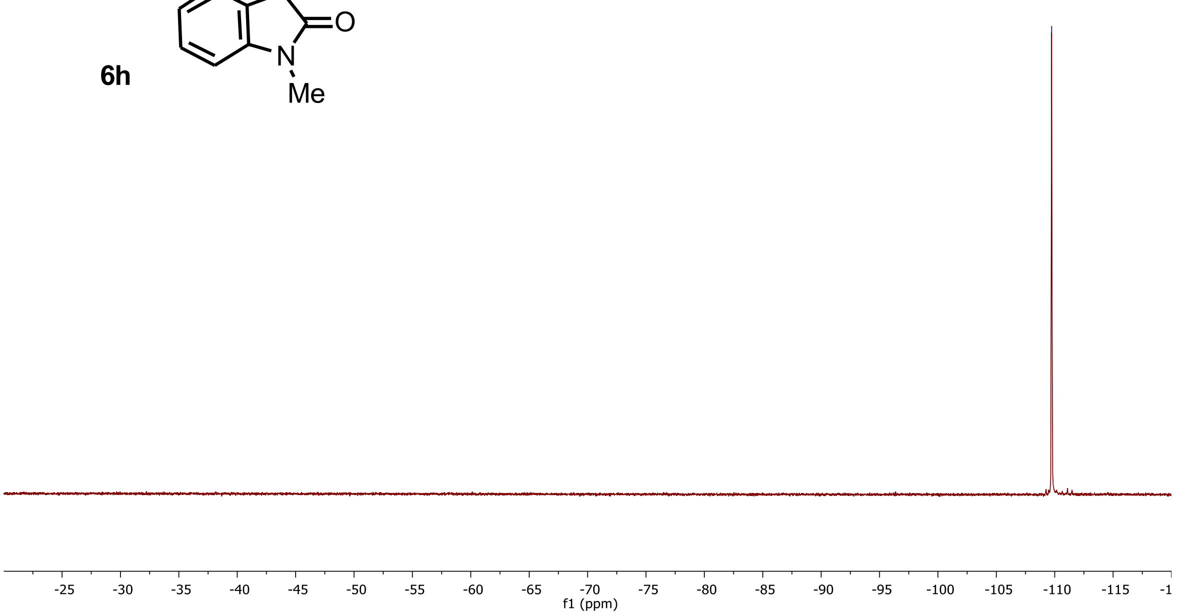

6i, ^{1}H NMR, 400 MHz, $CDCl_3$

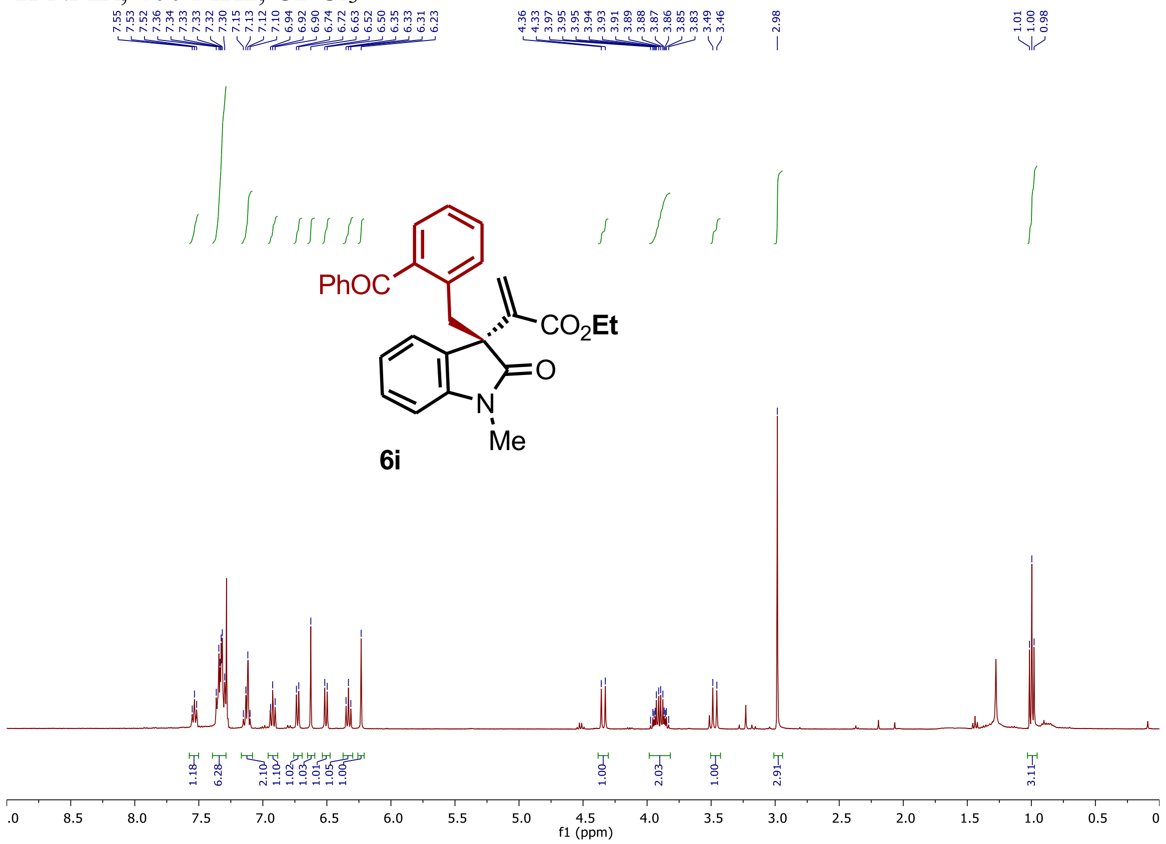

6i, ^{13}C NMR, 376 MHz, $CDCl_3$

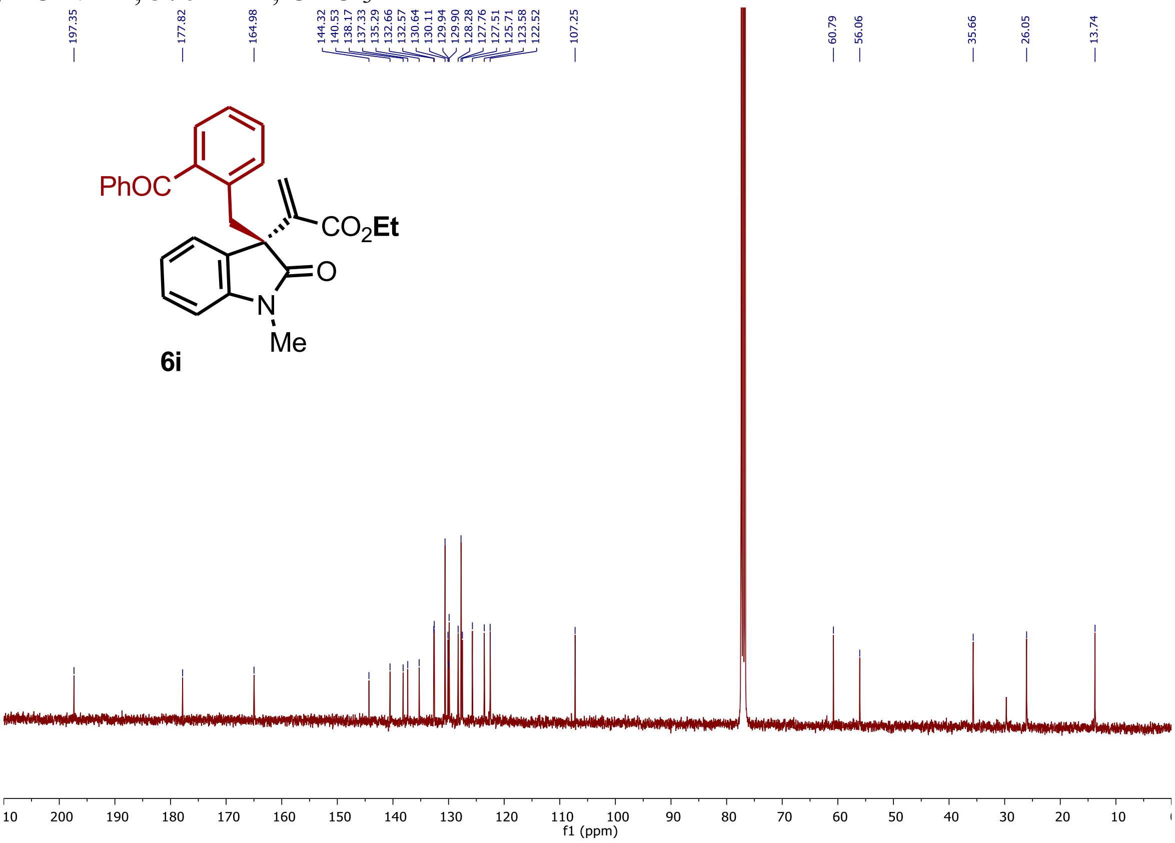

6j, ^{1}H NMR, 400 MHz, $CDCl_3$

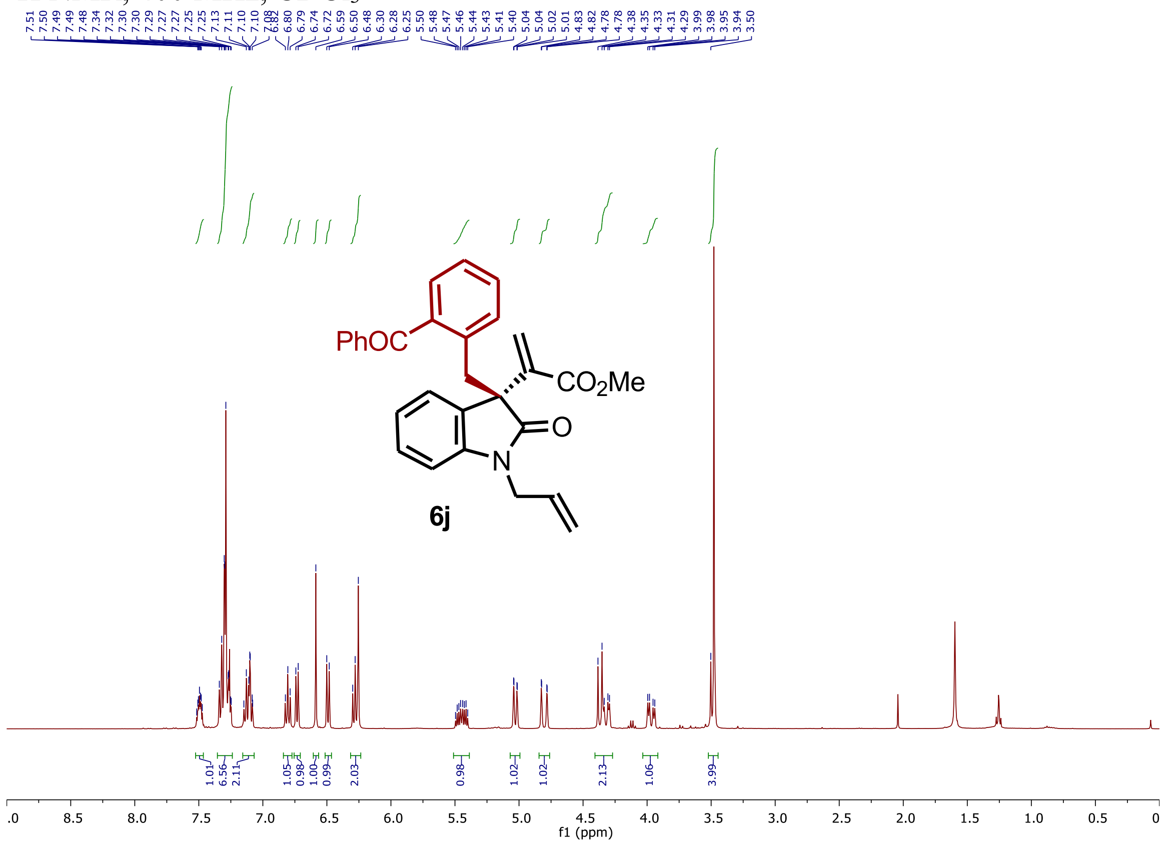

6j, ^{13}C NMR, 101 MHz, $CDCl_3$

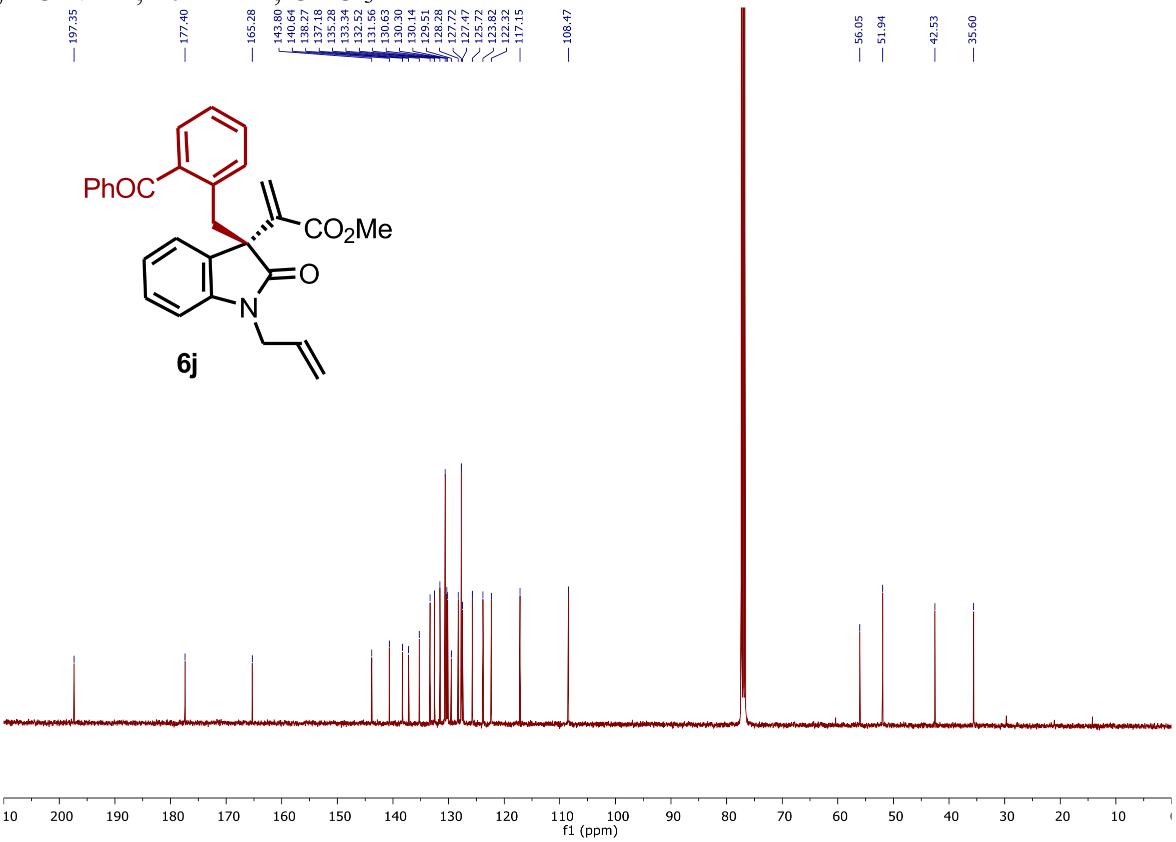

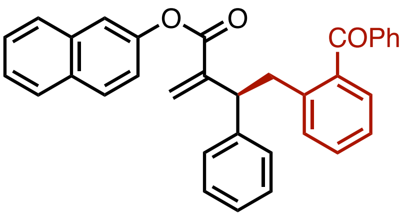

3g

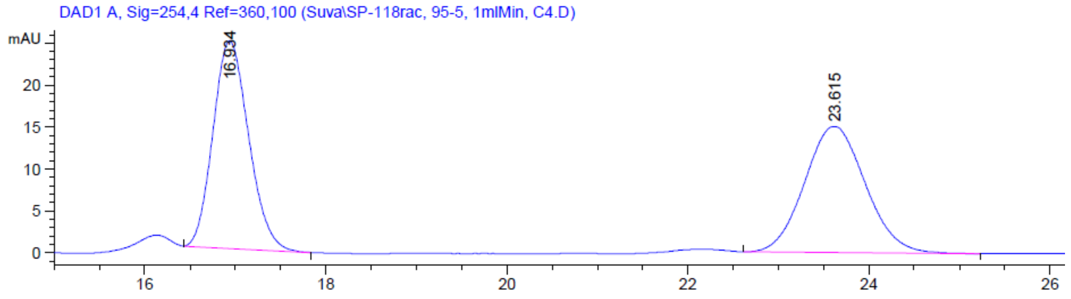

Peak #	RetTime [min]	Type	Width [min]	Area [mAU*s]	Height [mAU]	Area %
1	16.934	BB	0.4215	677.67737	24.74349	49.1535
2	23.615	BB	0.7172	701.01898	15.00156	50.8465

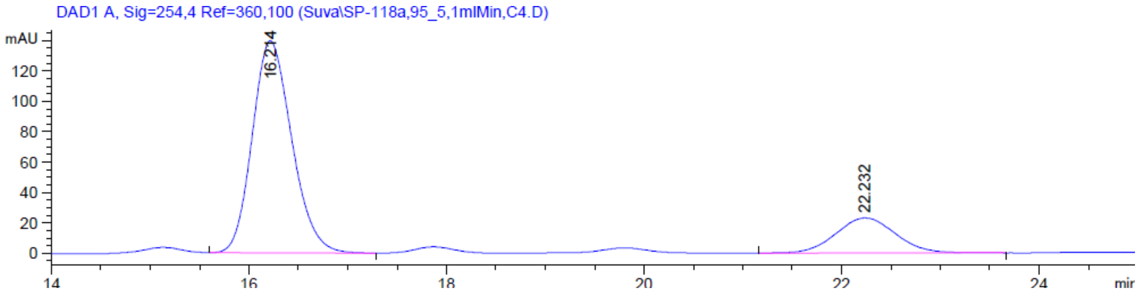

Peak #	RetTime [min]	Type	Width [min]	Area [mAU*s]	Height [mAU]	Area %
1	16.214	BB	0.4328	3862.41553	139.58955	80.0301
2	22.232	BB	0.6283	963.78638	23.02791	19.9699

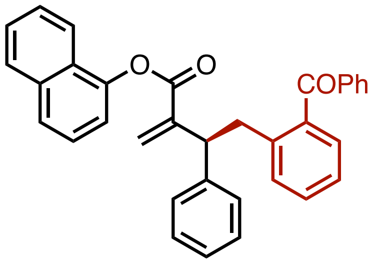

3f

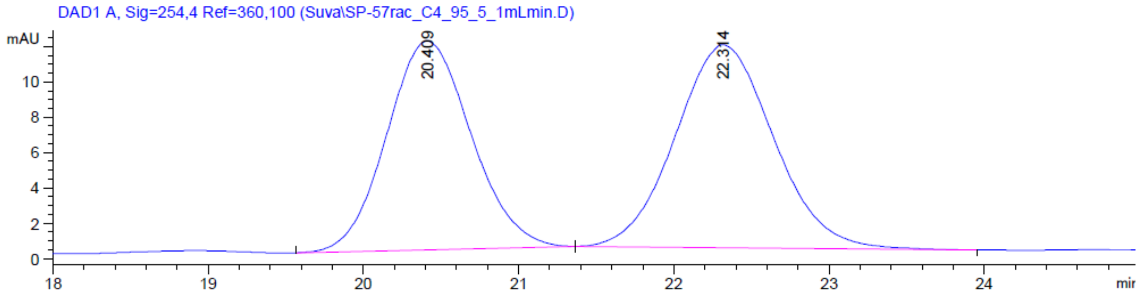

Peak #	RetTime [min]	Type	Width [min]	Area [mAU*s]	Height [mAU]	Area %
1	20.409	BB	0.5762	445.84631	11.81532	46.8566
2	22.314	BB	0.6598	505.66614	11.48290	53.1434

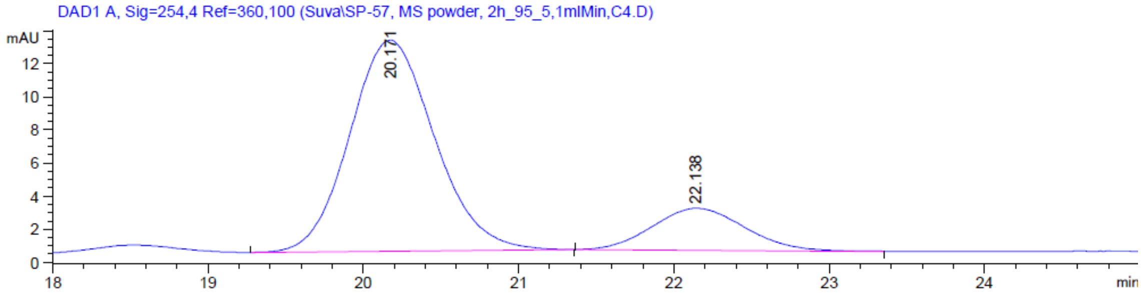

Peak #	RetTime [min]	Type	Width [min]	Area [mAU*s]	Height [mAU]	Area %
1	20.171	BB	0.5791	484.18054	12.74788	82.2421
2	22.138	BB	0.6013	104.54507	2.53419	17.7579

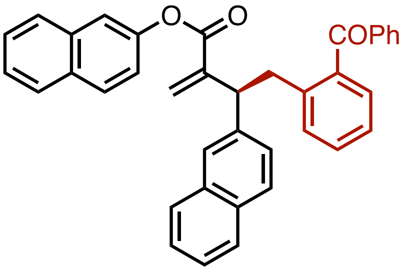

3h

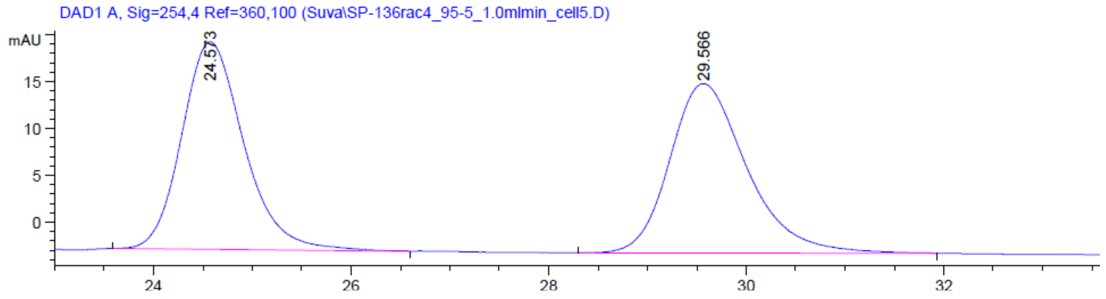

Peak #	RetTime [min]	Type	Width [min]	Area [mAU*s]	Height [mAU]	Area %
1	24.573	BB	0.6695	967.26190	22.14671	49.6593
2	29.566	BB	0.8046	980.53552	18.08253	50.3407

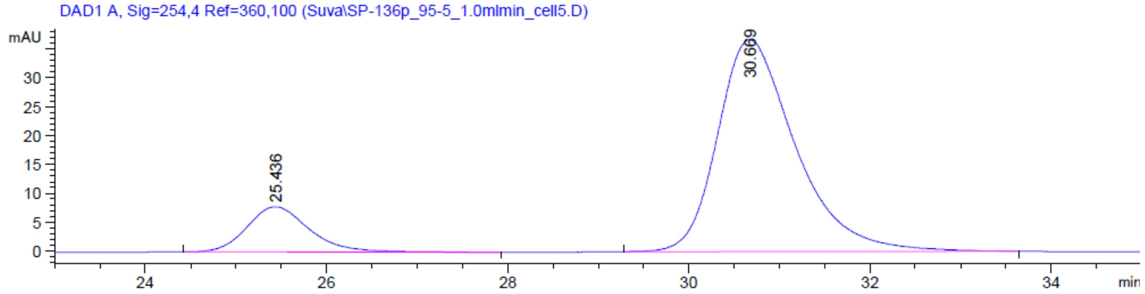

Peak #	RetTime [min]	Type	Width [min]	Area [mAU*s]	Height [mAU]	Area %
1	25.436	BB	0.6991	377.30524	7.84463	14.6958
2	30.669	BB	0.8662	2190.12549	36.70011	85.3042

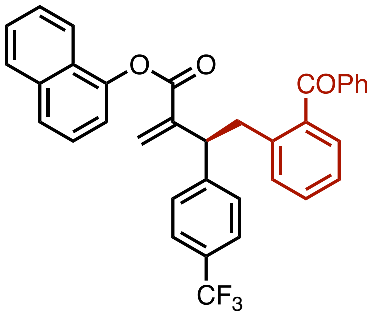

3i

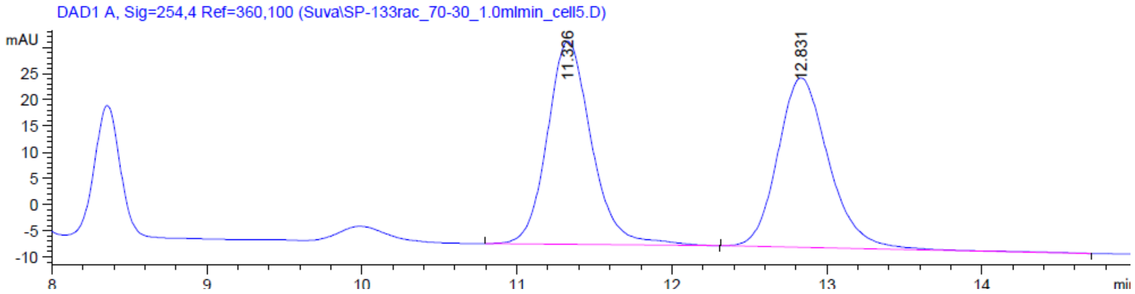

Peak #	RetTime [min]	Type	Width [min]	Area [mAU*s]	Height [mAU]	Area %
1	11.326	BB	0.3026	765.46954	38.72617	50.8649
2	12.831	BB	0.3510	739.43890	32.30672	49.1351

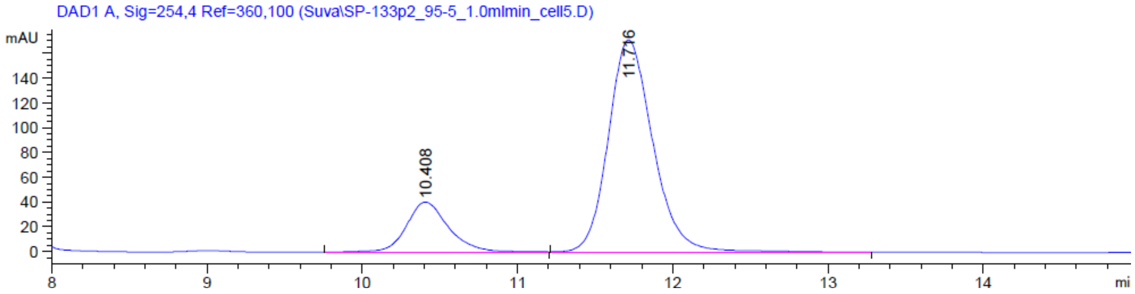

Peak #	RetTime [min]	Type	Width [min]	Area [mAU*s]	Height [mAU]	Area %
1	10.408	BB	0.2834	757.57202	40.28285	18.1250
2	11.716	BB	0.3052	3422.13745	171.18634	81.8750

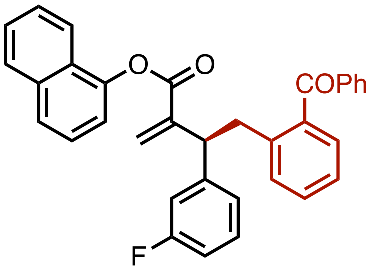

3j

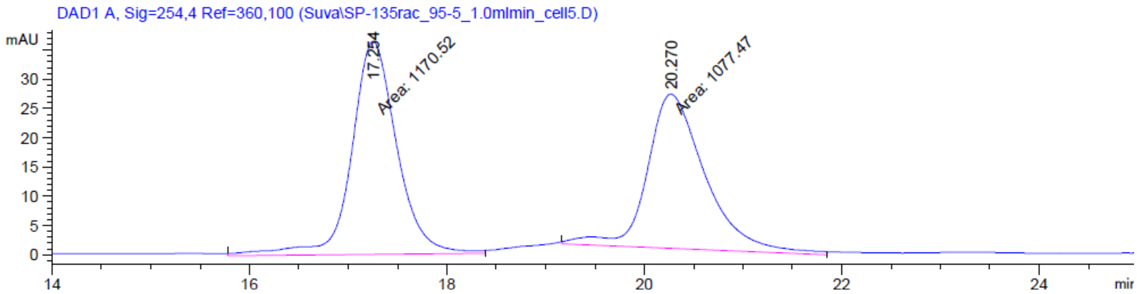

Peak #	RetTime [min]	Type	Width [min]	Area [mAU*s]	Height [mAU]	Area %
1	17.254	MM	0.5349	1170.52258	36.47438	52.0697
2	20.270	MM	0.6807	1077.46863	26.38059	47.9303

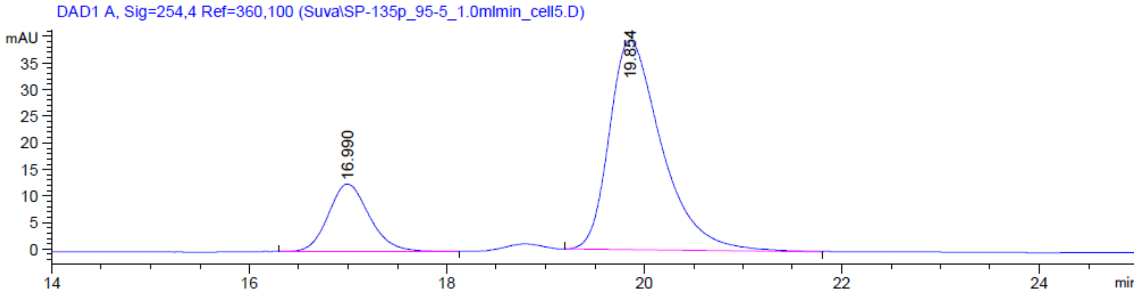

Peak #	RetTime [min]	Type	Width [min]	Area [mAU*s]	Height [mAU]	Area %
1	16.990	BB	0.4451	367.15152	12.70748	20.2171
2	19.854	BB	0.5593	1448.89636	39.37680	79.7829

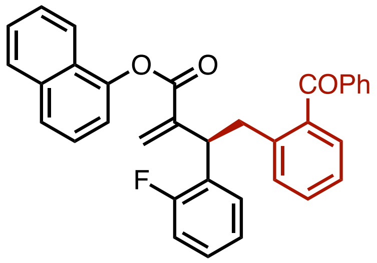

3k

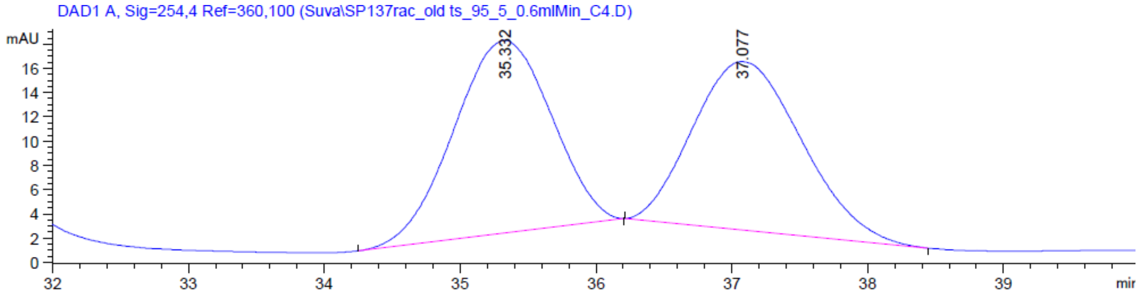

Peak #	RetTime [min]	Type	Width [min]	Area [mAU*s]	Height [mAU]	Area %
1	35.332	BB	0.7716	793.13129	15.90882	50.5961
2	37.077	BB	0.8391	774.44391	13.87766	49.4039

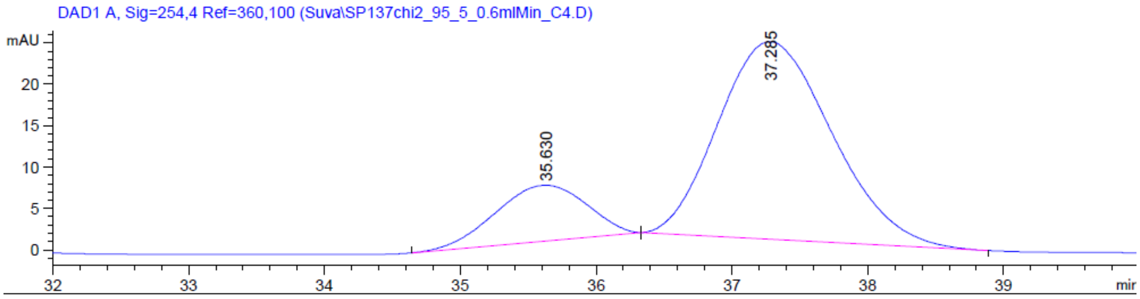

Peak #	RetTime [min]	Type	Width [min]	Area [mAU*s]	Height [mAU]	Area %
1	35.630	BB	0.7356	313.50244	6.72790	18.4291
2	37.285	BB	0.9048	1387.62927	23.84904	81.5709

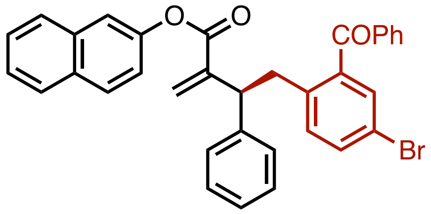

3l

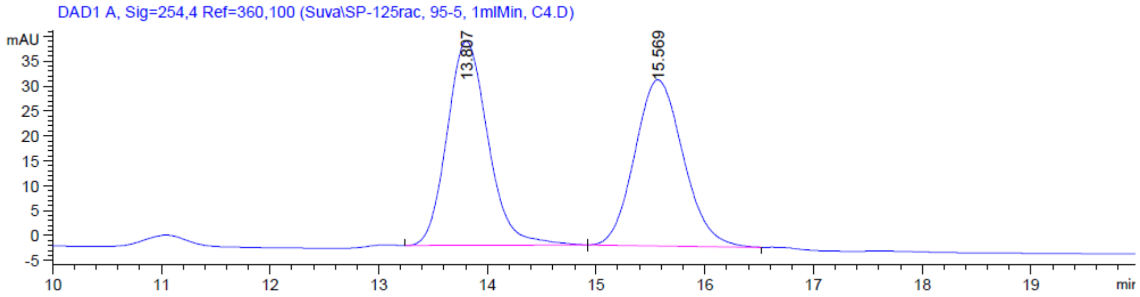

Peak #	RetTime [min]	Type	Width [min]	Area [mAU*s]	Height [mAU]	Area %
1	13.807	BB	0.4012	1072.34265	40.99566	50.9200
2	15.569	BB	0.4756	1033.59192	33.34778	49.0800

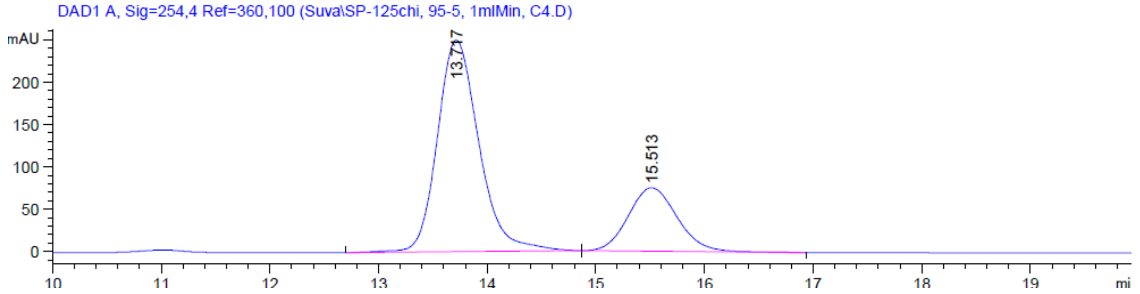

Peak #	RetTime [min]	Type	Width [min]	Area [mAU*s]	Height [mAU]	Area %
1	13.717	BB	0.4075	6631.46289	249.94769	73.9121
2	15.513	BB	0.4841	2340.62964	74.99515	26.0879

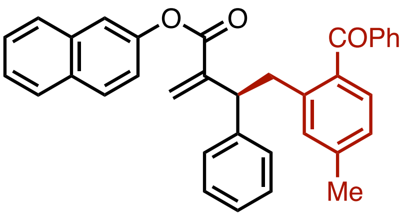

3m

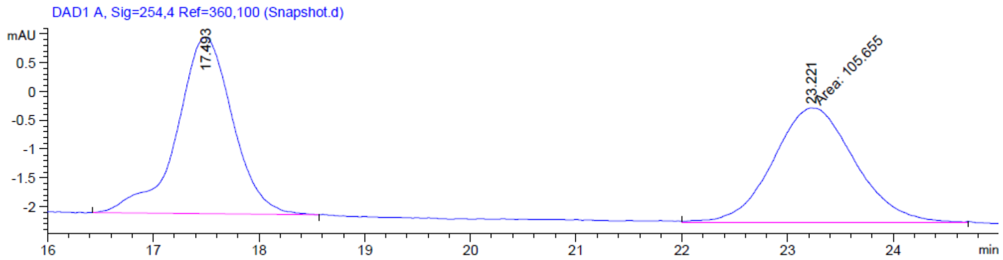

Peak #	RetTime [min]	Type	Width [min]	Area [mAU*s]	Height [mAU]	Area %
1	17.493	BB	0.5527	112.04498	3.06351	51.4675
2	23.221	MM	0.8896	105.65549	1.97938	48.5325

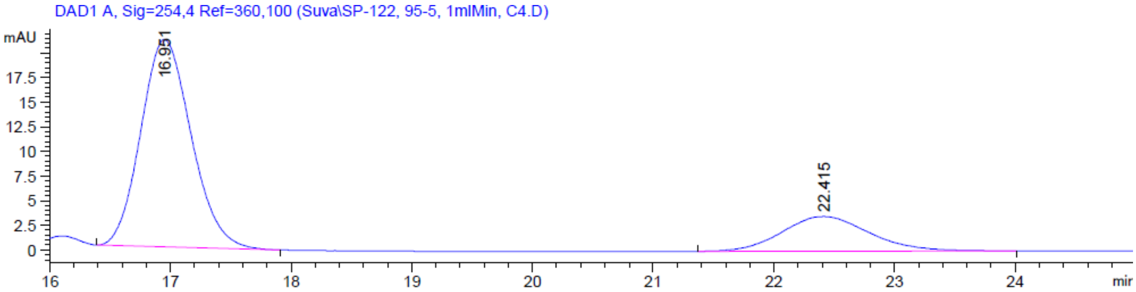

Peak #	RetTime [min]	Type	Width [min]	Area [mAU*s]	Height [mAU]	Area %
1	16.951	BB	0.4564	611.83734	21.09167	77.4953
2	22.415	BB	0.7018	177.67819	3.53874	22.5047

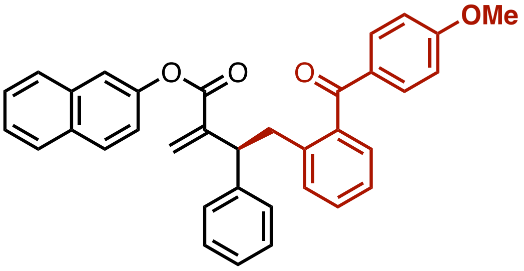

3n

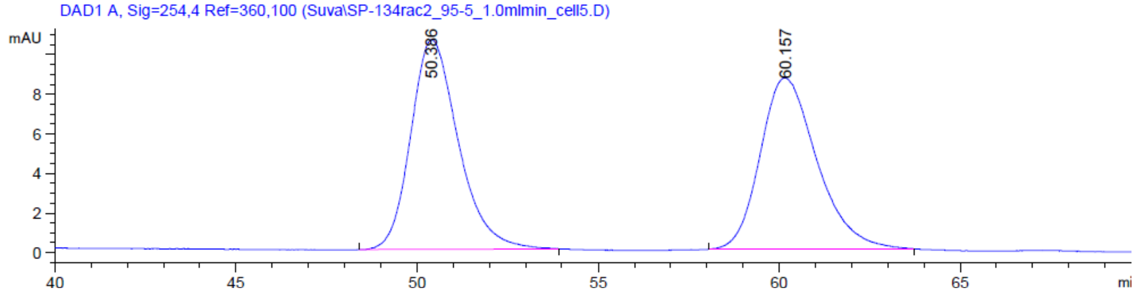

Peak #	RetTime [min]	Type	Width [min]	Area [mAU*s]	Height [mAU]	Area %
1	50.386	BB	1.1797	969.11493	10.60973	50.7225
2	60.157	BB	1.3240	941.50574	8.66249	49.2775

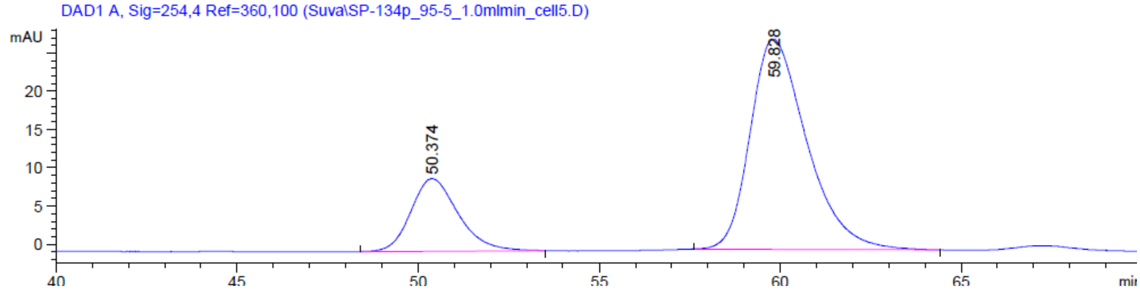

Peak #	RetTime [min]	Type	Width [min]	Area [mAU*s]	Height [mAU]	Area %
1	50.374	BB	1.1180	863.55078	9.50423	22.4007
2	59.828	BB	1.3886	2991.46411	27.46723	77.5993

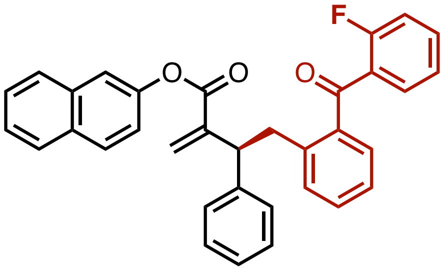

3o

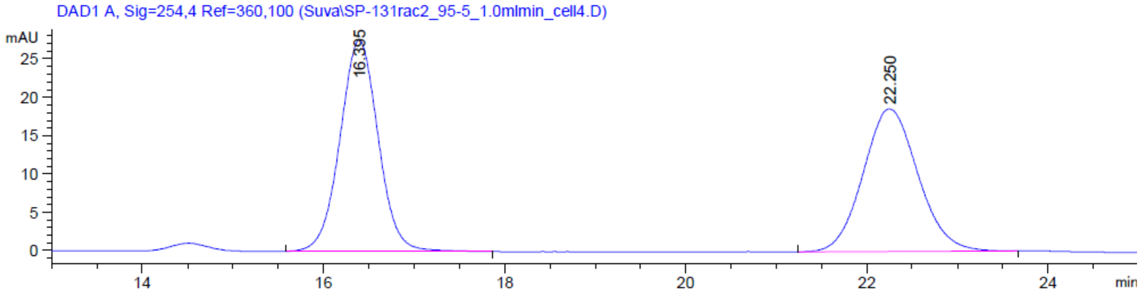

Peak #	RetTime [min]	Type	Width [min]	Area [mAU*s]	Height [mAU]	Area %
1	16.395	BB	0.4618	808.82037	27.60566	50.8339
2	22.250	BB	0.6316	782.28516	18.56698	49.1661

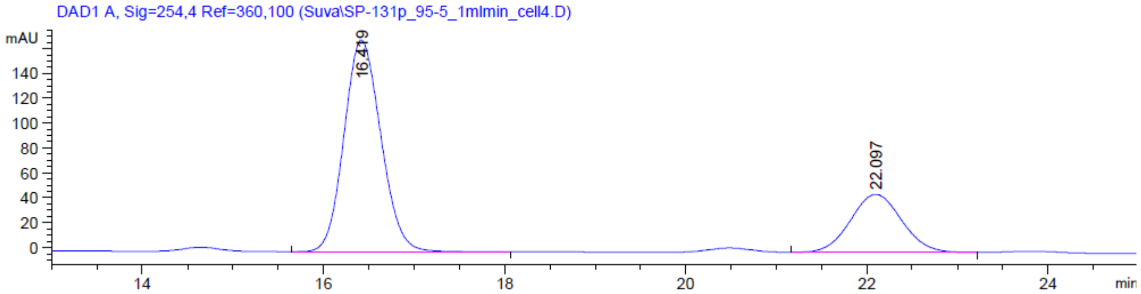

Peak #	RetTime [min]	Type	Width [min]	Area [mAU*s]	Height [mAU]	Area %
1	16.419	BB	0.4439	4807.10059	169.98293	72.3193
2	22.097	BB	0.6011	1839.94971	46.75963	27.6807

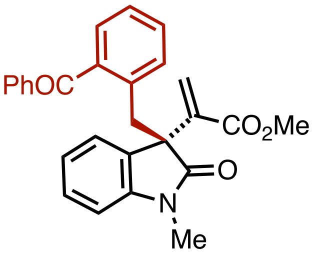

6a

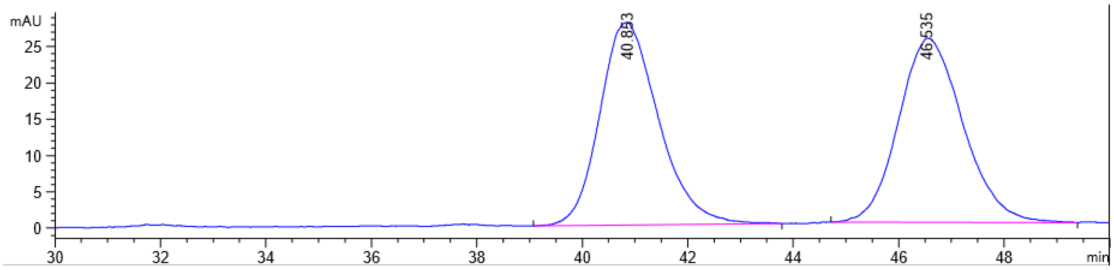

Peak #	RetTime [min]	Type	Width [min]	Area [mAU*s]	Height [mAU]	Area %
1	40.853	BB	1.0289	2170.60352	27.92491	50.1626
2	46.535	BB	1.0176	2156.53589	25.37279	49.8374

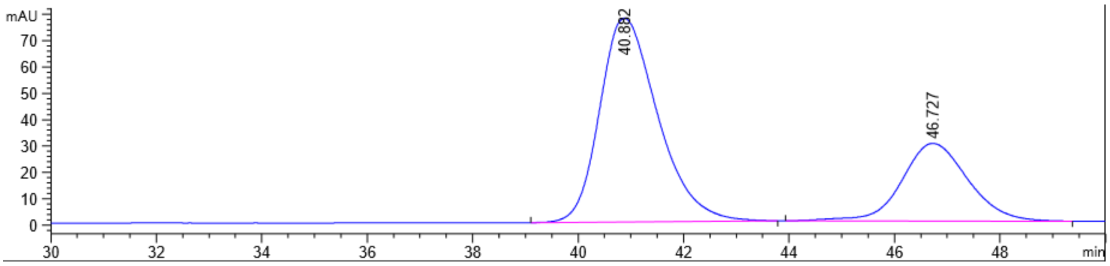

Peak #	RetTime [min]	Type	Width [min]	Area [mAU*s]	Height [mAU]	Area %
1	40.882	BB	1.1060	5935.52100	77.46069	69.7641
2	46.727	BB	1.2807	2572.47144	29.46126	30.2359

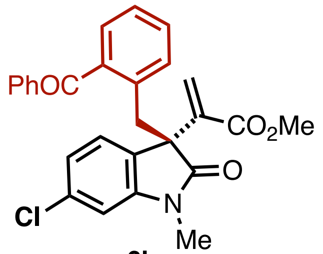

6b

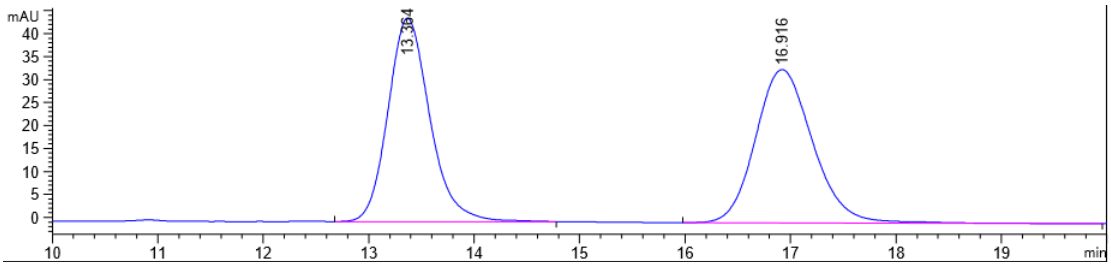

Peak #	RetTime [min]	Type	Width [min]	Area [mAU*s]	Height [mAU]	Area %
1	13.364	BB	0.4238	1221.61768	44.29236	49.1867
2	16.916	BB	0.5912	1262.01489	33.37462	50.8133

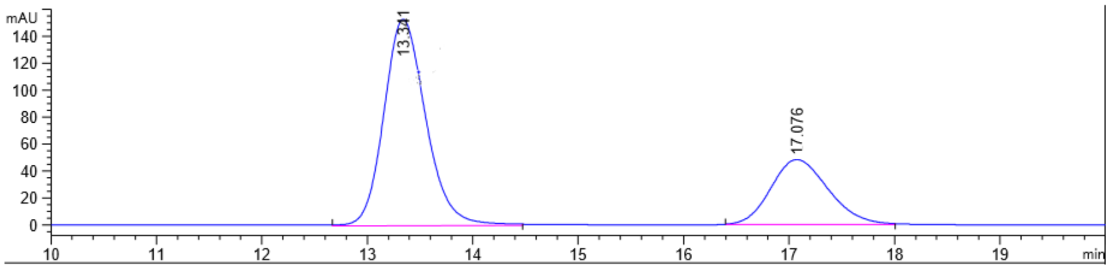

Peak #	RetTime [min]	Type	Width [min]	Area [mAU*s]	Height [mAU]	Area %
1	13.341	MM	0.4613	4239.04736	153.15678	70.1062
2	17.076	MM	0.6277	1807.55994	47.99307	29.8938

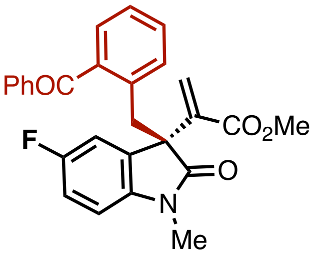

6c

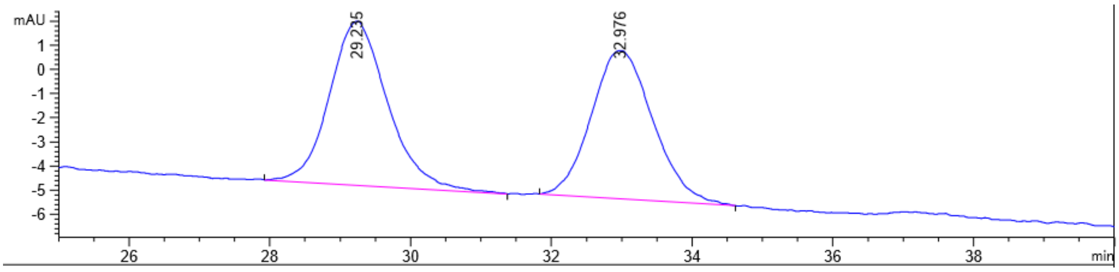

Peak #	RetTime [min]	Type	Width [min]	Area [mAU*s]	Height [mAU]	Area %
1	29.235	BB	0.8374	391.40472	6.80111	51.0273
2	32.976	BB	0.8350	375.64484	6.14667	48.9727

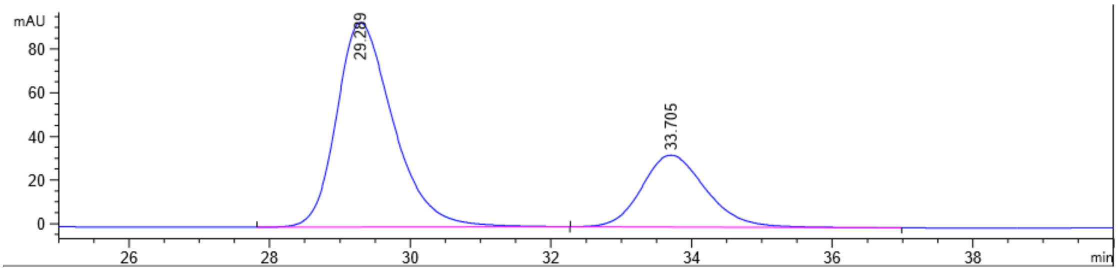

Peak #	RetTime [min]	Type	Width [min]	Area [mAU*s]	Height [mAU]	Area %
1	29.289	BB	0.8552	5254.84521	93.85522	71.5620
2	33.705	BB	0.9572	2088.22144	32.95712	28.4380

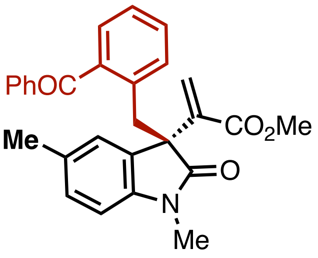

6d

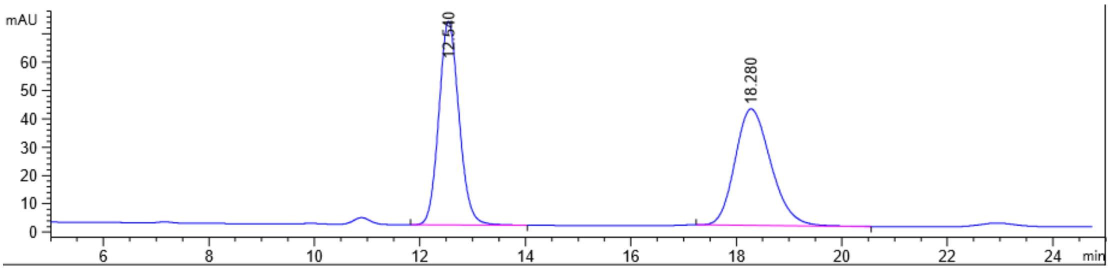

Peak #	RetTime [min]	Type	Width [min]	Area [mAU*s]	Height [mAU]	Area %
1	12.540	BB	0.4103	1911.80835	71.87636	49.6427
2	18.280	BB	0.7366	1939.32861	41.09378	50.3573

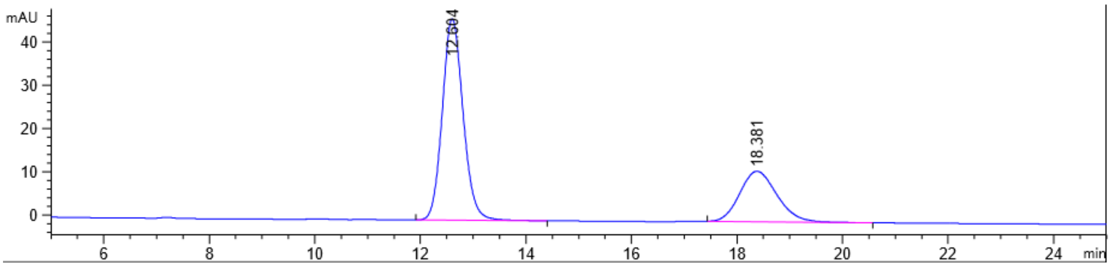

Peak #	RetTime [min]	Type	Width [min]	Area [mAU*s]	Height [mAU]	Area %
1	12.604	BB	0.4237	1274.77075	46.52517	69.0034
2	18.381	BB	0.7653	572.63123	11.65276	30.9966

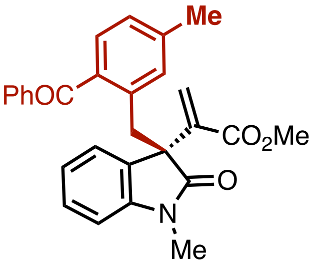

6e

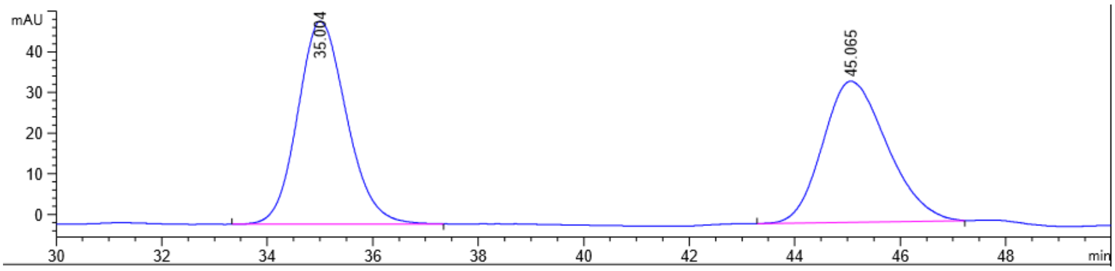

Peak #	RetTime [min]	Type	Width [min]	Area [mAU*s]	Height [mAU]	Area %
1	35.004	BB	0.9789	3212.99292	49.92331	52.1703
2	45.065	BB	1.1745	2945.67358	34.69617	47.8297

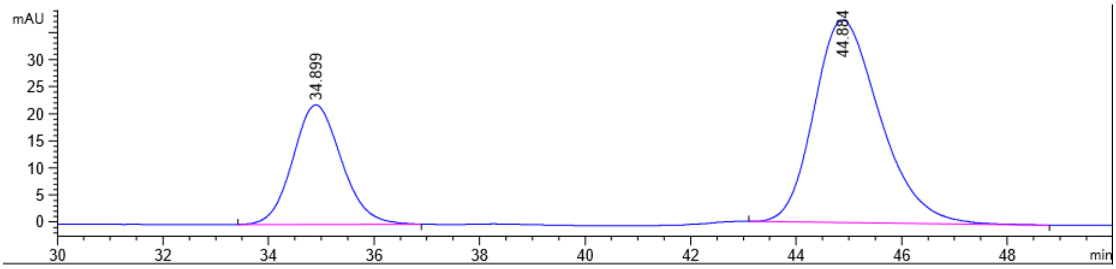

Peak #	RetTime [min]	Type	Width [min]	Area [mAU*s]	Height [mAU]	Area %
1	34.899	BB	0.9497	1415.66064	22.08766	30.2489
2	44.884	BB	1.3253	3264.38184	37.43648	69.7511

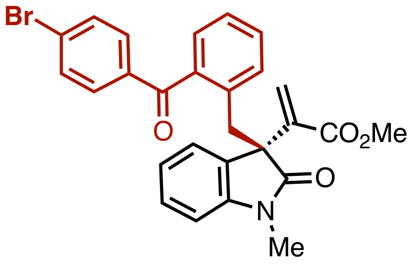

6g

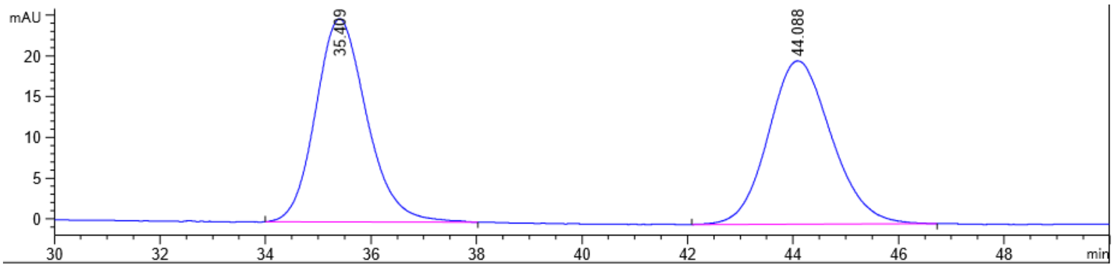

Peak #	RetTime [min]	Type	Width [min]	Area [mAU*s]	Height [mAU]	Area %
1	35.409	BB	0.9943	1681.88196	24.95560	50.1287
2	44.088	BB	1.1634	1673.24414	20.05376	49.8713

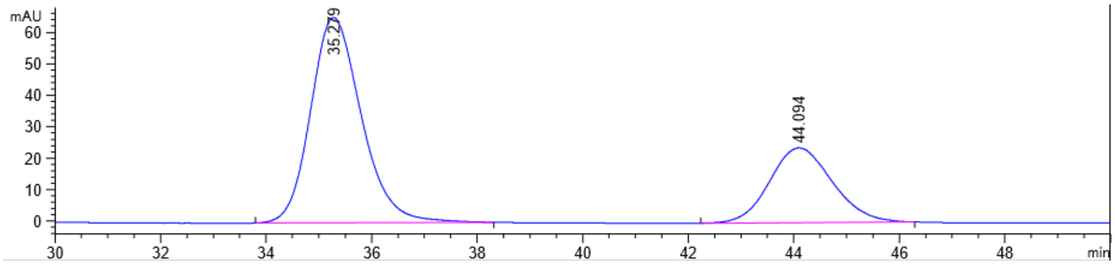

Peak #	RetTime [min]	Type	Width [min]	Area [mAU*s]	Height [mAU]	Area %
1	35.279	BB	1.0071	4370.07813	65.27428	68.9522
2	44.094	BB	1.1609	1967.76135	23.79143	31.0478

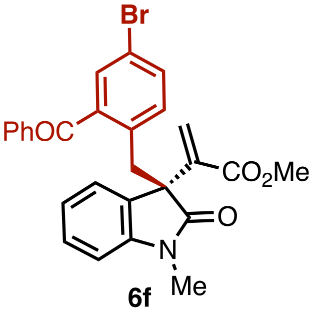

6f

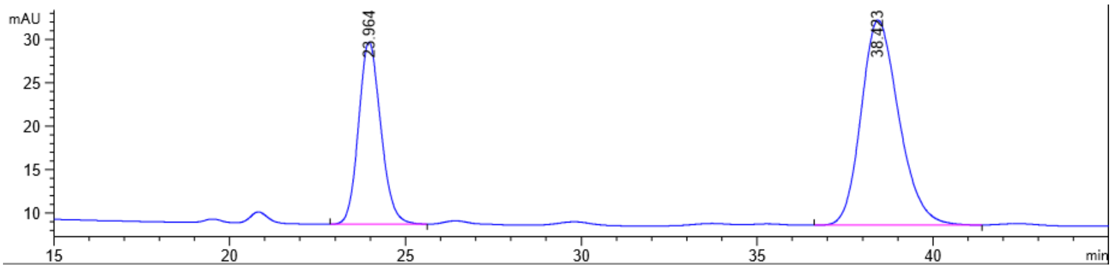

Peak #	RetTime [min]	Type	Width [min]	Area [mAU*s]	Height [mAU]	Area %
1	24.578	BB	0.6584	1141.74390	25.69363	49.9291
2	38.138	BB	1.0206	1144.98779	15.74059	50.0709

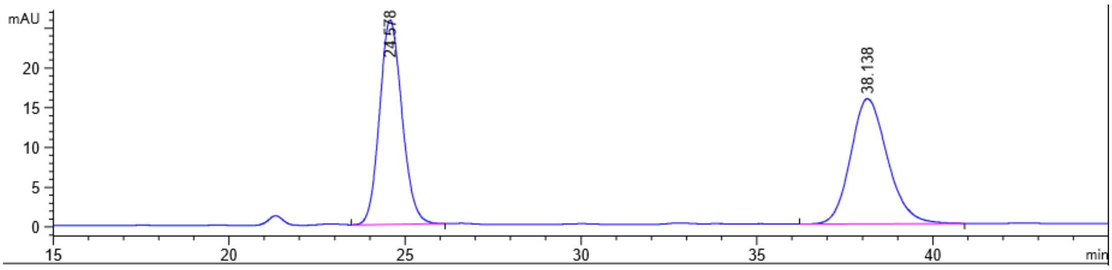

Peak #	RetTime [min]	Type	Width [min]	Area [mAU*s]	Height [mAU]	Area %
1	23.964	BB	0.6808	924.30701	20.94804	34.3835
2	38.423	BB	0.9852	1763.92322	23.62043	65.6165

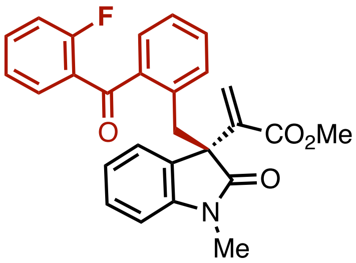

6h

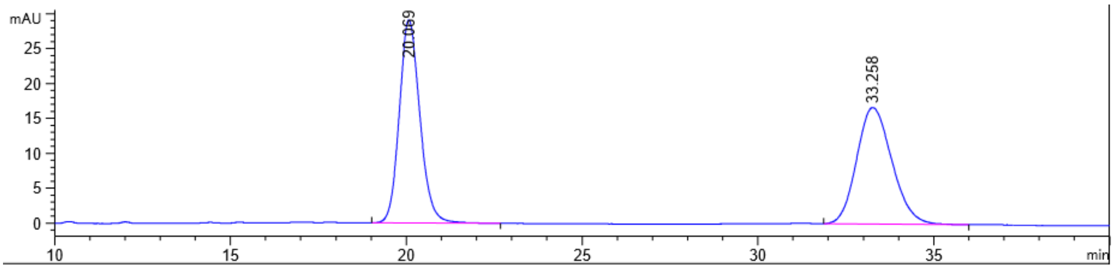

Peak #	RetTime [min]	Type	Width [min]	Area [mAU*s]	Height [mAU]	Area %
1	20.069	BB	0.6304	1187.71082	29.08329	50.4956
2	33.258	BB	1.0637	1164.39771	16.69535	49.5044

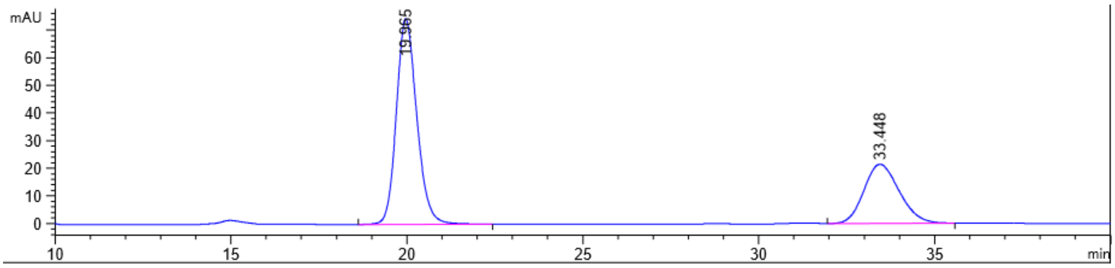

Peak #	RetTime [min]	Type	Width [min]	Area [mAU*s]	Height [mAU]	Area %
1	19.965	BB	0.6156	3008.31372	74.41463	66.8549
2	33.448	BB	1.0463	1491.45032	21.36810	33.1451

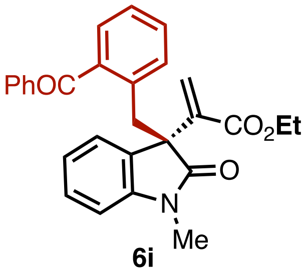

6i

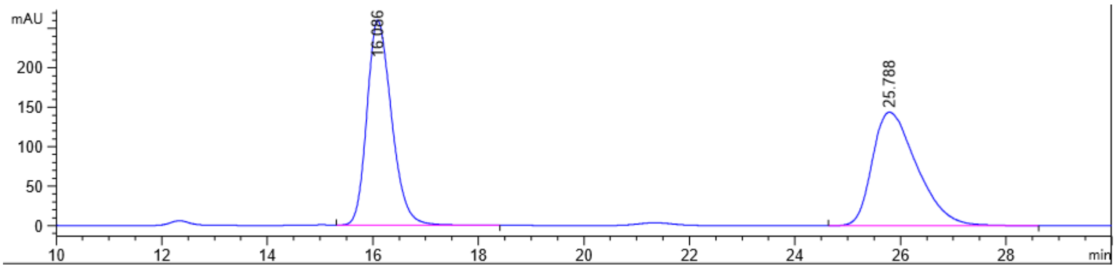

Peak #	RetTime [min]	Type	Width [min]	Area [mAU*s]	Height [mAU]	Area %
1	16.086	BB	0.5101	8434.07129	260.27405	49.9872
2	25.788	BB	0.7636	8438.38281	144.02235	50.0128

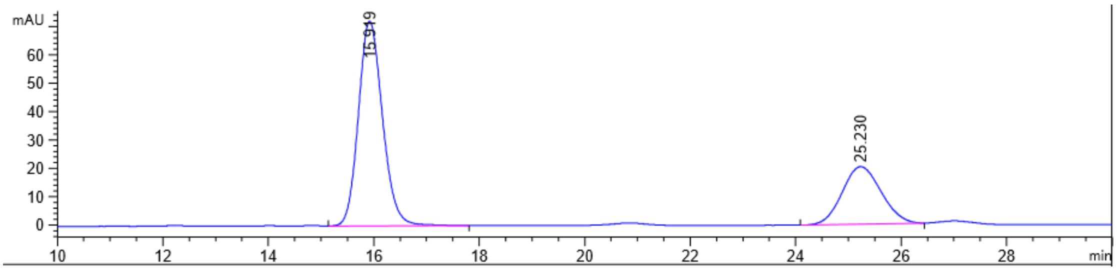

Peak #	RetTime [min]	Type	Width [min]	Area [mAU*s]	Height [mAU]	Area %
1	15.919	BB	0.4920	2288.85938	72.18044	68.7630
2	25.230	BB	0.7938	1039.76160	20.29088	31.2370

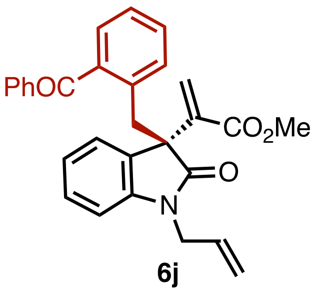

6j

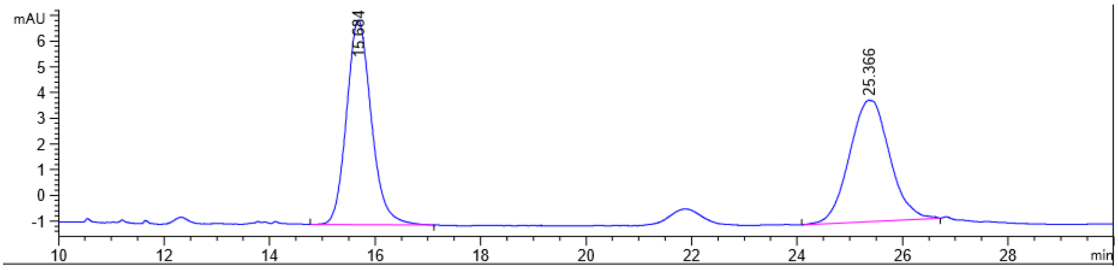

Peak #	RetTime [min]	Type	Width [min]	Area [mAU*s]	Height [mAU]	Area %
1	15.684	BB	0.5050	260.16006	7.96574	50.9707
2	25.366	BB	0.7218	250.25070	4.72569	49.0293

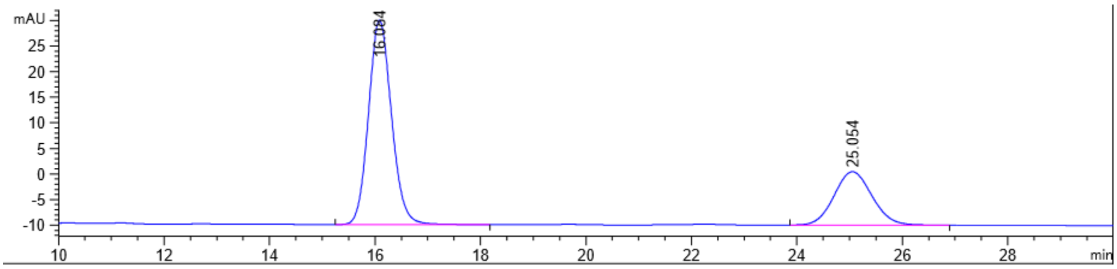

Peak #	RetTime [min]	Type	Width [min]	Area [mAU*s]	Height [mAU]	Area %
1	16.084	BB	0.4677	1212.86743	40.00750	69.9291
2	25.054	BB	0.7692	521.55707	10.43426	30.0709

H. References

[1] (a) Song, Y.: Wang, L.; Deng, L. *J. Am. Chem. Soc.* **2006**, *128*, 6048. (b) Hintermann, L.; Schmitz, M.; Englert, U. *Angew. Chem. Int. Ed.* **2007**, *46*, 5164. (c) Waldmann,H.; Khedkar, V.; Dückert, H. Schürmann, M.; Oppel, I. M. Kumar, I. M. *Angew. Chem. Int. Ed.* **2008**, *47*, 6869. (d) Martelli, G.; Orena, M.; Rinaldi, S. *Eur. J. Org. Chem.* **2012**, 4140. (e) Fan, X.; Yang, H.; Shi. M. *Adv. Synth. Catal.* **2017**, 359, 49. (e) Mato, R.; Manzano, R.; Reyes, E.; Carrillo, E.; Uria, U.; Vicario, J. L. *J. Am. Chem. Soc.* **2019**, *141*, 9495.

[2] Baidya, M.; Remennikov, G. Y.; Mayer, P.; Mayr, H. *Chem Eur. J.* **2010**, *16*, 1365.

[3] Shanmugam, P.; Viswambharan, B.; Selvakumar, K.; Madhavan, S. *Tetrahedron Lett.* **2008**, *49*, 2661.

[4] Dolomanov, O. V.; Bourhis, L. J.; Gildea, R. J.; Howard, J. A. K.; Puschmann, H.. *J. Appl. Crystallogr.* **2009**, *42*, 339. DOI: 10.1107/S0021889808042726.

[5] Sheldrick, G.M. *Acta Crystallogr. Sect. A Found. Adv.* **2015**, *71*, 3. DOI: 10.1107/S2053273314026370

[6] Sheldrick, G.M. *Acta Crystallogr. Sect. C Struct. Chem.* **2015**, *71*, 3. DOI: 10.1107/S2053229614024218.

[7] For a review see: Flack, H. D.; Bernardinelli, G. *Chirality*, **2008**, *20*, 681.

[8] For reviews, see: a) Bringmann, G.; Bruhn, T.; Maksimenka, K.; Hemberger, Y. *Eur. J. Org. Chem.* **2009**, 2717. b) Crawford, T. D.; Tam, M. C.; Abrams, M. L. *J. Chem. Phys. A* **2007**, *111*, 12057. c) Pescitelli, G.; Di Bari, L.; Berova, N. *Chem. Soc. Rev.* **2011**, *40*, 4603. d) Mazzanti, A.; Casarini, D. *WIRES Comput. Mol. Sci.* **2012**, *2*, 613 e) Superchi, S.; Scafato, P.; Górecki, M.; Pescitelli, G. *Curr. Med. Chem.* **2018**, *25*, 287.

[9] Marenich, A.V.; Cramer, C.J.; Truhlar, D.G. *J. Phys. Chem. B* **2009**, *113*, 6378.

[10] Grimme, S.; Antony, J.; Ehrlich S.; Krieg, H. *J. Chem. Phys.*, **2010**, *132*, 154104.

[11] Chai, J-D.; Head-Gordon, M. *Phys. Chem. Chem. Phys.*, **2008**, *10*, 6615.

[12] Yanai, T.; Tewand, D.; Handy, N. *Chem. Phys. Lett.* **2004**, *393*, 51.

[13] a) Mancinelli, M.; Franzini, R.; Renzetti, A.; Marotta, E.; Villlani, C.; Mazzanti, A. *RSC Adv.*, **2019**, *9*, 18165. b) Mancinelli, M.; Perticarari, S.; Prati, L.; Mazzanti, A. *J. Org. Chem.* **2017**, *82*, 6874. c) Meazza, M.; Light, M. E.; Mazzanti A.; Rios, R. *Chem. Sci.* **2016**, *7*, 984.

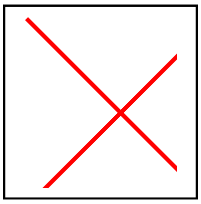

Supplement: Supplementary file 1 — jo0c00175_si_001.pdf [file jo0c00175_si_001.pdf]
